# Loneliness 5 years ante-mortem is associated with disease-related differential gene expression in postmortem dorsolateral prefrontal cortex

**DOI:** 10.1038/s41398-017-0086-2

**Published:** 2018-01-10

**Authors:** Turhan Canli, Lei Yu, Xiaoqing Yu, Hongyu Zhao, Debra Fleischman, Robert S. Wilson, Philip L. De Jager, David A. Bennett

**Affiliations:** 1 Departments of Psychology and Psychiatry, Stony Brook University, Stony Brook, NY USA; 2 Rush Alzheimer’s Disease Center, Department of Neurological Sciences, Rush University Medical Center, Chicago, IL USA; 3 Biostatistics Department, Yale School of Public Health, Yale University, New Haven, CT USA; 4 Department of Neurology, Columbia University, New York, NY USA

## Abstract

Subjective social isolation, loneliness, is associated with poor mental and physical health, but the underlying molecular mechanisms are poorly understood. Here we analyzed loneliness data collected on average 5 years ante-mortem and RNA gene expression at death in postmortem dorsolateral prefrontal cortex (DLPFC) from 181 participants in the Rush Memory and Aging Project (MAP), a longitudinal, prospective cohort study of common chronic conditions of aging. Our analytic protocol controlled for biographical variables (age, sex, education), psychological and health variables (depressive symptoms, interval between assessment and autopsy, slope of cognitive decline, AD pathology, presence of infarcts) and RNA integrity. Our results are based on a pre-ranked Gene Set Enrichment Analysis (GSEA) at FDR-corrected q-values <0.05, using these collections from the Molecular Signatures Database (v6.0 MSigDB): (1) Hallmarks, (2) Canonical, (3) Gene Ontology (GO), (4) Chemical and Genetic Perturbations, (5) Immunologic Signatures, (6) Oncogenic Signatures, and (7) Cancer Modules. We now report on 337 up-regulated and 43 down-regulated gene sets, among which the most significant ones were associated with Alzheimer’s disease, psychiatric illness, immune dysfunction, and cancer. These gene sets constitute attractive targets for future studies into the molecular mechanisms by which loneliness exacerbates a wide range of neurodegenerative, psychiatric, and somatic illnesses.

## Introduction

Loneliness is the subjective perception of social isolation^1^, which has been consistently associated with increased morbidity and mortality^2–4^. Loneliness is associated with poor mental health, most prominently depression^5–10^, but also with poor physical health^11^. Loneliness has been linked to accelerated decline of cognitive functions^12–14^, incident dementia^15^, and incident Alzheimer’s disease (AD)^13^, inflammatory diseases^16–18^, and cancer^19,20^.

One proposed mechanism by which loneliness may diminish health invokes a psychosocial stress model^2^ by which physiological stress triggers the release of catecholamines and glucocorticoids, the latter of which are known to place an allostatic load on the body^21^. Such physiological stress response signals have the capacity to regulate gene expression. For example, glucocorticoids can regulate gene expression by binding to glucocorticoid response elements (GREs) via glucocorticoid receptors (GRs). In support of this hypothesis, analyses of differentially expressed genes (DEGs) in blood leukocytes identified many genes which contain GRE binding sequences^22,23^.

Yet, many genes associated with common chronic conditions and cognitive decline do not contain GREs, suggesting that additional mechanisms may link loneliness to these disease states. Furthermore, given that loneliness is a subjective experience, gene expression profiling within relevant neural circuits may be required to elucidate these mechanisms. We recently reported on DEGs in postmortem nucleus accumbens^24^, motivated by a functional magnetic resonance imaging (fMRI) study^25^. On the basis of 26 donors with known loneliness phenotypes, gene set enrichment analysis (GSEA) identified significant enrichment for Alzheimer’s disease (AD), as well as significant pleiotropy among DEGs associated with neurological or behavioral disorders, inflammatory diseases, and cancer, among others. Network analyses revealed extensive links between these DEGs and upstream regulators, as well as networks among those regulators. The data suggested that seemingly unrelated health conditions exacerbated by loneliness were linked by an underlying regulatory gene network structure. It is unknown whether this pattern of gene expression is specific to nucleus accumbens or recapitulated in other brain regions.

Here, we report on DEGs in the dorsolateral prefrontal cortex (DLPFC), which is also a well-suited region for examination of gene expression as a function of loneliness. First, voxel-based morphometry data from a relatively large structural imaging study revealed increased regional gray matter volume in the DLPFC as a function of loneliness^26^.

Second, the DLPFC (and other cortical and limbic regions and connecting pathways) is associated with depression. For example, along with the ventromedial PFC, the DLPFC is engaged during processing of negative emotional information and cognitive control of emotion that requires reappraisal or suppression of emotional information^27^. On the basis of neuroimaging, patient lesion, and brain stimulation studies, these two regions are believed to play distinct roles^28^, with VMPFC involved in the generation of negative affect or self-awareness and reflection, and the DLPFC involved in the regulation of negative affect. Indeed, depressed patients exhibit reduced activity in the DLPFC during voluntary, conscious (as opposed to automatic, non-conscious) emotion regulation^27,29,30^. Two fMRI resting-state imaging studies using an analysis technique to identify abnormal regional synchronization reported abnormal DLPFC activity in elderly patients with late-life depression^31^ and late-life subthreshold depression^32^, relative to healthy controls. A voxel-based, whole-brain meta-analysis^33^ confirmed reduced activation of the DLPFC in depressed patients, relative to healthy controls, during the processing of negative emotional stimuli, which is then placed within a larger context of dysfunctional processing within cortical–striatal–pallidal–thalamic circuitry. Finally, patients with treatment-resistant depression who are treated with high-frequency transcranial magnetic stimulation directed at the left DLPFC show individual differences in treatment outcome, and responders were characterized by higher brain glucose metabolism at baseline in the DLPFC^34^.

Third, the DLPFC is associated with cognitive functions that are impaired in lonely individuals. For example, in a longitudinal study, loneliness was associated with lower levels of perceptual processing, visuospatial ability, and episodic, semantic, and working memory at baseline^13^, which requires the DLPFC^35^. Furthermore, elderly patients with late-onset depression exhibit reduced functional connectivity between cerebellum and DLPFC and posterior cingulate cortex, compared to healthy controls, and the degree of functional connectivity correlated with performance in the mini-mental state examination (MMSE)^36^. Loneliness is also associated with increased risk for AD^13^, and apolipoprotein E (*APOE*) *ε4* haplotype is a risk-factor for late-onset AD^37^. Disease progression correlates negatively with cortical thickness^38^, and DLPFC thickness is reduced in *ε4* carriers compared to *ε2* carriers^39^.

In this study, we examined RNAseq data from the DLPFC^40^ to identify gene sets associated with loneliness in 181 brain donors with known loneliness scores. These donors had participated in the Rush Memory and Aging Project (MAP), a longitudinal prospective cohort study of common chronic conditions of aging, in which participants agree to an annual detailed evaluation and organ donation at the time of death^41^.

## Materials/subjects and methods

### Subjects

All brain donors were participants in the Rush MAP, a prospective cohort study of common chronic conditions of aging^41^, as previously reported^24^. The study was approved by the Institutional Review Board of Rush University Medical Center and all subjects signed an informed consent and Anatomical Gift Act.

At the time of the generation of RNAseq data, 675 MAP participants had died and undergone brain autopsy. Of those, a sample of 181 (119 Females) individuals had both available RNAseq data that passed quality control and baseline measures of loneliness, as reported^13,42^.

### Self-report measures

Self-reported loneliness scores from baseline were used in the analyses, as previously reported^24^. Participants at baseline were relatively healthy, e.g., without dementia, and thus the baseline measure represents self-reported loneliness when participants were most cognitively intact. Each participant’s loneliness score was averaged from five response items scored on a 5-point Likert scale (1=strongly disagree, 5=strongly agree): 1. “I experience a general sense of emptiness”; 2. “I miss having people around”; 3. “I feel like I don’t have enough friends”; 4. “I often feel abandoned”; 5. “I miss having a really close friend”, as previously described^13^.

### Brain tissue

At autopsy, the brain was hemisected, with one hemisphere to be the source of gene-expression studies cut into 1 cm slabs in a plexiglass jig, put into individual freezer bags, placed on a metal plate, and put into a −80° freezer. A complete neuropathologic evaluation was performed that included the determination of global burden of AD pathology as described^43^.

### RNA extraction, preparation sequencing, and data processing

The methods for RNAseq protocols (sample extraction, library preparation, sequencing, and data processing) are provided online at https://www.synapse.org/#!Synapse:syn3388564. We briefly summarize these steps below.

#### Sample extraction

DLPFC samples were extracted using Qiagen’s miRNeasy mini kit (cat. no. 217004) and the RNase free DNase Set (cat. no. 79254; Qiagen, Hilden, Germany). These samples were quantified by Nanodrop (Wilmington, DE, USA) and quality was evaluated by Agilent Bioanalyzer (Agilent Technologies, Santa Clara, CA, USA).

#### Library preparation

The Broad Institute’s Genomics Platform performed RNA-Seq library preparation using the strand specific dUTP method^44^ with poly-A selection^45^. RNA-Seq data met quality (Bioanalyzer RNA integrity (RIN) score >5) and quantity thresholds (5 μg).

#### Sequencing

Sequencing was performed on the Illumina (Illumina Inc., San Diego, CA, USA) HiSeq with 101 bp paired-end reads and achieved coverage of 150 M reads of the first 12 samples. These 12 samples served as a deep coverage reference and included 2 males and 2 females of non-impaired, mild cognitive impaired, and Alzheimer’s cases. This was batch “0”. The remaining samples were sequenced with coverage of 50 M reads.

#### Data processing

RNA-Seq data were processed by our parallelized and automatic pipeline, which included trimming the beginning and ending bases from each read, identifying and trimming adapter sequences from reads, detecting and removing rRNA reads, aligning reads to reference genome. We used the non-gapped aligner Bowtie to align reads to transcriptome reference and then applied RSEM to estimate expression levels for all transcripts. The FPKM values were the outcome of our data RNA-Seq pipeline. We applied quantile normalization method to FPKM first and then used combat package to remove potential batch effect.

### Statistical analyses of RNA expression and loneliness

Analysis followed a two-step process. We first conducted a multiple regression analysis for each gene separately based on the following model: Gene expression~age+sex+education+interval between assessment and autopsy+slope for cognitive decline+AD pathology+presence of infarcts+RIN score+depressive symptoms+loneliness. This analysis provided a list of *t*-scores and corresponding *p*-values for loneliness in relation to each of the gene’s expression values, of which there were 1547 up-regulated and 1254 down-regulated genes at *p* < 0.05, with regression coefficients (column “Estimate” in Supplementary Table 1) capturing the magnitude (up-regulated if positive and down-regulated if negative) of individual gene expression with every 1 unit increase in our loneliness measure.

In a second step, gene set enrichment analysis was performed in GSEAPreranked (GSEAPreranked v3.0,^46,47^: 1000 permutations; enrichment statistic = weighted; max size = 2000; min size = 10; normalization mode = meandiv; ranked by t-score), based on genes that were either up- or down-regulated at *p* < 0.05, using these collections of gene sets from the Molecular Signatures Database (v6.0 MSigDB): (1) Hallmarks, (2) Canonical, (3) Gene Ontology (GO), (4) Chemical and Genetic Perturbations, (5) Immunologic Signatures, (6) Oncogenic Signatures, and (7) Cancer Modules. We only considered gene sets significantly enriched that met a threshold of FDR-corrected q-values <0.05.

## Results

Table 1 illustrates demographic characteristics of this sample with respect to measures of age at death, sex, education, depressive symptoms (as measured with CES-D), loneliness at baseline and last visit, time between baseline loneliness and autopsy, RNA integrity number (RIN) score, annual rate of cognitive decline, burden of Alzheimer’s disease (AD) pathology, and presence of chronic infarcts.

**Table 1:** Characteristics of the study subjects (*N*=181)

Age at death in years (mean, SD)	89.5, 6.2
Female Sex (*N*, %)	119, 65.8%
Education (mean, SD)	14.7, 2.7
CES-D (median, IQR)	1.0, 0-2.0
Baseline loneliness (mean, SD)	2.3, 0.7
Last loneliness measure prior to death (mean, SD)	2.5, 0.7
Time interval between baseline loneliness to autopsy (mean, SD)	4.9, 2.0
RIN scores (mean, SD)	7.1, 1.0
Annual rate of cognitive decline (mean, SD)	−0.015, 0.091
Burden of AD pathology	0.65, 0.37
Presence of chronic infarcts	75, 41.4%

*SD* Standard deviation, *IQR* Interquartile range

Across all MSigDB collections used here, there were 337 upregulated and 43 downregulated gene sets that showed significant enrichment. The complete set is provided in Supplementary Table 2, and the Top-25 up- and down-regulated gene sets, based on normalized Enrichment Score, are shown in Table 2. Gene sets previously shown to be up- and down-regulated in AD (“Blalock Alzheimer’s disease up” and “Blalock Alzheimer’s disease down”, respectively) were the most enriched sets for up- and down-regulated genes, respectively. Nine out of the Top-25 up-regulated gene sets belonged to the MSigDB collection of Cancer Modules. Nine out of the Top-25 down-regulated gene sets were associated with the aging brain, behavior, and neuronal or synaptic processes.

**Fig. 1:**
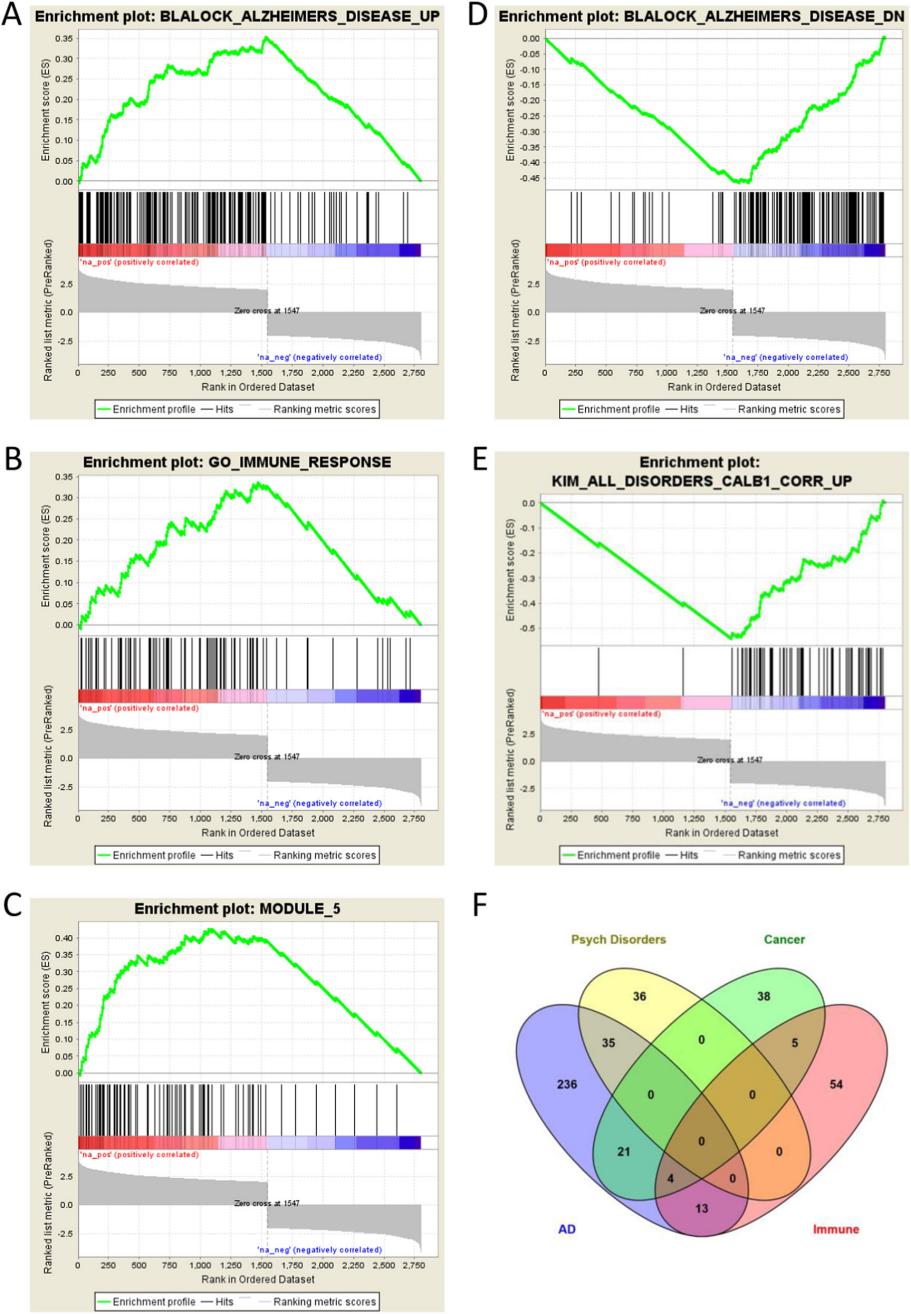
Up- and down-regulated Enrichment Plots for a priori gene sets AD, psychiatric disorders, immune function, and cancer (Panels **a**–**e**). Venn diagram illustrating the overlap between the gene sets depicted in **a**–**e**

Figure 1a–e shows the up- and down-regulated enrichment plots for selected a priori gene sets of interest from Table 2, because they are associated with conditions and diseases for which we previously reported differential gene expression in the human brain^24^, specifically: AD, psychiatric disorders, immune function, and cancer. Supplementary Tables 3–7 list the genes that made up the enrichment analyses for these gene sets. Figure 1f illustrates the overlap between the gene sets depicted in Fig. 1a–e (combining AD up- and down-regulated genes), based on significantly enriched genes only. There were thirteen highly pleiotropic genes that appeared across all four sets: ABL1, CD58, CD86, CTSH, GTPBP1, HFE, IFNA16, IL18, MASP1, MR1, PLCG2, TAPBP, TRIM38.

**Table 2:** Top-25 up- and down-regulated gene sets, based on normalized Enrichment Score

Collection	Upregulated Sets (sorted by positive NES)	SIZE	ES	NES	NOM p-val	FDR q-val	FWER p-val	RANK AT MAX	LEADING EDGE
ChemGenPerturb	BLALOCK_ALZHEIMERS_DISEASE_UP	198	0.35	4.28	0.000	0.000	0.000	1535	tags=87%, list=55%, signal=179%
CancerModules	MODULE_5	90	0.43	3.94	0.000	0.000	0.000	1067	tags=76%, list=38%, signal=118%
ChemGenPerturb	MILI_PSEUDOPODIA_HAPTOTAXIS_DN	98	0.38	3.60	0.000	0.000	0.000	1228	tags=79%, list=44%, signal=135%
ChemGenPerturb	REN_ALVEOLAR_RHABDOMYOSARCOMA_DN	65	0.43	3.55	0.000	0.000	0.000	1307	tags=88%, list=47%, signal=161%
CancerModules	MODULE_23	89	0.37	3.55	0.000	0.000	0.000	874	tags=63%, list=31%, signal=89%
CancerModules	MODULE_88	112	0.33	3.42	0.000	0.000	0.000	882	tags=60%, list=31%, signal=84%
CancerModules	MODULE_60	79	0.39	3.38	0.000	0.000	0.000	1425	tags=87%, list=51%, signal=173%
GO	GO_VASCULATURE_DEVELOPMENT	86	0.37	3.33	0.000	0.000	0.000	1545	tags=91%, list=55%, signal=196%
CancerModules	MODULE_55	108	0.33	3.29	0.000	0.000	0.000	882	tags=59%, list=31%, signal=83%
CancerModules	MODULE_38	89	0.36	3.29	0.000	0.000	0.000	1029	tags=67%, list=37%, signal=103%
CancerModules	MODULE_6	67	0.40	3.29	0.000	0.000	0.000	1096	tags=75%, list=39%, signal=120%
CancerModules	MODULE_84	76	0.38	3.26	0.000	0.000	0.000	1029	tags=70%, list=37%, signal=107%
ChemGenPerturb	LEE_BMP2_TARGETS_UP	112	0.31	3.24	0.000	0.000	0.000	1116	tags=67%, list=40%, signal=107%
CancerModules	MODULE_1	69	0.38	3.24	0.000	0.000	0.000	1102	tags=74%, list=39%, signal=119%
GO	GO_CELL_SURFACE	115	0.32	3.24	0.000	0.000	0.000	1008	tags=63%, list=36%, signal=95%
ChemGenPerturb	CHEN_METABOLIC_SYNDROM_NETWORK	172	0.27	3.14	0.000	0.000	0.000	871	tags=52%, list=31%, signal=71%
GO	GO_BLOOD_VESSEL_MORPHOGENESIS	64	0.39	3.12	0.000	0.000	0.000	1545	tags=92%, list=55%, signal=201%
GO	GO_IMMUNE_RESPONSE	90	0.34	3.11	0.000	0.000	0.000	1469	tags=84%, list=52%, signal=172%
Immunogenic	GSE21360_NAIVE_VS_PRIMARY_MEMORY_CD8_TCELL_UP	28	0.54	3.10	0.000	0.001	0.001	1230	tags=96%, list=44%, signal=170%
ChemGenPerturb	LEI_MYB_TARGETS	44	0.44	3.09	0.000	0.000	0.000	1212	tags=84%, list=43%, signal=146%
ChemGenPerturb	BLALOCK_ALZHEIMERS_DISEASE_INCIPIENT_UP	56	0.41	3.08	0.000	0.000	0.000	845	tags=64%, list=30%, signal=90%
GO	GO_CIRCULATORY_SYSTEM_DEVELOPMENT	130	0.29	3.05	0.000	0.000	0.000	1137	tags=65%, list=41%, signal=104%
GO	GO_EXTRACELLULAR_STRUCTURE_ORGANIZATION	45	0.44	3.05	0.000	0.000	0.000	1025	tags=78%, list=37%, signal=121%
ChemGenPerturb	MEISSNER_BRAIN_HCP_WITH_H3K4ME3_AND_H3K27ME3	155	0.27	3.04	0.000	0.000	0.000	1058	tags=59%, list=38%, signal=90%
ChemGenPerturb	JOHNSTONE_PARVB_TARGETS_3_UP	60	0.39	3.03	0.000	0.000	0.000	1081	tags=73%, list=39%, signal=117%
Collection	Downregulated Sets (sorted by negative NES)	SIZE	ES	NES	NOM p-val	FDR q-val	FWER p-val	RANK AT MAX	LEADING EDGE
ChemGenPerturb	BLALOCK_ALZHEIMERS_DISEASE_DN	158	−0.46	−5.89	0.000	0.000	0.000	1205	tags=87%, list=43%, signal=144%
ChemGenPerturb	KIM_ALL_DISORDERS_CALB1_CORR_UP	73	−0.54	−5.00	0.000	0.000	0.000	1248	tags=97%, list=45%, signal=171%
ChemGenPerturb	MILI_PSEUDOPODIA_HAPTOTAXIS_UP	48	−0.43	−3.30	0.000	0.000	0.000	1163	tags=83%, list=42%, signal=140%
ChemGenPerturb	SHEN_SMARCA2_TARGETS_UP	28	−0.53	−3.28	0.000	0.000	0.000	1243	tags=96%, list=44%, signal=172%
GO	GO_SYNAPSE_PART	101	−0.29	−3.11	0.000	0.000	0.000	1246	tags=73%, list=44%, signal=127%
ChemGenPerturb	SCHLOSSER_MYC_TARGETS_REPRESSED_BY_SERUM	19	−0.60	−3.11	0.000	0.000	0.002	1120	tags=100%, list=40%, signal=165%
ChemGenPerturb	HAMAI_APOPTOSIS_VIA_TRAIL_UP	52	−0.38	−3.10	0.000	0.000	0.002	1285	tags=83%, list=46%, signal=150%
Oncogenic	KRAS.KIDNEY_UP.V1_UP	27	−0.50	−3.06	0.000	0.000	0.000	1199	tags=93%, list=43%, signal=160%
ChemGenPerturb	LU_AGING_BRAIN_DN	25	−0.53	−3.04	0.000	0.000	0.002	1231	tags=96%, list=44%, signal=170%
GO	GO_SYNAPSE	117	−0.27	−3.04	0.000	0.000	0.000	1246	tags=71%, list=44%, signal=122%
Oncogenic	CAHOY_NEURONAL	18	−0.60	−3.02	0.000	0.000	0.000	995	tags=94%, list=36%, signal=146%
ChemGenPerturb	SENGUPTA_NASOPHARYNGEAL_CARCINOMA_WITH_LMP1_U	P 37	−0.43	−2.99	0.000	0.000	0.002	1160	tags=84%, list=41%, signal=141%
ChemGenPerturb	VERHAAK_GLIOBLASTOMA_PRONEURAL	29	−0.46	−2.79	0.000	0.001	0.009	1170	tags=86%, list=42%, signal=147%
ChemGenPerturb	STARK_PREFRONTAL_CORTEX_22Q11_DELETION_DN	48	−0.36	−2.78	0.000	0.001	0.009	1175	tags=77%, list=42%, signal=131%
GO	GO_SYNAPTIC_SIGNALING	64	−0.32	−2.78	0.000	0.001	0.006	1246	tags=77%, list=44%, signal=135%
ChemGenPerturb	MIKKELSEN_MEF_ICP_WITH_H3K4ME3_AND_H3K27ME3	10	−0.74	−2.78	0.000	0.001	0.010	737	tags=100%, list=26%, signal=135%
Oncogenic	PRC2_EED_UP.V1_UP	21	−0.53	−2.76	0.000	0.000	0.002	494	tags=67%, list=18%, signal=80%
Immunogenic	GSE34156_NOD2_LIGAND_VS_TLR1_TLR2_LIGAND_24H_TRE	T 13	−0.63	−2.75	0.000	0.025	0.037	878	tags=92%, list=31%, signal=134%
GO	GO_CATION_CHANNEL_COMPLEX	29	−0.45	−2.75	0.000	0.001	0.008	1226	tags=90%, list=44%, signal=158%
GO	GO_MICROTUBULE	31	−0.41	−2.69	0.000	0.002	0.014	512	tags=55%, list=18%, signal=66%
GO	GO_MRNA_METABOLIC_PROCESS	36	−0.40	−2.67	0.000	0.002	0.019	1157	tags=81%, list=41%, signal=135%
ChemGenPerturb	WEI_MYCN_TARGETS_WITH_E_BOX	65	−0.30	−2.65	0.000	0.001	0.021	1163	tags=71%, list=42%, signal=118%
GO	GO_BEHAVIOR	70	−0.29	−2.65	0.000	0.002	0.022	1248	tags=73%, list=45%, signal=128%
GO	GO_RNA_PROCESSING	48	−0.34	−2.62	0.000	0.003	0.032	1180	tags=75%, list=42%, signal=127%
ChemGenPerturb	GEORGES_TARGETS_OF_MIR192_AND_MIR215	87	−0.25	−2.59	0.000	0.002	0.037	1438	tags=77%, list=51%, signal=153%

*SIZE* Number of genes in the gene set after filtering out those genes not in the expression data set, *ES* Enrichment Score, *NES* Normalized Enrichment Score, *NOM p-val* Nominal P value, *FDR q-val* False Discovery Rate q value, *FWER p-val* Familywise-Error Rate q value, *RANK AT MAX* Rank at maximum enrichment score

## Discussion

Although the subjective experience of social isolation has significant health implications, understanding the biological basis of this association is impeded by limited availability of molecular genetic expression data from the human brain. Based on high-quality RNA expression data from the dorsolateral prefrontal cortex of 181 participant-donors from a longitudinal, prospective aging study, we now report on 337 upregulated and 43 downregulated gene sets whose expression at the time of death was significantly enriched (at FDR-corrected levels) as a function of experienced loneliness that was reported by participants almost 5 years prior to death. Based on prior associations between loneliness and a range of mental and physical diseases, and our prior results obtained in nucleus accumbens^24^, we were particularly interested whether loneliness reported at baseline would predict differential gene set expression associated with AD, neurological or behavioral disorders, inflammatory diseases, or cancer.

Indeed, AD was associated with both the most significant up- and down-regulated gene set across all MSigDB collections, respectively. This is an interesting result since we controlled for cognitive decline, as well as AD pathology and cerebral infarctions. Thus, self-reported loneliness at baseline predicted AD-related gene expression, over and above cognitive and pathological indicators, in the DLPFC at the time of death by almost 5 years. However, in the absence of gene expression data at the time of baseline, we do not know if any constituent gene’s expression value reflects underlying stable or dynamic processes.

Psychiatric disorders were implicated with the second-most down-regulated enriched gene set across all MSigDB collections, which was derived from a postmortem study of gene expression in the Brodman Area (BA) nine region of schizophrenic, bipolar, and depressed patients^48^. Our analysis controlled for depressive symptoms, suggesting that loneliness at baseline predicted differential gene expression at death over and above depressive symptoms. The gene set represents genes that correlate with a decrease in the density of calbindin-positive interneurons and that represent processes related to cellular metabolism, CNS development, cell motility and programmed cell death. Indeed, many other significant gene sets in our study were associated with these functions, such as the MSigDB Gene Ontology collections of genes related to cellular lipid metabolic processes, cell movement, cellular amino acid metabolic processes, and three Chemical and Genetic Perturbation collections of gene sets related to apoptosis.

The immune system was implicated across several gene set collections, particularly Gene Ontology’s sets on the immune response, immune system process, immune effector process, innate immune response, humoral immune response, and adaptive immune response. Relatedly, significant Gene Ontology and Hallmark sets were associated with the inflammatory response.

Cancer was prominently associated with differential gene set expression: among the Top-25 most-enriched gene sets across all MSigDB collections, nine sets came from the Cancer Module (CM) Collection. These represented ovary genes (CM 1), lung genes (CM 5), trachea genes (CM 6), liver metabolic and xenobiotic response genes (CM 23), placenta genes (CM 38), heart, liver, kidney and pancreas metabolic and xenobiotic response genes (CM 55 and 88), heart genes (CM 60), and immune (humoral) and inflammatory response genes (CM 84).

There were thirteen highly pleiotropic genes that that appeared across all of these four diseases among the top-scoring gene sets. Their pleiotropy reflect ubiquitous biological functions, such as transcription factors GTPBP1 and TRIM38, protein kinases ABL1 and CTSH, mRNA degradation GTPBP1, cytokines and growth factors IFNA16 and IL18, cell differentiation markers CD58 and CD86, and transmembrane signaling enzyme and glycoproteins PLCG2 and TAPBP.

Taken together, a picture emerges by which the varied diseases associated with loneliness can be viewed as symptomatic of differentially expressed gene sets and their associated individual genes, some of which are highly pleiotropic due to their ubiquitous biological functions. This picture is similar to what we observed in a previous study of gene expression in the nucleus accumbens, where we found gene sets that were also associated with a wide range of behavioral processes, mental disorders and physical diseases^24^. However, in nucleus accumbens, all of the reported genes were individually significant at FDR q-values < 0.05, whereas in the present much larger sample, none were individually significant at these levels. Given that there were technical differences between these studies in how the genes were identified (RNAseq for DLPFC, ArrayStar gene chip for nucleus accumbens) and in the analytical approaches that were conducted, future studies using harmonized methods need to be conducted to determine to what extent loneliness is associated with brain region-specific differences in gene expression.

The study has a number of strengths. First, the data come from a prospective community-based cohort study with high rates of follow-up and autopsy. Second, participants were tested in their homes, minimizing a healthy volunteer effect. Third, we used a measure of loneliness from the baseline assessment when participants were enrolled without dementia, but also could confirm with measurements taken during subsequent visits that self-reported scores remained nearly unchanged between assessments at baseline and at last visit prior to death, consistent with conceptualization of loneliness as a stable trait. Fourth, this is the first study to examine gene expression as a function of loneliness in the human DLPFC. We report patterns of gene expression that are consistent with the only other human gene expression study from nucleus accumbens from the same cohort. Fifth, the sample of 181 individuals is large for this kind of study, and in fact seven times larger than the only other comparable study^24^. Sixth, our analytic strategy controlled for a number of potential confounds, including biographical variables (age, sex, education), psychological and health variables (depressive symptoms, interval between assessment and autopsy, slope of cognitive decline, AD pathology, presence of infarcts) and RIN score^49^, which at 7.1 was of high quality. Finally, gene expression data were based on RNAseq (as opposed to array-based technologies), providing a more comprehensive and non-biased view of the transcriptome.

The study also has limitations. The tissue was processed without removal of blood leukocytes, or separation of neuronal from other cell types. It is therefore not clear which sources of cell types contributed, and to what degree, to the observed pattern of gene expression. There are also limitations regarding causal inferences. The direction of effects between gene expression and loneliness cannot be established. It cannot be determined whether differential expression of genes associated with a variety of mental and physical illnesses reported here is causally linked to the subjective experience of social isolation, be it as an antecedent or sequela variable. Such determination would require longitudinal studies with repeated measures of gene expression in vivo, which is not possible in human subjects. Relatedly, the underlying mechanisms of differential gene expression, whether due to variation in DNA sequences (polymorphisms) or epigenetic or other regulatory mechanisms, remain to be examined in future large-scale GWAS or experimental studies, and may well be gene or gene-set-specific. Finally, the sample size—although unprecedented for a postmortem study of loneliness-gene expression—was too small to conduct a validation study with a second sample at this time.

The results presented here shed further light on the underlying genomics of loneliness and its associated cognitive and health-related sequelae. They identify novel molecular targets for studies of diseases that are exacerbated by the experience of subjective social isolation, and begin to reveal the underlying molecular regulatory architecture by which pleiotropic genes participate in a wide-ranging set of disease-processes associated with loneliness.

## Electronic supplementary material

Supplemental Table 1

Supplemental Table 2

Supplemental Table 3

Supplemental Table 4

Supplemental Table 5

Supplemental Table 6

Supplemental Table 7
