# Supplementary material for: Loneliness 5 years ante-mortem is associated with disease-related differential gene expression in postmortem dorsolateral prefrontal cortex
Source: Transl Psychiatry. 2018 Jan 10;8:2. doi: 10.1038/s41398-017-0086-2 (PMC5802527; doi:10.1038/s41398-017-0086-2)
Supplement: Supplementary file 1 — Supplemental Table 1 [file 41398_2017_86_MOESM1_ESM.pdf]

Gene	genenm	geneid	Estimate	SE	t	P
ENSG00000183386.5_FHL3	FHL3	ENSG00000183386.5	0.575	0.131	4.403	0.000
ENSG00000177352.9_CCDC71	CCDC71	ENSG00000177352.9	0.401	0.095	4.219	0.000
ENSG00000255501.1_CARD18	CARD18	ENSG00000255501.1	0.191	0.045	4.213	0.000
ENSG00000121895.7_TMEM156	TMEM156	ENSG00000121895.7	0.166	0.041	4.082	0.000
ENSG00000100441.5_KHNYN	KHNYN	ENSG00000100441.5	0.700	0.172	4.078	0.000
ENSG00000162430.12_SEPN1	SEPN1	ENSG00000162430.12	2.100	0.538	3.907	0.000
ENSG00000101346.6_POFUT1	POFUT1	ENSG00000101346.6	0.250	0.067	3.750	0.000
ENSG00000138185.11_ENTPD1	ENTPD1	ENSG00000138185.11	0.305	0.082	3.709	0.000
ENSG00000198879.6_SFMBT2	SFMBT2	ENSG00000198879.6	0.225	0.061	3.705	0.000
ENSG00000143641.8_GALNT2	GALNT2	ENSG00000143641.8	1.051	0.284	3.696	0.000
ENSG00000053918.10_KCNQ1	KCNQ1	ENSG00000053918.10	0.131	0.036	3.619	0.000
ENSG00000149591.12_TAGLN	TAGLN	ENSG00000149591.12	8.232	2.294	3.588	0.000
ENSG00000154133.9_ROBO4	ROBO4	ENSG00000154133.9	0.859	0.240	3.582	0.000
ENSG00000152270.4_PDE3B	PDE3B	ENSG00000152270.4	0.147	0.041	3.577	0.000
ENSG00000179604.8_CDC42EP4	CDC42EP4	ENSG00000179604.8	5.822	1.632	3.567	0.000
ENSG00000234840.1_RP11-399D6.2	RP11-399D6.2	ENSG00000234840.1	0.015	0.004	3.554	0.000
ENSG00000143416.15_SELENBP1	SELENBP1	ENSG00000143416.15	4.066	1.147	3.546	0.001
ENSG00000254266.1_RP11-594N15.2	RP11-594N15.2	ENSG00000254266.1	0.011	0.003	3.541	0.001
ENSG00000255362.1_RP11-619A14.3	RP11-619A14.3	ENSG00000255362.1	0.084	0.024	3.539	0.001
ENSG00000125968.7_ID1	ID1	ENSG00000125968.7	10.221	2.902	3.522	0.001
ENSG00000114745.9_GORASP1	GORASP1	ENSG00000114745.9	1.030	0.293	3.514	0.001
ENSG00000172296.8_SPTLC3	SPTLC3	ENSG00000172296.8	0.069	0.020	3.493	0.001
ENSG00000166689.9_PLEKHA7	PLEKHA7	ENSG00000166689.9	0.453	0.130	3.489	0.001
ENSG00000240990.4_HOXA11-AS	HOXA11-AS	ENSG00000240990.4	0.012	0.003	3.469	0.001
ENSG00000106003.8_LFNG	LFNG	ENSG00000106003.8	1.776	0.512	3.468	0.001
ENSG00000257281.1_RP11-1K3.1	RP11-1K3.1	ENSG00000257281.1	0.011	0.003	3.463	0.001
ENSG00000198951.6_NAGA	NAGA	ENSG00000198951.6	0.357	0.104	3.418	0.001
ENSG00000200090.1_Y-RNA	Y-RNA	ENSG00000200090.1	0.132	0.038	3.418	0.001
ENSG00000131724.6_IL13RA1	IL13RA1	ENSG00000131724.6	0.759	0.222	3.417	0.001
ENSG00000213977.3_TAX1BP3	TAX1BP3	ENSG00000213977.3	3.414	1.001	3.411	0.001
ENSG00000182578.9_CSF1R	CSF1R	ENSG00000182578.9	1.562	0.459	3.403	0.001
ENSG00000136840.13_ST6GALNAC4	ST6GALNAC4	ENSG00000136840.13	0.596	0.175	3.402	0.001
ENSG00000249673.1_NOP14-AS1	NOP14-AS1	ENSG00000249673.1	0.356	0.105	3.393	0.001
ENSG00000124067.12_SLC12A4	SLC12A4	ENSG00000124067.12	1.094	0.323	3.387	0.001
ENSG00000087903.8_RFX2	RFX2	ENSG00000087903.8	0.594	0.176	3.375	0.001
ENSG00000169418.8_NPR1	NPR1	ENSG00000169418.8	0.140	0.042	3.370	0.001
ENSG00000162542.8_TMCO4	TMCO4	ENSG00000162542.8	0.246	0.073	3.363	0.001
ENSG00000185813.4_PCYT2	PCYT2	ENSG00000185813.4	1.535	0.457	3.362	0.001
ENSG00000182944.13_EWSR1	EWSR1	ENSG00000182944.13	3.236	0.963	3.360	0.001
ENSG00000241294.1_IGKV2-24	IGKV2-24	ENSG00000241294.1	0.003	0.001	3.355	0.001
ENSG00000100003.12_SEC14L2	SEC14L2	ENSG00000100003.12	2.740	0.819	3.345	0.001
ENSG00000261011.1_RP11-96C23.11	RP11-96C23.11	ENSG00000261011.1	0.220	0.066	3.342	0.001
ENSG00000137103.11_TMEM8B	TMEM8B	ENSG00000137103.11	0.675	0.203	3.332	0.001
ENSG00000232273.1_FTH1P1	FTH1P1	ENSG00000232273.1	0.000	0.000	3.330	0.001
ENSG00000133392.11_MYH11	MYH11	ENSG00000133392.11	0.561	0.169	3.316	0.001
ENSG00000226113.1_RP11-672F9.1	RP11-672F9.1	ENSG00000226113.1	0.034	0.010	3.314	0.001
ENSG00000267128.1_RNF157-AS1	RNF157-AS1	ENSG00000267128.1	0.298	0.090	3.310	0.001
ENSG00000105607.7_GCDH	GCDH	ENSG00000105607.7	1.368	0.415	3.299	0.001
ENSG00000236545.1_AP001619.3	AP001619.3	ENSG00000236545.1	0.021	0.006	3.295	0.001
ENSG00000186322.8_RP13-608F4.1	RP13-608F4.1	ENSG00000186322.8	0.314	0.095	3.292	0.001
ENSG00000142611.11_PRDM16	PRDM16	ENSG00000142611.11	0.408	0.124	3.280	0.001
ENSG00000228477.1_RP3-342P20.2	RP3-342P20.2	ENSG00000228477.1	0.291	0.089	3.279	0.001
ENSG00000163017.9_ACTG2	ACTG2	ENSG00000163017.9	0.111	0.034	3.273	0.001
ENSG00000244413.1_RP11-1057B8.1	RP11-1057B8.1	ENSG00000244413.1	0.006	0.002	3.271	0.001
ENSG00000149403.5_GRIK4	GRIK4	ENSG00000149403.5	0.190	0.058	3.264	0.001
ENSG00000181856.10_SLC2A4	SLC2A4	ENSG00000181856.10	0.236	0.072	3.263	0.001
ENSG00000107796.8_ACTA2	ACTA2	ENSG00000107796.8	2.232	0.685	3.260	0.001
ENSG00000139567.8_ACVRL1	ACVRL1	ENSG00000139567.8	1.391	0.428	3.254	0.001
ENSG00000184985.12_SORCS2	SORCS2	ENSG00000184985.12	0.857	0.263	3.252	0.001

ENSG00000254478.1_RP11-158I9.1	RP11-158I9.1	ENSG00000254478.1	0.043	0.013	3.249	0.001
ENSG00000072163.13_LIMS2	LIMS2	ENSG00000072163.13	2.689	0.829	3.244	0.001
ENSG00000198892.6_SHISA4	SHISA4	ENSG00000198892.6	5.699	1.759	3.240	0.001
ENSG00000179766.11_ATP8B5P	ATP8B5P	ENSG00000179766.11	0.011	0.004	3.236	0.001
ENSG00000233081.1_RP11-440G5.2	RP11-440G5.2	ENSG00000233081.1	0.035	0.011	3.227	0.002
ENSG00000203722.3_RAET1G	RAET1G	ENSG00000203722.3	0.052	0.016	3.223	0.002
ENSG00000174059.12_CD34	CD34	ENSG00000174059.12	2.504	0.778	3.217	0.002
ENSG00000144063.3_MALL	MALL	ENSG00000144063.3	0.164	0.051	3.214	0.002
ENSG00000179639.6_FCER1A	FCER1A	ENSG00000179639.6	0.062	0.019	3.211	0.002
ENSG00000101335.5_MYL9	MYL9	ENSG00000101335.5	3.230	1.007	3.207	0.002
ENSG00000117362.8_APH1A	APH1A	ENSG00000117362.8	2.096	0.654	3.205	0.002
ENSG00000173402.7_DAG1	DAG1	ENSG00000173402.7	1.271	0.397	3.198	0.002
ENSG00000139626.10_ITGB7	ITGB7	ENSG00000139626.10	0.107	0.033	3.196	0.002
ENSG00000178814.11_OPLAH	OPLAH	ENSG00000178814.11	0.636	0.199	3.189	0.002
ENSG00000248319.1_RP11-205M3.3	RP11-205M3.3	ENSG00000248319.1	0.004	0.001	3.186	0.002
ENSG00000071282.7_LMCD1	LMCD1	ENSG00000071282.7	0.475	0.149	3.182	0.002
ENSG00000215244.1_AL137145.2	AL137145.2	ENSG00000215244.1	0.083	0.026	3.172	0.002
ENSG00000175084.7_DES	DES	ENSG00000175084.7	0.182	0.057	3.172	0.002
ENSG00000225619.1_AC009232.2	AC009232.2	ENSG00000225619.1	0.006	0.002	3.170	0.002
ENSG00000267169.1_CTB-55O6.12	CTB-55O6.12	ENSG00000267169.1	0.198	0.062	3.168	0.002
ENSG00000251154.1_AC006153.3	AC006153.3	ENSG00000251154.1	0.004	0.001	3.163	0.002
ENSG00000197879.9_MYO1C	MYO1C	ENSG00000197879.9	0.644	0.204	3.163	0.002
ENSG00000198467.7_TPM2	TPM2	ENSG00000198467.7	5.050	1.601	3.155	0.002
ENSG00000101000.3_PROCR	PROCR	ENSG00000101000.3	0.274	0.087	3.154	0.002
ENSG00000138756.13_BMP2K	BMP2K	ENSG00000138756.13	0.334	0.106	3.152	0.002
ENSG00000197549.5_PRAMEL	PRAMEL	ENSG00000197549.5	0.001	0.000	3.152	0.002
ENSG00000267395.1_AC074212.6	AC074212.6	ENSG00000267395.1	0.117	0.037	3.147	0.002
ENSG00000106991.8_ENG	ENG	ENSG00000106991.8	3.728	1.185	3.146	0.002
ENSG00000160678.7_S100A1	S100A1	ENSG00000160678.7	37.482	11.923	3.144	0.002
ENSG00000243353.1_RP11-667M19.1	RP11-667M19.1	ENSG00000243353.1	1.922	0.611	3.143	0.002
ENSG00000173597.3_SULT1B1	SULT1B1	ENSG00000173597.3	0.045	0.014	3.137	0.002
ENSG00000165202.2_OR1Q1	OR1Q1	ENSG00000165202.2	0.019	0.006	3.133	0.002
ENSG00000243989.3_ACY1	ACY1	ENSG00000243989.3	0.750	0.239	3.132	0.002
ENSG00000130283.6_GDF1	GDF1	ENSG00000130283.6	4.502	1.438	3.131	0.002
ENSG00000119681.7_LTBP2	LTBP2	ENSG00000119681.7	0.048	0.015	3.127	0.002
ENSG00000087245.8_MMP2	MMP2	ENSG00000087245.8	0.194	0.062	3.118	0.002
ENSG00000261720.1_RP11-161M6.5	RP11-161M6.5	ENSG00000261720.1	0.013	0.004	3.113	0.002
ENSG00000251624.1_UNC93B7	UNC93B7	ENSG00000251624.1	0.016	0.005	3.111	0.002
ENSG00000088888.13_MAVS	MAVS	ENSG00000088888.13	0.629	0.202	3.111	0.002
ENSG00000206149.6_HERC2P9	HERC2P9	ENSG00000206149.6	4.840	1.557	3.109	0.002
ENSG00000167693.12_NXN	NXN	ENSG00000167693.12	0.908	0.292	3.108	0.002
ENSG00000153029.10_MR1	MR1	ENSG00000153029.10	0.160	0.052	3.106	0.002
ENSG00000233122.1_CTAGE7P	CTAGE7P	ENSG00000233122.1	0.004	0.001	3.102	0.002
ENSG00000228412.1_RP4-625H18.2	RP4-625H18.2	ENSG00000228412.1	0.075	0.024	3.102	0.002
ENSG00000206168.1_Z69890.1	Z69890.1	ENSG00000206168.1	0.113	0.036	3.100	0.002
ENSG00000108405.3_P2RX1	P2RX1	ENSG00000108405.3	0.044	0.014	3.100	0.002
ENSG00000161509.9_GRIN2C	GRIN2C	ENSG00000161509.9	2.419	0.781	3.098	0.002
ENSG00000123384.9_LRP1	LRP1	ENSG00000123384.9	3.289	1.063	3.095	0.002
ENSG00000172057.5_ORMDL3	ORMDL3	ENSG00000172057.5	1.552	0.502	3.090	0.002
ENSG00000110057.3_UNC93B1	UNC93B1	ENSG00000110057.3	0.599	0.194	3.090	0.002
ENSG00000188549.8_C15orf52	C15orf52	ENSG00000188549.8	0.447	0.145	3.083	0.002
ENSG00000177697.12_CD151	CD151	ENSG00000177697.12	3.523	1.146	3.073	0.002
ENSG00000135744.7_AGT	AGT	ENSG00000135744.7	18.545	6.043	3.069	0.003
ENSG00000262098.1_CTD-2561B21.10	CTD-2561B21.10	ENSG00000262098.1	0.808	0.263	3.069	0.003
ENSG00000240211.1_RP11-758P17.3	RP11-758P17.3	ENSG00000240211.1	0.002	0.001	3.067	0.003
ENSG00000167874.6_TMEM88	TMEM88	ENSG00000167874.6	0.633	0.207	3.067	0.003
ENSG00000254917.1_OR7E15P	OR7E15P	ENSG00000254917.1	0.015	0.005	3.065	0.003
ENSG00000263050.1_RP11-667K14.3	RP11-667K14.3	ENSG00000263050.1	0.115	0.038	3.063	0.003
ENSG00000237921.2_AC004543.2	AC004543.2	ENSG00000237921.2	0.170	0.055	3.058	0.003
ENSG00000265927.1_CTD-2532D12.5	CTD-2532D12.5	ENSG00000265927.1	0.003	0.001	3.057	0.003

ENSG00000251278.1_AC006499.2	AC006499.2	ENSG00000251278.1	0.000	0.000	3.056	0.003
ENSG00000266499.1_AC008536.1	AC008536.1	ENSG00000266499.1	0.001	0.000	3.056	0.003
ENSG00000234136.1_AC055764.1	AC055764.1	ENSG00000234136.1	0.000	0.000	3.056	0.003
ENSG00000258542.1_AC068831.11	AC068831.11	ENSG00000258542.1	0.000	0.000	3.056	0.003
ENSG00000223523.1_AC079613.1	AC079613.1	ENSG00000223523.1	0.000	0.000	3.056	0.003
ENSG00000229380.1_AC147651.5	AC147651.5	ENSG00000229380.1	0.001	0.000	3.056	0.003
ENSG00000223896.1_CCNJP2	CCNJP2	ENSG00000223896.1	0.000	0.000	3.056	0.003
ENSG00000250866.1_CTD-2297D10.1	CTD-2297D10.1	ENSG00000250866.1	0.000	0.000	3.056	0.003
ENSG00000232948.1_DEFB130	DEFB130	ENSG00000232948.1	0.000	0.000	3.056	0.003
ENSG00000244203.2_FOXP1-AS1	FOXP1-AS1	ENSG00000244203.2	0.000	0.000	3.056	0.003
ENSG00000206474.6_OR10C1	OR10C1	ENSG00000206474.6	0.000	0.000	3.056	0.003
ENSG00000180259.5_PRNT	PRNT	ENSG00000180259.5	0.000	0.000	3.056	0.003
ENSG00000233050.1_RP11-1236K1.1	RP11-1236K1.1	ENSG00000233050.1	0.000	0.000	3.056	0.003
ENSG00000253111.1_RP11-136O12.2	RP11-136O12.2	ENSG00000253111.1	0.001	0.000	3.056	0.003
ENSG00000254261.1_RP11-18A15.1	RP11-18A15.1	ENSG00000254261.1	0.000	0.000	3.056	0.003
ENSG00000253634.1_RP11-587H10.2	RP11-587H10.2	ENSG00000253634.1	0.000	0.000	3.056	0.003
ENSG00000248349.1_RP11-79C6.1	RP11-79C6.1	ENSG00000248349.1	0.000	0.000	3.056	0.003
ENSG00000258784.1_RP11-813I20.2	RP11-813I20.2	ENSG00000258784.1	0.000	0.000	3.056	0.003
ENSG00000267162.1_RP11-815J4.4	RP11-815J4.4	ENSG00000267162.1	0.000	0.000	3.056	0.003
ENSG00000256484.1_RP13-507P19.1	RP13-507P19.1	ENSG00000256484.1	0.000	0.000	3.056	0.003
ENSG00000187806.3_TMEM202	TMEM202	ENSG00000187806.3	0.000	0.000	3.056	0.003
ENSG00000235278.2_ZNF652P1	ZNF652P1	ENSG00000235278.2	0.000	0.000	3.056	0.003
ENSG00000237303.2_HIGD1AP8	HIGD1AP8	ENSG00000237303.2	7.222	2.364	3.055	0.003
ENSG00000126458.2_RRAS	RRAS	ENSG00000126458.2	2.119	0.694	3.052	0.003
ENSG00000181741.7_FDX1P1	FDX1P1	ENSG00000181741.7	0.035	0.011	3.050	0.003
ENSG00000136868.8_SLC31A1	SLC31A1	ENSG00000136868.8	0.336	0.110	3.041	0.003
ENSG00000199879.1_U1	RNU1-120P	ENSG00000199879.1	0.051	0.017	3.040	0.003
ENSG00000115318.7_LOXL3	LOXL3	ENSG00000115318.7	0.203	0.067	3.034	0.003
ENSG00000148400.9_NOTCH1	NOTCH1	ENSG00000148400.9	0.667	0.220	3.033	0.003
ENSG00000245522.2_RP11-540A21.2	RP11-540A21.2	ENSG00000245522.2	0.011	0.003	3.033	0.003
ENSG00000197943.5_PLCG2	PLCG2	ENSG00000197943.5	0.212	0.070	3.030	0.003
ENSG00000241013.1_HIST2H2BB	HIST2H2BB	ENSG00000241013.1	0.075	0.025	3.028	0.003
ENSG00000256069.3_A2MP1	A2MP1	ENSG00000256069.3	0.026	0.008	3.027	0.003
ENSG00000166816.9_LDHD	LDHD	ENSG00000166816.9	1.666	0.551	3.025	0.003
ENSG00000162576.11_MXRA8	MXRA8	ENSG00000162576.11	0.988	0.327	3.025	0.003
ENSG00000167644.6_C19orf33	C19orf33	ENSG00000167644.6	0.458	0.152	3.021	0.003
ENSG00000226324.1_RP11-115M14.1	RP11-115M14.1	ENSG00000226324.1	0.115	0.038	3.019	0.003
ENSG00000206190.7_ATP10A	ATP10A	ENSG00000206190.7	0.705	0.234	3.014	0.003
ENSG00000114026.16_OGG1	OGG1	ENSG00000114026.16	0.454	0.151	3.013	0.003
ENSG00000187244.5_BCAM	BCAM	ENSG00000187244.5	2.438	0.809	3.013	0.003
ENSG00000257513.2_RP11-677O4.1	RP11-677O4.1	ENSG00000257513.2	0.024	0.008	3.011	0.003
ENSG00000250237.1_CTC-498J12.1	CTC-498J12.1	ENSG00000250237.1	0.060	0.020	3.010	0.003
ENSG00000109066.9_TMEM104	TMEM104	ENSG00000109066.9	0.255	0.085	3.008	0.003
ENSG00000232749.1_RP11-15B24.4	RP11-15B24.4	ENSG00000232749.1	0.005	0.002	3.005	0.003
ENSG00000095321.11_CRAT	CRAT	ENSG00000095321.11	1.196	0.399	3.002	0.003
ENSG00000117298.10_ECE1	ECE1	ENSG00000117298.10	1.489	0.496	3.000	0.003
ENSG00000182557.3_SPNS3	SPNS3	ENSG00000182557.3	0.070	0.023	2.999	0.003
ENSG00000161911.7_TREML1	TREML1	ENSG00000161911.7	0.116	0.039	2.999	0.003
ENSG00000065320.4_NTN1	NTN1	ENSG00000065320.4	0.176	0.059	2.998	0.003
ENSG00000111341.5_MGP	MGP	ENSG00000111341.5	1.867	0.623	2.998	0.003
ENSG00000103507.9_BCKDK	BCKDK	ENSG00000103507.9	0.951	0.317	2.996	0.003
ENSG00000179715.8_PCED1B	PCED1B	ENSG00000179715.8	0.105	0.035	2.996	0.003
ENSG00000168899.4_VAMP5	VAMP5	ENSG00000168899.4	3.923	1.310	2.994	0.003
ENSG00000211701.2_TRGV1	TRGV1	ENSG00000211701.2	0.002	0.001	2.992	0.003
ENSG00000148803.7_FUOM	FUOM	ENSG00000148803.7	0.352	0.118	2.988	0.003
ENSG00000206144.5_RP11-400K9.2	RP11-400K9.2	ENSG00000206144.5	0.004	0.001	2.985	0.003
ENSG00000259470.1_KRT8P9	KRT8P9	ENSG00000259470.1	0.008	0.003	2.985	0.003
ENSG00000154930.10_ACSS1	ACSS1	ENSG00000154930.10	4.535	1.519	2.985	0.003
ENSG00000188643.5_S100A16	S100A16	ENSG00000188643.5	4.653	1.559	2.984	0.003
ENSG00000040531.10_CTNS	CTNS	ENSG00000040531.10	0.276	0.093	2.984	0.003

ENSG00000159348.8_CYB5R1	CYB5R1	ENSG00000159348.8	1.074	0.361	2.978	0.003
ENSG00000006534.11_ALDH3B1	ALDH3B1	ENSG00000006534.11	0.129	0.043	2.975	0.003
ENSG00000077942.13_FBLN1	FBLN1	ENSG00000077942.13	1.247	0.419	2.973	0.003
ENSG00000125245.8_GPR18	GPR18	ENSG00000125245.8	0.009	0.003	2.971	0.003
ENSG00000263884.1_RP11-705O1.8	RP11-705O1.8	ENSG00000263884.1	0.039	0.013	2.967	0.003
ENSG00000147475.9_ERLIN2	ERLIN2	ENSG00000147475.9	0.463	0.156	2.967	0.003
ENSG00000234906.3_APOC2	APOC2	ENSG00000234906.3	0.168	0.057	2.954	0.004
ENSG00000228970.4_UBTFL6	UBTFL6	ENSG00000228970.4	0.025	0.008	2.952	0.004
ENSG00000006756.11_ARSD	ARSD	ENSG00000006756.11	0.281	0.095	2.951	0.004
ENSG00000103187.5_COTL1	COTL1	ENSG00000103187.5	3.783	1.282	2.950	0.004
ENSG00000119535.13_CSF3R	CSF3R	ENSG00000119535.13	0.709	0.240	2.950	0.004
ENSG00000049239.7_H6PD	H6PD	ENSG00000049239.7	0.407	0.138	2.950	0.004
ENSG00000185339.4_TCN2	TCN2	ENSG00000185339.4	1.086	0.369	2.941	0.004
ENSG00000224930.2_LINC00162	LINC00162	ENSG00000224930.2	0.020	0.007	2.941	0.004
ENSG00000182118.5_FAM89A	FAM89A	ENSG00000182118.5	1.022	0.348	2.940	0.004
ENSG00000235371.1_RP4-764D2.1	RP4-764D2.1	ENSG00000235371.1	0.004	0.001	2.940	0.004
ENSG00000100297.8_MCM5	MCM5	ENSG00000100297.8	0.350	0.119	2.940	0.004
ENSG00000198848.8_CES1	CES1	ENSG00000198848.8	0.381	0.130	2.939	0.004
ENSG00000182054.5_IDH2	IDH2	ENSG00000182054.5	4.667	1.588	2.938	0.004
ENSG00000109501.9_WFS1	WFS1	ENSG00000109501.9	2.678	0.912	2.936	0.004
ENSG00000122642.6_FKBP9	FKBP9	ENSG00000122642.6	1.052	0.359	2.931	0.004
ENSG00000103257.4_SLC7A5	SLC7A5	ENSG00000103257.4	6.144	2.097	2.930	0.004
ENSG00000160789.15_LMNA	LMNA	ENSG00000160789.15	2.682	0.915	2.930	0.004
ENSG00000231817.1_RP11-189B4.6	RP11-189B4.6	ENSG00000231817.1	0.001	0.000	2.929	0.004
ENSG00000099260.5_PALMD	PALMD	ENSG00000099260.5	0.399	0.136	2.927	0.004
ENSG00000265535.1_MetazoaSRP	RN7SL475P	ENSG00000265535.1	0.030	0.010	2.926	0.004
ENSG00000264235.1_RP13-270P17.1	RP13-270P17.1	ENSG00000264235.1	0.047	0.016	2.925	0.004
ENSG00000048471.8_SNX29	SNX29	ENSG00000048471.8	0.191	0.065	2.925	0.004
ENSG00000100243.15_CYB5R3	CYB5R3	ENSG00000100243.15	6.805	2.330	2.921	0.004
ENSG00000178209.10_PLEC	PLEC	ENSG00000178209.10	3.277	1.122	2.920	0.004
ENSG00000137491.9_SLCO2B1	SLCO2B1	ENSG00000137491.9	2.181	0.748	2.917	0.004
ENSG00000172819.12_RARG	RARG	ENSG00000172819.12	0.343	0.118	2.912	0.004
ENSG00000228587.1_RP1-41P2.7	RP1-41P2.7	ENSG00000228587.1	0.001	0.000	2.909	0.004
ENSG00000107736.14_CDH23	CDH23	ENSG00000107736.14	0.428	0.147	2.907	0.004
ENSG00000110799.9_VWF	VWF	ENSG00000110799.9	3.057	1.052	2.907	0.004
ENSG00000167601.6_AXL	AXL	ENSG00000167601.6	1.044	0.360	2.904	0.004
ENSG00000136802.7_LRRC8A	LRRC8A	ENSG00000136802.7	4.603	1.586	2.902	0.004
ENSG00000243431.1_RPL5P30	RPL5P30	ENSG00000243431.1	0.116	0.040	2.902	0.004
ENSG00000183255.7_PTTG1IP	PTTG1IP	ENSG00000183255.7	5.834	2.010	2.902	0.004
ENSG00000164574.11_GALNT10	GALNT10	ENSG00000164574.11	0.331	0.114	2.895	0.004
ENSG00000202125.1_U1	RNU1-100P	ENSG00000202125.1	0.115	0.040	2.894	0.004
ENSG00000249306.1_RP11-267A15.1	RP11-267A15.1	ENSG00000249306.1	0.579	0.200	2.892	0.004
ENSG00000258740.1_RP11-293M10.1	RP11-293M10.1	ENSG00000258740.1	0.035	0.012	2.891	0.004
ENSG00000231064.1_RP11-263K19.4	RP11-263K19.4	ENSG00000231064.1	2.130	0.737	2.890	0.004
ENSG00000010704.14_HFE	HFE	ENSG00000010704.14	0.133	0.046	2.887	0.004
ENSG00000100345.15_MYH9	MYH9	ENSG00000100345.15	2.719	0.942	2.886	0.004
ENSG00000245017.2_RP11-181C3.1	RP11-181C3.1	ENSG00000245017.2	0.051	0.018	2.883	0.004
ENSG00000141497.9_ZMYND15	ZMYND15	ENSG00000141497.9	0.121	0.042	2.880	0.004
ENSG00000135222.6_CSN2	CSN2	ENSG00000135222.6	0.000	0.000	2.880	0.004
ENSG00000104812.8_GYS1	GYS1	ENSG00000104812.8	0.785	0.273	2.880	0.004
ENSG00000197119.7_SLC25A29	SLC25A29	ENSG00000197119.7	3.737	1.301	2.871	0.005
ENSG00000267515.1_RP11-861E21.3	RP11-861E21.3	ENSG00000267515.1	0.068	0.024	2.870	0.005
ENSG00000196497.11_IPO4	IPO4	ENSG00000196497.11	0.229	0.080	2.866	0.005
ENSG00000234135.1_RPL23AP83	RPL23AP83	ENSG00000234135.1	0.007	0.002	2.865	0.005
ENSG00000232480.1_RP11-224O19.2	RP11-224O19.2	ENSG00000232480.1	0.067	0.023	2.860	0.005
ENSG00000084774.8_CAD	CAD	ENSG00000084774.8	0.468	0.164	2.858	0.005
ENSG00000255521.1_RP4-607I7.1	RP4-607I7.1	ENSG00000255521.1	0.026	0.009	2.856	0.005
ENSG00000100599.11_RIN3	RIN3	ENSG00000100599.11	0.337	0.118	2.855	0.005
ENSG00000114353.12_GNAI2	GNAI2	ENSG00000114353.12	7.422	2.600	2.854	0.005
ENSG00000182718.11_ANXA2	ANXA2	ENSG00000182718.11	2.911	1.022	2.848	0.005

ENSG00000223802.2_CERS1	CERS1	ENSG00000223802.2	9.001	3.161	2.848	0.005
ENSG00000100344.6_PNPLA3	PNPLA3	ENSG00000100344.6	0.097	0.034	2.846	0.005
ENSG00000248762.2_RP11-31K23.2	RP11-31K23.2	ENSG00000248762.2	0.000	0.000	2.845	0.005
ENSG00000172638.8_EFEMP2	EFEMP2	ENSG00000172638.8	2.379	0.837	2.843	0.005
ENSG00000167291.9_TBC1D16	TBC1D16	ENSG00000167291.9	0.699	0.246	2.842	0.005
ENSG00000140497.12_SCAMP2	SCAMP2	ENSG00000140497.12	1.007	0.355	2.841	0.005
ENSG00000168938.5_PPIC	PPIC	ENSG00000168938.5	0.296	0.104	2.837	0.005
ENSG00000100311.12_PDGFB	PDGFB	ENSG00000100311.12	0.616	0.217	2.837	0.005
ENSG00000163520.9_FBLN2	FBLN2	ENSG00000163520.9	0.242	0.085	2.837	0.005
ENSG00000149923.9_PPP4C	PPP4C	ENSG00000149923.9	1.173	0.414	2.835	0.005
ENSG00000227009.1_FUNDC2P4	FUNDC2P4	ENSG00000227009.1	0.119	0.042	2.834	0.005
ENSG00000259863.1_SH3RF3-AS1	SH3RF3-AS1	ENSG00000259863.1	0.069	0.024	2.831	0.005
ENSG00000177943.8_MAMDC4	MAMDC4	ENSG00000177943.8	0.314	0.111	2.831	0.005
ENSG00000226889.1_RP11-474I16.8	RP11-474I16.8	ENSG00000226889.1	0.027	0.010	2.830	0.005
ENSG00000160396.7_HIPK4	HIPK4	ENSG00000160396.7	0.007	0.002	2.829	0.005
ENSG00000249381.1_LINC00500	LINC00500	ENSG00000249381.1	0.149	0.053	2.829	0.005
ENSG00000134463.10_ECHDC3	ECHDC3	ENSG00000134463.10	0.163	0.058	2.829	0.005
ENSG00000142798.11_HSPG2	HSPG2	ENSG00000142798.11	0.437	0.155	2.826	0.005
ENSG00000226824.2_RP4-756H11.3	RP4-756H11.3	ENSG00000226824.2	0.121	0.043	2.826	0.005
ENSG00000072310.12_SREBF1	SREBF1	ENSG00000072310.12	4.292	1.519	2.826	0.005
ENSG00000189334.4_S100A14	S100A14	ENSG00000189334.4	0.118	0.042	2.823	0.005
ENSG00000107719.7_PALD1	PALD1	ENSG00000107719.7	0.693	0.245	2.822	0.005
ENSG00000105971.9_CAV2	CAV2	ENSG00000105971.9	0.270	0.096	2.819	0.005
ENSG00000166341.6_DCHS1	DCHS1	ENSG00000166341.6	0.276	0.098	2.818	0.005
ENSG00000068078.13_FGFR3	FGFR3	ENSG00000068078.13	9.670	3.433	2.817	0.005
ENSG00000144476.5_CXCR7	CXCR7	ENSG00000144476.5	0.626	0.223	2.810	0.006
ENSG00000263331.1_CTC-508F8.1	CTC-508F8.1	ENSG00000263331.1	0.881	0.314	2.808	0.006
ENSG00000099889.9_ARVCF	ARVCF	ENSG00000099889.9	1.381	0.492	2.805	0.006
ENSG00000104903.4_LYL1	LYL1	ENSG00000104903.4	0.471	0.168	2.805	0.006
ENSG00000178922.12_HYI	HYI	ENSG00000178922.12	1.917	0.684	2.802	0.006
ENSG00000236216.4_PPP1R11P1	PPP1R11P1	ENSG00000236216.4	0.014	0.005	2.801	0.006
ENSG00000234189.1_AC099799.1	AC099799.1	ENSG00000234189.1	0.000	0.000	2.795	0.006
ENSG00000147885.2_IFNA16	IFNA16	ENSG00000147885.2	0.000	0.000	2.795	0.006
ENSG00000251311.1_RP11-249M12.2	RP11-249M12.2	ENSG00000251311.1	0.000	0.000	2.795	0.006
ENSG00000260624.1_RP11-24D15.1	RP11-24D15.1	ENSG00000260624.1	0.007	0.002	2.795	0.006
ENSG00000231104.1_RP11-354M20.3	RP11-354M20.3	ENSG00000231104.1	0.004	0.002	2.795	0.006
ENSG00000265698.1_RP11-41O4.3	RP11-41O4.3	ENSG00000265698.1	0.000	0.000	2.795	0.006
ENSG00000255870.1_RP11-667M19.5	RP11-667M19.5	ENSG00000255870.1	0.000	0.000	2.795	0.006
ENSG00000265217.1_RP11-775G23.1	RP11-775G23.1	ENSG00000265217.1	0.000	0.000	2.795	0.006
ENSG00000237131.1_RP1-69E11.3	RP1-69E11.3	ENSG00000237131.1	0.003	0.001	2.795	0.006
ENSG00000188765.6_TMSB4XP2	TMSB4XP2	ENSG00000188765.6	0.000	0.000	2.795	0.006
ENSG00000200545.1_U3	U3	ENSG00000200545.1	0.000	0.000	2.795	0.006
ENSG00000251748.1_U4atac	U4atac	ENSG00000251748.1	0.000	0.000	2.795	0.006
ENSG00000176692.4_FOXC2	FOXC2	ENSG00000176692.4	0.051	0.018	2.795	0.006
ENSG00000131730.11_CKMT2	CKMT2	ENSG00000131730.11	0.571	0.204	2.794	0.006
ENSG00000166484.14_MAPK7	MAPK7	ENSG00000166484.14	0.292	0.104	2.794	0.006
ENSG00000251999.1_SNORA31	SNORA31	ENSG00000251999.1	0.087	0.031	2.794	0.006
ENSG00000241810.1_HMGN2P13	HMGN2P13	ENSG00000241810.1	0.875	0.313	2.794	0.006
ENSG00000131634.9_TMEM204	TMEM204	ENSG00000131634.9	1.286	0.460	2.794	0.006
ENSG00000105483.10_CARD8	CARD8	ENSG00000105483.10	0.540	0.193	2.793	0.006
ENSG00000261794.1_GOLGA8H	GOLGA8H	ENSG00000261794.1	0.030	0.011	2.791	0.006
ENSG00000239204.1_AC002992.4	AC002992.4	ENSG00000239204.1	1.833	0.657	2.790	0.006
ENSG00000222974.1_7SK	RN7SKP228	ENSG00000222974.1	0.053	0.019	2.790	0.006
ENSG00000165795.14_NDRG2	NDRG2	ENSG00000165795.14	79.993	28.675	2.790	0.006
ENSG00000230495.1_RP11-462D18.2	RP11-462D18.2	ENSG00000230495.1	0.001	0.000	2.787	0.006
ENSG00000113721.8_PDGFRB	PDGFRB	ENSG00000113721.8	1.618	0.581	2.785	0.006
ENSG00000141506.9_PIK3R5	PIK3R5	ENSG00000141506.9	0.126	0.045	2.783	0.006
ENSG00000148288.7_GBGT1	GBGT1	ENSG00000148288.7	0.265	0.095	2.783	0.006
ENSG00000267573.1_CTC-782O7.2	CTC-782O7.2	ENSG00000267573.1	0.000	0.000	2.783	0.006
ENSG00000153885.9_KCTD15	KCTD15	ENSG00000153885.9	1.101	0.396	2.781	0.006

ENSG00000072274.8_TFRC	TFRC	ENSG00000072274.8	1.784	0.642	2.781	0.006
ENSG00000175899.10_A2M	A2M	ENSG00000175899.10	4.975	1.790	2.779	0.006
ENSG00000116016.9_EPAS1	EPAS1	ENSG00000116016.9	5.371	1.933	2.779	0.006
ENSG00000173706.8_HEG1	HEG1	ENSG00000173706.8	0.520	0.187	2.778	0.006
ENSG00000254887.1_CTC-378H22.1	CTC-378H22.1	ENSG00000254887.1	0.025	0.009	2.778	0.006
ENSG00000226579.1_RP11-351K23.3	RP11-351K23.3	ENSG00000226579.1	0.000	0.000	2.777	0.006
ENSG00000214782.3_MS4A18	MS4A18	ENSG00000214782.3	0.035	0.012	2.776	0.006
ENSG00000225972.1_MTND1P23	MTND1P23	ENSG00000225972.1	1.173	0.423	2.776	0.006
ENSG00000224566.1_FAM96AP2	FAM96AP2	ENSG00000224566.1	0.008	0.003	2.775	0.006
ENSG00000214525.4_AC130709.1	AC130709.1	ENSG00000214525.4	0.041	0.015	2.774	0.006
ENSG00000137486.12_ARRB1	ARRB1	ENSG00000137486.12	1.745	0.630	2.772	0.006
ENSG00000254286.1_RP11-89K10.1	RP11-89K10.1	ENSG00000254286.1	0.075	0.027	2.771	0.006
ENSG00000124942.8_AHNAK	AHNAK	ENSG00000124942.8	2.211	0.798	2.769	0.006
ENSG00000228132.1_RP11-279E1.1	RP11-279E1.1	ENSG00000228132.1	0.010	0.004	2.766	0.006
ENSG00000149485.11_FADS1	FADS1	ENSG00000149485.11	2.168	0.784	2.765	0.006
ENSG00000017483.10_SLC38A5	SLC38A5	ENSG00000017483.10	2.390	0.865	2.765	0.006
ENSG00000213820.3_RPL13P2	RPL13P2	ENSG00000213820.3	0.004	0.002	2.763	0.006
ENSG00000247595.2_SPTY2D1-AS1	SPTY2D1-AS1	ENSG00000247595.2	0.117	0.042	2.762	0.006
ENSG00000123104.7_ITPR2	ITPR2	ENSG00000123104.7	0.260	0.094	2.758	0.006
ENSG00000147082.13_CCNB3	CCNB3	ENSG00000147082.13	0.063	0.023	2.756	0.006
ENSG00000100234.10_TIMP3	TIMP3	ENSG00000100234.10	2.672	0.970	2.756	0.006
ENSG00000249094.2_RP1-7G5.6	RP1-7G5.6	ENSG00000249094.2	0.021	0.008	2.752	0.007
ENSG00000226705.1_RP11-298K24.2	RP11-298K24.2	ENSG00000226705.1	0.009	0.003	2.747	0.007
ENSG00000060971.12_ACAA1	ACAA1	ENSG00000060971.12	2.253	0.821	2.743	0.007
ENSG00000166682.6_TMPRSS5	TMPRSS5	ENSG00000166682.6	1.627	0.593	2.742	0.007
ENSG00000164849.7_GPR146	GPR146	ENSG00000164849.7	1.375	0.502	2.742	0.007
ENSG00000203711.6_C6orf99	C6orf99	ENSG00000203711.6	0.043	0.016	2.741	0.007
ENSG00000198838.7_RYR3	RYR3	ENSG00000198838.7	0.769	0.281	2.741	0.007
ENSG00000087077.7_TRIP6	TRIP6	ENSG00000087077.7	2.017	0.736	2.740	0.007
ENSG00000078061.7_ARAF	ARAF	ENSG00000078061.7	0.741	0.271	2.739	0.007
ENSG00000232287.2_SLC6A1-AS1	SLC6A1-AS1	ENSG00000232287.2	0.020	0.007	2.737	0.007
ENSG00000223741.1_PSMD4P1	PSMD4P1	ENSG00000223741.1	0.003	0.001	2.737	0.007
ENSG00000127241.12_MASP1	MASP1	ENSG00000127241.12	0.283	0.103	2.736	0.007
ENSG00000219863.3_RP3-483K16.3	RP3-483K16.3	ENSG00000219863.3	0.002	0.001	2.735	0.007
ENSG00000107738.13_C10orf54	C10orf54	ENSG00000107738.13	3.256	1.192	2.733	0.007
ENSG00000162512.11_SDC3	SDC3	ENSG00000162512.11	3.409	1.247	2.733	0.007
ENSG00000131669.5_NINJ1	NINJ1	ENSG00000131669.5	2.068	0.757	2.731	0.007
ENSG00000253988.1_RP11-489O18.1	RP11-489O18.1	ENSG00000253988.1	0.578	0.212	2.728	0.007
ENSG00000110651.6_CD81	CD81	ENSG00000110651.6	19.106	7.004	2.728	0.007
ENSG00000135540.7_NHSL1	NHSL1	ENSG00000135540.7	0.311	0.114	2.726	0.007
ENSG00000160111.6_CPAMD8	CPAMD8	ENSG00000160111.6	0.054	0.020	2.725	0.007
ENSG00000236308.1_RP11-316M21.7	RP11-316M21.7	ENSG00000236308.1	0.035	0.013	2.725	0.007
ENSG00000187720.10_THSD4	THSD4	ENSG00000187720.10	0.194	0.071	2.724	0.007
ENSG00000152661.7_GJA1	GJA1	ENSG00000152661.7	20.755	7.623	2.723	0.007
ENSG00000163599.10_CTLA4	CTLA4	ENSG00000163599.10	0.036	0.013	2.720	0.007
ENSG00000256671.4_LIMS3L	LIMS3L	ENSG00000256671.4	0.034	0.012	2.717	0.007
ENSG00000141448.4_GATA6	GATA6	ENSG00000141448.4	0.007	0.002	2.717	0.007
ENSG00000110756.13_HPS5	HPS5	ENSG00000110756.13	0.179	0.066	2.713	0.007
ENSG00000164520.7_RAET1E	RAET1E	ENSG00000164520.7	0.043	0.016	2.711	0.007
ENSG00000227619.1_RP11-492E3.2	RP11-492E3.2	ENSG00000227619.1	0.070	0.026	2.708	0.007
ENSG00000177119.11_ANO6	ANO6	ENSG00000177119.11	0.276	0.102	2.707	0.007
ENSG00000114738.6_MAPKAPK3	MAPKAPK3	ENSG00000114738.6	0.665	0.246	2.706	0.007
ENSG00000253671.1_RP11-806O11.1	RP11-806O11.1	ENSG00000253671.1	0.130	0.048	2.703	0.008
ENSG00000232889.2_AC087499.4	AC087499.4	ENSG00000232889.2	0.001	0.000	2.701	0.008
ENSG00000168918.9_INPP5D	INPP5D	ENSG00000168918.9	0.828	0.307	2.699	0.008
ENSG00000129354.7_AP1M2	AP1M2	ENSG00000129354.7	0.177	0.066	2.698	0.008
ENSG00000116729.9_WLS	WLS	ENSG00000116729.9	3.804	1.410	2.697	0.008
ENSG00000211890.3_IGHA2	IGHA2	ENSG00000211890.3	0.058	0.021	2.695	0.008
ENSG00000124782.14_RREB1	RREB1	ENSG00000124782.14	0.092	0.034	2.695	0.008
ENSG00000182853.7_VMO1	VMO1	ENSG00000182853.7	0.371	0.138	2.695	0.008

ENSG00000229605.4_RP11-492M23.2	RP11-492M23.2	ENSG00000229605.4	0.004	0.002	2.695	0.008
ENSG00000185585.15_OLFML2A	OLFML2A	ENSG00000185585.15	0.125	0.046	2.694	0.008
ENSG00000149090.7_PAMR1	PAMR1	ENSG00000149090.7	1.307	0.485	2.692	0.008
ENSG00000150977.9_RILPL2	RILPL2	ENSG00000150977.9	0.446	0.166	2.692	0.008
ENSG00000135097.2_MSI1	MSI1	ENSG00000135097.2	0.520	0.193	2.691	0.008
ENSG00000144857.10_BOC	BOC	ENSG00000144857.10	0.437	0.163	2.687	0.008
ENSG00000225527.1_RP11-383B4.4	RP11-383B4.4	ENSG00000225527.1	0.037	0.014	2.685	0.008
ENSG00000100206.5_DMC1	DMC1	ENSG00000100206.5	0.231	0.086	2.685	0.008
ENSG00000144579.3_CTDSP1	CTDSP1	ENSG00000144579.3	2.169	0.808	2.684	0.008
ENSG00000167930.11_ITFG3	ITFG3	ENSG00000167930.11	0.792	0.295	2.683	0.008
ENSG00000177303.5_CASKIN2	CASKIN2	ENSG00000177303.5	0.847	0.316	2.683	0.008
ENSG00000257496.1_RP11-474P2.4	RP11-474P2.4	ENSG00000257496.1	0.008	0.003	2.682	0.008
ENSG00000119943.6_PYROXD2	PYROXD2	ENSG00000119943.6	0.393	0.147	2.681	0.008
ENSG00000134762.12_DSC3	DSC3	ENSG00000134762.12	0.008	0.003	2.681	0.008
ENSG00000042493.10_CAPG	CAPG	ENSG00000042493.10	3.112	1.162	2.678	0.008
ENSG00000198435.2_NRARP	NRARP	ENSG00000198435.2	0.328	0.123	2.674	0.008
ENSG00000239748.2_MetazoaSRP	RN7SL795P	ENSG00000239748.2	0.152	0.057	2.672	0.008
ENSG00000188338.9_SLC38A3	SLC38A3	ENSG00000188338.9	2.055	0.769	2.671	0.008
ENSG00000100106.15_TRIOBP	TRIOBP	ENSG00000100106.15	0.804	0.301	2.670	0.008
ENSG00000261269.1_RP11-389C8.2	RP11-389C8.2	ENSG00000261269.1	0.357	0.134	2.670	0.008
ENSG00000020181.12_GPR124	GPR124	ENSG00000020181.12	0.352	0.132	2.670	0.008
ENSG00000155366.11_RHOC	RHOC	ENSG00000155366.11	7.328	2.748	2.667	0.008
ENSG00000254090.1_MTND2P32	MTND2P32	ENSG00000254090.1	0.039	0.014	2.666	0.008
ENSG00000171777.11_RASGRP4	RASGRP4	ENSG00000171777.11	0.083	0.031	2.660	0.009
ENSG00000089820.11_ARHGAP4	ARHGAP4	ENSG00000089820.11	0.846	0.319	2.657	0.009
ENSG00000226421.1_SLC25A5P5	SLC25A5P5	ENSG00000226421.1	0.001	0.000	2.657	0.009
ENSG00000187140.4_FOXD3	FOXD3	ENSG00000187140.4	0.006	0.002	2.656	0.009
ENSG00000237223.2_SULT1C2P1	SULT1C2P1	ENSG00000237223.2	0.020	0.008	2.656	0.009
ENSG00000136279.13_DBNL	DBNL	ENSG00000136279.13	1.715	0.647	2.651	0.009
ENSG00000049323.10_LTBP1	LTBP1	ENSG00000049323.10	0.249	0.094	2.650	0.009
ENSG00000196796.4_CTB-134H23.2	CTB-134H23.2	ENSG00000196796.4	0.038	0.014	2.649	0.009
ENSG00000162390.12_ACOT11	ACOT11	ENSG00000162390.12	0.585	0.221	2.648	0.009
ENSG00000241544.1_RP11-6F2.5	RP11-6F2.5	ENSG00000241544.1	0.017	0.006	2.646	0.009
ENSG00000164050.8_PLXNB1	PLXNB1	ENSG00000164050.8	6.412	2.425	2.644	0.009
ENSG00000228013.1_RP11-350G8.5	RP11-350G8.5	ENSG00000228013.1	0.123	0.047	2.643	0.009
ENSG00000188126.7_MYO15B	MYO15B	ENSG00000188126.7	0.364	0.138	2.643	0.009
ENSG00000105122.6_RASAL3	RASAL3	ENSG00000105122.6	0.131	0.050	2.643	0.009
ENSG00000238042.1_RP11-815M8.1	RP11-815M8.1	ENSG00000238042.1	0.027	0.010	2.639	0.009
ENSG00000167487.6_KLHL26	KLHL26	ENSG00000167487.6	0.533	0.202	2.639	0.009
ENSG00000259403.1_RP11-315L6.1	RP11-315L6.1	ENSG00000259403.1	0.041	0.016	2.639	0.009
ENSG00000241177.1_RP11-615J4.1	RP11-615J4.1	ENSG00000241177.1	0.007	0.003	2.636	0.009
ENSG00000223324.1_7SK	RN7SKP273	ENSG00000223324.1	0.071	0.027	2.636	0.009
ENSG00000197324.4_LRP10	LRP10	ENSG00000197324.4	1.220	0.463	2.636	0.009
ENSG00000266450.1_CTD-2015H3.2	CTD-2015H3.2	ENSG00000266450.1	0.005	0.002	2.636	0.009
ENSG00000197818.7_SLC9A8	SLC9A8	ENSG00000197818.7	0.243	0.092	2.635	0.009
ENSG00000228307.1_RP11-113A10.3	RP11-113A10.3	ENSG00000228307.1	0.028	0.010	2.635	0.009
ENSG00000211941.2_IGHV3-11	IGHV3-11	ENSG00000211941.2	0.019	0.007	2.632	0.009
ENSG00000087111.16_PIGS	PIGS	ENSG00000087111.16	0.579	0.220	2.628	0.009
ENSG00000153162.8_BMP6	BMP6	ENSG00000153162.8	0.268	0.102	2.627	0.009
ENSG00000265261.1_RP11-162A12.3	RP11-162A12.3	ENSG00000265261.1	0.000	0.000	2.627	0.009
ENSG00000179348.7_GATA2	GATA2	ENSG00000179348.7	0.374	0.142	2.627	0.009
ENSG00000114790.8_ARHGEF26	ARHGEF26	ENSG00000114790.8	1.191	0.454	2.626	0.009
ENSG00000235818.3_VN1R17P	VN1R17P	ENSG00000235818.3	0.015	0.006	2.624	0.009
ENSG00000243836.1_WDR86-AS1	WDR86-AS1	ENSG00000243836.1	0.032	0.012	2.622	0.010
ENSG00000161217.6_PCYT1A	PCYT1A	ENSG00000161217.6	0.412	0.157	2.619	0.010
ENSG00000260785.1_RP11-879D6.1	RP11-879D6.1	ENSG00000260785.1	0.004	0.002	2.619	0.010
ENSG00000154330.7_PGM5	PGM5	ENSG00000154330.7	0.125	0.048	2.618	0.010
ENSG00000177469.12_PTRF	PTRF	ENSG00000177469.12	1.638	0.626	2.617	0.010
ENSG00000172660.7_TAF15	TAF15	ENSG00000172660.7	3.793	1.450	2.616	0.010
ENSG00000224849.2_RP1-104O17.1	RP1-104O17.1	ENSG00000224849.2	0.045	0.017	2.612	0.010

ENSG00000260052.1_CTC-527H23.3	CTC-527H23.3	ENSG00000260052.1	0.033	0.013	2.611	0.010
ENSG00000196576.10_PLXNB2	PLXNB2	ENSG00000196576.10	2.003	0.768	2.608	0.010
ENSG00000126561.11_STAT5A	STAT5A	ENSG00000126561.11	0.253	0.097	2.607	0.010
ENSG00000213871.3_TAF9BP1	TAF9BP1	ENSG00000213871.3	0.002	0.001	2.607	0.010
ENSG00000241346.1_RP11-379B18.3	RP11-379B18.3	ENSG00000241346.1	0.268	0.103	2.606	0.010
ENSG00000264940.1_SNORD3C	SNORD3C	ENSG00000264940.1	0.233	0.089	2.606	0.010
ENSG00000211679.2_IGLC3	IGLC3	ENSG00000211679.2	0.312	0.120	2.606	0.010
ENSG00000227408.1_AMYP1	AMYP1	ENSG00000227408.1	0.043	0.016	2.601	0.010
ENSG00000135912.6_TTLL4	TTLL4	ENSG00000135912.6	0.704	0.271	2.601	0.010
ENSG00000260874.1_RP11-715J22.4	RP11-715J22.4	ENSG00000260874.1	0.054	0.021	2.600	0.010
ENSG00000100147.8_CCDC134	CCDC134	ENSG00000100147.8	0.070	0.027	2.600	0.010
ENSG00000115165.5_CYTIP	CYTIP	ENSG00000115165.5	0.065	0.025	2.600	0.010
ENSG00000117560.6_FASLG	FASLG	ENSG00000117560.6	0.013	0.005	2.599	0.010
ENSG00000005243.5_COPZ2	COPZ2	ENSG00000005243.5	0.641	0.247	2.598	0.010
ENSG00000213654.5_GPSM3	GPSM3	ENSG00000213654.5	0.660	0.255	2.593	0.010
ENSG00000250073.2_RP11-677M14.3	RP11-677M14.3	ENSG00000250073.2	0.040	0.015	2.593	0.010
ENSG00000165478.6_HEPACAM	HEPACAM	ENSG00000165478.6	8.674	3.346	2.592	0.010
ENSG00000264015.1_RP11-176N18.2	RP11-176N18.2	ENSG00000264015.1	0.021	0.008	2.592	0.010
ENSG00000260565.1_ERVK13-1	ERVK13-1	ENSG00000260565.1	0.618	0.238	2.591	0.010
ENSG00000101144.8_BMP7	BMP7	ENSG00000101144.8	1.373	0.530	2.591	0.010
ENSG00000167748.5_KLK1	KLK1	ENSG00000167748.5	0.011	0.004	2.591	0.010
ENSG00000132185.12_FCRLA	FCRLA	ENSG00000132185.12	0.038	0.015	2.590	0.010
ENSG00000239542.2_MetazoaSRP	RN7SL399P	ENSG00000239542.2	0.133	0.052	2.588	0.010
ENSG00000126903.11_SLC10A3	SLC10A3	ENSG00000126903.11	0.236	0.091	2.587	0.011
ENSG00000224963.2_U82695.9	U82695.9	ENSG00000224963.2	0.129	0.050	2.584	0.011
ENSG00000238207.1_AC009312.1	AC009312.1	ENSG00000238207.1	0.035	0.014	2.583	0.011
ENSG00000170075.7_GPR37L1	GPR37L1	ENSG00000170075.7	7.911	3.063	2.583	0.011
ENSG00000211707.3_TRBV7-1	TRBV7-1	ENSG00000211707.3	0.066	0.025	2.580	0.011
ENSG00000238086.3_PPP1R26P1	PPP1R26P1	ENSG00000238086.3	0.005	0.002	2.578	0.011
ENSG00000185504.12_C17orf70	C17orf70	ENSG00000185504.12	0.534	0.207	2.577	0.011
ENSG00000241238.1_AP001052.9	AP001052.9	ENSG00000241238.1	0.053	0.021	2.577	0.011
ENSG00000214063.6_TSPAN4	TSPAN4	ENSG00000214063.6	0.875	0.340	2.576	0.011
ENSG00000236992.1_RPL12L3	RPL12L3	ENSG00000236992.1	0.057	0.022	2.576	0.011
ENSG00000168077.8_SCARA3	SCARA3	ENSG00000168077.8	4.170	1.621	2.573	0.011
ENSG00000250948.1_RP11-1079K10.2	RP11-1079K10.2	ENSG00000250948.1	0.131	0.051	2.572	0.011
ENSG00000258888.1_RP11-326A13.1	RP11-326A13.1	ENSG00000258888.1	0.023	0.009	2.572	0.011
ENSG00000150281.6_CTF1	CTF1	ENSG00000150281.6	0.361	0.140	2.570	0.011
ENSG00000264050.1_RP11-22N12.2	RP11-22N12.2	ENSG00000264050.1	0.026	0.010	2.569	0.011
ENSG00000222664.1_7SK	RN7SKP123	ENSG00000222664.1	0.000	0.000	2.569	0.011
ENSG00000225798.1_AC025918.2	AC025918.2	ENSG00000225798.1	0.000	0.000	2.569	0.011
ENSG00000262313.1_CTD-2561B21.4	CTD-2561B21.4	ENSG00000262313.1	0.000	0.000	2.569	0.011
ENSG00000164822.4_DEFA6	DEFA6	ENSG00000164822.4	0.000	0.000	2.569	0.011
ENSG00000226255.1_RP11-288D15.2	RP11-288D15.2	ENSG00000226255.1	0.000	0.000	2.569	0.011
ENSG00000226664.1_RP4-745E8.2	RP4-745E8.2	ENSG00000226664.1	0.001	0.000	2.569	0.011
ENSG00000202428.1_U6	RNU6-108P	ENSG00000202428.1	0.002	0.001	2.569	0.011
ENSG00000223962.1_UBBP3	UBBP3	ENSG00000223962.1	0.000	0.000	2.569	0.011
ENSG00000167157.8_PRRX2	PRRX2	ENSG00000167157.8	0.055	0.022	2.568	0.011
ENSG00000166323.8_C11orf65	C11orf65	ENSG00000166323.8	0.127	0.049	2.567	0.011
ENSG00000237697.2_LINC00312	LINC00312	ENSG00000237697.2	0.057	0.022	2.566	0.011
ENSG00000122783.11_C7orf49	C7orf49	ENSG00000122783.11	0.321	0.125	2.565	0.011
ENSG00000130307.6_USHBP1	USHBP1	ENSG00000130307.6	0.312	0.122	2.565	0.011
ENSG00000160200.13_CBS	CBS	ENSG00000160200.13	4.118	1.608	2.561	0.011
ENSG00000104870.7_FCGRT	FCGRT	ENSG00000104870.7	1.814	0.709	2.560	0.011
ENSG00000007062.7_PROM1	PROM1	ENSG00000007062.7	0.231	0.090	2.556	0.011
ENSG00000182957.9_SPATA13	SPATA13	ENSG00000182957.9	0.433	0.170	2.554	0.012
ENSG00000267380.1_RP11-697E22.3	RP11-697E22.3	ENSG00000267380.1	0.006	0.002	2.554	0.012
ENSG00000185033.10_SEMA4B	SEMA4B	ENSG00000185033.10	1.365	0.535	2.554	0.012
ENSG00000258892.1_RP11-193F5.1	RP11-193F5.1	ENSG00000258892.1	1.311	0.513	2.554	0.012
ENSG00000178719.12_GRINA	GRINA	ENSG00000178719.12	7.409	2.902	2.553	0.012
ENSG00000260833.2_AC124789.1	AC124789.1	ENSG00000260833.2	0.047	0.019	2.550	0.012

ENSG00000100033.11_PRODH	PRODH	ENSG00000100033.11	12.886	5.055	2.549	0.012
ENSG00000227289.1_HSFY3P	HSFY3P	ENSG00000227289.1	0.006	0.003	2.549	0.012
ENSG00000258021.1_RP11-1100L3.4	RP11-1100L3.4	ENSG00000258021.1	0.008	0.003	2.547	0.012
ENSG00000255857.1_PXN-AS1	PXN-AS1	ENSG00000255857.1	0.310	0.122	2.547	0.012
ENSG00000176895.8_OR51A7	OR51A7	ENSG00000176895.8	0.002	0.001	2.546	0.012
ENSG00000082781.6_ITGB5	ITGB5	ENSG00000082781.6	1.200	0.471	2.546	0.012
ENSG00000250574.1_CTD-2275D10.2	CTD-2275D10.2	ENSG00000250574.1	0.000	0.000	2.546	0.012
ENSG00000155465.14_SLC7A7	SLC7A7	ENSG00000155465.14	0.169	0.067	2.545	0.012
ENSG00000267317.1_CTB-25B13.12	CTB-25B13.12	ENSG00000267317.1	0.159	0.063	2.544	0.012
ENSG00000231079.1_AC105402.4	AC105402.4	ENSG00000231079.1	0.006	0.002	2.543	0.012
ENSG00000263391.1_AL512428.1	AL512428.1	ENSG00000263391.1	1.478	0.581	2.542	0.012
ENSG00000154310.12_TNIK	TNIK	ENSG00000154310.12	1.125	0.443	2.539	0.012
ENSG00000157227.8_MMP14	MMP14	ENSG00000157227.8	0.319	0.126	2.538	0.012
ENSG00000242137.1_RPL11P5	RPL11P5	ENSG00000242137.1	0.009	0.003	2.537	0.012
ENSG00000235169.3_SMIM1	SMIM1	ENSG00000235169.3	0.190	0.075	2.537	0.012
ENSG00000105612.4_DNASE2	DNASE2	ENSG00000105612.4	0.674	0.266	2.536	0.012
ENSG00000187268.7_FAM9C	FAM9C	ENSG00000187268.7	0.019	0.007	2.535	0.012
ENSG00000167261.9_DPEP2	DPEP2	ENSG00000167261.9	0.127	0.050	2.533	0.012
ENSG00000179674.2_ARL14	ARL14	ENSG00000179674.2	0.001	0.001	2.530	0.012
ENSG00000178175.7_ZNF366	ZNF366	ENSG00000178175.7	0.156	0.062	2.529	0.012
ENSG00000102265.7_TIMP1	TIMP1	ENSG00000102265.7	7.806	3.088	2.528	0.012
ENSG00000253605.1_CTD-3064M3.6	CTD-3064M3.6	ENSG00000253605.1	0.014	0.005	2.528	0.012
ENSG00000232946.1_RP11-390F4.2	RP11-390F4.2	ENSG00000232946.1	0.056	0.022	2.527	0.012
ENSG00000149182.9_ARFGAP2	ARFGAP2	ENSG00000149182.9	1.726	0.683	2.527	0.012
ENSG00000175309.10_AGXT2L2	AGXT2L2	ENSG00000175309.10	0.999	0.395	2.527	0.012
ENSG00000160339.11_FCN2	FCN2	ENSG00000160339.11	0.021	0.008	2.527	0.012
ENSG00000112977.11_DAP	DAP	ENSG00000112977.11	1.011	0.400	2.527	0.012
ENSG00000133055.4_MYBPH	MYBPH	ENSG00000133055.4	0.286	0.113	2.526	0.012
ENSG00000114316.8_USP4	USP4	ENSG00000114316.8	0.690	0.273	2.525	0.012
ENSG00000154928.12_EPHB1	EPHB1	ENSG00000154928.12	0.343	0.136	2.523	0.013
ENSG00000198223.9_CSF2RA	CSF2RA	ENSG00000198223.9	0.458	0.182	2.523	0.013
ENSG00000253898.1_RP11-51M18.1	RP11-51M18.1	ENSG00000253898.1	0.001	0.001	2.522	0.013
ENSG00000225398.2_AL078621.5	AL078621.5	ENSG00000225398.2	0.198	0.079	2.519	0.013
ENSG00000256545.1_AL139819.1	AL139819.1	ENSG00000256545.1	0.019	0.008	2.518	0.013
ENSG00000263818.1_CTD-2206N4.4	CTD-2206N4.4	ENSG00000263818.1	0.024	0.009	2.518	0.013
ENSG00000166801.11_FAM111A	FAM111A	ENSG00000166801.11	0.241	0.096	2.515	0.013
ENSG00000134569.5_LRP4	LRP4	ENSG00000134569.5	4.544	1.808	2.513	0.013
ENSG00000172113.4_NME6	NME6	ENSG00000172113.4	0.425	0.169	2.513	0.013
ENSG00000164976.7_KIAA1161	KIAA1161	ENSG00000164976.7	1.363	0.542	2.512	0.013
ENSG00000255108.1_AP006621.8	AP006621.8	ENSG00000255108.1	0.034	0.014	2.512	0.013
ENSG00000129353.10_SLC44A2	SLC44A2	ENSG00000129353.10	4.085	1.627	2.512	0.013
ENSG00000267551.1_AC005264.2	AC005264.2	ENSG00000267551.1	0.007	0.003	2.511	0.013
ENSG00000102935.7_ZNF423	ZNF423	ENSG00000102935.7	0.591	0.235	2.510	0.013
ENSG00000254321.1_RP11-495O10.1	RP11-495O10.1	ENSG00000254321.1	0.011	0.004	2.510	0.013
ENSG00000213972.3_RP3-522P13.2	RP3-522P13.2	ENSG00000213972.3	0.020	0.008	2.510	0.013
ENSG00000260800.1_RP11-294K24.4	RP11-294K24.4	ENSG00000260800.1	3.255	1.297	2.510	0.013
ENSG00000211584.9_SLC48A1	SLC48A1	ENSG00000211584.9	3.035	1.209	2.510	0.013
ENSG00000250385.1_RP11-310P5.2	RP11-310P5.2	ENSG00000250385.1	0.009	0.004	2.509	0.013
ENSG00000253813.1_RP11-384C12.2	RP11-384C12.2	ENSG00000253813.1	0.029	0.011	2.509	0.013
ENSG00000167676.3_PLIN4	PLIN4	ENSG00000167676.3	0.485	0.193	2.509	0.013
ENSG00000110876.8_SELPLG	SELPLG	ENSG00000110876.8	0.607	0.242	2.507	0.013
ENSG00000237620.1_GCNT1P5	GCNT1P5	ENSG00000237620.1	0.094	0.038	2.502	0.013
ENSG00000143842.10_SOX13	SOX13	ENSG00000143842.10	0.816	0.326	2.502	0.013
ENSG00000231486.3_IGKJ5	IGKJ5	ENSG00000231486.3	0.044	0.018	2.501	0.013
ENSG00000042980.8_ADAM28	ADAM28	ENSG00000042980.8	0.279	0.112	2.501	0.013
ENSG00000170915.8_PAQR8	PAQR8	ENSG00000170915.8	2.499	0.999	2.501	0.013
ENSG00000162407.8_PPAP2B	PPAP2B	ENSG00000162407.8	6.331	2.534	2.499	0.013
ENSG00000236110.1_RP11-113A10.5	RP11-113A10.5	ENSG00000236110.1	0.127	0.051	2.497	0.013
ENSG00000244706.2_RP11-298O21.3	RP11-298O21.3	ENSG00000244706.2	0.049	0.020	2.497	0.013
ENSG00000227711.1_RP11-275O4.3	RP11-275O4.3	ENSG00000227711.1	0.002	0.001	2.497	0.013

ENSG00000226581.1_RP11-340I6.8	RP11-340I6.8	ENSG00000226581.1	0.038	0.015	2.497	0.013
ENSG00000241935.4_HOGA1	HOGA1	ENSG00000241935.4	0.264	0.106	2.495	0.014
ENSG00000153944.6_MSI2	MSI2	ENSG00000153944.6	2.245	0.900	2.495	0.014
ENSG00000251372.1_LINC00499	LINC00499	ENSG00000251372.1	1.028	0.412	2.494	0.014
ENSG00000142347.11_MYO1F	MYO1F	ENSG00000142347.11	0.268	0.108	2.494	0.014
ENSG00000170989.8_S1PR1	S1PR1	ENSG00000170989.8	2.388	0.958	2.492	0.014
ENSG00000132376.14_INPP5K	INPP5K	ENSG00000132376.14	0.497	0.200	2.490	0.014
ENSG00000165633.6_VSTM4	VSTM4	ENSG00000165633.6	0.152	0.061	2.490	0.014
ENSG00000163295.4_ALPI	ALPI	ENSG00000163295.4	0.026	0.010	2.489	0.014
ENSG00000196689.6_TRPV1	TRPV1	ENSG00000196689.6	0.467	0.188	2.489	0.014
ENSG00000124334.11_IL9R	IL9R	ENSG00000124334.11	0.017	0.007	2.488	0.014
ENSG00000166507.9_NDST2	NDST2	ENSG00000166507.9	0.269	0.108	2.488	0.014
ENSG00000126005.10_MT1P3	MT1P3	ENSG00000126005.10	2.728	1.097	2.488	0.014
ENSG00000182759.3_MAFA	MAFA	ENSG00000182759.3	0.021	0.009	2.487	0.014
ENSG00000105131.2_EPHX3	EPHX3	ENSG00000105131.2	0.024	0.010	2.487	0.014
ENSG00000267206.1_LCN6	LCN6	ENSG00000267206.1	0.253	0.102	2.487	0.014
ENSG00000043355.6_ZIC2	ZIC2	ENSG00000043355.6	0.805	0.324	2.487	0.014
ENSG00000138696.6_BMPR1B	BMPR1B	ENSG00000138696.6	0.563	0.226	2.487	0.014
ENSG00000124615.13_MOCS1	MOCS1	ENSG00000124615.13	0.338	0.136	2.486	0.014
ENSG00000125434.6_SLC25A35	SLC25A35	ENSG00000125434.6	0.226	0.091	2.486	0.014
ENSG00000149499.6_EML3	EML3	ENSG00000149499.6	2.213	0.891	2.485	0.014
ENSG00000129535.8_NRL	NRL	ENSG00000129535.8	0.237	0.096	2.482	0.014
ENSG00000182871.10_COL18A1	COL18A1	ENSG00000182871.10	0.844	0.340	2.481	0.014
ENSG00000173349.4_SFT2D3	SFT2D3	ENSG00000173349.4	0.094	0.038	2.481	0.014
ENSG00000117318.8_ID3	ID3	ENSG00000117318.8	4.725	1.905	2.480	0.014
ENSG00000179761.6_PIPOX	PIPOX	ENSG00000179761.6	0.513	0.207	2.480	0.014
ENSG00000235718.3_MFRP	MFRP	ENSG00000235718.3	0.002	0.001	2.480	0.014
ENSG00000113657.8_DPYSL3	DPYSL3	ENSG00000113657.8	1.309	0.528	2.480	0.014
ENSG00000236156.1_RP11-397D12.7	RP11-397D12.7	ENSG00000236156.1	0.080	0.032	2.480	0.014
ENSG00000131477.6_RAMP2	RAMP2	ENSG00000131477.6	2.127	0.858	2.480	0.014
ENSG00000251168.1_CTD-2072I24.1	CTD-2072I24.1	ENSG00000251168.1	0.005	0.002	2.480	0.014
ENSG00000139990.13_DCAF5	DCAF5	ENSG00000139990.13	0.378	0.152	2.480	0.014
ENSG00000148358.13_GPR107	GPR107	ENSG00000148358.13	0.464	0.187	2.479	0.014
ENSG00000129925.6_TMEM8A	TMEM8A	ENSG00000129925.6	0.555	0.224	2.479	0.014
ENSG00000171940.9_ZNF217	ZNF217	ENSG00000171940.9	0.112	0.045	2.478	0.014
ENSG00000264444.1_SDHCP1	SDHCP1	ENSG00000264444.1	0.003	0.001	2.477	0.014
ENSG00000104894.6_CD37	CD37	ENSG00000104894.6	0.794	0.321	2.477	0.014
ENSG00000116815.11_CD58	CD58	ENSG00000116815.11	0.270	0.109	2.476	0.014
ENSG00000141522.7_ARHGDIA	ARHGDIA	ENSG00000141522.7	4.404	1.781	2.473	0.014
ENSG00000128849.9_CGNL1	CGNL1	ENSG00000128849.9	0.824	0.333	2.473	0.014
ENSG00000237709.1_EEF1A1P28	EEF1A1P28	ENSG00000237709.1	0.004	0.002	2.473	0.014
ENSG00000100280.12_AP1B1	AP1B1	ENSG00000100280.12	1.138	0.460	2.473	0.014
ENSG00000076716.7_GPC4	GPC4	ENSG00000076716.7	0.320	0.129	2.473	0.014
ENSG00000227432.1_AC053503.11	AC053503.11	ENSG00000227432.1	0.085	0.034	2.472	0.014
ENSG00000146648.11_EGFR	EGFR	ENSG00000146648.11	0.411	0.166	2.472	0.014
ENSG00000100298.11_APOBEC3H	APOBEC3H	ENSG00000100298.11	0.022	0.009	2.471	0.014
ENSG00000173068.11_BNC2	BNC2	ENSG00000173068.11	0.024	0.010	2.471	0.014
ENSG00000118855.13_MFSD1	MFSD1	ENSG00000118855.13	0.438	0.177	2.470	0.015
ENSG00000204580.7_DDR1	DDR1	ENSG00000204580.7	5.500	2.227	2.470	0.015
ENSG00000172728.11_FUT10	FUT10	ENSG00000172728.11	0.148	0.060	2.469	0.015
ENSG00000144908.9_ALDH1L1	ALDH1L1	ENSG00000144908.9	4.985	2.019	2.468	0.015
ENSG00000257210.1_RP11-202G11.1	RP11-202G11.1	ENSG00000257210.1	0.031	0.013	2.468	0.015
ENSG00000163453.6_IGFBP7	IGFBP7	ENSG00000163453.6	13.283	5.385	2.467	0.015
ENSG00000188130.9_MAPK12	MAPK12	ENSG00000188130.9	1.377	0.558	2.467	0.015
ENSG00000177406.4_RP11-218M22.1	RP11-218M22.1	ENSG00000177406.4	0.144	0.058	2.466	0.015
ENSG00000141736.8_ERBB2	ERBB2	ENSG00000141736.8	0.734	0.298	2.465	0.015
ENSG00000211677.2_IGLC2	IGLC2	ENSG00000211677.2	0.293	0.119	2.465	0.015
ENSG00000148908.10_RGS10	RGS10	ENSG00000148908.10	0.958	0.389	2.462	0.015
ENSG00000084207.11_GSTP1	GSTP1	ENSG00000084207.11	8.995	3.655	2.461	0.015
ENSG00000264215.1_RP11-283C24.1	RP11-283C24.1	ENSG00000264215.1	0.003	0.001	2.461	0.015

ENSG00000137393.8_RNF144B	RNF144B	ENSG00000137393.8	0.215	0.087	2.460	0.015
ENSG00000101986.7_ABCD1	ABCD1	ENSG00000101986.7	0.130	0.053	2.460	0.015
ENSG00000241809.1_CTD-2207L17.1	CTD-2207L17.1	ENSG00000241809.1	0.023	0.010	2.459	0.015
ENSG00000198736.7_MSRB1	MSRB1	ENSG00000198736.7	0.712	0.289	2.459	0.015
ENSG00000176108.5_CHMP6	CHMP6	ENSG00000176108.5	0.711	0.289	2.459	0.015
ENSG00000106336.8_FBXO24	FBXO24	ENSG00000106336.8	0.073	0.030	2.459	0.015
ENSG00000083857.9_FAT1	FAT1	ENSG00000083857.9	0.987	0.401	2.458	0.015
ENSG00000117400.10_MPL	MPL	ENSG00000117400.10	0.019	0.008	2.456	0.015
ENSG00000241032.2_MetazoaSRP	RN7SL709P	ENSG00000241032.2	0.008	0.003	2.456	0.015
ENSG00000100979.10_PLTP	PLTP	ENSG00000100979.10	4.779	1.946	2.455	0.015
ENSG00000007372.14_PAX6	PAX6	ENSG00000007372.14	1.899	0.773	2.455	0.015
ENSG00000125257.9_ABCC4	ABCC4	ENSG00000125257.9	0.073	0.030	2.454	0.015
ENSG00000213557.4_RP11-240E2.2	RP11-240E2.2	ENSG00000213557.4	0.040	0.016	2.452	0.015
ENSG00000183579.11_ZNRF3	ZNRF3	ENSG00000183579.11	0.713	0.291	2.451	0.015
ENSG00000119927.9_GPAM	GPAM	ENSG00000119927.9	0.554	0.226	2.451	0.015
ENSG00000073849.10_ST6GAL1	ST6GAL1	ENSG00000073849.10	0.561	0.229	2.450	0.015
ENSG00000053371.8_AKR7A2	AKR7A2	ENSG00000053371.8	1.214	0.496	2.449	0.015
ENSG00000026025.9_VIM	VIM	ENSG00000026025.9	20.196	8.255	2.446	0.015
ENSG00000175482.4_POLD4	POLD4	ENSG00000175482.4	2.033	0.831	2.445	0.016
ENSG00000241839.5_PLEKHO2	PLEKHO2	ENSG00000241839.5	1.203	0.492	2.445	0.016
ENSG00000162341.10_TPCN2	TPCN2	ENSG00000162341.10	0.205	0.084	2.444	0.016
ENSG00000240533.2_MetazoaSRP	RN7SL69P	ENSG00000240533.2	0.713	0.292	2.443	0.016
ENSG00000260026.1_CTD-2015G9.1	CTD-2015G9.1	ENSG00000260026.1	0.033	0.013	2.442	0.016
ENSG00000177663.8_IL17RA	IL17RA	ENSG00000177663.8	0.213	0.087	2.441	0.016
ENSG00000258920.1_FOXN3-AS1	FOXN3-AS1	ENSG00000258920.1	0.129	0.053	2.441	0.016
ENSG00000115649.11_CNPPD1	CNPPD1	ENSG00000115649.11	0.975	0.399	2.441	0.016
ENSG00000122971.4_ACADS	ACADS	ENSG00000122971.4	0.943	0.387	2.440	0.016
ENSG00000070614.9_NDST1	NDST1	ENSG00000070614.9	0.631	0.259	2.439	0.016
ENSG00000186513.2_OR9Q2	OR9Q2	ENSG00000186513.2	0.000	0.000	2.438	0.016
ENSG00000213145.5_CRIP1	CRIP1	ENSG00000213145.5	1.669	0.685	2.438	0.016
ENSG00000205669.2_ACOT6	ACOT6	ENSG00000205669.2	0.077	0.031	2.435	0.016
ENSG00000234927.1_HMGN1P18	HMGN1P18	ENSG00000234927.1	0.094	0.039	2.434	0.016
ENSG00000182902.8_SLC25A18	SLC25A18	ENSG00000182902.8	8.306	3.412	2.434	0.016
ENSG00000229304.1_RP11-198M6.5	RP11-198M6.5	ENSG00000229304.1	0.031	0.013	2.433	0.016
ENSG00000140836.9_ZFHX3	ZFHX3	ENSG00000140836.9	0.117	0.048	2.432	0.016
ENSG00000223491.1_RP3-328E19.4	RP3-328E19.4	ENSG00000223491.1	3.975	1.634	2.432	0.016
ENSG00000236735.2_RPL31P63	RPL31P63	ENSG00000236735.2	0.025	0.010	2.431	0.016
ENSG00000245864.2_CTC-467M3.1	CTC-467M3.1	ENSG00000245864.2	0.003	0.001	2.431	0.016
ENSG00000159110.14_IFNAR2	IFNAR2	ENSG00000159110.14	0.353	0.145	2.430	0.016
ENSG00000240660.1_RP11-564C4.7	RP11-564C4.7	ENSG00000240660.1	0.005	0.002	2.430	0.016
ENSG00000230415.1_RP5-902P8.10	RP5-902P8.10	ENSG00000230415.1	0.381	0.157	2.430	0.016
ENSG00000134250.12_NOTCH2	NOTCH2	ENSG00000134250.12	0.748	0.308	2.430	0.016
ENSG00000239830.1_RPS4XP22	RPS4XP22	ENSG00000239830.1	0.022	0.009	2.429	0.016
ENSG00000198744.5_RP5-857K21.11	RP5-857K21.11	ENSG00000198744.5	35.469	14.612	2.427	0.016
ENSG00000151491.8_EPS8	EPS8	ENSG00000151491.8	0.510	0.210	2.427	0.016
ENSG00000230484.2_OR51A10P	OR51A10P	ENSG00000230484.2	0.030	0.012	2.427	0.016
ENSG00000232623.1_AP000266.7	AP000266.7	ENSG00000232623.1	0.024	0.010	2.426	0.016
ENSG00000123992.14_DNPEP	DNPEP	ENSG00000123992.14	0.699	0.288	2.426	0.016
ENSG00000213793.2_AC010487.1	AC010487.1	ENSG00000213793.2	1.670	0.688	2.425	0.016
ENSG00000255254.1_HIGD1AP5	HIGD1AP5	ENSG00000255254.1	0.002	0.001	2.425	0.016
ENSG00000267630.1_AC005758.1	AC005758.1	ENSG00000267630.1	0.000	0.000	2.424	0.016
ENSG00000234877.2_AC092660.1	AC092660.1	ENSG00000234877.2	0.000	0.000	2.424	0.016
ENSG00000224069.1_CTD-2183H9.7	CTD-2183H9.7	ENSG00000224069.1	0.000	0.000	2.424	0.016
ENSG00000237603.1_HMGB3P12	HMGB3P12	ENSG00000237603.1	0.002	0.001	2.424	0.016
ENSG00000244425.2_MetazoaSRP	RN7SL268P	ENSG00000244425.2	0.005	0.002	2.424	0.016
ENSG00000198868.3_MIR4461	MIR4461	ENSG00000198868.3	0.003	0.001	2.424	0.016
ENSG00000227029.1_RP11-168P8.3	RP11-168P8.3	ENSG00000227029.1	0.000	0.000	2.424	0.016
ENSG00000257165.1_RP11-171L9.1	RP11-171L9.1	ENSG00000257165.1	0.000	0.000	2.424	0.016
ENSG00000244470.2_RP11-395M19.1	RP11-395M19.1	ENSG00000244470.2	0.000	0.000	2.424	0.016
ENSG00000198022.5_RP11-432N13.4	RP11-432N13.4	ENSG00000198022.5	0.000	0.000	2.424	0.016

ENSG00000253341.1_RP11-730G20.2	RP11-730G20.2	ENSG00000253341.1	0.000	0.000	2.424	0.016
ENSG00000257137.1_RP11-845M18.3	RP11-845M18.3	ENSG00000257137.1	0.000	0.000	2.424	0.016
ENSG00000182271.8_TMIGD1	TMIGD1	ENSG00000182271.8	0.000	0.000	2.424	0.016
ENSG00000249654.1_VN1R104P	VN1R104P	ENSG00000249654.1	0.000	0.000	2.424	0.016
ENSG00000165029.11_ABCA1	ABCA1	ENSG00000165029.11	0.112	0.046	2.423	0.016
ENSG00000241884.1_RP11-85I21.1	RP11-85I21.1	ENSG00000241884.1	0.001	0.001	2.423	0.016
ENSG00000232797.1_FAM207CP	FAM207CP	ENSG00000232797.1	0.777	0.321	2.423	0.016
ENSG00000265408.1_RP11-361L15.4	RP11-361L15.4	ENSG00000265408.1	0.002	0.001	2.423	0.016
ENSG00000243321.1_RP11-167H9.3	RP11-167H9.3	ENSG00000243321.1	0.034	0.014	2.419	0.017
ENSG00000138356.9_AOX1	AOX1	ENSG00000138356.9	0.033	0.014	2.419	0.017
ENSG00000155438.6_MKI67IP	MKI67IP	ENSG00000155438.6	0.438	0.181	2.416	0.017
ENSG00000211899.3_IGHM	IGHM	ENSG00000211899.3	0.133	0.055	2.416	0.017
ENSG00000242829.1_RPS26P21	RPS26P21	ENSG00000242829.1	0.060	0.025	2.415	0.017
ENSG00000101400.5_SNTA1	SNTA1	ENSG00000101400.5	9.046	3.747	2.414	0.017
ENSG00000167508.6_MVD	MVD	ENSG00000167508.6	3.846	1.594	2.413	0.017
ENSG00000259605.2_AC074212.5	AC074212.5	ENSG00000259605.2	0.141	0.058	2.413	0.017
ENSG00000245322.2_RP11-15B17.1	RP11-15B17.1	ENSG00000245322.2	0.043	0.018	2.413	0.017
ENSG00000161671.11_EMC10	EMC10	ENSG00000161671.11	2.027	0.840	2.413	0.017
ENSG00000243629.1_RP11-6F2.4	RP11-6F2.4	ENSG00000243629.1	0.010	0.004	2.413	0.017
ENSG00000111058.3_ACSS3	ACSS3	ENSG00000111058.3	0.513	0.213	2.412	0.017
ENSG00000176087.10_SLC35A4	SLC35A4	ENSG00000176087.10	0.580	0.240	2.412	0.017
ENSG00000214268.2_RPS3AP33	RPS3AP33	ENSG00000214268.2	0.037	0.016	2.411	0.017
ENSG00000228408.1_RP1-111D6.3	RP1-111D6.3	ENSG00000228408.1	1.507	0.625	2.411	0.017
ENSG00000101850.8_GPR143	GPR143	ENSG00000101850.8	0.385	0.160	2.411	0.017
ENSG00000179950.9_PUF60	PUF60	ENSG00000179950.9	1.986	0.824	2.410	0.017
ENSG00000237441.4_RGL2	RGL2	ENSG00000237441.4	1.469	0.610	2.409	0.017
ENSG00000160191.13_PDE9A	PDE9A	ENSG00000160191.13	0.460	0.191	2.409	0.017
ENSG00000204740.5_C10orf112	C10orf112	ENSG00000204740.5	0.038	0.016	2.408	0.017
ENSG00000232537.1_RP11-385M4.1	RP11-385M4.1	ENSG00000232537.1	0.015	0.006	2.408	0.017
ENSG00000226479.3_TMEM185B	TMEM185B	ENSG00000226479.3	0.220	0.091	2.408	0.017
ENSG00000174990.3_CA5A	CA5A	ENSG00000174990.3	0.070	0.029	2.407	0.017
ENSG00000237417.1_RP11-280G19.2	RP11-280G19.2	ENSG00000237417.1	0.001	0.000	2.406	0.017
ENSG00000223923.1_AC010136.2	AC010136.2	ENSG00000223923.1	0.035	0.015	2.406	0.017
ENSG00000244301.1_AC007163.4	AC007163.4	ENSG00000244301.1	0.020	0.008	2.405	0.017
ENSG00000163382.7_APOA1BP	APOA1BP	ENSG00000163382.7	1.717	0.714	2.404	0.017
ENSG00000234509.1_AP000253.1	AP000253.1	ENSG00000234509.1	0.043	0.018	2.399	0.018
ENSG00000149809.10_TM7SF2	TM7SF2	ENSG00000149809.10	1.726	0.720	2.398	0.018
ENSG00000226549.3_SCDP1	SCDP1	ENSG00000226549.3	0.001	0.000	2.398	0.018
ENSG00000071626.11_DAZAP1	DAZAP1	ENSG00000071626.11	0.835	0.348	2.398	0.018
ENSG00000235926.1_RP13-650G11.1	RP13-650G11.1	ENSG00000235926.1	0.000	0.000	2.397	0.018
ENSG00000069702.6_TGFBR3	TGFBR3	ENSG00000069702.6	0.298	0.124	2.397	0.018
ENSG00000158863.16_FAM160B2	FAM160B2	ENSG00000158863.16	0.944	0.394	2.396	0.018
ENSG00000158428.3_C2orf62	C2orf62	ENSG00000158428.3	0.070	0.029	2.395	0.018
ENSG00000198931.6_APRT	APRT	ENSG00000198931.6	1.462	0.610	2.395	0.018
ENSG00000165905.12_GYLTL1B	GYLTL1B	ENSG00000165905.12	0.018	0.007	2.395	0.018
ENSG00000078549.10_ADCYAP1R1	ADCYAP1R1	ENSG00000078549.10	2.002	0.836	2.394	0.018
ENSG00000007384.10_RHBDF1	RHBDF1	ENSG00000007384.10	0.761	0.318	2.393	0.018
ENSG00000100162.10_CENPM	CENPM	ENSG00000100162.10	0.154	0.064	2.392	0.018
ENSG00000258465.1_RP11-574F21.3	RP11-574F21.3	ENSG00000258465.1	0.022	0.009	2.390	0.018
ENSG00000243403.1_RP11-330L19.1	RP11-330L19.1	ENSG00000243403.1	0.107	0.045	2.389	0.018
ENSG00000167258.8_CDK12	CDK12	ENSG00000167258.8	0.170	0.071	2.388	0.018
ENSG00000198523.5_PLN	PLN	ENSG00000198523.5	0.060	0.025	2.387	0.018
ENSG00000185920.10_PTCH1	PTCH1	ENSG00000185920.10	0.412	0.173	2.386	0.018
ENSG00000123989.9_CHPF	CHPF	ENSG00000123989.9	1.278	0.536	2.386	0.018
ENSG00000087266.11_SH3BP2	SH3BP2	ENSG00000087266.11	1.130	0.474	2.386	0.018
ENSG00000169992.5_NLGN2	NLGN2	ENSG00000169992.5	1.544	0.647	2.386	0.018
ENSG00000254979.1_RP11-872D17.8	RP11-872D17.8	ENSG00000254979.1	0.020	0.008	2.384	0.018
ENSG00000105221.10_AKT2	AKT2	ENSG00000105221.10	1.357	0.570	2.383	0.018
ENSG00000230311.1_AC004074.3	AC004074.3	ENSG00000230311.1	0.001	0.000	2.382	0.018
ENSG00000214535.3_RPS15AP1	RPS15AP1	ENSG00000214535.3	0.004	0.002	2.382	0.018

ENSG00000130287.7_NCAN	NCAN	ENSG00000130287.7	3.169	1.331	2.380	0.018
ENSG00000243466.1_IGKV1-5	IGKV1-5	ENSG00000243466.1	0.016	0.007	2.380	0.018
ENSG00000155918.3_RAET1L	RAET1L	ENSG00000155918.3	0.016	0.007	2.378	0.019
ENSG00000114779.15_ABHD14B	ABHD14B	ENSG00000114779.15	0.618	0.260	2.378	0.019
ENSG00000164199.11_GPR98	GPR98	ENSG00000164199.11	1.331	0.560	2.376	0.019
ENSG00000139531.7_SUOX	SUOX	ENSG00000139531.7	0.418	0.176	2.375	0.019
ENSG00000230184.1_SMYD3-IT1	SMYD3-IT1	ENSG00000230184.1	0.035	0.015	2.375	0.019
ENSG00000108784.5_NAGLU	NAGLU	ENSG00000108784.5	0.517	0.218	2.374	0.019
ENSG00000140545.10_MFGE8	MFGE8	ENSG00000140545.10	2.664	1.122	2.374	0.019
ENSG00000163359.10_COL6A3	COL6A3	ENSG00000163359.10	0.021	0.009	2.373	0.019
ENSG00000136935.8_GOLGA1	GOLGA1	ENSG00000136935.8	0.147	0.062	2.373	0.019
ENSG00000164850.10_GPER	GPER	ENSG00000164850.10	1.109	0.467	2.372	0.019
ENSG00000173638.14_SLC19A1	SLC19A1	ENSG00000173638.14	1.463	0.617	2.372	0.019
ENSG00000197858.6_GPAA1	GPAA1	ENSG00000197858.6	1.434	0.605	2.372	0.019
ENSG00000105329.4_TGFB1	TGFB1	ENSG00000105329.4	0.898	0.379	2.371	0.019
ENSG00000151117.3_TMEM86A	TMEM86A	ENSG00000151117.3	0.100	0.042	2.370	0.019
ENSG00000185669.5_SNAI3	SNAI3	ENSG00000185669.5	0.052	0.022	2.370	0.019
ENSG00000258186.1_SLC7A5P2	SLC7A5P2	ENSG00000258186.1	0.145	0.061	2.369	0.019
ENSG00000231435.1_AC011747.3	AC011747.3	ENSG00000231435.1	0.008	0.003	2.368	0.019
ENSG00000169291.5_SHE	SHE	ENSG00000169291.5	0.334	0.141	2.367	0.019
ENSG00000260410.1_RP11-505K9.3	RP11-505K9.3	ENSG00000260410.1	0.004	0.002	2.366	0.019
ENSG00000172456.12_FGGY	FGGY	ENSG00000172456.12	0.340	0.144	2.365	0.019
ENSG00000074370.13_ATP2A3	ATP2A3	ENSG00000074370.13	0.164	0.069	2.364	0.019
ENSG00000135916.11_ITM2C	ITM2C	ENSG00000135916.11	34.574	14.624	2.364	0.019
ENSG00000211592.2_IGKC	IGKC	ENSG00000211592.2	0.947	0.401	2.362	0.019
ENSG00000147596.3_PRDM14	PRDM14	ENSG00000147596.3	0.002	0.001	2.361	0.019
ENSG00000262222.1_RP11-876N24.4	RP11-876N24.4	ENSG00000262222.1	0.048	0.021	2.361	0.019
ENSG00000079805.11_DNM2	DNM2	ENSG00000079805.11	1.783	0.756	2.358	0.019
ENSG00000140807.4_NKD1	NKD1	ENSG00000140807.4	0.114	0.048	2.358	0.020
ENSG00000237221.1_PPEF1-AS1	PPEF1-AS1	ENSG00000237221.1	0.021	0.009	2.357	0.020
ENSG00000266709.1_RP11-214O1.2	RP11-214O1.2	ENSG00000266709.1	0.013	0.006	2.357	0.020
ENSG00000126016.9_AMOT	AMOT	ENSG00000126016.9	0.978	0.415	2.356	0.020
ENSG00000258917.1_CTD-2175M1.3	CTD-2175M1.3	ENSG00000258917.1	0.024	0.010	2.356	0.020
ENSG00000261837.1_RP11-264L1.3	RP11-264L1.3	ENSG00000261837.1	0.041	0.018	2.355	0.020
ENSG00000100324.8_TAB1	TAB1	ENSG00000100324.8	0.673	0.286	2.354	0.020
ENSG00000114204.10_SERPINI2	SERPINI2	ENSG00000114204.10	0.123	0.052	2.354	0.020
ENSG00000125457.9_MIF4GD	MIF4GD	ENSG00000125457.9	0.496	0.211	2.353	0.020
ENSG00000122678.10_POLM	POLM	ENSG00000122678.10	0.319	0.136	2.352	0.020
ENSG00000215494.1_AP001619.1	AP001619.1	ENSG00000215494.1	0.068	0.029	2.352	0.020
ENSG00000213064.4_SFT2D2	SFT2D2	ENSG00000213064.4	0.191	0.081	2.352	0.020
ENSG00000223749.2_AC004383.4	AC004383.4	ENSG00000223749.2	0.206	0.088	2.352	0.020
ENSG00000099954.13_CECR2	CECR2	ENSG00000099954.13	0.136	0.058	2.350	0.020
ENSG00000186818.6_LILRB4	LILRB4	ENSG00000186818.6	0.237	0.101	2.350	0.020
ENSG00000229953.1_RP11-284F21.7	RP11-284F21.7	ENSG00000229953.1	0.044	0.019	2.350	0.020
ENSG00000249261.1_RP11-284A20.2	RP11-284A20.2	ENSG00000249261.1	0.014	0.006	2.349	0.020
ENSG00000144040.8_SFXN5	SFXN5	ENSG00000144040.8	7.629	3.249	2.348	0.020
ENSG00000141551.10_CSNK1D	CSNK1D	ENSG00000141551.10	1.423	0.606	2.348	0.020
ENSG00000091409.10_ITGA6	ITGA6	ENSG00000091409.10	0.691	0.295	2.347	0.020
ENSG00000130300.3_PLVAP	PLVAP	ENSG00000130300.3	0.097	0.041	2.347	0.020
ENSG00000225377.1_RP5-1103G7.4	RP5-1103G7.4	ENSG00000225377.1	0.389	0.166	2.347	0.020
ENSG00000151012.9_SLC7A11	SLC7A11	ENSG00000151012.9	0.525	0.224	2.346	0.020
ENSG00000213398.3_LCAT	LCAT	ENSG00000213398.3	1.224	0.522	2.346	0.020
ENSG00000166333.9_ILK	ILK	ENSG00000166333.9	1.890	0.806	2.344	0.020
ENSG00000226683.1_PWWP2AP1	PWWP2AP1	ENSG00000226683.1	0.005	0.002	2.342	0.020
ENSG00000235836.1_AC124944.4	AC124944.4	ENSG00000235836.1	0.010	0.004	2.342	0.020
ENSG00000168890.9_TMEM150A	TMEM150A	ENSG00000168890.9	0.426	0.182	2.342	0.020
ENSG00000127329.10_PTPRB	PTPRB	ENSG00000127329.10	0.440	0.188	2.340	0.020
ENSG00000253147.1_RP11-369E15.4	RP11-369E15.4	ENSG00000253147.1	0.011	0.005	2.340	0.020
ENSG00000122729.12_ACO1	ACO1	ENSG00000122729.12	0.421	0.180	2.340	0.020
ENSG00000249388.1_RP11-834C11.6	RP11-834C11.6	ENSG00000249388.1	0.127	0.054	2.338	0.021

ENSG00000226405.1_RP5-984P4.1	RP5-984P4.1	ENSG00000226405.1	0.029	0.012	2.338	0.021
ENSG00000249188.1_RP11-556O5.1	RP11-556O5.1	ENSG00000249188.1	0.022	0.009	2.338	0.021
ENSG00000254508.1_RP11-646J21.5	RP11-646J21.5	ENSG00000254508.1	0.025	0.011	2.337	0.021
ENSG00000204850.3_AC011484.1	AC011484.1	ENSG00000204850.3	0.035	0.015	2.337	0.021
ENSG00000075234.12_TTC38	TTC38	ENSG00000075234.12	0.528	0.226	2.337	0.021
ENSG00000214814.2_FER1L6	FER1L6	ENSG00000214814.2	0.006	0.003	2.336	0.021
ENSG00000236031.1_RP11-269F20.1	RP11-269F20.1	ENSG00000236031.1	0.014	0.006	2.335	0.021
ENSG00000197146.2_AL133458.1	AL133458.1	ENSG00000197146.2	0.376	0.161	2.335	0.021
ENSG00000170190.11_SLC16A5	SLC16A5	ENSG00000170190.11	0.050	0.022	2.334	0.021
ENSG00000205517.8_RGL3	RGL3	ENSG00000205517.8	0.953	0.408	2.332	0.021
ENSG00000240184.2_PCDHGC3	PCDHGC3	ENSG00000240184.2	4.552	1.952	2.332	0.021
ENSG00000168389.13_MFSD2A	MFSD2A	ENSG00000168389.13	1.451	0.622	2.331	0.021
ENSG00000259839.1_RP11-491F9.6	RP11-491F9.6	ENSG00000259839.1	0.026	0.011	2.331	0.021
ENSG00000120907.13_ADRA1A	ADRA1A	ENSG00000120907.13	0.102	0.044	2.330	0.021
ENSG00000186815.8_TPCN1	TPCN1	ENSG00000186815.8	1.660	0.712	2.330	0.021
ENSG00000210841.1_U6atac	U6atac	ENSG00000210841.1	0.002	0.001	2.329	0.021
ENSG00000169515.5_CCDC8	CCDC8	ENSG00000169515.5	0.211	0.091	2.328	0.021
ENSG00000172037.9_LAMB2	LAMB2	ENSG00000172037.9	5.741	2.466	2.328	0.021
ENSG00000217783.2_LDHAL6FP	LDHAL6FP	ENSG00000217783.2	0.012	0.005	2.327	0.021
ENSG00000229206.1_RP11-397O4.1	RP11-397O4.1	ENSG00000229206.1	0.137	0.059	2.327	0.021
ENSG00000259540.1_RP11-526I2.1	RP11-526I2.1	ENSG00000259540.1	0.008	0.003	2.326	0.021
ENSG00000231369.1_RP1-40G4P.1	RP1-40G4P.1	ENSG00000231369.1	0.014	0.006	2.326	0.021
ENSG00000076555.11_ACACB	ACACB	ENSG00000076555.11	1.134	0.487	2.326	0.021
ENSG00000163623.4_NKX6-1	NKX6-1	ENSG00000163623.4	0.038	0.017	2.324	0.021
ENSG00000235387.1_RP11-327L3.1	RP11-327L3.1	ENSG00000235387.1	0.255	0.110	2.323	0.021
ENSG00000256628.1_RP11-454H13.5	RP11-454H13.5	ENSG00000256628.1	0.086	0.037	2.322	0.021
ENSG00000072121.11_ZFYVE26	ZFYVE26	ENSG00000072121.11	0.104	0.045	2.322	0.021
ENSG00000267594.1_CYP4F24P	CYP4F24P	ENSG00000267594.1	0.387	0.167	2.321	0.021
ENSG00000162551.9_ALPL	ALPL	ENSG00000162551.9	1.214	0.523	2.321	0.021
ENSG00000161714.7_PLCD3	PLCD3	ENSG00000161714.7	1.979	0.852	2.321	0.021
ENSG00000012211.8_PRICKLE3	PRICKLE3	ENSG00000012211.8	0.131	0.056	2.320	0.022
ENSG00000182551.7_ADI1	ADI1	ENSG00000182551.7	1.850	0.797	2.320	0.022
ENSG00000231711.1_RP11-398F12.1	RP11-398F12.1	ENSG00000231711.1	0.096	0.042	2.319	0.022
ENSG00000220666.2_RCC2P7	RCC2P7	ENSG00000220666.2	0.007	0.003	2.318	0.022
ENSG00000143140.6_GJA5	GJA5	ENSG00000143140.6	0.127	0.055	2.317	0.022
ENSG00000232633.3_CTD-2201G3.1	CTD-2201G3.1	ENSG00000232633.3	0.028	0.012	2.317	0.022
ENSG00000173599.9_PC	PC	ENSG00000173599.9	2.160	0.933	2.317	0.022
ENSG00000081692.8_JMJD4	JMJD4	ENSG00000081692.8	0.411	0.177	2.316	0.022
ENSG00000109321.6_AREG	AREG	ENSG00000109321.6	0.086	0.037	2.316	0.022
ENSG00000258443.1_RP3-414A15.11	RP3-414A15.11	ENSG00000258443.1	0.010	0.004	2.315	0.022
ENSG00000252812.1_RN7SKP2	RN7SKP2	ENSG00000252812.1	0.002	0.001	2.315	0.022
ENSG00000185483.7_ROR1	ROR1	ENSG00000185483.7	0.038	0.017	2.315	0.022
ENSG00000242280.1_RP4-669B10.4	RP4-669B10.4	ENSG00000242280.1	0.001	0.000	2.315	0.022
ENSG00000256588.1_RP11-613F22.8	RP11-613F22.8	ENSG00000256588.1	0.020	0.009	2.314	0.022
ENSG00000180448.6_HMHA1	HMHA1	ENSG00000180448.6	0.419	0.181	2.314	0.022
ENSG00000109107.9_ALDOC	ALDOC	ENSG00000109107.9	36.881	15.947	2.313	0.022
ENSG00000099203.2_TMED1	TMED1	ENSG00000099203.2	0.574	0.248	2.312	0.022
ENSG00000186866.12_POFUT2	POFUT2	ENSG00000186866.12	0.352	0.152	2.311	0.022
ENSG00000168763.11_CNNM3	CNNM3	ENSG00000168763.11	0.252	0.109	2.311	0.022
ENSG00000142973.8_CYP4B1	CYP4B1	ENSG00000142973.8	0.127	0.055	2.310	0.022
ENSG00000204301.5_NOTCH4	NOTCH4	ENSG00000204301.5	1.317	0.571	2.308	0.022
ENSG00000160172.6_FAM86C2P	FAM86C2P	ENSG00000160172.6	0.079	0.034	2.308	0.022
ENSG00000240122.1_FABP5P11	FABP5P11	ENSG00000240122.1	0.021	0.009	2.307	0.022
ENSG00000197479.4_PCDHB11	PCDHB11	ENSG00000197479.4	0.118	0.051	2.306	0.022
ENSG00000119772.12_DNMT3A	DNMT3A	ENSG00000119772.12	0.208	0.090	2.306	0.022
ENSG00000130304.11_SLC27A1	SLC27A1	ENSG00000130304.11	1.534	0.665	2.306	0.022
ENSG00000230069.3_LRRC37A15P	LRRC37A15P	ENSG00000230069.3	0.016	0.007	2.304	0.022
ENSG00000167701.9_GPT	GPT	ENSG00000167701.9	0.503	0.219	2.303	0.023
ENSG00000124097.7_HMGB1P1	HMGB1P1	ENSG00000124097.7	0.037	0.016	2.302	0.023
ENSG00000161999.6_JMJD8	JMJD8	ENSG00000161999.6	0.646	0.281	2.301	0.023

ENSG00000258137.1_RP11-753H16.3	RP11-753H16.3	ENSG00000258137.1	0.027	0.012	2.301	0.023
ENSG00000229820.1_RP1-308E4.1	RP1-308E4.1	ENSG00000229820.1	0.075	0.033	2.301	0.023
ENSG00000010319.2_SEMA3G	SEMA3G	ENSG00000010319.2	0.572	0.249	2.301	0.023
ENSG00000119655.4_NPC2	NPC2	ENSG00000119655.4	2.655	1.155	2.299	0.023
ENSG00000182218.5_HHIPL1	HHIPL1	ENSG00000182218.5	0.364	0.158	2.298	0.023
ENSG00000102575.6_ACP5	ACP5	ENSG00000102575.6	0.082	0.036	2.297	0.023
ENSG00000015285.6_WAS	WAS	ENSG00000015285.6	0.284	0.124	2.296	0.023
ENSG00000131435.8_PDLIM4	PDLIM4	ENSG00000131435.8	1.683	0.733	2.295	0.023
ENSG00000229671.1_AC051649.16	AC051649.16	ENSG00000229671.1	0.097	0.042	2.295	0.023
ENSG00000126752.7_SSX1	SSX1	ENSG00000126752.7	0.003	0.001	2.295	0.023
ENSG00000250938.1_RP11-679C8.2	RP11-679C8.2	ENSG00000250938.1	0.048	0.021	2.294	0.023
ENSG00000253964.1_RPL31P40	RPL31P40	ENSG00000253964.1	0.023	0.010	2.294	0.023
ENSG00000235959.1_AC009237.17	AC009237.17	ENSG00000235959.1	0.015	0.007	2.293	0.023
ENSG00000227120.1_AC009238.7	AC009238.7	ENSG00000227120.1	0.015	0.007	2.293	0.023
ENSG00000134508.8_CABLES1	CABLES1	ENSG00000134508.8	3.078	1.342	2.293	0.023
ENSG00000054179.6_ENTPD2	ENTPD2	ENSG00000054179.6	1.187	0.518	2.292	0.023
ENSG00000018625.10_ATP1A2	ATP1A2	ENSG00000018625.10	24.421	10.655	2.292	0.023
ENSG00000227805.1_RP11-248J23.5	RP11-248J23.5	ENSG00000227805.1	0.163	0.071	2.290	0.023
ENSG00000105357.10_MYH14	MYH14	ENSG00000105357.10	0.729	0.319	2.289	0.023
ENSG00000177558.3_FAM187B	FAM187B	ENSG00000177558.3	0.013	0.006	2.288	0.023
ENSG00000255767.1_RP13-512J5.1	RP13-512J5.1	ENSG00000255767.1	0.144	0.063	2.288	0.023
ENSG00000008516.12_MMP25	MMP25	ENSG00000008516.12	0.052	0.023	2.287	0.023
ENSG00000255320.1_RP11-755F10.1	RP11-755F10.1	ENSG00000255320.1	0.064	0.028	2.286	0.023
ENSG00000136932.9_C9orf156	C9orf156	ENSG00000136932.9	0.212	0.093	2.286	0.023
ENSG00000130876.6_SLC7A10	SLC7A10	ENSG00000130876.6	0.893	0.391	2.286	0.024
ENSG00000255355.1_AP000640.2	AP000640.2	ENSG00000255355.1	0.025	0.011	2.285	0.024
ENSG00000250670.1_AC004063.1	AC004063.1	ENSG00000250670.1	0.019	0.008	2.284	0.024
ENSG00000173269.6_MMRN2	MMRN2	ENSG00000173269.6	0.526	0.230	2.283	0.024
ENSG00000105808.11_RASA4	RASA4	ENSG00000105808.11	3.329	1.459	2.283	0.024
ENSG00000148180.10_GSN	GSN	ENSG00000148180.10	9.308	4.079	2.282	0.024
ENSG00000135363.7_LMO2	LMO2	ENSG00000135363.7	1.672	0.733	2.282	0.024
ENSG00000007866.14_TEAD3	TEAD3	ENSG00000007866.14	0.209	0.092	2.282	0.024
ENSG00000111679.11_PTPN6	PTPN6	ENSG00000111679.11	0.574	0.252	2.282	0.024
ENSG00000077984.4_CST7	CST7	ENSG00000077984.4	0.149	0.065	2.279	0.024
ENSG00000134824.9_FADS2	FADS2	ENSG00000134824.9	6.811	2.988	2.279	0.024
ENSG00000076258.5_FMO4	FMO4	ENSG00000076258.5	0.061	0.027	2.279	0.024
ENSG00000178761.10_FAM219B	FAM219B	ENSG00000178761.10	0.815	0.358	2.279	0.024
ENSG00000263683.1_RP4-777O23.1	RP4-777O23.1	ENSG00000263683.1	0.002	0.001	2.278	0.024
ENSG00000100711.8_ZFYVE21	ZFYVE21	ENSG00000100711.8	4.115	1.807	2.278	0.024
ENSG00000115594.7_IL1R1	IL1R1	ENSG00000115594.7	0.391	0.172	2.278	0.024
ENSG00000143994.9_ABHD1	ABHD1	ENSG00000143994.9	0.210	0.092	2.278	0.024
ENSG00000142748.8_FCN3	FCN3	ENSG00000142748.8	0.148	0.065	2.277	0.024
ENSG00000258655.1_ARHGAP5-AS1	ARHGAP5-AS1	ENSG00000258655.1	0.386	0.169	2.277	0.024
ENSG00000145936.3_KCNMB1	KCNMB1	ENSG00000145936.3	0.072	0.032	2.276	0.024
ENSG00000078295.11_ADCY2	ADCY2	ENSG00000078295.11	2.852	1.254	2.275	0.024
ENSG00000223912.1_EEF1A1P36	EEF1A1P36	ENSG00000223912.1	0.001	0.001	2.275	0.024
ENSG00000179406.6_LINC00174	LINC00174	ENSG00000179406.6	0.222	0.098	2.275	0.024
ENSG00000164743.4_C8orf48	C8orf48	ENSG00000164743.4	0.061	0.027	2.274	0.024
ENSG00000162337.7_LRP5	LRP5	ENSG00000162337.7	0.319	0.140	2.274	0.024
ENSG00000183153.5_GJD3	GJD3	ENSG00000183153.5	0.014	0.006	2.274	0.024
ENSG00000231512.1_RP11-261C10.2	RP11-261C10.2	ENSG00000231512.1	0.027	0.012	2.273	0.024
ENSG00000165071.10_TMEM71	TMEM71	ENSG00000165071.10	0.065	0.029	2.272	0.024
ENSG00000228721.1_AC016909.2	AC016909.2	ENSG00000228721.1	0.001	0.000	2.272	0.024
ENSG00000144043.7_TEX261	TEX261	ENSG00000144043.7	0.612	0.270	2.271	0.024
ENSG00000154319.10_FAM167A	FAM167A	ENSG00000154319.10	1.022	0.450	2.271	0.024
ENSG00000229382.1_BX322557.13	BX322557.13	ENSG00000229382.1	0.004	0.002	2.270	0.024
ENSG00000080573.6_COL5A3	COL5A3	ENSG00000080573.6	3.611	1.590	2.270	0.024
ENSG00000242307.1_RPS26P52	RPS26P52	ENSG00000242307.1	0.003	0.001	2.270	0.024
ENSG00000252311.1_U1	RNU1-103P	ENSG00000252311.1	0.402	0.177	2.269	0.025
ENSG00000089472.12_HEPH	HEPH	ENSG00000089472.12	0.634	0.279	2.268	0.025

ENSG00000237836.1_PHKA2-AS1	PHKA2-AS1	ENSG00000237836.1	0.024	0.011	2.268	0.025
ENSG00000234223.2_AC003988.1	AC003988.1	ENSG00000234223.2	0.001	0.001	2.267	0.025
ENSG00000230843.1_RP11-380D15.3	RP11-380D15.3	ENSG00000230843.1	0.001	0.001	2.267	0.025
ENSG00000149782.7_PLCB3	PLCB3	ENSG00000149782.7	0.450	0.198	2.267	0.025
ENSG00000231447.1_RP1-14D6.7	RP1-14D6.7	ENSG00000231447.1	0.023	0.010	2.267	0.025
ENSG00000248278.1_RP11-463M16.4	RP11-463M16.4	ENSG00000248278.1	0.056	0.025	2.266	0.025
ENSG00000187634.6_SAMD11	SAMD11	ENSG00000187634.6	0.193	0.085	2.266	0.025
ENSG00000258583.1_RP11-112J1.2	RP11-112J1.2	ENSG00000258583.1	0.013	0.006	2.265	0.025
ENSG00000196981.2_WDR5B	WDR5B	ENSG00000196981.2	0.051	0.023	2.265	0.025
ENSG00000196436.6_NPIPL2	NPIPL2	ENSG00000196436.6	1.062	0.469	2.264	0.025
ENSG00000187553.5_CYP26C1	CYP26C1	ENSG00000187553.5	0.012	0.005	2.264	0.025
ENSG00000214021.10_TTLL3	TTLL3	ENSG00000214021.10	1.112	0.491	2.263	0.025
ENSG00000236290.1_RP11-25O10.1	RP11-25O10.1	ENSG00000236290.1	0.022	0.010	2.261	0.025
ENSG00000165555.5_NOXRED1	NOXRED1	ENSG00000165555.5	0.024	0.011	2.260	0.025
ENSG00000146005.3_PSD2	PSD2	ENSG00000146005.3	3.149	1.394	2.260	0.025
ENSG00000186474.10_KLK12	KLK12	ENSG00000186474.10	0.000	0.000	2.259	0.025
ENSG00000203814.5_HIST2H2BF	HIST2H2BF	ENSG00000203814.5	0.322	0.143	2.259	0.025
ENSG00000234156.1_RP11-64P14.7	RP11-64P14.7	ENSG00000234156.1	0.092	0.041	2.258	0.025
ENSG00000124440.10_HIF3A	HIF3A	ENSG00000124440.10	4.319	1.913	2.258	0.025
ENSG00000265352.1_RP11-723G8.2	RP11-723G8.2	ENSG00000265352.1	0.001	0.000	2.258	0.025
ENSG00000078808.11_SDF4	SDF4	ENSG00000078808.11	1.875	0.831	2.257	0.025
ENSG00000233628.1_VN2R5P	VN2R5P	ENSG00000233628.1	0.011	0.005	2.257	0.025
ENSG00000079337.10_RAPGEF3	RAPGEF3	ENSG00000079337.10	3.848	1.705	2.257	0.025
ENSG00000196743.4_GM2A	GM2A	ENSG00000196743.4	0.891	0.395	2.256	0.025
ENSG00000184384.8_MAML2	MAML2	ENSG00000184384.8	0.155	0.069	2.256	0.025
ENSG00000106459.10_NRF1	NRF1	ENSG00000106459.10	0.187	0.083	2.255	0.025
ENSG00000132613.10_MTSS1L	MTSS1L	ENSG00000132613.10	14.895	6.605	2.255	0.025
ENSG00000012779.5_ALOX5	ALOX5	ENSG00000012779.5	0.312	0.138	2.254	0.025
ENSG00000250249.1_RP11-30P21.2	RP11-30P21.2	ENSG00000250249.1	0.008	0.004	2.253	0.026
ENSG00000251243.1_AC005178.1	AC005178.1	ENSG00000251243.1	0.002	0.001	2.252	0.026
ENSG00000160588.5_MPZL3	MPZL3	ENSG00000160588.5	0.020	0.009	2.250	0.026
ENSG00000226102.2_SEPT7P3	SEPT7P3	ENSG00000226102.2	1.124	0.500	2.249	0.026
ENSG00000254568.1_RP11-664I21.5	RP11-664I21.5	ENSG00000254568.1	0.020	0.009	2.249	0.026
ENSG00000148204.7_CRB2	CRB2	ENSG00000148204.7	0.812	0.361	2.249	0.026
ENSG00000044446.6_PHKA2	PHKA2	ENSG00000044446.6	0.326	0.145	2.248	0.026
ENSG00000115607.5_IL18RAP	IL18RAP	ENSG00000115607.5	0.023	0.010	2.246	0.026
ENSG00000255121.2_RP11-110I1.12	RP11-110I1.12	ENSG00000255121.2	0.089	0.040	2.246	0.026
ENSG00000117984.8_CTSD	CTSD	ENSG00000117984.8	7.048	3.139	2.246	0.026
ENSG00000169604.15_ANTXR1	ANTXR1	ENSG00000169604.15	0.512	0.228	2.243	0.026
ENSG00000104886.4_PLEKHJ1	PLEKHJ1	ENSG00000104886.4	1.735	0.774	2.242	0.026
ENSG00000241975.1_TCEB1P19	TCEB1P19	ENSG00000241975.1	0.102	0.046	2.242	0.026
ENSG00000231668.2_AC144536.4	AC144536.4	ENSG00000231668.2	0.008	0.003	2.242	0.026
ENSG00000065534.14_MYLK	MYLK	ENSG00000065534.14	0.906	0.404	2.242	0.026
ENSG00000225246.1_RPS2P1	RPS2P1	ENSG00000225246.1	0.023	0.010	2.240	0.026
ENSG00000145685.8_LHFPL2	LHFPL2	ENSG00000145685.8	0.205	0.091	2.239	0.026
ENSG00000234988.1_AC068538.4	AC068538.4	ENSG00000234988.1	0.015	0.007	2.239	0.026
ENSG00000109111.10_SUPT6H	SUPT6H	ENSG00000109111.10	0.635	0.284	2.238	0.027
ENSG00000229989.2_RP11-31E23.1	RP11-31E23.1	ENSG00000229989.2	0.020	0.009	2.238	0.027
ENSG00000178075.14_GRAMD1C	GRAMD1C	ENSG00000178075.14	0.633	0.283	2.237	0.027
ENSG00000223633.1_RP11-143A22.1	RP11-143A22.1	ENSG00000223633.1	0.002	0.001	2.235	0.027
ENSG00000111254.2_AKAP3	AKAP3	ENSG00000111254.2	0.089	0.040	2.234	0.027
ENSG00000230438.3_RP11-420G6.4	RP11-420G6.4	ENSG00000230438.3	0.098	0.044	2.233	0.027
ENSG00000105552.8_BCAT2	BCAT2	ENSG00000105552.8	1.124	0.504	2.232	0.027
ENSG00000172725.9_CORO1B	CORO1B	ENSG00000172725.9	1.133	0.508	2.231	0.027
ENSG00000239804.1_RP11-379B18.1	RP11-379B18.1	ENSG00000239804.1	0.079	0.036	2.231	0.027
ENSG00000126215.9_XRCC3	XRCC3	ENSG00000126215.9	0.259	0.116	2.231	0.027
ENSG00000212172.1_U1	RNU1-149P	ENSG00000212172.1	0.190	0.085	2.230	0.027
ENSG00000244514.2_MetazoaSRP	RN7SL125P	ENSG00000244514.2	2.644	1.186	2.229	0.027
ENSG00000267014.1_CTD-2081K17.2	CTD-2081K17.2	ENSG00000267014.1	0.155	0.070	2.228	0.027
ENSG00000142039.2_CCDC97	CCDC97	ENSG00000142039.2	0.275	0.124	2.228	0.027

ENSG00000224919.1_RP11-144G6.4	RP11-144G6.4	ENSG00000224919.1	0.001	0.001	2.228	0.027
ENSG00000163346.11_PBXIP1	PBXIP1	ENSG00000163346.11	12.044	5.407	2.227	0.027
ENSG00000230977.1_AC023274.6	AC023274.6	ENSG00000230977.1	0.000	0.000	2.227	0.027
ENSG00000172893.10_DHCR7	DHCR7	ENSG00000172893.10	1.253	0.563	2.227	0.027
ENSG00000011405.9_PIK3C2A	PIK3C2A	ENSG00000011405.9	0.356	0.160	2.227	0.027
ENSG00000238018.1_AC093110.3	AC093110.3	ENSG00000238018.1	0.000	0.000	2.227	0.027
ENSG00000068724.11_TTC7A	TTC7A	ENSG00000068724.11	0.296	0.133	2.224	0.027
ENSG00000130202.5_PVRL2	PVRL2	ENSG00000130202.5	1.152	0.518	2.223	0.028
ENSG00000132554.15_RGS22	RGS22	ENSG00000132554.15	0.062	0.028	2.222	0.028
ENSG00000100767.11_PAPLN	PAPLN	ENSG00000100767.11	1.792	0.807	2.221	0.028
ENSG00000249275.1_RP11-364P22.2	RP11-364P22.2	ENSG00000249275.1	0.019	0.008	2.220	0.028
ENSG00000256238.1_RP11-473N11.2	RP11-473N11.2	ENSG00000256238.1	0.003	0.001	2.219	0.028
ENSG00000172663.4_TMEM134	TMEM134	ENSG00000172663.4	1.459	0.658	2.219	0.028
ENSG00000260611.1_RP11-352B15.2	RP11-352B15.2	ENSG00000260611.1	0.001	0.000	2.218	0.028
ENSG00000135424.11_ITGA7	ITGA7	ENSG00000135424.11	3.871	1.745	2.218	0.028
ENSG00000137948.14_BRDT	BRDT	ENSG00000137948.14	0.003	0.001	2.217	0.028
ENSG00000167565.7_SERTAD3	SERTAD3	ENSG00000167565.7	0.237	0.107	2.217	0.028
ENSG00000150687.6_PRSS23	PRSS23	ENSG00000150687.6	0.668	0.301	2.217	0.028
ENSG00000203883.5_SOX18	SOX18	ENSG00000203883.5	0.852	0.385	2.216	0.028
ENSG00000228923.1_AP000355.2	AP000355.2	ENSG00000228923.1	0.016	0.007	2.216	0.028
ENSG00000054598.5_FOXC1	FOXC1	ENSG00000054598.5	0.257	0.116	2.216	0.028
ENSG00000024422.6_EHD2	EHD2	ENSG00000024422.6	0.905	0.409	2.214	0.028
ENSG00000164292.7_RHOBTB3	RHOBTB3	ENSG00000164292.7	2.792	1.261	2.213	0.028
ENSG00000250742.1_RP11-834C11.4	RP11-834C11.4	ENSG00000250742.1	0.101	0.046	2.213	0.028
ENSG00000258245.1_RP11-114H23.3	RP11-114H23.3	ENSG00000258245.1	0.025	0.011	2.213	0.028
ENSG00000239769.1_RP11-324H6.7	RP11-324H6.7	ENSG00000239769.1	0.031	0.014	2.212	0.028
ENSG00000146250.5_PRSS35	PRSS35	ENSG00000146250.5	0.168	0.076	2.212	0.028
ENSG00000074800.8_ENO1	ENO1	ENSG00000074800.8	14.789	6.687	2.212	0.028
ENSG00000264352.1_MetazoaSRP	RN7SL602P	ENSG00000264352.1	0.197	0.089	2.211	0.028
ENSG00000213673.3_SLC25A5P3	SLC25A5P3	ENSG00000213673.3	0.019	0.009	2.211	0.028
ENSG00000141959.12_PFKL	PFKL	ENSG00000141959.12	3.314	1.500	2.209	0.029
ENSG00000165025.10_SYK	SYK	ENSG00000165025.10	0.111	0.050	2.206	0.029
ENSG00000131386.13_GALNTL2	GALNTL2	ENSG00000131386.13	0.974	0.442	2.205	0.029
ENSG00000235499.1_AC073046.25	AC073046.25	ENSG00000235499.1	0.039	0.018	2.205	0.029
ENSG00000162496.4_DHRS3	DHRS3	ENSG00000162496.4	1.424	0.646	2.203	0.029
ENSG00000173548.8_SNX33	SNX33	ENSG00000173548.8	0.361	0.164	2.203	0.029
ENSG00000254703.1_FLI1-AS1	FLI1-AS1	ENSG00000254703.1	0.094	0.043	2.202	0.029
ENSG00000185664.9_PMEL	PMEL	ENSG00000185664.9	0.019	0.009	2.201	0.029
ENSG00000011600.5_TYROBP	TYROBP	ENSG00000011600.5	3.735	1.699	2.199	0.029
ENSG00000229320.3_KRT8P12	KRT8P12	ENSG00000229320.3	0.172	0.078	2.198	0.029
ENSG00000213995.6_CARKD	CARKD	ENSG00000213995.6	1.031	0.469	2.197	0.029
ENSG00000257645.1_RP11-804F13.2	RP11-804F13.2	ENSG00000257645.1	0.016	0.007	2.197	0.029
ENSG00000035403.11_VCL	VCL	ENSG00000035403.11	0.299	0.136	2.196	0.029
ENSG00000226009.1_RP11-190J1.10	RP11-190J1.10	ENSG00000226009.1	0.035	0.016	2.196	0.029
ENSG00000183762.8_KREMEN1	KREMEN1	ENSG00000183762.8	0.391	0.178	2.195	0.029
ENSG00000157578.7_LCA5L	LCA5L	ENSG00000157578.7	0.057	0.026	2.195	0.030
ENSG00000139835.8_GRTP1	GRTP1	ENSG00000139835.8	0.147	0.067	2.194	0.030
ENSG00000203306.3_AP001007.1	AP001007.1	ENSG00000203306.3	0.076	0.034	2.194	0.030
ENSG00000179776.13_CDH5	CDH5	ENSG00000179776.13	0.712	0.325	2.194	0.030
ENSG00000100385.8_IL2RB	IL2RB	ENSG00000100385.8	0.015	0.007	2.193	0.030
ENSG00000253864.1_AC131025.8	AC131025.8	ENSG00000253864.1	0.048	0.022	2.193	0.030
ENSG00000113209.6_PCDHB5	PCDHB5	ENSG00000113209.6	0.154	0.070	2.193	0.030
ENSG00000154721.9_JAM2	JAM2	ENSG00000154721.9	0.566	0.258	2.192	0.030
ENSG00000177685.11_EFCAB4A	EFCAB4A	ENSG00000177685.11	0.336	0.153	2.192	0.030
ENSG00000218793.1_RP3-382I10.3	RP3-382I10.3	ENSG00000218793.1	0.008	0.004	2.192	0.030
ENSG00000228421.1_AC005013.5	AC005013.5	ENSG00000228421.1	0.027	0.012	2.192	0.030
ENSG00000173801.12_JUP	JUP	ENSG00000173801.12	0.659	0.301	2.191	0.030
ENSG00000135821.11_GLUL	GLUL	ENSG00000135821.11	68.800	31.397	2.191	0.030
ENSG00000249869.1_RP11-93K22.7	RP11-93K22.7	ENSG00000249869.1	0.006	0.003	2.191	0.030
ENSG00000248618.1_RP11-93K22.9	RP11-93K22.9	ENSG00000248618.1	0.219	0.100	2.191	0.030

ENSG00000235239.1_RP1-203C2.4	RP1-203C2.4	ENSG00000235239.1	0.001	0.000	2.191	0.030
ENSG00000241399.2_CD302	CD302	ENSG00000241399.2	0.238	0.109	2.190	0.030
ENSG00000258525.1_RP11-829H16.3	RP11-829H16.3	ENSG00000258525.1	0.014	0.006	2.189	0.030
ENSG00000264275.1_MetazoaSRP	RN7SL753P	ENSG00000264275.1	0.052	0.024	2.189	0.030
ENSG00000259480.1_RP11-26F2.1	RP11-26F2.1	ENSG00000259480.1	0.035	0.016	2.189	0.030
ENSG00000100427.11_MLC1	MLC1	ENSG00000100427.11	10.074	4.604	2.188	0.030
ENSG00000137273.3_FOXF2	FOXF2	ENSG00000137273.3	0.247	0.113	2.188	0.030
ENSG00000184925.6_LCN12	LCN12	ENSG00000184925.6	1.335	0.610	2.188	0.030
ENSG00000120563.4_LYZL1	LYZL1	ENSG00000120563.4	0.003	0.001	2.188	0.030
ENSG00000131152.4_C16orf95	C16orf95	ENSG00000131152.4	0.078	0.036	2.188	0.030
ENSG00000077782.15_FGFR1	FGFR1	ENSG00000077782.15	1.484	0.678	2.187	0.030
ENSG00000232654.1_FAM136BP	FAM136BP	ENSG00000232654.1	0.111	0.051	2.187	0.030
ENSG00000235102.1_RP3-450M14.1	RP3-450M14.1	ENSG00000235102.1	0.003	0.002	2.187	0.030
ENSG00000267772.1_RP11-15E18.4	RP11-15E18.4	ENSG00000267772.1	0.001	0.000	2.186	0.030
ENSG00000134253.5_TRIM45	TRIM45	ENSG00000134253.5	0.231	0.106	2.186	0.030
ENSG00000112473.11_SLC39A7	SLC39A7	ENSG00000112473.11	0.803	0.367	2.185	0.030
ENSG00000188039.9_NWD1	NWD1	ENSG00000188039.9	0.514	0.235	2.185	0.030
ENSG00000228782.3_MRPL45P2	MRPL45P2	ENSG00000228782.3	0.105	0.048	2.185	0.030
ENSG00000254665.1_RP11-152H18.3	RP11-152H18.3	ENSG00000254665.1	0.016	0.007	2.182	0.030
ENSG00000228838.1_RP4-784A16.2	RP4-784A16.2	ENSG00000228838.1	0.000	0.000	2.181	0.031
ENSG00000172000.3_ZNF556	ZNF556	ENSG00000172000.3	0.018	0.008	2.180	0.031
ENSG00000165474.5_GJB2	GJB2	ENSG00000165474.5	0.120	0.055	2.179	0.031
ENSG00000215533.4_LINC00189	LINC00189	ENSG00000215533.4	0.014	0.006	2.179	0.031
ENSG00000003400.10_CASP10	CASP10	ENSG00000003400.10	0.097	0.044	2.178	0.031
ENSG00000257253.1_RP11-70F11.7	RP11-70F11.7	ENSG00000257253.1	0.006	0.003	2.178	0.031
ENSG00000119737.5_GPR75	GPR75	ENSG00000119737.5	0.354	0.163	2.178	0.031
ENSG00000264659.1_CTD-2006K23.2	CTD-2006K23.2	ENSG00000264659.1	0.003	0.001	2.178	0.031
ENSG00000099139.8_PCSK5	PCSK5	ENSG00000099139.8	0.064	0.029	2.178	0.031
ENSG00000031081.6_ARHGAP31	ARHGAP31	ENSG00000031081.6	0.244	0.112	2.177	0.031
ENSG00000175463.7_TBC1D10C	TBC1D10C	ENSG00000175463.7	0.046	0.021	2.177	0.031
ENSG00000186994.6_KANK3	KANK3	ENSG00000186994.6	0.753	0.346	2.176	0.031
ENSG00000227398.2_RP11-447D11.2	RP11-447D11.2	ENSG00000227398.2	0.058	0.026	2.176	0.031
ENSG00000129566.8_TEP1	TEP1	ENSG00000129566.8	0.202	0.093	2.175	0.031
ENSG00000228434.1_AC004951.6	AC004951.6	ENSG00000228434.1	1.112	0.511	2.175	0.031
ENSG00000267582.1_CTD-3252C9.2	CTD-3252C9.2	ENSG00000267582.1	0.003	0.001	2.175	0.031
ENSG00000213757.3_CTC-451P13.1	CTC-451P13.1	ENSG00000213757.3	0.016	0.007	2.175	0.031
ENSG00000106100.6_NOD1	NOD1	ENSG00000106100.6	0.139	0.064	2.175	0.031
ENSG00000166526.12_ZNF3	ZNF3	ENSG00000166526.12	0.219	0.101	2.174	0.031
ENSG00000103811.11_CTSH	CTSH	ENSG00000103811.11	4.442	2.044	2.173	0.031
ENSG00000172201.6_ID4	ID4	ENSG00000172201.6	2.591	1.193	2.172	0.031
ENSG00000184313.14_HEATR8	HEATR8	ENSG00000184313.14	0.624	0.287	2.172	0.031
ENSG00000146839.14_ZAN	ZAN	ENSG00000146839.14	0.000	0.000	2.172	0.031
ENSG00000214513.3_NOTO	NOTO	ENSG00000214513.3	0.002	0.001	2.172	0.031
ENSG00000196924.10_FLNA	FLNA	ENSG00000196924.10	3.972	1.830	2.171	0.031
ENSG00000205358.3_MT1H	MT1H	ENSG00000205358.3	3.626	1.671	2.170	0.031
ENSG00000261813.1_RP11-151H2.3	RP11-151H2.3	ENSG00000261813.1	0.051	0.024	2.170	0.031
ENSG00000227671.2_RP11-488L18.4	RP11-488L18.4	ENSG00000227671.2	0.214	0.099	2.166	0.032
ENSG00000185475.3_TMEM179B	TMEM179B	ENSG00000185475.3	0.910	0.420	2.166	0.032
ENSG00000177105.9_RHOG	RHOG	ENSG00000177105.9	1.675	0.773	2.166	0.032
ENSG00000125458.2_NT5C	NT5C	ENSG00000125458.2	1.189	0.549	2.166	0.032
ENSG00000107882.7_SUFU	SUFU	ENSG00000107882.7	0.171	0.079	2.165	0.032
ENSG00000162068.1_NTN3	NTN3	ENSG00000162068.1	0.034	0.016	2.165	0.032
ENSG00000146828.12_SLC12A9	SLC12A9	ENSG00000146828.12	0.729	0.337	2.164	0.032
ENSG00000231925.7_TAPBP	TAPBP	ENSG00000231925.7	1.482	0.685	2.164	0.032
ENSG00000104728.11_ARHGEF10	ARHGEF10	ENSG00000104728.11	0.825	0.381	2.164	0.032
ENSG00000130811.6_EIF3G	EIF3G	ENSG00000130811.6	2.204	1.018	2.164	0.032
ENSG00000205632.2_WI2-81516E3.1	WI2-81516E3.1	ENSG00000205632.2	0.042	0.020	2.163	0.032
ENSG00000186788.8_FAM75D3	FAM75D3	ENSG00000186788.8	0.002	0.001	2.163	0.032
ENSG00000259318.1_RP11-454L9.2	RP11-454L9.2	ENSG00000259318.1	0.108	0.050	2.163	0.032
ENSG00000108679.7_LGALS3BP	LGALS3BP	ENSG00000108679.7	1.982	0.917	2.163	0.032

ENSG00000130052.9_STARD8	STARD8	ENSG00000130052.9	0.298	0.138	2.163	0.032
ENSG00000135919.8_SERPINE2	SERPINE2	ENSG00000135919.8	6.056	2.801	2.162	0.032
ENSG00000110046.8_ATG2A	ATG2A	ENSG00000110046.8	0.359	0.166	2.162	0.032
ENSG00000260439.1_LA16c-366D3.1	LA16c-366D3.1	ENSG00000260439.1	0.050	0.023	2.161	0.032
ENSG00000225357.2_RPF2P1	RPF2P1	ENSG00000225357.2	0.003	0.002	2.159	0.032
ENSG00000068784.8_SRBD1	SRBD1	ENSG00000068784.8	0.061	0.028	2.159	0.032
ENSG00000214856.6_KRT16P1	KRT16P1	ENSG00000214856.6	0.015	0.007	2.159	0.032
ENSG00000226026.1_RP11-57H12.3	RP11-57H12.3	ENSG00000226026.1	0.066	0.030	2.158	0.032
ENSG00000188603.12_CLN3	CLN3	ENSG00000188603.12	0.428	0.199	2.158	0.032
ENSG00000100889.7_PCK2	PCK2	ENSG00000100889.7	0.227	0.105	2.157	0.032
ENSG00000142910.10_TINAGL1	TINAGL1	ENSG00000142910.10	1.620	0.751	2.156	0.032
ENSG00000172354.5_GNB2	GNB2	ENSG00000172354.5	4.439	2.059	2.156	0.032
ENSG00000229572.1_EIF4BP4	EIF4BP4	ENSG00000229572.1	0.001	0.000	2.155	0.033
ENSG00000174989.7_FBXW8	FBXW8	ENSG00000174989.7	0.118	0.055	2.155	0.033
ENSG00000226977.1_HMGN1P24	HMGN1P24	ENSG00000226977.1	0.085	0.039	2.155	0.033
ENSG00000171444.12_MCC	MCC	ENSG00000171444.12	0.260	0.121	2.155	0.033
ENSG00000118707.4_TGIF2	TGIF2	ENSG00000118707.4	0.102	0.048	2.153	0.033
ENSG00000018408.10_WWTR1	WWTR1	ENSG00000018408.10	0.496	0.230	2.153	0.033
ENSG00000172123.8_SLFN12	SLFN12	ENSG00000172123.8	0.066	0.031	2.151	0.033
ENSG00000101017.9_CD40	CD40	ENSG00000101017.9	0.246	0.115	2.151	0.033
ENSG00000151650.6_VENTX	VENTX	ENSG00000151650.6	0.084	0.039	2.151	0.033
ENSG00000120063.5_GNA13	GNA13	ENSG00000120063.5	0.535	0.249	2.151	0.033
ENSG00000139514.8_SLC7A1	SLC7A1	ENSG00000139514.8	0.743	0.345	2.150	0.033
ENSG00000158321.11_AUTS2	AUTS2	ENSG00000158321.11	0.443	0.206	2.149	0.033
ENSG00000164626.8_KCNK5	KCNK5	ENSG00000164626.8	0.009	0.004	2.149	0.033
ENSG00000237803.1_LINC00211	LINC00211	ENSG00000237803.1	0.003	0.001	2.149	0.033
ENSG00000255484.1_RP11-65M17.1	RP11-65M17.1	ENSG00000255484.1	0.002	0.001	2.148	0.033
ENSG00000187762.4_RP11-527B17.2	RP11-527B17.2	ENSG00000187762.4	0.056	0.026	2.147	0.033
ENSG00000104447.6_TRPS1	TRPS1	ENSG00000104447.6	0.268	0.125	2.147	0.033
ENSG00000177728.10_KIAA0195	KIAA0195	ENSG00000177728.10	3.348	1.560	2.147	0.033
ENSG00000110921.7_MVK	MVK	ENSG00000110921.7	0.549	0.256	2.146	0.033
ENSG00000005206.10_SPPL2B	SPPL2B	ENSG00000005206.10	1.619	0.754	2.146	0.033
ENSG00000213699.4_C2orf18	C2orf18	ENSG00000213699.4	0.376	0.175	2.146	0.033
ENSG00000163406.6_SLC15A2	SLC15A2	ENSG00000163406.6	0.459	0.214	2.146	0.033
ENSG00000079335.13_CDC14A	CDC14A	ENSG00000079335.13	0.130	0.060	2.146	0.033
ENSG00000123338.8_NCKAP1L	NCKAP1L	ENSG00000123338.8	0.170	0.079	2.145	0.033
ENSG00000172936.8_MYD88	MYD88	ENSG00000172936.8	0.340	0.159	2.145	0.033
ENSG00000182179.6_UBA7	UBA7	ENSG00000182179.6	0.793	0.370	2.145	0.033
ENSG00000115604.6_IL18R1	IL18R1	ENSG00000115604.6	0.148	0.069	2.144	0.033
ENSG00000148482.7_SLC39A12	SLC39A12	ENSG00000148482.7	1.541	0.719	2.144	0.033
ENSG00000186283.9_TOR3A	TOR3A	ENSG00000186283.9	0.274	0.128	2.143	0.034
ENSG00000240712.2_RP11-244K5.6	RP11-244K5.6	ENSG00000240712.2	0.028	0.013	2.142	0.034
ENSG00000225463.1_ZNF70P1	ZNF70P1	ENSG00000225463.1	0.000	0.000	2.141	0.034
ENSG00000165458.8_INPPL1	INPPL1	ENSG00000165458.8	1.678	0.784	2.141	0.034
ENSG00000170549.3_IRX1	IRX1	ENSG00000170549.3	0.009	0.004	2.141	0.034
ENSG00000244265.1_SIAH2-AS1	SIAH2-AS1	ENSG00000244265.1	0.010	0.005	2.141	0.034
ENSG00000248795.1_RP11-173E2.1	RP11-173E2.1	ENSG00000248795.1	0.086	0.040	2.140	0.034
ENSG00000186049.4_KRT73	KRT73	ENSG00000186049.4	0.047	0.022	2.140	0.034
ENSG00000226871.1_AC135178.7	AC135178.7	ENSG00000226871.1	0.001	0.000	2.140	0.034
ENSG00000132855.4_ANGPTL3	ANGPTL3	ENSG00000132855.4	0.000	0.000	2.140	0.034
ENSG00000258571.1_PTTG4P	PTTG4P	ENSG00000258571.1	0.000	0.000	2.140	0.034
ENSG00000173762.3_CD7	CD7	ENSG00000173762.3	0.097	0.046	2.140	0.034
ENSG00000174600.9_CMKLR1	CMKLR1	ENSG00000174600.9	0.071	0.033	2.140	0.034
ENSG00000226808.1_RP11-168P8.2	RP11-168P8.2	ENSG00000226808.1	0.020	0.009	2.139	0.034
ENSG00000243153.1_RP11-342M1.4	RP11-342M1.4	ENSG00000243153.1	0.028	0.013	2.139	0.034
ENSG00000162383.7_SLC1A7	SLC1A7	ENSG00000162383.7	0.858	0.401	2.138	0.034
ENSG00000214992.5_AKAP16BP	AKAP16BP	ENSG00000214992.5	0.009	0.004	2.138	0.034
ENSG00000233672.1_RNASEH2B-AS1	RNASEH2B-AS1	ENSG00000233672.1	0.022	0.010	2.137	0.034
ENSG00000144810.11_COL8A1	COL8A1	ENSG00000144810.11	0.065	0.031	2.137	0.034
ENSG00000229644.4_NAMPTL	NAMPTL	ENSG00000229644.4	0.069	0.032	2.137	0.034

ENSG00000117394.13_SLC2A1	SLC2A1	ENSG00000117394.13	6.450	3.019	2.136	0.034
ENSG00000234667.1_AC105935.1	AC105935.1	ENSG00000234667.1	0.008	0.004	2.136	0.034
ENSG00000105669.7_COPE	COPE	ENSG00000105669.7	4.471	2.093	2.136	0.034
ENSG00000248925.1_CTD-2083E4.6	CTD-2083E4.6	ENSG00000248925.1	0.094	0.044	2.135	0.034
ENSG00000167614.8_TTYH1	TTYH1	ENSG00000167614.8	19.768	9.262	2.134	0.034
ENSG00000152939.10_MARVELD2	MARVELD2	ENSG00000152939.10	0.051	0.024	2.134	0.034
ENSG00000127249.10_ATP13A4	ATP13A4	ENSG00000127249.10	0.921	0.432	2.134	0.034
ENSG00000071127.12_WDR1	WDR1	ENSG00000071127.12	1.537	0.720	2.133	0.034
ENSG00000170743.11_SYT9	SYT9	ENSG00000170743.11	0.130	0.061	2.133	0.034
ENSG00000213443.2_NPM1P5	NPM1P5	ENSG00000213443.2	0.107	0.050	2.133	0.034
ENSG00000103740.5_ACSBG1	ACSBG1	ENSG00000103740.5	3.041	1.426	2.133	0.034
ENSG00000114349.5_GNAT1	GNAT1	ENSG00000114349.5	0.016	0.007	2.132	0.034
ENSG00000121940.11_CLCC1	CLCC1	ENSG00000121940.11	0.521	0.244	2.132	0.034
ENSG00000124541.5_RRP36	RRP36	ENSG00000124541.5	0.592	0.278	2.132	0.034
ENSG00000204356.6_RDBP	RDBP	ENSG00000204356.6	0.870	0.408	2.132	0.034
ENSG00000122121.6_XPNPEP2	XPNPEP2	ENSG00000122121.6	0.007	0.003	2.132	0.034
ENSG00000167264.13_DUS2L	DUS2L	ENSG00000167264.13	0.216	0.101	2.131	0.035
ENSG00000225855.2_RUSC1-AS1	RUSC1-AS1	ENSG00000225855.2	0.075	0.035	2.131	0.035
ENSG00000170469.6_SPATA24	SPATA24	ENSG00000170469.6	0.159	0.075	2.130	0.035
ENSG00000244509.2_APOBEC3C	APOBEC3C	ENSG00000244509.2	0.156	0.073	2.129	0.035
ENSG00000221978.7_CCNL2	CCNL2	ENSG00000221978.7	2.222	1.044	2.129	0.035
ENSG00000137076.12_TLN1	TLN1	ENSG00000137076.12	1.436	0.675	2.129	0.035
ENSG00000130749.4_ZC3H4	ZC3H4	ENSG00000130749.4	0.214	0.101	2.129	0.035
ENSG00000160446.13_ZDHHC12	ZDHHC12	ENSG00000160446.13	0.356	0.167	2.129	0.035
ENSG00000099797.6_TECR	TECR	ENSG00000099797.6	6.075	2.855	2.128	0.035
ENSG00000253830.1_AC005895.3	AC005895.3	ENSG00000253830.1	0.093	0.044	2.128	0.035
ENSG00000267650.1_CTD-2553C6.1	CTD-2553C6.1	ENSG00000267650.1	0.024	0.011	2.128	0.035
ENSG00000213689.5_TREX1	TREX1	ENSG00000213689.5	1.424	0.669	2.128	0.035
ENSG00000161132.6_XXbac-B444P24.10	XXbac-B444P24.10	ENSG00000161132.6	0.079	0.037	2.128	0.035
ENSG00000257817.1_RP4-601P9.2	RP4-601P9.2	ENSG00000257817.1	0.002	0.001	2.125	0.035
ENSG00000138772.8_ANXA3	ANXA3	ENSG00000138772.8	1.075	0.506	2.125	0.035
ENSG00000182809.6_CRIP2	CRIP2	ENSG00000182809.6	7.616	3.588	2.123	0.035
ENSG00000213853.5_EMP2	EMP2	ENSG00000213853.5	1.005	0.474	2.121	0.035
ENSG00000143178.8_TBX19	TBX19	ENSG00000143178.8	0.063	0.030	2.121	0.035
ENSG00000107099.10_DOCK8	DOCK8	ENSG00000107099.10	0.082	0.039	2.120	0.035
ENSG00000231290.1_APCDD1L-AS1	APCDD1L-AS1	ENSG00000231290.1	0.015	0.007	2.119	0.036
ENSG00000174374.9_WBSCR16	WBSCR16	ENSG00000174374.9	0.600	0.283	2.119	0.036
ENSG00000253888.1_RP11-521M14.1	RP11-521M14.1	ENSG00000253888.1	0.000	0.000	2.118	0.036
ENSG00000169184.5_MN1	MN1	ENSG00000169184.5	0.087	0.041	2.118	0.036
ENSG00000168710.13_AHCYL1	AHCYL1	ENSG00000168710.13	11.996	5.664	2.118	0.036
ENSG00000244457.1_ENO1P1	ENO1P1	ENSG00000244457.1	0.009	0.004	2.117	0.036
ENSG00000136044.7_APPL2	APPL2	ENSG00000136044.7	0.804	0.380	2.117	0.036
ENSG00000237102.2_AC040160.1	AC040160.1	ENSG00000237102.2	0.044	0.021	2.117	0.036
ENSG00000259595.1_RP11-516C1.1	RP11-516C1.1	ENSG00000259595.1	0.002	0.001	2.117	0.036
ENSG00000255542.1_RP4-683L5.1	RP4-683L5.1	ENSG00000255542.1	0.050	0.024	2.115	0.036
ENSG00000164776.5_PHKG1	PHKG1	ENSG00000164776.5	1.030	0.487	2.115	0.036
ENSG00000213366.7_GSTM2	GSTM2	ENSG00000213366.7	5.657	2.675	2.115	0.036
ENSG00000105355.4_PLIN3	PLIN3	ENSG00000105355.4	0.902	0.427	2.115	0.036
ENSG00000239791.1_AC002310.7	AC002310.7	ENSG00000239791.1	0.016	0.008	2.114	0.036
ENSG00000176024.11_ZNF613	ZNF613	ENSG00000176024.11	0.059	0.028	2.114	0.036
ENSG00000010610.5_CD4	CD4	ENSG00000010610.5	0.439	0.208	2.114	0.036
ENSG00000110719.5_TCIRG1	TCIRG1	ENSG00000110719.5	1.160	0.549	2.113	0.036
ENSG00000154856.7_APCDD1	APCDD1	ENSG00000154856.7	1.847	0.874	2.113	0.036
ENSG00000113645.8_WWC1	WWC1	ENSG00000113645.8	1.376	0.651	2.112	0.036
ENSG00000111450.9_STX2	STX2	ENSG00000111450.9	0.392	0.186	2.112	0.036
ENSG00000205336.6_GPR56	GPR56	ENSG00000205336.6	6.387	3.024	2.112	0.036
ENSG00000250563.1_RP11-622O11.1	RP11-622O11.1	ENSG00000250563.1	0.007	0.003	2.112	0.036
ENSG00000046889.14_PREX2	PREX2	ENSG00000046889.14	0.299	0.142	2.111	0.036
ENSG00000181481.9_RNF135	RNF135	ENSG00000181481.9	0.236	0.112	2.111	0.036
ENSG00000266744.1_RP11-131K5.1	RP11-131K5.1	ENSG00000266744.1	0.021	0.010	2.110	0.036

ENSG00000232490.2_OSBPL10-AS1	OSBPL10-AS1	ENSG00000232490.2	0.752	0.357	2.110	0.036
ENSG00000141756.13_FKBP10	FKBP10	ENSG00000141756.13	1.152	0.546	2.110	0.036
ENSG00000205500.4_AC013472.3	AC013472.3	ENSG00000205500.4	0.011	0.005	2.109	0.036
ENSG00000237807.3_RP11-400K9.4	RP11-400K9.4	ENSG00000237807.3	0.063	0.030	2.109	0.036
ENSG00000155324.5_GRAMD3	GRAMD3	ENSG00000155324.5	2.424	1.150	2.108	0.037
ENSG00000266925.1_RP11-434D2.7	RP11-434D2.7	ENSG00000266925.1	0.001	0.000	2.107	0.037
ENSG00000187838.12_PLSCR3	PLSCR3	ENSG00000187838.12	0.345	0.164	2.107	0.037
ENSG00000237162.1_RP11-443F16.1	RP11-443F16.1	ENSG00000237162.1	0.045	0.021	2.107	0.037
ENSG00000132703.3_APCS	APCS	ENSG00000132703.3	0.030	0.014	2.105	0.037
ENSG00000260540.1_FAM108A9P	FAM108A9P	ENSG00000260540.1	0.000	0.000	2.105	0.037
ENSG00000239556.1_AC004951.5	AC004951.5	ENSG00000239556.1	0.020	0.009	2.105	0.037
ENSG00000158816.10_VWA5B1	VWA5B1	ENSG00000158816.10	0.013	0.006	2.103	0.037
ENSG00000189430.7_NCR1	NCR1	ENSG00000189430.7	0.013	0.006	2.103	0.037
ENSG00000197747.4_S100A10	S100A10	ENSG00000197747.4	2.271	1.080	2.103	0.037
ENSG00000228214.1_AC109586.1	AC109586.1	ENSG00000228214.1	0.129	0.061	2.102	0.037
ENSG00000103335.14_PIEZO1	PIEZO1	ENSG00000103335.14	2.253	1.072	2.102	0.037
ENSG00000245080.3_RP11-320N21.1	RP11-320N21.1	ENSG00000245080.3	0.002	0.001	2.102	0.037
ENSG00000263316.1_RP11-530N7.3	RP11-530N7.3	ENSG00000263316.1	0.018	0.008	2.102	0.037
ENSG00000242507.2_RP3-477O4.5	RP3-477O4.5	ENSG00000242507.2	0.000	0.000	2.101	0.037
ENSG00000102174.7_PHEX	PHEX	ENSG00000102174.7	0.024	0.011	2.101	0.037
ENSG00000117868.10_ESYT2	ESYT2	ENSG00000117868.10	0.560	0.267	2.100	0.037
ENSG00000212855.5_TTTY2	TTTY2	ENSG00000212855.5	0.002	0.001	2.100	0.037
ENSG00000212856.5_TTTY2B	TTTY2B	ENSG00000212856.5	0.002	0.001	2.100	0.037
ENSG00000253965.1_CTC-329D1.3	CTC-329D1.3	ENSG00000253965.1	0.036	0.017	2.099	0.037
ENSG00000184640.12_41891	41891	ENSG00000184640.12	2.633	1.256	2.097	0.037
ENSG00000090924.8_PLEKHG2	PLEKHG2	ENSG00000090924.8	0.259	0.123	2.097	0.037
ENSG00000197070.9_ARRDC1	ARRDC1	ENSG00000197070.9	0.910	0.434	2.097	0.038
ENSG00000103549.16_RNF40	RNF40	ENSG00000103549.16	0.832	0.397	2.095	0.038
ENSG00000198312.4_RP11-381O7.1	RP11-381O7.1	ENSG00000198312.4	0.049	0.024	2.094	0.038
ENSG00000156535.8_CD109	CD109	ENSG00000156535.8	0.057	0.027	2.093	0.038
ENSG00000204351.6_SKIV2L	SKIV2L	ENSG00000204351.6	1.221	0.583	2.093	0.038
ENSG00000138639.13_ARHGAP24	ARHGAP24	ENSG00000138639.13	0.275	0.131	2.091	0.038
ENSG00000263400.1_CTC-297N7.5	CTC-297N7.5	ENSG00000263400.1	0.147	0.070	2.091	0.038
ENSG00000113396.8_SLC27A6	SLC27A6	ENSG00000113396.8	0.016	0.008	2.091	0.038
ENSG00000189149.7_CRYM-AS1	CRYM-AS1	ENSG00000189149.7	0.012	0.006	2.090	0.038
ENSG00000084093.11_REST	REST	ENSG00000084093.11	0.129	0.062	2.090	0.038
ENSG00000228293.1_RP4-686C3.7	RP4-686C3.7	ENSG00000228293.1	0.009	0.004	2.090	0.038
ENSG00000228992.2_RPL5P32	RPL5P32	ENSG00000228992.2	0.036	0.017	2.090	0.038
ENSG00000162882.10_HAAO	HAAO	ENSG00000162882.10	0.125	0.060	2.090	0.038
ENSG00000105519.7_CAPS	CAPS	ENSG00000105519.7	1.515	0.725	2.090	0.038
ENSG00000249119.1_MTND6P4	MTND6P4	ENSG00000249119.1	0.214	0.103	2.089	0.038
ENSG00000182492.10_BGN	BGN	ENSG00000182492.10	2.843	1.361	2.089	0.038
ENSG00000233221.2_AC133785.1	AC133785.1	ENSG00000233221.2	0.077	0.037	2.088	0.038
ENSG00000226872.1_AC002472.11	AC002472.11	ENSG00000226872.1	0.018	0.008	2.088	0.038
ENSG00000112782.10_CLIC5	CLIC5	ENSG00000112782.10	0.099	0.047	2.088	0.038
ENSG00000222515.1_7SK	RN7SKP240	ENSG00000222515.1	0.045	0.021	2.088	0.038
ENSG00000110721.6_CHKA	CHKA	ENSG00000110721.6	2.195	1.052	2.087	0.038
ENSG00000143819.8_EPHX1	EPHX1	ENSG00000143819.8	9.327	4.470	2.087	0.038
ENSG00000144655.10_CSRNP1	CSRNP1	ENSG00000144655.10	0.878	0.421	2.086	0.038
ENSG00000165801.5_ARHGEF40	ARHGEF40	ENSG00000165801.5	0.795	0.381	2.084	0.039
ENSG00000233954.4_RP11-169K16.7	RP11-169K16.7	ENSG00000233954.4	0.197	0.095	2.083	0.039
ENSG00000198960.6_ARMCX6	ARMCX6	ENSG00000198960.6	0.518	0.249	2.083	0.039
ENSG00000136205.12_TNS3	TNS3	ENSG00000136205.12	2.714	1.303	2.083	0.039
ENSG00000237861.1_RP11-332L8.1	RP11-332L8.1	ENSG00000237861.1	0.014	0.007	2.082	0.039
ENSG00000232628.1_RP11-365O16.3	RP11-365O16.3	ENSG00000232628.1	0.083	0.040	2.082	0.039
ENSG00000259396.1_RP11-16O9.2	RP11-16O9.2	ENSG00000259396.1	0.003	0.001	2.081	0.039
ENSG00000228043.1_AC097721.2	AC097721.2	ENSG00000228043.1	0.027	0.013	2.081	0.039
ENSG00000102970.6_CCL17	CCL17	ENSG00000102970.6	0.017	0.008	2.081	0.039
ENSG00000214738.2_ABHD11-AS2	ABHD11-AS2	ENSG00000214738.2	0.003	0.001	2.080	0.039
ENSG00000259753.1_RP11-290H9.2	RP11-290H9.2	ENSG00000259753.1	0.004	0.002	2.078	0.039

ENSG00000178033.5_FAM26E	FAM26E	ENSG00000178033.5	0.017	0.008	2.078	0.039
ENSG00000152284.4_TCF7L1	TCF7L1	ENSG00000152284.4	0.193	0.093	2.077	0.039
ENSG00000264458.1_RP11-220C2.1	RP11-220C2.1	ENSG00000264458.1	0.013	0.006	2.077	0.039
ENSG00000265506.1_AC004656.1	AC004656.1	ENSG00000265506.1	0.001	0.000	2.076	0.039
ENSG00000223436.1_AC011625.1	AC011625.1	ENSG00000223436.1	0.002	0.001	2.076	0.039
ENSG00000224345.1_AKIRIN2-AS1	AKIRIN2-AS1	ENSG00000224345.1	0.000	0.000	2.076	0.039
ENSG00000267110.1_CTD-2587H24.4	CTD-2587H24.4	ENSG00000267110.1	0.000	0.000	2.076	0.039
ENSG00000266117.1_LOC732538	LOC732538	ENSG00000266117.1	0.000	0.000	2.076	0.039
ENSG00000250741.2_NT5C1B-RDH14	NT5C1B-RDH14	ENSG00000250741.2	0.000	0.000	2.076	0.039
ENSG00000253434.1_RP11-1101K5.1	RP11-1101K5.1	ENSG00000253434.1	0.000	0.000	2.076	0.039
ENSG00000259086.1_RP11-134E15.2	RP11-134E15.2	ENSG00000259086.1	0.004	0.002	2.076	0.039
ENSG00000217379.2_RP11-254A17.1	RP11-254A17.1	ENSG00000217379.2	0.000	0.000	2.076	0.039
ENSG00000243038.1_RP11-58E21.1	RP11-58E21.1	ENSG00000243038.1	0.000	0.000	2.076	0.039
ENSG00000255125.1_RP11-685M7.5	RP11-685M7.5	ENSG00000255125.1	0.000	0.000	2.076	0.039
ENSG00000217314.2_UQCRFS1P3	UQCRFS1P3	ENSG00000217314.2	0.000	0.000	2.076	0.039
ENSG00000229836.1_XXbac-BPG248L24.1(	XXbac-BPG248L24.10	ENSG00000229836.1	0.001	0.000	2.076	0.039
ENSG00000170385.9_SLC30A1	SLC30A1	ENSG00000170385.9	0.443	0.213	2.075	0.039
ENSG00000243899.1_BMS1P7	BMS1P7	ENSG00000243899.1	0.061	0.029	2.075	0.039
ENSG00000133313.10_CNDP2	CNDP2	ENSG00000133313.10	1.809	0.872	2.075	0.040
ENSG00000252122.1_SNORA76	SNORA76	ENSG00000252122.1	0.002	0.001	2.074	0.040
ENSG00000114013.11_CD86	CD86	ENSG00000114013.11	0.108	0.052	2.074	0.040
ENSG00000112343.7_TRIM38	TRIM38	ENSG00000112343.7	0.104	0.050	2.074	0.040
ENSG00000166979.8_FAM176C	FAM176C	ENSG00000166979.8	0.659	0.318	2.073	0.040
ENSG00000136830.6_FAM129B	FAM129B	ENSG00000136830.6	1.307	0.631	2.073	0.040
ENSG00000249934.1_RP11-466G12.3	RP11-466G12.3	ENSG00000249934.1	0.016	0.008	2.073	0.040
ENSG00000012660.9_ELOVL5	ELOVL5	ENSG00000012660.9	1.817	0.877	2.072	0.040
ENSG00000227500.5_SCAMP4	SCAMP4	ENSG00000227500.5	0.936	0.452	2.072	0.040
ENSG00000248767.1_WI2-2373I1.2	WI2-2373I1.2	ENSG00000248767.1	0.002	0.001	2.071	0.040
ENSG00000179364.9_PACS2	PACS2	ENSG00000179364.9	2.439	1.178	2.071	0.040
ENSG00000166482.7_MFAP4	MFAP4	ENSG00000166482.7	0.137	0.066	2.071	0.040
ENSG00000197054.6_ZNF763	ZNF763	ENSG00000197054.6	0.092	0.044	2.070	0.040
ENSG00000257337.1_RP11-983P16.4	RP11-983P16.4	ENSG00000257337.1	0.732	0.354	2.069	0.040
ENSG00000124570.12_SERPINB6	SERPINB6	ENSG00000124570.12	2.564	1.240	2.069	0.040
ENSG00000143878.8_RHOB	RHOB	ENSG00000143878.8	9.614	4.647	2.069	0.040
ENSG00000258466.1_RP11-1012A1.4	RP11-1012A1.4	ENSG00000258466.1	0.025	0.012	2.068	0.040
ENSG00000234742.1_AC144530.1	AC144530.1	ENSG00000234742.1	0.047	0.023	2.068	0.040
ENSG00000197153.3_HIST1H3J	HIST1H3J	ENSG00000197153.3	0.018	0.009	2.068	0.040
ENSG00000261393.1_RP11-21B23.1	RP11-21B23.1	ENSG00000261393.1	0.014	0.007	2.068	0.040
ENSG00000072840.8_EVC	EVC	ENSG00000072840.8	0.071	0.034	2.067	0.040
ENSG00000007314.7_SCN4A	SCN4A	ENSG00000007314.7	0.011	0.005	2.067	0.040
ENSG00000168065.11_SLC22A11	SLC22A11	ENSG00000168065.11	0.012	0.006	2.067	0.040
ENSG00000108179.8_PPIF	PPIF	ENSG00000108179.8	0.940	0.455	2.066	0.040
ENSG00000228659.1_RP1-23K20.2	RP1-23K20.2	ENSG00000228659.1	0.007	0.003	2.066	0.040
ENSG00000092820.13_EZR	EZR	ENSG00000092820.13	4.654	2.253	2.066	0.040
ENSG00000223473.1_GS1-124K5.3	GS1-124K5.3	ENSG00000223473.1	0.055	0.027	2.066	0.040
ENSG00000005471.11_ABCB4	ABCB4	ENSG00000005471.11	0.045	0.022	2.065	0.040
ENSG00000216347.1_RP3-334F4.2	RP3-334F4.2	ENSG00000216347.1	0.000	0.000	2.065	0.040
ENSG00000100721.6_TCL1A	TCL1A	ENSG00000100721.6	0.019	0.009	2.064	0.041
ENSG00000118762.3_PKD2	PKD2	ENSG00000118762.3	0.265	0.128	2.064	0.041
ENSG00000196154.7_S100A4	S100A4	ENSG00000196154.7	1.389	0.673	2.063	0.041
ENSG00000225485.3_ARHGAP23	ARHGAP23	ENSG00000225485.3	1.334	0.647	2.061	0.041
ENSG00000232746.1_RP11-767C1.1	RP11-767C1.1	ENSG00000232746.1	0.008	0.004	2.061	0.041
ENSG00000231765.1_PPP1R11P2	PPP1R11P2	ENSG00000231765.1	0.029	0.014	2.060	0.041
ENSG00000110203.4_FOLR3	FOLR3	ENSG00000110203.4	0.058	0.028	2.059	0.041
ENSG00000238224.1_RP11-522M21.2	RP11-522M21.2	ENSG00000238224.1	0.010	0.005	2.059	0.041
ENSG00000189275.3_AL450307.1	AL450307.1	ENSG00000189275.3	0.420	0.204	2.059	0.041
ENSG00000258637.1_RP11-242P2.1	RP11-242P2.1	ENSG00000258637.1	0.000	0.000	2.059	0.041
ENSG00000168309.12_FAM107A	FAM107A	ENSG00000168309.12	50.641	24.607	2.058	0.041
ENSG00000244101.1_HMGN1P10	HMGN1P10	ENSG00000244101.1	0.001	0.001	2.058	0.041
ENSG00000260132.1_LA16c-312E8.2	LA16c-312E8.2	ENSG00000260132.1	0.037	0.018	2.058	0.041

ENSG00000237857.1_RP11-435O5.2	RP11-435O5.2	ENSG00000237857.1	0.016	0.008	2.058	0.041
ENSG00000183943.5_PRKX	PRKX	ENSG00000183943.5	0.396	0.193	2.058	0.041
ENSG00000133246.6_PRAM1	PRAM1	ENSG00000133246.6	0.236	0.115	2.057	0.041
ENSG00000150782.7_IL18	IL18	ENSG00000150782.7	0.319	0.155	2.057	0.041
ENSG00000164089.4_AGXT2L1	AGXT2L1	ENSG00000164089.4	8.110	3.943	2.057	0.041
ENSG00000240876.1_RP4-669L17.11	RP4-669L17.11	ENSG00000240876.1	0.155	0.076	2.056	0.041
ENSG00000254265.1_CTD-2336O2.2	CTD-2336O2.2	ENSG00000254265.1	0.016	0.008	2.056	0.041
ENSG00000250062.1_RP11-778J15.1	RP11-778J15.1	ENSG00000250062.1	0.001	0.000	2.056	0.041
ENSG00000165449.7_SLC16A9	SLC16A9	ENSG00000165449.7	0.312	0.152	2.056	0.041
ENSG00000253640.1_CTD-2281E23.1	CTD-2281E23.1	ENSG00000253640.1	0.000	0.000	2.056	0.041
ENSG00000049192.10_ADAMTS6	ADAMTS6	ENSG00000049192.10	0.034	0.017	2.056	0.041
ENSG00000131148.3_EMC8	EMC8	ENSG00000131148.3	0.240	0.117	2.054	0.041
ENSG00000248102.1_RP11-79P5.3-001	RP11-79P5.3-001	ENSG00000248102.1	0.014	0.007	2.054	0.042
ENSG00000131446.11_MGAT1	MGAT1	ENSG00000131446.11	1.470	0.716	2.054	0.042
ENSG00000154620.5_TMSB4Y	TMSB4Y	ENSG00000154620.5	0.093	0.045	2.053	0.042
ENSG00000163378.9_EOGT	EOGT	ENSG00000163378.9	0.191	0.093	2.053	0.042
ENSG00000223953.3_C1QTNF5	C1QTNF5	ENSG00000223953.3	0.852	0.415	2.053	0.042
ENSG00000111780.8_GATC	GATC	ENSG00000111780.8	0.017	0.008	2.053	0.042
ENSG00000130720.7_FIBCD1	FIBCD1	ENSG00000130720.7	0.059	0.029	2.051	0.042
ENSG00000259423.1_RP11-265N7.2	RP11-265N7.2	ENSG00000259423.1	0.000	0.000	2.051	0.042
ENSG00000131748.11_STARD3	STARD3	ENSG00000131748.11	1.659	0.809	2.050	0.042
ENSG00000132478.5_UNK	UNK	ENSG00000132478.5	0.237	0.115	2.050	0.042
ENSG00000237513.1_RP11-325F22.2	RP11-325F22.2	ENSG00000237513.1	0.011	0.005	2.050	0.042
ENSG00000084754.6_HADHA	HADHA	ENSG00000084754.6	1.838	0.897	2.050	0.042
ENSG00000110906.8_KCTD10	KCTD10	ENSG00000110906.8	0.320	0.156	2.050	0.042
ENSG00000120318.10_ARAP3	ARAP3	ENSG00000120318.10	0.344	0.168	2.049	0.042
ENSG00000265342.1_RP11-16C1.1	RP11-16C1.1	ENSG00000265342.1	0.855	0.417	2.048	0.042
ENSG00000248803.1_RP11-287J9.1	RP11-287J9.1	ENSG00000248803.1	0.020	0.010	2.048	0.042
ENSG00000111725.6_PRKAB1	PRKAB1	ENSG00000111725.6	0.402	0.196	2.048	0.042
ENSG00000223804.1_BX284650.1	BX284650.1	ENSG00000223804.1	0.144	0.070	2.048	0.042
ENSG00000023892.9_DEF6	DEF6	ENSG00000023892.9	0.245	0.120	2.047	0.042
ENSG00000105366.9_SIGLEC8	SIGLEC8	ENSG00000105366.9	0.214	0.105	2.046	0.042
ENSG00000186350.8_RXRA	RXRA	ENSG00000186350.8	1.431	0.699	2.046	0.042
ENSG00000267359.1_RP11-1094M14.12	RP11-1094M14.12	ENSG00000267359.1	0.000	0.000	2.046	0.042
ENSG00000233745.1_AC010997.1	AC010997.1	ENSG00000233745.1	0.014	0.007	2.044	0.042
ENSG00000159176.9_CSRP1	CSRP1	ENSG00000159176.9	23.530	11.513	2.044	0.043
ENSG00000256463.4_SALL3	SALL3	ENSG00000256463.4	0.397	0.194	2.044	0.043
ENSG00000214456.4_PLIN5	PLIN5	ENSG00000214456.4	1.400	0.685	2.043	0.043
ENSG00000234112.1_RP11-145A3.4	RP11-145A3.4	ENSG00000234112.1	0.039	0.019	2.043	0.043
ENSG00000205036.2_C16orf85	C16orf85	ENSG00000205036.2	0.038	0.018	2.043	0.043
ENSG00000140873.11_ADAMTS18	ADAMTS18	ENSG00000140873.11	0.057	0.028	2.042	0.043
ENSG00000243789.6_JMJD7	JMJD7	ENSG00000243789.6	0.647	0.317	2.041	0.043
ENSG00000169006.6_NTSR2	NTSR2	ENSG00000169006.6	2.907	1.425	2.041	0.043
ENSG00000169413.2_RNASE6	RNASE6	ENSG00000169413.2	0.364	0.179	2.040	0.043
ENSG00000153902.9_LGI4	LGI4	ENSG00000153902.9	6.063	2.972	2.040	0.043
ENSG00000145476.11_CYP4V2	CYP4V2	ENSG00000145476.11	0.634	0.311	2.040	0.043
ENSG00000263041.1_RP11-355F22.1	RP11-355F22.1	ENSG00000263041.1	0.010	0.005	2.040	0.043
ENSG00000266846.1_RP11-793A3.2	RP11-793A3.2	ENSG00000266846.1	0.000	0.000	2.039	0.043
ENSG00000160410.9_SHKBP1	SHKBP1	ENSG00000160410.9	0.348	0.171	2.039	0.043
ENSG00000255966.1_RP5-940J5.3	RP5-940J5.3	ENSG00000255966.1	0.049	0.024	2.039	0.043
ENSG00000183160.8_TMEM119	TMEM119	ENSG00000183160.8	0.378	0.186	2.038	0.043
ENSG00000206897.1_SNORA9	SNORA9	ENSG00000206897.1	0.805	0.395	2.038	0.043
ENSG00000111713.2_GYS2	GYS2	ENSG00000111713.2	0.010	0.005	2.038	0.043
ENSG00000254126.2_AC108868.4	AC108868.4	ENSG00000254126.2	0.122	0.060	2.037	0.043
ENSG00000140829.7_DHX38	DHX38	ENSG00000140829.7	0.720	0.354	2.036	0.043
ENSG00000130787.9_HIP1R	HIP1R	ENSG00000130787.9	4.441	2.181	2.036	0.043
ENSG00000129521.8_EGLN3	EGLN3	ENSG00000129521.8	1.145	0.562	2.035	0.043
ENSG00000255103.1_KIAA0754	KIAA0754	ENSG00000255103.1	0.053	0.026	2.035	0.043
ENSG00000228718.1_RP11-145H9.3	RP11-145H9.3	ENSG00000228718.1	0.003	0.002	2.035	0.043
ENSG00000267442.1_AC005391.3	AC005391.3	ENSG00000267442.1	0.025	0.012	2.035	0.043

ENSG00000120915.9_EPHX2	EPHX2	ENSG00000120915.9	0.542	0.267	2.034	0.043
ENSG00000105011.4_ASF1B	ASF1B	ENSG00000105011.4	0.023	0.011	2.034	0.044
ENSG00000116833.9_NR5A2	NR5A2	ENSG00000116833.9	0.007	0.003	2.033	0.044
ENSG00000066336.7_SPI1	SPI1	ENSG00000066336.7	1.003	0.493	2.033	0.044
ENSG00000201457.1_SNORA55	SNORA55	ENSG00000201457.1	0.001	0.000	2.033	0.044
ENSG00000115902.6_SLC1A4	SLC1A4	ENSG00000115902.6	1.102	0.542	2.033	0.044
ENSG00000231027.1_AC079325.6	AC079325.6	ENSG00000231027.1	0.019	0.009	2.032	0.044
ENSG00000116478.7_HDAC1	HDAC1	ENSG00000116478.7	0.745	0.367	2.032	0.044
ENSG00000166527.3_CLEC4D	CLEC4D	ENSG00000166527.3	0.011	0.006	2.032	0.044
ENSG00000130147.11_SH3BP4	SH3BP4	ENSG00000130147.11	0.381	0.188	2.032	0.044
ENSG00000092621.7_PHGDH	PHGDH	ENSG00000092621.7	4.467	2.199	2.032	0.044
ENSG00000236058.2_RP11-215A21.2	RP11-215A21.2	ENSG00000236058.2	0.022	0.011	2.031	0.044
ENSG00000267775.1_OR7E16P	OR7E16P	ENSG00000267775.1	0.005	0.003	2.031	0.044
ENSG00000139350.7_NEDD1	NEDD1	ENSG00000139350.7	0.068	0.034	2.030	0.044
ENSG00000172081.9_MOB3A	MOB3A	ENSG00000172081.9	0.684	0.337	2.030	0.044
ENSG00000233325.2_MIPEPP3	MIPEPP3	ENSG00000233325.2	0.014	0.007	2.029	0.044
ENSG00000184489.7_PTP4A3	PTP4A3	ENSG00000184489.7	1.840	0.907	2.029	0.044
ENSG00000163945.11_UVSSA	UVSSA	ENSG00000163945.11	0.315	0.155	2.029	0.044
ENSG00000255089.1_RP11-326C3.10	RP11-326C3.10	ENSG00000255089.1	0.069	0.034	2.029	0.044
ENSG00000206113.2_RP11-503N18.1	RP11-503N18.1	ENSG00000206113.2	0.022	0.011	2.028	0.044
ENSG00000088298.7_EDEM2	EDEM2	ENSG00000088298.7	0.180	0.089	2.028	0.044
ENSG00000157017.11_GHRL	GHRL	ENSG00000157017.11	0.131	0.064	2.027	0.044
ENSG00000181449.2_SOX2	SOX2	ENSG00000181449.2	4.588	2.264	2.027	0.044
ENSG00000166025.13_AMOTL1	AMOTL1	ENSG00000166025.13	0.119	0.059	2.026	0.044
ENSG00000078246.10_TULP3	TULP3	ENSG00000078246.10	0.461	0.227	2.025	0.044
ENSG00000116761.7_CTH	CTH	ENSG00000116761.7	0.180	0.089	2.024	0.044
ENSG00000028137.12_TNFRSF1B	TNFRSF1B	ENSG00000028137.12	0.672	0.332	2.023	0.045
ENSG00000168792.4_ABHD15	ABHD15	ENSG00000168792.4	0.130	0.064	2.023	0.045
ENSG00000125820.5_NKX2-2	NKX2-2	ENSG00000125820.5	0.305	0.151	2.023	0.045
ENSG00000205537.2_RP11-89H19.1	RP11-89H19.1	ENSG00000205537.2	0.000	0.000	2.023	0.045
ENSG00000248677.1_CTD-2044J15.1	CTD-2044J15.1	ENSG00000248677.1	0.014	0.007	2.022	0.045
ENSG00000137834.10_SMAD6	SMAD6	ENSG00000137834.10	0.694	0.343	2.022	0.045
ENSG00000100226.11_GTPBP1	GTPBP1	ENSG00000100226.11	0.482	0.238	2.022	0.045
ENSG00000266964.1_FXYD1	FXYD1	ENSG00000266964.1	19.120	9.460	2.021	0.045
ENSG00000261641.1_LA16c-390E6.5	LA16c-390E6.5	ENSG00000261641.1	0.003	0.001	2.021	0.045
ENSG00000214164.2_AC018696.5	AC018696.5	ENSG00000214164.2	0.111	0.055	2.021	0.045
ENSG00000176984.2_AP000679.2	AP000679.2	ENSG00000176984.2	0.026	0.013	2.020	0.045
ENSG00000116641.11_DOCK7	DOCK7	ENSG00000116641.11	0.409	0.203	2.019	0.045
ENSG00000233109.3_RP11-22C8.1	RP11-22C8.1	ENSG00000233109.3	0.003	0.002	2.019	0.045
ENSG00000144233.5_AMMECR1L	AMMECR1L	ENSG00000144233.5	0.091	0.045	2.019	0.045
ENSG00000081320.5_STK17B	STK17B	ENSG00000081320.5	0.188	0.093	2.018	0.045
ENSG00000164736.5_SOX17	SOX17	ENSG00000164736.5	0.150	0.074	2.018	0.045
ENSG00000109062.5_SLC9A3R1	SLC9A3R1	ENSG00000109062.5	3.655	1.811	2.018	0.045
ENSG00000233159.1_AC007390.4	AC007390.4	ENSG00000233159.1	0.078	0.039	2.017	0.045
ENSG00000184140.4_OR4F6	OR4F6	ENSG00000184140.4	0.004	0.002	2.016	0.045
ENSG00000162738.5_VANGL2	VANGL2	ENSG00000162738.5	0.168	0.083	2.016	0.045
ENSG00000123146.14_CD97	CD97	ENSG00000123146.14	0.205	0.102	2.016	0.045
ENSG00000121310.11_ECHDC2	ECHDC2	ENSG00000121310.11	3.622	1.797	2.016	0.045
ENSG00000224806.2_ARL5AP4	ARL5AP4	ENSG00000224806.2	0.002	0.001	2.016	0.045
ENSG00000229130.2_RP11-76P2.3	RP11-76P2.3	ENSG00000229130.2	0.014	0.007	2.016	0.045
ENSG00000011143.12_MKS1	MKS1	ENSG00000011143.12	0.214	0.106	2.015	0.045
ENSG00000097007.12_ABL1	ABL1	ENSG00000097007.12	0.514	0.255	2.015	0.045
ENSG00000189221.5_MAOA	MAOA	ENSG00000189221.5	0.755	0.375	2.014	0.046
ENSG00000090621.8_PABPC4	PABPC4	ENSG00000090621.8	0.984	0.489	2.014	0.046
ENSG00000152620.8_NADKD1	NADKD1	ENSG00000152620.8	0.767	0.381	2.014	0.046
ENSG00000185753.8_CXorf38	CXorf38	ENSG00000185753.8	0.133	0.066	2.013	0.046
ENSG00000137693.9_YAP1	YAP1	ENSG00000137693.9	0.594	0.295	2.013	0.046
ENSG00000204176.8_SYT15	SYT15	ENSG00000204176.8	0.264	0.131	2.013	0.046
ENSG00000132669.8_RIN2	RIN2	ENSG00000132669.8	0.560	0.278	2.013	0.046
ENSG00000198663.10_C6orf89	C6orf89	ENSG00000198663.10	0.286	0.142	2.012	0.046

ENSG00000163064.5_EN1	EN1	ENSG00000163064.5	0.007	0.003	2.012	0.046
ENSG00000227081.4_RP11-543P15.1	RP11-543P15.1	ENSG00000227081.4	0.592	0.295	2.010	0.046
ENSG00000177197.7_PCNPP5	PCNPP5	ENSG00000177197.7	0.004	0.002	2.008	0.046
ENSG00000157193.10_LRP8	LRP8	ENSG00000157193.10	0.366	0.182	2.008	0.046
ENSG00000251567.1_RP11-775H9.2	RP11-775H9.2	ENSG00000251567.1	0.002	0.001	2.008	0.046
ENSG00000244716.2_RP11-20O24.4	RP11-20O24.4	ENSG00000244716.2	0.023	0.011	2.008	0.046
ENSG00000243711.1_RPL21P116	RPL21P116	ENSG00000243711.1	0.028	0.014	2.007	0.046
ENSG00000185112.4_FAM43A	FAM43A	ENSG00000185112.4	0.353	0.176	2.007	0.046
ENSG00000230082.1_PRRT3-AS1	PRRT3-AS1	ENSG00000230082.1	0.086	0.043	2.007	0.046
ENSG00000234597.1_AC010096.1	AC010096.1	ENSG00000234597.1	0.007	0.004	2.006	0.046
ENSG00000139899.6_CBLN3	CBLN3	ENSG00000139899.6	0.158	0.079	2.006	0.046
ENSG00000124201.10_ZNFX1	ZNFX1	ENSG00000124201.10	0.212	0.106	2.005	0.047
ENSG00000223569.1_RP11-1286E23.10	RP11-1286E23.10	ENSG00000223569.1	0.008	0.004	2.005	0.047
ENSG00000234775.1_RP11-335O13.7	RP11-335O13.7	ENSG00000234775.1	0.035	0.017	2.004	0.047
ENSG00000251452.1_RP13-539F13.2	RP13-539F13.2	ENSG00000251452.1	0.035	0.017	2.004	0.047
ENSG00000149289.6_ZC3H12C	ZC3H12C	ENSG00000149289.6	0.071	0.035	2.004	0.047
ENSG00000185043.6_CIB1	CIB1	ENSG00000185043.6	1.845	0.921	2.004	0.047
ENSG00000152213.3_ARL11	ARL11	ENSG00000152213.3	0.022	0.011	2.003	0.047
ENSG00000134755.10_DSC2	DSC2	ENSG00000134755.10	0.060	0.030	2.002	0.047
ENSG00000255661.2_RP11-59H1.3	RP11-59H1.3	ENSG00000255661.2	0.050	0.025	2.001	0.047
ENSG00000236542.1_RP4-694B14.4	RP4-694B14.4	ENSG00000236542.1	0.044	0.022	2.001	0.047
ENSG00000125843.6_AP5S1	AP5S1	ENSG00000125843.6	0.349	0.174	2.000	0.047
ENSG00000160703.10_NLRX1	NLRX1	ENSG00000160703.10	0.228	0.114	2.000	0.047
ENSG00000101310.10_SEC23B	SEC23B	ENSG00000101310.10	0.373	0.187	2.000	0.047
ENSG00000069248.8_NUP133	NUP133	ENSG00000069248.8	0.309	0.155	2.000	0.047
ENSG00000244097.1_RPS4XP17	RPS4XP17	ENSG00000244097.1	0.022	0.011	1.999	0.047
ENSG00000246731.2_CTD-2514K5.2	CTD-2514K5.2	ENSG00000246731.2	0.327	0.163	1.999	0.047
ENSG00000064490.7_RFXANK	RFXANK	ENSG00000064490.7	1.695	0.848	1.999	0.047
ENSG00000249428.1_RP11-503N18.3	RP11-503N18.3	ENSG00000249428.1	0.017	0.009	1.999	0.047
ENSG00000182938.4_OTOP3	OTOP3	ENSG00000182938.4	0.002	0.001	1.998	0.047
ENSG00000099994.10_SUSD2	SUSD2	ENSG00000099994.10	0.088	0.044	1.998	0.047
ENSG00000205267.3_AC004876.1	AC004876.1	ENSG00000205267.3	0.043	0.022	1.997	0.047
ENSG00000072786.7_STK10	STK10	ENSG00000072786.7	0.258	0.129	1.997	0.047
ENSG00000064205.6_WISP2	WISP2	ENSG00000064205.6	0.115	0.057	1.997	0.047
ENSG00000259561.1_RP11-300G22.2	RP11-300G22.2	ENSG00000259561.1	0.019	0.010	1.997	0.047
ENSG00000181264.4_TMEM136	TMEM136	ENSG00000181264.4	0.356	0.178	1.997	0.047
ENSG00000224877.2_C17orf89	C17orf89	ENSG00000224877.2	3.852	1.930	1.996	0.048
ENSG00000182446.8_NPLOC4	NPLOC4	ENSG00000182446.8	0.480	0.241	1.995	0.048
ENSG00000181192.7_DHTKD1	DHTKD1	ENSG00000181192.7	0.424	0.212	1.995	0.048
ENSG00000252992.1_SCARNA11	SCARNA11	ENSG00000252992.1	0.038	0.019	1.995	0.048
ENSG00000188740.6_RP11-483I13.4	RP11-483I13.4	ENSG00000188740.6	0.032	0.016	1.995	0.048
ENSG00000187954.8_CYHR1	CYHR1	ENSG00000187954.8	1.866	0.936	1.994	0.048
ENSG00000228067.1_RP11-61J19.3	RP11-61J19.3	ENSG00000228067.1	0.064	0.032	1.993	0.048
ENSG00000106571.8_GLI3	GLI3	ENSG00000106571.8	0.118	0.059	1.993	0.048
ENSG00000261103.1_RP11-298D21.3	RP11-298D21.3	ENSG00000261103.1	0.018	0.009	1.992	0.048
ENSG00000229996.1_AC093585.6	AC093585.6	ENSG00000229996.1	0.030	0.015	1.992	0.048
ENSG00000132688.10_NES	NES	ENSG00000132688.10	0.975	0.489	1.992	0.048
ENSG00000255829.1_RP11-20D14.3	RP11-20D14.3	ENSG00000255829.1	0.022	0.011	1.991	0.048
ENSG00000120278.10_PLEKHG1	PLEKHG1	ENSG00000120278.10	0.169	0.085	1.991	0.048
ENSG00000203843.3_PFN1P2	PFN1P2	ENSG00000203843.3	0.076	0.038	1.991	0.048
ENSG00000236370.1_RP11-574K11.16	RP11-574K11.16	ENSG00000236370.1	0.111	0.056	1.991	0.048
ENSG00000225473.1_ATP13A4-AS1	ATP13A4-AS1	ENSG00000225473.1	0.048	0.024	1.991	0.048
ENSG00000175283.7_DOLK	DOLK	ENSG00000175283.7	0.190	0.096	1.991	0.048
ENSG00000196663.11_TECPR2	TECPR2	ENSG00000196663.11	0.258	0.130	1.990	0.048
ENSG00000243113.1_RP11-480I12.9	RP11-480I12.9	ENSG00000243113.1	0.034	0.017	1.989	0.048
ENSG00000260394.2_LA16c-313D11.9	LA16c-313D11.9	ENSG00000260394.2	0.015	0.008	1.989	0.048
ENSG00000267723.1_CTD-2189E23.1	CTD-2189E23.1	ENSG00000267723.1	0.024	0.012	1.989	0.048
ENSG00000111859.12_NEDD9	NEDD9	ENSG00000111859.12	0.358	0.180	1.989	0.048
ENSG00000261764.1_KRT18P18	KRT18P18	ENSG00000261764.1	0.010	0.005	1.988	0.048
ENSG00000239282.3_GATSL3	GATSL3	ENSG00000239282.3	1.016	0.511	1.988	0.048

ENSG00000162889.6_MAPKAPK2	MAPKAPK2	ENSG00000162889.6	1.211	0.609	1.988	0.048
ENSG00000110066.10_SUV420H1	SUV420H1	ENSG00000110066.10	0.315	0.158	1.988	0.048
ENSG00000227695.1_DNMBP-AS1	DNMBP-AS1	ENSG00000227695.1	0.038	0.019	1.988	0.048
ENSG00000185862.5_EVI2B	EVI2B	ENSG00000185862.5	0.133	0.067	1.988	0.048
ENSG00000196209.7_SIRPB2	SIRPB2	ENSG00000196209.7	0.101	0.051	1.988	0.048
ENSG00000156639.7_ZFAND3	ZFAND3	ENSG00000156639.7	1.133	0.570	1.988	0.048
ENSG00000134871.13_COL4A2	COL4A2	ENSG00000134871.13	0.427	0.215	1.987	0.049
ENSG00000256218.1_RP11-1038A11.2	RP11-1038A11.2	ENSG00000256218.1	0.005	0.002	1.986	0.049
ENSG00000241280.1_RP11-221J22.2	RP11-221J22.2	ENSG00000241280.1	0.007	0.004	1.986	0.049
ENSG00000104881.10_PPP1R13L	PPP1R13L	ENSG00000104881.10	0.386	0.194	1.986	0.049
ENSG00000129219.8_PLD2	PLD2	ENSG00000129219.8	1.010	0.509	1.985	0.049
ENSG00000239249.2_MetazoaSRP	RN7SL757P	ENSG00000239249.2	0.151	0.076	1.985	0.049
ENSG00000203710.6_CR1	CR1	ENSG00000203710.6	0.023	0.012	1.985	0.049
ENSG00000159905.10_ZNF221	ZNF221	ENSG00000159905.10	0.094	0.047	1.985	0.049
ENSG00000167094.11_TTC16	TTC16	ENSG00000167094.11	0.039	0.020	1.984	0.049
ENSG00000264151.1_RP11-739N10.1	RP11-739N10.1	ENSG00000264151.1	0.014	0.007	1.984	0.049
ENSG00000168913.6_ENHO	ENHO	ENSG00000168913.6	19.389	9.778	1.983	0.049
ENSG00000158195.6_WASF2	WASF2	ENSG00000158195.6	1.412	0.712	1.983	0.049
ENSG00000123096.7_SSPN	SSPN	ENSG00000123096.7	0.296	0.149	1.983	0.049
ENSG00000251369.2_AC003682.1	AC003682.1	ENSG00000251369.2	0.013	0.006	1.983	0.049
ENSG00000173992.4_CCS	CCS	ENSG00000173992.4	1.371	0.692	1.982	0.049
ENSG00000111077.11_TENC1	TENC1	ENSG00000111077.11	2.994	1.511	1.982	0.049
ENSG00000175287.14_PHYHD1	PHYHD1	ENSG00000175287.14	3.602	1.817	1.982	0.049
ENSG00000204802.3_RP11-111F5.4	RP11-111F5.4	ENSG00000204802.3	0.146	0.074	1.982	0.049
ENSG00000117305.9_HMGCL	HMGCL	ENSG00000117305.9	0.760	0.384	1.982	0.049
ENSG00000230550.1_RP11-74C13.3	RP11-74C13.3	ENSG00000230550.1	0.003	0.001	1.982	0.049
ENSG00000196498.9_NCOR2	NCOR2	ENSG00000196498.9	1.423	0.718	1.981	0.049
ENSG00000165915.9_SLC39A13	SLC39A13	ENSG00000165915.9	1.051	0.531	1.981	0.049
ENSG00000259720.1_RP11-348B17.1	RP11-348B17.1	ENSG00000259720.1	0.001	0.000	1.981	0.049
ENSG00000196196.2_HRCT1	HRCT1	ENSG00000196196.2	0.431	0.217	1.980	0.049
ENSG00000180747.11_SLC7A5P1	SLC7A5P1	ENSG00000180747.11	0.679	0.343	1.980	0.049
ENSG00000185633.6_NDUFA4L2	NDUFA4L2	ENSG00000185633.6	1.937	0.979	1.980	0.049
ENSG00000229124.1_RP11-124N14.4	RP11-124N14.4	ENSG00000229124.1	0.034	0.017	1.980	0.049
ENSG00000150630.2_VEGFC	VEGFC	ENSG00000150630.2	0.075	0.038	1.980	0.049
ENSG00000171345.9_KRT19	KRT19	ENSG00000171345.9	0.139	0.070	1.979	0.049
ENSG00000068976.9_PYGM	PYGM	ENSG00000068976.9	1.237	0.625	1.979	0.049
ENSG00000135506.11_OS9	OS9	ENSG00000135506.11	1.490	0.753	1.978	0.050
ENSG00000232093.1_RP11-307C12.11	RP11-307C12.11	ENSG00000232093.1	0.078	0.040	1.978	0.050
ENSG00000225194.2_LINC00092	LINC00092	ENSG00000225194.2	0.841	0.425	1.977	0.050
ENSG00000233237.1_LINC00472	LINC00472	ENSG00000233237.1	0.065	0.033	1.977	0.050
ENSG00000214274.5_ANG	ANG	ENSG00000214274.5	0.139	0.070	1.977	0.050
ENSG00000144455.9_SUMF1	SUMF1	ENSG00000144455.9	0.194	0.098	1.977	0.050
ENSG00000242858.1_CTC-484M2.1	CTC-484M2.1	ENSG00000242858.1	0.021	0.011	1.977	0.050
ENSG00000227508.1_RP5-894D12.3	RP5-894D12.3	ENSG00000227508.1	0.001	0.000	1.977	0.050
ENSG00000157353.12_FUK	FUK	ENSG00000157353.12	0.380	0.192	1.976	0.050
ENSG00000257564.1_RP11-123M21.2	RP11-123M21.2	ENSG00000257564.1	0.011	0.006	1.976	0.050
ENSG00000128602.5_SMO	SMO	ENSG00000128602.5	0.360	0.182	1.974	0.050
ENSG00000175898.4_S1PR2	S1PR2	ENSG00000175898.4	0.106	0.054	1.974	0.050
ENSG00000172264.12_MACROD2	MACROD2	ENSG00000172264.12	-0.385	0.195	-1.974	0.050
ENSG00000237754.1_RP11-521C10.1	RP11-521C10.1	ENSG00000237754.1	0.000	0.000	-1.974	0.050
ENSG00000259569.1_RP11-97O12.3	RP11-97O12.3	ENSG00000259569.1	-0.052	0.026	-1.975	0.050
ENSG00000259957.2_RP11-491F9.8	RP11-491F9.8	ENSG00000259957.2	-0.005	0.002	-1.975	0.050
ENSG00000220412.1_RP11-95M15.2	RP11-95M15.2	ENSG00000220412.1	0.000	0.000	-1.975	0.050
ENSG00000253174.2_RP11-360L9.7	RP11-360L9.7	ENSG00000253174.2	-0.031	0.016	-1.976	0.050
ENSG00000060566.8_CREB3L3	CREB3L3	ENSG00000060566.8	-0.065	0.033	-1.976	0.050
ENSG00000132386.5_SERPINF1	SERPINF1	ENSG00000132386.5	-2.553	1.292	-1.976	0.050
ENSG00000189134.3_NKAPL	NKAPL	ENSG00000189134.3	-0.439	0.222	-1.976	0.050
ENSG00000147432.2_CHRNB3	CHRNB3	ENSG00000147432.2	-0.017	0.009	-1.977	0.050
ENSG00000264695.1_RP11-178F10.2	RP11-178F10.2	ENSG00000264695.1	-0.020	0.010	-1.977	0.050
ENSG00000229107.2_FAM108A5P	FAM108A5P	ENSG00000229107.2	-0.026	0.013	-1.977	0.050

ENSG00000023516.7_AKAP11	AKAP11	ENSG00000023516.7	-0.695	0.352	-1.977	0.050
ENSG00000259262.1_RP11-154B12.2	RP11-154B12.2	ENSG00000259262.1	-0.101	0.051	-1.977	0.050
ENSG00000198785.4_GRIN3A	GRIN3A	ENSG00000198785.4	-0.085	0.043	-1.977	0.050
ENSG00000259158.1_ADAM20P1	ADAM20P1	ENSG00000259158.1	-0.081	0.041	-1.978	0.050
ENSG00000186675.5_MAGEE2	MAGEE2	ENSG00000186675.5	-0.142	0.072	-1.978	0.050
ENSG00000250957.1_RP11-248N22.1	RP11-248N22.1	ENSG00000250957.1	-0.006	0.003	-1.978	0.050
ENSG00000113522.9_RAD50	RAD50	ENSG00000113522.9	-0.728	0.368	-1.978	0.050
ENSG00000205293.3_RP11-1112C15.1	RP11-1112C15.1	ENSG00000205293.3	-0.003	0.002	-1.979	0.049
ENSG00000260838.1_RP11-531A24.3	RP11-531A24.3	ENSG00000260838.1	-0.107	0.054	-1.979	0.049
ENSG00000164900.4_GBX1	GBX1	ENSG00000164900.4	-0.006	0.003	-1.979	0.049
ENSG00000228376.2_RP11-520B13.4	RP11-520B13.4	ENSG00000228376.2	-0.093	0.047	-1.979	0.049
ENSG00000136197.7_C7orf25	C7orf25	ENSG00000136197.7	-0.290	0.147	-1.979	0.049
ENSG00000172137.14_CALB2	CALB2	ENSG00000172137.14	-1.207	0.610	-1.980	0.049
ENSG00000233457.1_RP11-336N8.1	RP11-336N8.1	ENSG00000233457.1	-0.011	0.006	-1.980	0.049
ENSG00000169851.10_PCDH7	PCDH7	ENSG00000169851.10	-0.554	0.280	-1.980	0.049
ENSG00000267319.1_CTD-2528L19.3	CTD-2528L19.3	ENSG00000267319.1	-0.081	0.041	-1.981	0.049
ENSG00000215126.5_CBWD7	CBWD7	ENSG00000215126.5	-0.359	0.181	-1.981	0.049
ENSG00000107147.6_KCNT1	KCNT1	ENSG00000107147.6	-2.089	1.055	-1.981	0.049
ENSG00000158528.7_PPP1R9A	PPP1R9A	ENSG00000158528.7	-0.287	0.145	-1.981	0.049
ENSG00000257642.1_RP11-474B16.1	RP11-474B16.1	ENSG00000257642.1	-0.017	0.009	-1.981	0.049
ENSG00000240661.1_RP11-174O3.3	RP11-174O3.3	ENSG00000240661.1	-0.020	0.010	-1.982	0.049
ENSG00000117215.10_PLA2G2D	PLA2G2D	ENSG00000117215.10	-0.003	0.002	-1.982	0.049
ENSG00000186952.10_TMEM232	TMEM232	ENSG00000186952.10	-0.106	0.054	-1.982	0.049
ENSG00000152932.6_RAB3C	RAB3C	ENSG00000152932.6	-0.853	0.430	-1.983	0.049
ENSG00000236520.1_GPC6-AS1	GPC6-AS1	ENSG00000236520.1	0.000	0.000	-1.983	0.049
ENSG00000197584.7_KCNMB2	KCNMB2	ENSG00000197584.7	-0.105	0.053	-1.984	0.049
ENSG00000223356.1_RP11-66D17.5	RP11-66D17.5	ENSG00000223356.1	-0.243	0.123	-1.985	0.049
ENSG00000125977.6_EIF2S2	EIF2S2	ENSG00000125977.6	-1.264	0.637	-1.985	0.049
ENSG00000259467.1_NDUFAF4P1	NDUFAF4P1	ENSG00000259467.1	-0.017	0.009	-1.985	0.049
ENSG00000171129.7_HSFX2	HSFX2	ENSG00000171129.7	-0.236	0.119	-1.986	0.049
ENSG00000256995.2_RP11-114G22.1	RP11-114G22.1	ENSG00000256995.2	-0.026	0.013	-1.986	0.049
ENSG00000177511.5_ST8SIA3	ST8SIA3	ENSG00000177511.5	-3.755	1.889	-1.987	0.049
ENSG00000132740.4_IGHMBP2	IGHMBP2	ENSG00000132740.4	-0.313	0.157	-1.987	0.049
ENSG00000159708.13_LRRC36	LRRC36	ENSG00000159708.13	-0.055	0.028	-1.987	0.049
ENSG00000145365.9_TIFA	TIFA	ENSG00000145365.9	-0.086	0.044	-1.987	0.048
ENSG00000163071.6_SPATA18	SPATA18	ENSG00000163071.6	-0.032	0.016	-1.988	0.048
ENSG00000159784.13_FAM131B	FAM131B	ENSG00000159784.13	-1.300	0.654	-1.988	0.048
ENSG00000237361.1_RP11-269C23.3	RP11-269C23.3	ENSG00000237361.1	-0.016	0.008	-1.989	0.048
ENSG00000155511.10_GRIA1	GRIA1	ENSG00000155511.10	-0.785	0.395	-1.989	0.048
ENSG00000162694.9_EXTL2	EXTL2	ENSG00000162694.9	-0.599	0.301	-1.990	0.048
ENSG00000144746.6_ARL6IP5	ARL6IP5	ENSG00000144746.6	-2.244	1.128	-1.990	0.048
ENSG00000253733.1_LZTS1-AS1	LZTS1-AS1	ENSG00000253733.1	0.000	0.000	-1.991	0.048
ENSG00000265399.1_RP13-270P17.2	RP13-270P17.2	ENSG00000265399.1	-0.003	0.002	-1.991	0.048
ENSG00000243864.1_RPS3AP50	RPS3AP50	ENSG00000243864.1	-0.015	0.008	-1.992	0.048
ENSG00000152214.7_RIT2	RIT2	ENSG00000152214.7	-0.797	0.400	-1.992	0.048
ENSG00000188674.6_C2orf80	C2orf80	ENSG00000188674.6	-1.173	0.589	-1.992	0.048
ENSG00000024862.12_CCDC28A	CCDC28A	ENSG00000024862.12	-0.663	0.333	-1.992	0.048
ENSG00000091844.2_RGS17	RGS17	ENSG00000091844.2	-0.225	0.113	-1.992	0.048
ENSG00000220548.2_VIMP1	VIMP1	ENSG00000220548.2	-0.009	0.005	-1.992	0.048
ENSG00000197927.7_C2orf27A	C2orf27A	ENSG00000197927.7	-0.243	0.122	-1.993	0.048
ENSG00000214140.5_PRCD	PRCD	ENSG00000214140.5	-0.597	0.299	-1.994	0.048
ENSG00000050426.10_LETMD1	LETMD1	ENSG00000050426.10	-1.041	0.522	-1.994	0.048
ENSG00000127914.12_AKAP9	AKAP9	ENSG00000127914.12	-1.482	0.743	-1.994	0.048
ENSG00000167257.6_RNF214	RNF214	ENSG00000167257.6	-0.210	0.105	-1.994	0.048
ENSG00000267713.1_RP11-10I6.2	RP11-10I6.2	ENSG00000267713.1	-0.056	0.028	-1.995	0.048
ENSG00000015676.12_NUDCD3	NUDCD3	ENSG00000015676.12	-1.423	0.713	-1.995	0.048
ENSG00000132872.5_SYT4	SYT4	ENSG00000132872.5	-1.744	0.874	-1.995	0.048
ENSG00000226383.1_AC093375.1	AC093375.1	ENSG00000226383.1	-0.006	0.003	-1.996	0.048
ENSG00000224153.2_RP11-433C9.2	RP11-433C9.2	ENSG00000224153.2	-0.220	0.110	-1.996	0.048
ENSG00000260083.1_MIR4519	MIR4519	ENSG00000260083.1	-0.168	0.084	-1.996	0.048

ENSG00000233208.1_LINC00642	LINC00642	ENSG00000233208.1	-0.051	0.025	-1.997	0.047
ENSG00000236043.1_RP11-78C6.1	RP11-78C6.1	ENSG00000236043.1	-0.026	0.013	-1.997	0.047
ENSG00000134389.8_CFHR5	CFHR5	ENSG00000134389.8	-0.004	0.002	-1.997	0.047
ENSG00000133858.11_ZFC3H1	ZFC3H1	ENSG00000133858.11	-0.270	0.135	-1.997	0.047
ENSG00000120756.8_PLS1	PLS1	ENSG00000120756.8	-0.084	0.042	-1.997	0.047
ENSG00000259046.1_RP11-857B24.2	RP11-857B24.2	ENSG00000259046.1	-0.008	0.004	-1.998	0.047
ENSG00000198919.8_DZIP3	DZIP3	ENSG00000198919.8	-0.635	0.318	-1.998	0.047
ENSG00000170325.10_PRDM10	PRDM10	ENSG00000170325.10	-0.070	0.035	-1.998	0.047
ENSG00000127588.4_GNG13	GNG13	ENSG00000127588.4	-0.661	0.331	-1.999	0.047
ENSG00000110427.10_KIAA1549L	KIAA1549L	ENSG00000110427.10	-0.686	0.343	-2.000	0.047
ENSG00000185069.2_KRT76	KRT76	ENSG00000185069.2	0.000	0.000	-2.000	0.047
ENSG00000134909.13_ARHGAP32	ARHGAP32	ENSG00000134909.13	-0.696	0.348	-2.000	0.047
ENSG00000182400.10_TRAPPC6B	TRAPPC6B	ENSG00000182400.10	-0.642	0.321	-2.000	0.047
ENSG00000018189.7_RUFY3	RUFY3	ENSG00000018189.7	-1.275	0.637	-2.001	0.047
ENSG00000183049.8_CAMK1D	CAMK1D	ENSG00000183049.8	-1.586	0.793	-2.001	0.047
ENSG00000260917.1_RP11-57H14.4	RP11-57H14.4	ENSG00000260917.1	-0.149	0.074	-2.003	0.047
ENSG00000184613.6_NELL2	NELL2	ENSG00000184613.6	-3.552	1.773	-2.003	0.047
ENSG00000080546.9_SESN1	SESN1	ENSG00000080546.9	-0.858	0.428	-2.003	0.047
ENSG00000181240.8_SLC25A41	SLC25A41	ENSG00000181240.8	-0.485	0.242	-2.003	0.047
ENSG00000106331.10_PAX4	PAX4	ENSG00000106331.10	-0.003	0.001	-2.004	0.047
ENSG00000119705.5_SLIRP	SLIRP	ENSG00000119705.5	-6.270	3.129	-2.004	0.047
ENSG00000132911.4_NMUR2	NMUR2	ENSG00000132911.4	-0.024	0.012	-2.004	0.047
ENSG00000250254.1_PTTG2	PTTG2	ENSG00000250254.1	-0.063	0.031	-2.004	0.047
ENSG00000077063.6_CTTNBP2	CTTNBP2	ENSG00000077063.6	-0.207	0.103	-2.004	0.047
ENSG00000219186.2_FTH1P19	FTH1P19	ENSG00000219186.2	-0.095	0.047	-2.004	0.047
ENSG00000104435.8_STMN2	STMN2	ENSG00000104435.8	-18.013	8.986	-2.005	0.047
ENSG00000164985.9_PSIP1	PSIP1	ENSG00000164985.9	-2.768	1.381	-2.005	0.047
ENSG00000227612.1_RP5-1177E19.2	RP5-1177E19.2	ENSG00000227612.1	0.000	0.000	-2.005	0.047
ENSG00000082497.7_SERTAD4	SERTAD4	ENSG00000082497.7	-0.102	0.051	-2.005	0.047
ENSG00000003393.10_ALS2	ALS2	ENSG00000003393.10	-0.222	0.111	-2.006	0.046
ENSG00000125962.7_ARMCX5	ARMCX5	ENSG00000125962.7	-0.458	0.228	-2.006	0.046
ENSG00000199319.1_7SK	RN7SKP25	ENSG00000199319.1	0.000	0.000	-2.006	0.046
ENSG00000181013.3_C17orf47	C17orf47	ENSG00000181013.3	-0.001	0.001	-2.006	0.046
ENSG00000197852.7_FAM212B	FAM212B	ENSG00000197852.7	-0.431	0.215	-2.006	0.046
ENSG00000166840.8_GLYATL1	GLYATL1	ENSG00000166840.8	-0.020	0.010	-2.007	0.046
ENSG00000104442.5_ARMC1	ARMC1	ENSG00000104442.5	-0.382	0.191	-2.007	0.046
ENSG00000106701.7_FSD1L	FSD1L	ENSG00000106701.7	-0.198	0.099	-2.007	0.046
ENSG00000113088.5_GZMK	GZMK	ENSG00000113088.5	-0.008	0.004	-2.008	0.046
ENSG00000151092.12_NGLY1	NGLY1	ENSG00000151092.12	-0.238	0.118	-2.008	0.046
ENSG00000227663.1_RPL7P2	RPL7P2	ENSG00000227663.1	-0.001	0.000	-2.009	0.046
ENSG00000242578.1_RP11-469J4.3	RP11-469J4.3	ENSG00000242578.1	-0.012	0.006	-2.009	0.046
ENSG00000162545.5_CAMK2N1	CAMK2N1	ENSG00000162545.5	-11.694	5.820	-2.009	0.046
ENSG00000101882.5_NKAP	NKAP	ENSG00000101882.5	-0.744	0.370	-2.009	0.046
ENSG00000103035.5_PSMD7	PSMD7	ENSG00000103035.5	-1.495	0.744	-2.010	0.046
ENSG00000189139.5_FSCB	FSCB	ENSG00000189139.5	-0.004	0.002	-2.010	0.046
ENSG00000168484.8_SFTPC	SFTPC	ENSG00000168484.8	-0.512	0.255	-2.010	0.046
ENSG00000126950.7_TMEM35	TMEM35	ENSG00000126950.7	-1.183	0.588	-2.011	0.046
ENSG00000241878.5_PISD	PISD	ENSG00000241878.5	-1.021	0.508	-2.011	0.046
ENSG00000137876.5_RSL24D1	RSL24D1	ENSG00000137876.5	-0.869	0.432	-2.011	0.046
ENSG00000141384.7_TAF4B	TAF4B	ENSG00000141384.7	-0.145	0.072	-2.012	0.046
ENSG00000266667.1_RP11-849N15.4	RP11-849N15.4	ENSG00000266667.1	-0.001	0.001	-2.013	0.046
ENSG00000146151.8_HMGCLL1	HMGCLL1	ENSG00000146151.8	-0.330	0.164	-2.015	0.045
ENSG00000136918.3_WDR38	WDR38	ENSG00000136918.3	-0.025	0.012	-2.015	0.045
ENSG00000196482.12_ESRRG	ESRRG	ENSG00000196482.12	-0.127	0.063	-2.015	0.045
ENSG00000145293.10_ENOPH1	ENOPH1	ENSG00000145293.10	-1.105	0.548	-2.015	0.045
ENSG00000183072.9_NKX2-5	NKX2-5	ENSG00000183072.9	-0.001	0.001	-2.016	0.045
ENSG00000175768.8_TOMM5	TOMM5	ENSG00000175768.8	-1.655	0.821	-2.016	0.045
ENSG00000162989.3_KCNJ3	KCNJ3	ENSG00000162989.3	-0.594	0.295	-2.016	0.045
ENSG00000233894.1_RP4-650F12.2	RP4-650F12.2	ENSG00000233894.1	-0.152	0.075	-2.017	0.045
ENSG00000164112.8_TMEM155	TMEM155	ENSG00000164112.8	-0.801	0.397	-2.017	0.045

ENSG00000256870.2_SLC5A8	SLC5A8	ENSG00000256870.2	-0.018	0.009	-2.017	0.045
ENSG00000127955.11_GNAI1	GNAI1	ENSG00000127955.11	-0.572	0.284	-2.018	0.045
ENSG00000184675.5_FAM123B	FAM123B	ENSG00000184675.5	-0.041	0.021	-2.019	0.045
ENSG00000066422.4_ZBTB11	ZBTB11	ENSG00000066422.4	-0.199	0.099	-2.019	0.045
ENSG00000143847.10_PPFIA4	PPFIA4	ENSG00000143847.10	-1.373	0.680	-2.019	0.045
ENSG00000138495.2_COX17	COX17	ENSG00000138495.2	-1.932	0.957	-2.020	0.045
ENSG00000230622.1_UQCRHP1	UQCRHP1	ENSG00000230622.1	-0.042	0.021	-2.020	0.045
ENSG00000248699.1_CTD-2313D3.1	CTD-2313D3.1	ENSG00000248699.1	-0.002	0.001	-2.021	0.045
ENSG00000166863.7_TAC3	TAC3	ENSG00000166863.7	-0.876	0.433	-2.021	0.045
ENSG00000261305.1_RP4-584D14.7	RP4-584D14.7	ENSG00000261305.1	-0.020	0.010	-2.021	0.045
ENSG00000135472.3_FAIM2	FAIM2	ENSG00000135472.3	-9.492	4.695	-2.022	0.045
ENSG00000231165.2_TRBV26OR9-2	TRBV26OR9-2	ENSG00000231165.2	-0.036	0.018	-2.022	0.045
ENSG00000144550.8_CPNE9	CPNE9	ENSG00000144550.8	-1.040	0.514	-2.023	0.045
ENSG00000165659.12_DACH1	DACH1	ENSG00000165659.12	-0.067	0.033	-2.023	0.045
ENSG00000236785.1_AC007248.7	AC007248.7	ENSG00000236785.1	-0.001	0.001	-2.023	0.045
ENSG00000015153.10_YAF2	YAF2	ENSG00000015153.10	-1.573	0.777	-2.024	0.045
ENSG00000129084.13_PSMA1	PSMA1	ENSG00000129084.13	-1.805	0.892	-2.024	0.045
ENSG00000091157.9_WDR7	WDR7	ENSG00000091157.9	-0.498	0.246	-2.024	0.045
ENSG00000168438.8_CDC40	CDC40	ENSG00000168438.8	-0.440	0.218	-2.024	0.044
ENSG00000087502.13_ERGIC2	ERGIC2	ENSG00000087502.13	-0.957	0.473	-2.025	0.044
ENSG00000251381.2_CTC-497E21.4	CTC-497E21.4	ENSG00000251381.2	-0.067	0.033	-2.026	0.044
ENSG00000267152.1_CTD-2528L19.6	CTD-2528L19.6	ENSG00000267152.1	-0.114	0.056	-2.026	0.044
ENSG00000143756.7_FBXO28	FBXO28	ENSG00000143756.7	-0.219	0.108	-2.027	0.044
ENSG00000203756.3_TMEM244	TMEM244	ENSG00000203756.3	-0.027	0.013	-2.027	0.044
ENSG00000113163.9_COL4A3BP	COL4A3BP	ENSG00000113163.9	-0.371	0.183	-2.028	0.044
ENSG00000180957.12_PITPNB	PITPNB	ENSG00000180957.12	-0.829	0.409	-2.028	0.044
ENSG00000163947.7_ARHGEF3	ARHGEF3	ENSG00000163947.7	-0.803	0.396	-2.029	0.044
ENSG00000114948.8_ADAM23	ADAM23	ENSG00000114948.8	-0.873	0.430	-2.029	0.044
ENSG00000186868.11_MAPT	MAPT	ENSG00000186868.11	-3.660	1.804	-2.029	0.044
ENSG00000166848.4_TERF2IP	TERF2IP	ENSG00000166848.4	-3.738	1.841	-2.030	0.044
ENSG00000143387.8_CTSK	CTSK	ENSG00000143387.8	-0.126	0.062	-2.031	0.044
ENSG00000134056.6_MRPS36	MRPS36	ENSG00000134056.6	-0.457	0.225	-2.031	0.044
ENSG00000198216.6_CACNA1E	CACNA1E	ENSG00000198216.6	-0.249	0.122	-2.032	0.044
ENSG00000230935.1_RPS3P1	RPS3P1	ENSG00000230935.1	0.000	0.000	-2.032	0.044
ENSG00000125352.4_RNF113A	RNF113A	ENSG00000125352.4	-0.356	0.175	-2.032	0.044
ENSG00000228983.4_AC025627.7	AC025627.7	ENSG00000228983.4	-0.022	0.011	-2.033	0.044
ENSG00000143198.7_MGST3	MGST3	ENSG00000143198.7	-6.425	3.158	-2.034	0.043
ENSG00000124568.6_SLC17A1	SLC17A1	ENSG00000124568.6	-0.003	0.002	-2.034	0.043
ENSG00000236541.1_VN2R9P	VN2R9P	ENSG00000236541.1	-0.011	0.005	-2.036	0.043
ENSG00000259076.1_RP11-973N13.3	RP11-973N13.3	ENSG00000259076.1	-0.001	0.001	-2.036	0.043
ENSG00000260668.1_RP11-744D14.1	RP11-744D14.1	ENSG00000260668.1	-0.008	0.004	-2.038	0.043
ENSG00000261965.1_ISCA1P3	ISCA1P3	ENSG00000261965.1	-0.002	0.001	-2.038	0.043
ENSG00000170571.6_EMB	EMB	ENSG00000170571.6	-0.073	0.036	-2.038	0.043
ENSG00000267194.1_RP1-193H18.2	RP1-193H18.2	ENSG00000267194.1	-0.132	0.065	-2.040	0.043
ENSG00000251609.1_SETP12	SETP12	ENSG00000251609.1	-0.041	0.020	-2.041	0.043
ENSG00000163497.2_FEV	FEV	ENSG00000163497.2	-0.064	0.031	-2.042	0.043
ENSG00000198934.3_MAGEE1	MAGEE1	ENSG00000198934.3	-1.108	0.543	-2.042	0.043
ENSG00000169018.5_FEM1B	FEM1B	ENSG00000169018.5	-0.389	0.190	-2.042	0.043
ENSG00000165259.8_HDX	HDX	ENSG00000165259.8	-0.040	0.019	-2.042	0.043
ENSG00000077092.14_RARB	RARB	ENSG00000077092.14	-0.227	0.111	-2.042	0.043
ENSG00000250232.1_AF196779.12	AF196779.12	ENSG00000250232.1	-0.018	0.009	-2.042	0.043
ENSG00000120458.5_MSANTD2	MSANTD2	ENSG00000120458.5	-0.132	0.065	-2.042	0.043
ENSG00000215174.2_RP13-487C10.1	RP13-487C10.1	ENSG00000215174.2	-0.022	0.011	-2.043	0.043
ENSG00000156026.9_MCU	MCU	ENSG00000156026.9	-0.175	0.086	-2.044	0.042
ENSG00000241391.2_MetazoaSRP	RN7SL234P	ENSG00000241391.2	-0.001	0.000	-2.044	0.042
ENSG00000165061.10_ZMAT4	ZMAT4	ENSG00000165061.10	-0.594	0.290	-2.045	0.042
ENSG00000184523.2_PTGER4P2	PTGER4P2	ENSG00000184523.2	-0.062	0.030	-2.045	0.042
ENSG00000198900.5_TOP1	TOP1	ENSG00000198900.5	-0.716	0.350	-2.045	0.042
ENSG00000267326.1_AC015849.14	AC015849.14	ENSG00000267326.1	0.000	0.000	-2.045	0.042
ENSG00000152332.11_UHMK1	UHMK1	ENSG00000152332.11	-0.386	0.189	-2.045	0.042

ENSG00000218336.3_ODZ3	ODZ3	ENSG00000218336.3	-0.152	0.074	-2.046	0.042
ENSG00000134058.6_CDK7	CDK7	ENSG00000134058.6	-0.326	0.159	-2.047	0.042
ENSG00000235026.1_AC066593.1	AC066593.1	ENSG00000235026.1	-0.459	0.224	-2.047	0.042
ENSG00000183454.9_GRIN2A	GRIN2A	ENSG00000183454.9	-0.771	0.377	-2.048	0.042
ENSG00000249984.1_CTC-529L17.2	CTC-529L17.2	ENSG00000249984.1	-0.011	0.005	-2.048	0.042
ENSG00000236607.3_RP11-691L4.2	RP11-691L4.2	ENSG00000236607.3	-0.001	0.001	-2.049	0.042
ENSG00000214517.4_PPME1	PPME1	ENSG00000214517.4	-2.074	1.012	-2.050	0.042
ENSG00000265500.1_SENP3-EIF4A1	SENP3-EIF4A1	ENSG00000265500.1	-0.017	0.008	-2.051	0.042
ENSG00000112367.6_FIG4	FIG4	ENSG00000112367.6	-0.522	0.254	-2.051	0.042
ENSG00000171714.10_ANO5	ANO5	ENSG00000171714.10	-0.166	0.081	-2.051	0.042
ENSG00000168004.5_HRASLS5	HRASLS5	ENSG00000168004.5	-0.116	0.057	-2.051	0.042
ENSG00000101928.8_MOSPD1	MOSPD1	ENSG00000101928.8	-0.113	0.055	-2.052	0.042
ENSG00000152503.5_TRIM36	TRIM36	ENSG00000152503.5	-0.501	0.244	-2.052	0.042
ENSG00000204442.1_FAM155A	FAM155A	ENSG00000204442.1	-0.529	0.258	-2.052	0.042
ENSG00000228549.1_RP11-108M9.3	RP11-108M9.3	ENSG00000228549.1	-0.016	0.008	-2.052	0.042
ENSG00000171723.11_GPHN	GPHN	ENSG00000171723.11	-1.070	0.521	-2.053	0.042
ENSG00000148377.5_IDI2	IDI2	ENSG00000148377.5	-0.007	0.003	-2.053	0.042
ENSG00000116983.8_HPCAL4	HPCAL4	ENSG00000116983.8	-2.347	1.143	-2.053	0.042
ENSG00000153815.10_CMIP	CMIP	ENSG00000153815.10	-1.419	0.691	-2.054	0.042
ENSG00000167863.7_ATP5H	ATP5H	ENSG00000167863.7	-8.244	4.013	-2.054	0.041
ENSG00000129625.8_REEP5	REEP5	ENSG00000129625.8	-2.808	1.366	-2.056	0.041
ENSG00000122565.13_CBX3	CBX3	ENSG00000122565.13	-0.884	0.430	-2.056	0.041
ENSG00000207513.1_RNU1-3	RNU1-3	ENSG00000207513.1	-1.194	0.581	-2.056	0.041
ENSG00000262484.1_CTC-360G5.1	CTC-360G5.1	ENSG00000262484.1	-0.375	0.182	-2.056	0.041
ENSG00000233714.1_AC012506.2	AC012506.2	ENSG00000233714.1	-0.001	0.000	-2.057	0.041
ENSG00000231216.1_GS1-600G8.3	GS1-600G8.3	ENSG00000231216.1	-0.003	0.001	-2.057	0.041
ENSG00000244019.1_CTC-337B15.1	CTC-337B15.1	ENSG00000244019.1	-0.006	0.003	-2.059	0.041
ENSG00000237321.1_RP11-374A22.1	RP11-374A22.1	ENSG00000237321.1	-0.024	0.012	-2.059	0.041
ENSG00000174442.7_ZWILCH	ZWILCH	ENSG00000174442.7	-0.107	0.052	-2.059	0.041
ENSG00000258399.1_MEG8	MEG8	ENSG00000258399.1	-0.320	0.155	-2.059	0.041
ENSG00000232348.1_LINC00279	LINC00279	ENSG00000232348.1	-0.001	0.001	-2.059	0.041
ENSG00000130830.9_MPP1	MPP1	ENSG00000130830.9	-1.348	0.654	-2.060	0.041
ENSG00000100483.9_METTL21D	METTL21D	ENSG00000100483.9	-0.238	0.115	-2.060	0.041
ENSG00000132958.13_TPTE2	TPTE2	ENSG00000132958.13	-0.030	0.014	-2.061	0.041
ENSG00000168772.9_CXXC4	CXXC4	ENSG00000168772.9	-0.231	0.112	-2.061	0.041
ENSG00000143061.13_IGSF3	IGSF3	ENSG00000143061.13	-0.130	0.063	-2.063	0.041
ENSG00000260124.1_RP4-791K14.2	RP4-791K14.2	ENSG00000260124.1	-1.564	0.758	-2.064	0.041
ENSG00000185215.4_TNFAIP2	TNFAIP2	ENSG00000185215.4	-1.154	0.558	-2.066	0.040
ENSG00000258544.1_RP11-750I4.2	RP11-750I4.2	ENSG00000258544.1	-0.064	0.031	-2.067	0.040
ENSG00000115310.13_RTN4	RTN4	ENSG00000115310.13	-8.977	4.343	-2.067	0.040
ENSG00000188343.8_FAM92A1	FAM92A1	ENSG00000188343.8	-0.803	0.388	-2.067	0.040
ENSG00000132640.10_BTBD3	BTBD3	ENSG00000132640.10	-0.732	0.354	-2.069	0.040
ENSG00000115392.7_FANCL	FANCL	ENSG00000115392.7	-0.444	0.215	-2.069	0.040
ENSG00000106105.9_GARS	GARS	ENSG00000106105.9	-1.927	0.931	-2.070	0.040
ENSG00000265912.1_RP11-583F2.2	RP11-583F2.2	ENSG00000265912.1	-0.003	0.002	-2.070	0.040
ENSG00000135638.8_EMX1	EMX1	ENSG00000135638.8	-1.316	0.636	-2.070	0.040
ENSG00000213551.3_DNAJC9	DNAJC9	ENSG00000213551.3	-0.416	0.201	-2.071	0.040
ENSG00000231672.2_DIRC3	DIRC3	ENSG00000231672.2	-0.024	0.012	-2.072	0.040
ENSG00000022355.10_GABRA1	GABRA1	ENSG00000022355.10	-2.311	1.115	-2.072	0.040
ENSG00000198825.7_INPP5F	INPP5F	ENSG00000198825.7	-2.610	1.260	-2.073	0.040
ENSG00000249463.1_RP11-1E22.1	RP11-1E22.1	ENSG00000249463.1	-0.062	0.030	-2.073	0.040
ENSG00000086289.7_EPDR1	EPDR1	ENSG00000086289.7	-2.063	0.994	-2.075	0.040
ENSG00000116030.12_SUMO1	SUMO1	ENSG00000116030.12	-1.576	0.759	-2.075	0.039
ENSG00000108278.7_ZNHIT3	ZNHIT3	ENSG00000108278.7	-1.032	0.497	-2.075	0.039
ENSG00000229495.1_RP11-173D14.3	RP11-173D14.3	ENSG00000229495.1	-0.005	0.002	-2.075	0.039
ENSG00000127399.10_LRRC61	LRRC61	ENSG00000127399.10	-0.392	0.189	-2.075	0.039
ENSG00000149295.9_DRD2	DRD2	ENSG00000149295.9	-0.058	0.028	-2.076	0.039
ENSG00000224830.1_RP11-438H8.9	RP11-438H8.9	ENSG00000224830.1	-0.002	0.001	-2.076	0.039
ENSG00000264036.1_MetazoaSRP	RN7SL198P	ENSG00000264036.1	-0.014	0.007	-2.077	0.039
ENSG00000162630.5_B3GALT2	B3GALT2	ENSG00000162630.5	-0.424	0.204	-2.077	0.039

ENSG00000175322.6_ZNF519	ZNF519	ENSG00000175322.6	-0.164	0.079	-2.078	0.039
ENSG00000234036.3_RP11-123J14.2	RP11-123J14.2	ENSG00000234036.3	-0.001	0.000	-2.078	0.039
ENSG00000237433.1_AC097639.8	AC097639.8	ENSG00000237433.1	-0.023	0.011	-2.078	0.039
ENSG00000100218.7_RTDR1	RTDR1	ENSG00000100218.7	-0.297	0.143	-2.078	0.039
ENSG00000144827.4_ABHD10	ABHD10	ENSG00000144827.4	-0.438	0.211	-2.079	0.039
ENSG00000067445.15_TRO	TRO	ENSG00000067445.15	-1.068	0.514	-2.079	0.039
ENSG00000261658.1_RP11-102F4.3	RP11-102F4.3	ENSG00000261658.1	-0.016	0.008	-2.079	0.039
ENSG00000112077.11_RHAG	RHAG	ENSG00000112077.11	-0.005	0.002	-2.079	0.039
ENSG00000180543.3_TSPYL5	TSPYL5	ENSG00000180543.3	-0.336	0.162	-2.079	0.039
ENSG00000167281.13_RBFOX3	RBFOX3	ENSG00000167281.13	-7.014	3.374	-2.079	0.039
ENSG00000267122.1_AC004490.1	AC004490.1	ENSG00000267122.1	-0.028	0.013	-2.080	0.039
ENSG00000166006.8_KCNC2	KCNC2	ENSG00000166006.8	-1.230	0.591	-2.080	0.039
ENSG00000139190.12_VAMP1	VAMP1	ENSG00000139190.12	-6.768	3.253	-2.081	0.039
ENSG00000106415.8_GLCCI1	GLCCI1	ENSG00000106415.8	-0.297	0.143	-2.081	0.039
ENSG00000196632.6_WNK3	WNK3	ENSG00000196632.6	-0.038	0.018	-2.083	0.039
ENSG00000135999.7_EPC2	EPC2	ENSG00000135999.7	-0.234	0.112	-2.084	0.039
ENSG00000161544.5_CYGB	CYGB	ENSG00000161544.5	-0.779	0.374	-2.084	0.039
ENSG00000226284.1_ARPC3P1	ARPC3P1	ENSG00000226284.1	-0.002	0.001	-2.084	0.039
ENSG00000139200.8_PIANP	PIANP	ENSG00000139200.8	-3.503	1.681	-2.085	0.039
ENSG00000130037.3_KCNA5	KCNA5	ENSG00000130037.3	-0.140	0.067	-2.085	0.039
ENSG00000230896.1_RP11-767N6.7	RP11-767N6.7	ENSG00000230896.1	-0.164	0.079	-2.085	0.039
ENSG00000237766.2_RP11-512G13.1	RP11-512G13.1	ENSG00000237766.2	-0.125	0.060	-2.085	0.039
ENSG00000065559.9_MAP2K4	MAP2K4	ENSG00000065559.9	-1.243	0.596	-2.085	0.039
ENSG00000134940.8_ACRV1	ACRV1	ENSG00000134940.8	-0.141	0.067	-2.086	0.038
ENSG00000154162.8_CDH12	CDH12	ENSG00000154162.8	-0.100	0.048	-2.086	0.038
ENSG00000109475.12_RPL34	RPL34	ENSG00000109475.12	-29.022	13.913	-2.086	0.038
ENSG00000234352.3_AC009264.1	AC009264.1	ENSG00000234352.3	-0.002	0.001	-2.086	0.038
ENSG00000119042.11_SATB2	SATB2	ENSG00000119042.11	-0.455	0.218	-2.086	0.038
ENSG00000036054.8_TBC1D23	TBC1D23	ENSG00000036054.8	-0.188	0.090	-2.086	0.038
ENSG00000125898.7_FAM110A	FAM110A	ENSG00000125898.7	-0.172	0.082	-2.087	0.038
ENSG00000251188.1_RP11-478C6.6	RP11-478C6.6	ENSG00000251188.1	-0.036	0.017	-2.087	0.038
ENSG00000148229.8_POLE3	POLE3	ENSG00000148229.8	-0.513	0.246	-2.087	0.038
ENSG00000182916.7_TCEAL7	TCEAL7	ENSG00000182916.7	-2.605	1.248	-2.087	0.038
ENSG00000236172.2_AC097517.2	AC097517.2	ENSG00000236172.2	0.000	0.000	-2.087	0.038
ENSG00000260230.1_RP11-526J3.3	RP11-526J3.3	ENSG00000260230.1	-1.108	0.531	-2.088	0.038
ENSG00000204071.5_TCEAL6	TCEAL6	ENSG00000204071.5	-2.758	1.321	-2.088	0.038
ENSG00000265533.1_RP11-638L3.1	RP11-638L3.1	ENSG00000265533.1	-0.003	0.002	-2.088	0.038
ENSG00000180771.9_SRSF8	SRSF8	ENSG00000180771.9	-0.396	0.190	-2.088	0.038
ENSG00000204789.3_ZNF204P	ZNF204P	ENSG00000204789.3	-0.847	0.406	-2.089	0.038
ENSG00000235628.1_TNR-IT1	TNR-IT1	ENSG00000235628.1	-0.016	0.008	-2.089	0.038
ENSG00000183775.4_KCTD16	KCTD16	ENSG00000183775.4	-0.158	0.076	-2.090	0.038
ENSG00000087152.11_ATXN7L3	ATXN7L3	ENSG00000087152.11	-2.661	1.273	-2.091	0.038
ENSG00000058091.12_CDK14	CDK14	ENSG00000058091.12	-1.035	0.495	-2.091	0.038
ENSG00000123505.9_AMD1	AMD1	ENSG00000123505.9	-0.940	0.450	-2.091	0.038
ENSG00000264765.1_RP11-92B11.4	RP11-92B11.4	ENSG00000264765.1	-0.011	0.005	-2.091	0.038
ENSG00000230809.1_RP11-309P22.1	RP11-309P22.1	ENSG00000230809.1	-8.577	4.100	-2.092	0.038
ENSG00000231589.1_AC009110.1	AC009110.1	ENSG00000231589.1	0.000	0.000	-2.092	0.038
ENSG00000266728.1_AC015688.3	AC015688.3	ENSG00000266728.1	0.000	0.000	-2.092	0.038
ENSG00000235689.1_AP000351.13	AP000351.13	ENSG00000235689.1	0.000	0.000	-2.092	0.038
ENSG00000237735.1_AP000473.6	AP000473.6	ENSG00000237735.1	0.000	0.000	-2.092	0.038
ENSG00000255102.1_RP11-164N3.2	RP11-164N3.2	ENSG00000255102.1	0.000	0.000	-2.092	0.038
ENSG00000241599.1_RP11-34P13.9	RP11-34P13.9	ENSG00000241599.1	0.000	0.000	-2.092	0.038
ENSG00000263343.1_RP11-388M20.7	RP11-388M20.7	ENSG00000263343.1	0.000	0.000	-2.092	0.038
ENSG00000254043.1_RP11-3N13.2	RP11-3N13.2	ENSG00000254043.1	0.000	0.000	-2.092	0.038
ENSG00000218472.2_RP1-140K8.2	RP1-140K8.2	ENSG00000218472.2	0.000	0.000	-2.092	0.038
ENSG00000224034.1_RP11-445P17.8	RP11-445P17.8	ENSG00000224034.1	0.000	0.000	-2.092	0.038
ENSG00000258666.1_RP11-638I2.8	RP11-638I2.8	ENSG00000258666.1	0.000	0.000	-2.092	0.038
ENSG00000220771.2_RP3-322L4.2	RP3-322L4.2	ENSG00000220771.2	0.000	0.000	-2.092	0.038
ENSG00000200620.1_SNORA7	SNORA7	ENSG00000200620.1	0.000	0.000	-2.092	0.038
ENSG00000169933.8_FRMPD4	FRMPD4	ENSG00000169933.8	-0.233	0.112	-2.092	0.038

ENSG00000121871.3_SLITRK3	SLITRK3	ENSG00000121871.3	-0.187	0.090	-2.092	0.038
ENSG00000237414.1_RP11-321L2.2	RP11-321L2.2	ENSG00000237414.1	-0.020	0.009	-2.092	0.038
ENSG00000260747.1_RP11-421N8.1	RP11-421N8.1	ENSG00000260747.1	-0.131	0.063	-2.093	0.038
ENSG00000260412.1_RP11-438B23.2	RP11-438B23.2	ENSG00000260412.1	-0.087	0.041	-2.094	0.038
ENSG00000230448.1_LINC00276	LINC00276	ENSG00000230448.1	-0.014	0.007	-2.094	0.038
ENSG00000152642.6_GPD1L	GPD1L	ENSG00000152642.6	-0.591	0.282	-2.095	0.038
ENSG00000067842.13_ATP2B3	ATP2B3	ENSG00000067842.13	-0.674	0.322	-2.095	0.038
ENSG00000078018.15_MAP2	MAP2	ENSG00000078018.15	-4.539	2.167	-2.095	0.038
ENSG00000007237.12_GAS7	GAS7	ENSG00000007237.12	-2.342	1.118	-2.095	0.038
ENSG00000175581.9_MRPL48	MRPL48	ENSG00000175581.9	-0.906	0.432	-2.096	0.038
ENSG00000248876.1_RP11-231G15.3	RP11-231G15.3	ENSG00000248876.1	-0.017	0.008	-2.096	0.038
ENSG00000144406.12_UNC80	UNC80	ENSG00000144406.12	-0.366	0.174	-2.097	0.037
ENSG00000149313.6_AASDHPPT	AASDHPPT	ENSG00000149313.6	-1.179	0.562	-2.097	0.037
ENSG00000259834.1_RP11-284N8.3	RP11-284N8.3	ENSG00000259834.1	-0.206	0.098	-2.097	0.037
ENSG00000154822.11_PLCL2	PLCL2	ENSG00000154822.11	-0.258	0.123	-2.097	0.037
ENSG00000261546.1_CTD-2555A7.3	CTD-2555A7.3	ENSG00000261546.1	-0.004	0.002	-2.098	0.037
ENSG00000185304.12_RGPD2	RGPD2	ENSG00000185304.12	-0.146	0.069	-2.098	0.037
ENSG00000216915.2_RP1-97D16.1	RP1-97D16.1	ENSG00000216915.2	-0.016	0.008	-2.099	0.037
ENSG00000089682.12_RBM41	RBM41	ENSG00000089682.12	-0.147	0.070	-2.100	0.037
ENSG00000205810.4_KLRC3	KLRC3	ENSG00000205810.4	-0.139	0.066	-2.100	0.037
ENSG00000134258.12_VTCN1	VTCN1	ENSG00000134258.12	-0.006	0.003	-2.100	0.037
ENSG00000125337.12_KIF25	KIF25	ENSG00000125337.12	-0.456	0.217	-2.100	0.037
ENSG00000180316.7_PNPLA1	PNPLA1	ENSG00000180316.7	-0.009	0.005	-2.100	0.037
ENSG00000101445.4_PPP1R16B	PPP1R16B	ENSG00000101445.4	-1.410	0.671	-2.101	0.037
ENSG00000232388.1_LINC00493	LINC00493	ENSG00000232388.1	-2.435	1.159	-2.101	0.037
ENSG00000267242.1_AC069278.4	AC069278.4	ENSG00000267242.1	-0.109	0.052	-2.101	0.037
ENSG00000197721.12_CR1L	CR1L	ENSG00000197721.12	-0.023	0.011	-2.102	0.037
ENSG00000185158.8_LRRC37B	LRRC37B	ENSG00000185158.8	-0.568	0.270	-2.103	0.037
ENSG00000198040.6_ZNF84	ZNF84	ENSG00000198040.6	-0.460	0.219	-2.104	0.037
ENSG00000198780.7_FAM169A	FAM169A	ENSG00000198780.7	-0.332	0.158	-2.104	0.037
ENSG00000136810.8_TXN	TXN	ENSG00000136810.8	-2.975	1.414	-2.104	0.037
ENSG00000010818.4_HIVEP2	HIVEP2	ENSG00000010818.4	-0.470	0.223	-2.105	0.037
ENSG00000178235.6_SLITRK1	SLITRK1	ENSG00000178235.6	-0.304	0.144	-2.105	0.037
ENSG00000038274.11_MAT2B	MAT2B	ENSG00000038274.11	-1.097	0.521	-2.106	0.037
ENSG00000165152.4_TMEM246	TMEM246	ENSG00000165152.4	-0.985	0.468	-2.106	0.037
ENSG00000162852.9_CNST	CNST	ENSG00000162852.9	-0.527	0.250	-2.106	0.037
ENSG00000177992.9_FAM75E1	FAM75E1	ENSG00000177992.9	-0.004	0.002	-2.106	0.037
ENSG00000172772.3_OR10W1	OR10W1	ENSG00000172772.3	-0.001	0.000	-2.107	0.037
ENSG00000081803.11_CADPS2	CADPS2	ENSG00000081803.11	-1.272	0.604	-2.108	0.037
ENSG00000261829.1_RP11-223I10.1	RP11-223I10.1	ENSG00000261829.1	-1.951	0.925	-2.109	0.036
ENSG00000174529.6_TMEM81	TMEM81	ENSG00000174529.6	-0.448	0.212	-2.109	0.036
ENSG00000198920.5_KIAA0753	KIAA0753	ENSG00000198920.5	-0.192	0.091	-2.110	0.036
ENSG00000214288.4_HMGB3P13	HMGB3P13	ENSG00000214288.4	0.000	0.000	-2.113	0.036
ENSG00000259846.1_RP11-467L24.1	RP11-467L24.1	ENSG00000259846.1	-0.007	0.003	-2.113	0.036
ENSG00000112031.11_MTRF1L	MTRF1L	ENSG00000112031.11	-0.324	0.153	-2.114	0.036
ENSG00000136643.7_RPS6KC1	RPS6KC1	ENSG00000136643.7	-0.239	0.113	-2.114	0.036
ENSG00000233008.1_RP11-475O6.1	RP11-475O6.1	ENSG00000233008.1	-0.044	0.021	-2.114	0.036
ENSG00000047249.11_ATP6V1H	ATP6V1H	ENSG00000047249.11	-5.229	2.473	-2.114	0.036
ENSG00000239831.1_RNF7P1	RNF7P1	ENSG00000239831.1	-0.031	0.015	-2.115	0.036
ENSG00000115419.8_GLS	GLS	ENSG00000115419.8	-2.930	1.385	-2.115	0.036
ENSG00000205277.5_MUC12	MUC12	ENSG00000205277.5	-0.425	0.201	-2.116	0.036
ENSG00000248474.1_CTD-2292M14.1	CTD-2292M14.1	ENSG00000248474.1	-0.007	0.003	-2.116	0.036
ENSG00000178429.8_RPS3AP5	RPS3AP5	ENSG00000178429.8	-0.007	0.003	-2.118	0.036
ENSG00000111880.11_RNGTT	RNGTT	ENSG00000111880.11	-0.131	0.062	-2.119	0.036
ENSG00000144834.8_TAGLN3	TAGLN3	ENSG00000144834.8	-15.099	7.124	-2.119	0.036
ENSG00000232531.2_AC027612.1	AC027612.1	ENSG00000232531.2	-0.007	0.003	-2.119	0.036
ENSG00000166352.10_C11orf74	C11orf74	ENSG00000166352.10	-0.777	0.367	-2.119	0.036
ENSG00000116396.9_KCNC4	KCNC4	ENSG00000116396.9	-0.931	0.439	-2.120	0.035
ENSG00000185596.11_WASH3P	WASH3P	ENSG00000185596.11	-3.070	1.447	-2.121	0.035
ENSG00000188419.8_CHM	CHM	ENSG00000188419.8	-0.369	0.174	-2.121	0.035

ENSG00000224631.3_RP11-51O6.1	RP11-51O6.1	ENSG00000224631.3	-0.025	0.012	-2.122	0.035
ENSG00000134243.7_SORT1	SORT1	ENSG00000134243.7	-1.317	0.621	-2.122	0.035
ENSG00000145075.7_CCDC39	CCDC39	ENSG00000145075.7	-0.051	0.024	-2.123	0.035
ENSG00000253598.1_SLC10A5	SLC10A5	ENSG00000253598.1	-0.044	0.021	-2.123	0.035
ENSG00000070501.6_POLB	POLB	ENSG00000070501.6	-0.637	0.300	-2.123	0.035
ENSG00000111790.9_FGFR1OP2	FGFR1OP2	ENSG00000111790.9	-0.903	0.425	-2.123	0.035
ENSG00000254731.1_CTD-2005H7.1	CTD-2005H7.1	ENSG00000254731.1	-0.042	0.020	-2.123	0.035
ENSG00000015479.13_MATR3	MATR3	ENSG00000015479.13	-4.541	2.139	-2.123	0.035
ENSG00000249339.1_RP11-321E2.7	RP11-321E2.7	ENSG00000249339.1	-0.008	0.004	-2.123	0.035
ENSG00000248858.2_RP11-100L22.2	RP11-100L22.2	ENSG00000248858.2	-0.018	0.009	-2.123	0.035
ENSG00000163618.13_CADPS	CADPS	ENSG00000163618.13	-2.699	1.271	-2.123	0.035
ENSG00000141665.7_FBXO15	FBXO15	ENSG00000141665.7	-0.207	0.097	-2.123	0.035
ENSG00000172167.3_MTBP	MTBP	ENSG00000172167.3	-0.077	0.036	-2.125	0.035
ENSG00000129460.11_NGDN	NGDN	ENSG00000129460.11	-0.740	0.348	-2.125	0.035
ENSG00000054116.7_TRAPPC3	TRAPPC3	ENSG00000054116.7	-1.015	0.478	-2.125	0.035
ENSG00000179611.2_DGKZP1	DGKZP1	ENSG00000179611.2	-0.005	0.002	-2.125	0.035
ENSG00000235086.1_FNDC1-IT1	FNDC1-IT1	ENSG00000235086.1	-0.001	0.001	-2.126	0.035
ENSG00000242607.1_RPS3AP34	RPS3AP34	ENSG00000242607.1	-0.160	0.075	-2.127	0.035
ENSG00000135454.8_B4GALNT1	B4GALNT1	ENSG00000135454.8	-2.073	0.974	-2.128	0.035
ENSG00000241834.2_MetazoaSRP	RN7SL149P	ENSG00000241834.2	-0.066	0.031	-2.129	0.035
ENSG00000258785.1_CTD-2643K12.2	CTD-2643K12.2	ENSG00000258785.1	-0.015	0.007	-2.130	0.035
ENSG00000119048.3_UBE2B	UBE2B	ENSG00000119048.3	-2.570	1.206	-2.131	0.035
ENSG00000132932.11_ATP8A2	ATP8A2	ENSG00000132932.11	-0.533	0.250	-2.131	0.035
ENSG00000144034.10_TPRKB	TPRKB	ENSG00000144034.10	-0.450	0.211	-2.132	0.034
ENSG00000266878.1_RP11-1090M7.2	RP11-1090M7.2	ENSG00000266878.1	-0.054	0.025	-2.133	0.034
ENSG00000031003.6_FAM13B	FAM13B	ENSG00000031003.6	-0.406	0.190	-2.133	0.034
ENSG00000162981.12_FAM84A	FAM84A	ENSG00000162981.12	-0.991	0.465	-2.133	0.034
ENSG00000125675.13_GRIA3	GRIA3	ENSG00000125675.13	-1.309	0.614	-2.133	0.034
ENSG00000169744.8_LDB2	LDB2	ENSG00000169744.8	-1.831	0.858	-2.135	0.034
ENSG00000164440.10_TXLNB	TXLNB	ENSG00000164440.10	-0.032	0.015	-2.136	0.034
ENSG00000117155.11_SSX2IP	SSX2IP	ENSG00000117155.11	-0.489	0.229	-2.137	0.034
ENSG00000231720.1_RP11-568A7.3	RP11-568A7.3	ENSG00000231720.1	-0.005	0.002	-2.138	0.034
ENSG00000204466.5_DGKK	DGKK	ENSG00000204466.5	-0.004	0.002	-2.138	0.034
ENSG00000169519.14_METTL15	METTL15	ENSG00000169519.14	-0.118	0.055	-2.139	0.034
ENSG00000214189.2_ZNF788	ZNF788	ENSG00000214189.2	-0.065	0.030	-2.139	0.034
ENSG00000239572.1_RP11-451B8.1	RP11-451B8.1	ENSG00000239572.1	-0.019	0.009	-2.139	0.034
ENSG00000205323.4_SARNP	SARNP	ENSG00000205323.4	-1.098	0.513	-2.139	0.034
ENSG00000114279.8_FGF12	FGF12	ENSG00000114279.8	-1.123	0.525	-2.140	0.034
ENSG00000115977.13_AAK1	AAK1	ENSG00000115977.13	-6.920	3.234	-2.140	0.034
ENSG00000206652.1_RNU1-1	RNU1-1	ENSG00000206652.1	-1.403	0.655	-2.141	0.034
ENSG00000207005.1_RNU1-2	RNU1-2	ENSG00000207005.1	-1.403	0.655	-2.141	0.034
ENSG00000207389.1_RNU1-4	RNU1-4	ENSG00000207389.1	-1.403	0.655	-2.141	0.034
ENSG00000206737.1_RNU1-5	RNU1-5	ENSG00000206737.1	-1.403	0.655	-2.141	0.034
ENSG00000206596.1_RNU1-7	RNU1-7	ENSG00000206596.1	-1.403	0.655	-2.141	0.034
ENSG00000206588.1_RNU1-8	RNU1-8	ENSG00000206588.1	-1.403	0.655	-2.141	0.034
ENSG00000248518.1_RP11-231C18.2	RP11-231C18.2	ENSG00000248518.1	-0.024	0.011	-2.142	0.034
ENSG00000136888.6_ATP6V1G1	ATP6V1G1	ENSG00000136888.6	-1.736	0.810	-2.143	0.034
ENSG00000139910.15_NOVA1	NOVA1	ENSG00000139910.15	-2.050	0.956	-2.144	0.033
ENSG00000243648.1_RP11-215P8.1	RP11-215P8.1	ENSG00000243648.1	-0.028	0.013	-2.145	0.033
ENSG00000152409.8_JMY	JMY	ENSG00000152409.8	-0.119	0.056	-2.145	0.033
ENSG00000221886.3_C5orf54	C5orf54	ENSG00000221886.3	-0.100	0.047	-2.146	0.033
ENSG00000203616.2_RHOT1P2	RHOT1P2	ENSG00000203616.2	-0.147	0.069	-2.146	0.033
ENSG00000186288.4_PABPC1L2A	PABPC1L2A	ENSG00000186288.4	-0.380	0.177	-2.148	0.033
ENSG00000096092.5_TMEM14A	TMEM14A	ENSG00000096092.5	-2.076	0.967	-2.148	0.033
ENSG00000242525.1_OR7E100P	OR7E100P	ENSG00000242525.1	-0.012	0.006	-2.148	0.033
ENSG00000041357.11_PSMA4	PSMA4	ENSG00000041357.11	-1.940	0.903	-2.149	0.033
ENSG00000198626.9_RYR2	RYR2	ENSG00000198626.9	-0.598	0.278	-2.149	0.033
ENSG00000229994.1_RP4-765A10.1	RP4-765A10.1	ENSG00000229994.1	-0.154	0.071	-2.149	0.033
ENSG00000124535.10_WRNIP1	WRNIP1	ENSG00000124535.10	-0.626	0.291	-2.150	0.033
ENSG00000180066.5_C10orf91	C10orf91	ENSG00000180066.5	-0.013	0.006	-2.150	0.033

ENSG00000260415.1_RP11-923I11.1	RP11-923I11.1	ENSG00000260415.1	-1.341	0.624	-2.150	0.033
ENSG00000260395.1_CTD-2649C14.1	CTD-2649C14.1	ENSG00000260395.1	-0.100	0.047	-2.151	0.033
ENSG00000165300.5_SLITRK5	SLITRK5	ENSG00000165300.5	-0.408	0.190	-2.151	0.033
ENSG00000235997.2_AC109642.1	AC109642.1	ENSG00000235997.2	-0.006	0.003	-2.153	0.033
ENSG00000100320.17_RBFOX2	RBFOX2	ENSG00000100320.17	-1.377	0.640	-2.153	0.033
ENSG00000228643.1_AC079779.4	AC079779.4	ENSG00000228643.1	-0.028	0.013	-2.153	0.033
ENSG00000230453.3_ANKRD18B	ANKRD18B	ENSG00000230453.3	-1.026	0.476	-2.154	0.033
ENSG00000259594.1_CTD-2034I4.2	CTD-2034I4.2	ENSG00000259594.1	-0.021	0.010	-2.154	0.033
ENSG00000112290.8_WASF1	WASF1	ENSG00000112290.8	-3.254	1.510	-2.155	0.033
ENSG00000177030.11_DEAF1	DEAF1	ENSG00000177030.11	-3.813	1.768	-2.156	0.032
ENSG00000185774.9_KCNIP4	KCNIP4	ENSG00000185774.9	-1.308	0.607	-2.156	0.032
ENSG00000221823.5_PPP3R1	PPP3R1	ENSG00000221823.5	-4.816	2.233	-2.156	0.032
ENSG00000251970.1_U1	RNU1-41P	ENSG00000251970.1	-0.032	0.015	-2.158	0.032
ENSG00000252209.1_U1	RNU1-48P	ENSG00000252209.1	-0.032	0.015	-2.158	0.032
ENSG00000228262.2_AC073218.2	AC073218.2	ENSG00000228262.2	-0.016	0.007	-2.159	0.032
ENSG00000110851.7_PRDM4	PRDM4	ENSG00000110851.7	-0.242	0.112	-2.159	0.032
ENSG00000262294.1_RP11-1260E13.2	RP11-1260E13.2	ENSG00000262294.1	-0.006	0.003	-2.159	0.032
ENSG00000103121.4_CMC2	CMC2	ENSG00000103121.4	-1.497	0.693	-2.160	0.032
ENSG00000234477.1_AC004231.2	AC004231.2	ENSG00000234477.1	-0.031	0.015	-2.161	0.032
ENSG00000106299.6_WASL	WASL	ENSG00000106299.6	-0.712	0.329	-2.161	0.032
ENSG00000147400.8_CETN2	CETN2	ENSG00000147400.8	-0.956	0.442	-2.162	0.032
ENSG00000264729.1_RP11-219A15.2	RP11-219A15.2	ENSG00000264729.1	-0.015	0.007	-2.162	0.032
ENSG00000242661.1_RP11-113C12.1	RP11-113C12.1	ENSG00000242661.1	-0.045	0.021	-2.162	0.032
ENSG00000134758.9_RNF138	RNF138	ENSG00000134758.9	-0.129	0.059	-2.162	0.032
ENSG00000075292.12_ZNF638	ZNF638	ENSG00000075292.12	-2.488	1.151	-2.162	0.032
ENSG00000115844.6_DLX2	DLX2	ENSG00000115844.6	-0.086	0.040	-2.163	0.032
ENSG00000236508.1_ATP13A5-AS1	ATP13A5-AS1	ENSG00000236508.1	-0.037	0.017	-2.163	0.032
ENSG00000189157.8_FAM47E	FAM47E	ENSG00000189157.8	-0.575	0.266	-2.164	0.032
ENSG00000169676.4_DRD5	DRD5	ENSG00000169676.4	-0.120	0.055	-2.164	0.032
ENSG00000227887.1_RP11-459K23.1	RP11-459K23.1	ENSG00000227887.1	-0.017	0.008	-2.164	0.032
ENSG00000156219.12_ART3	ART3	ENSG00000156219.12	-0.421	0.194	-2.164	0.032
ENSG00000118402.5_ELOVL4	ELOVL4	ENSG00000118402.5	-0.522	0.241	-2.166	0.032
ENSG00000148965.4_SAA4	SAA4	ENSG00000148965.4	-0.032	0.015	-2.166	0.032
ENSG00000235482.1_AC093415.4	AC093415.4	ENSG00000235482.1	-0.058	0.027	-2.166	0.032
ENSG00000235113.1_RP11-241I20.4	RP11-241I20.4	ENSG00000235113.1	-0.003	0.001	-2.166	0.032
ENSG00000144285.10_SCN1A	SCN1A	ENSG00000144285.10	-0.675	0.311	-2.167	0.032
ENSG00000248939.1_RP11-395F4.1	RP11-395F4.1	ENSG00000248939.1	-0.001	0.000	-2.168	0.032
ENSG00000176406.16_RIMS2	RIMS2	ENSG00000176406.16	-1.011	0.466	-2.168	0.032
ENSG00000225015.1_SNX18P15	SNX18P15	ENSG00000225015.1	-0.027	0.012	-2.169	0.031
ENSG00000186930.3_KRTAP6-2	KRTAP6-2	ENSG00000186930.3	-0.002	0.001	-2.169	0.031
ENSG00000185760.11_KCNQ5	KCNQ5	ENSG00000185760.11	-0.784	0.361	-2.169	0.031
ENSG00000234553.1_AC022431.3	AC022431.3	ENSG00000234553.1	-0.015	0.007	-2.169	0.031
ENSG00000103723.8_AP3B2	AP3B2	ENSG00000103723.8	-3.533	1.628	-2.170	0.031
ENSG00000260686.1_CTB-36H16.2	CTB-36H16.2	ENSG00000260686.1	-0.123	0.057	-2.172	0.031
ENSG00000146147.10_MLIP	MLIP	ENSG00000146147.10	-0.335	0.154	-2.173	0.031
ENSG00000122126.10_OCRL	OCRL	ENSG00000122126.10	-0.397	0.183	-2.173	0.031
ENSG00000134986.9_NREP	NREP	ENSG00000134986.9	-2.388	1.099	-2.174	0.031
ENSG00000253183.1_RP4-701O16.5	RP4-701O16.5	ENSG00000253183.1	-0.009	0.004	-2.174	0.031
ENSG00000122550.12_KLHL7	KLHL7	ENSG00000122550.12	-0.396	0.182	-2.174	0.031
ENSG00000215120.2_AL590763.5	AL590763.5	ENSG00000215120.2	-0.003	0.001	-2.175	0.031
ENSG00000119844.10_AFTPH	AFTPH	ENSG00000119844.10	-0.582	0.267	-2.176	0.031
ENSG00000021645.13_NRXN3	NRXN3	ENSG00000021645.13	-1.553	0.713	-2.177	0.031
ENSG00000124140.7_SLC12A5	SLC12A5	ENSG00000124140.7	-3.036	1.395	-2.177	0.031
ENSG00000228941.1_UBE3AP2	UBE3AP2	ENSG00000228941.1	-0.005	0.002	-2.178	0.031
ENSG00000256533.1_RP11-212D19.5	RP11-212D19.5	ENSG00000256533.1	-0.071	0.032	-2.178	0.031
ENSG00000106443.10_PHF14	PHF14	ENSG00000106443.10	-0.904	0.415	-2.179	0.031
ENSG00000239840.1_RPL23AP72	RPL23AP72	ENSG00000239840.1	-0.025	0.011	-2.179	0.031
ENSG00000125354.18_41888	41888	ENSG00000125354.18	-0.605	0.278	-2.179	0.031
ENSG00000224080.1_AC126118.1	AC126118.1	ENSG00000224080.1	-0.044	0.020	-2.181	0.031
ENSG00000065609.9_SNAP91	SNAP91	ENSG00000065609.9	-3.182	1.459	-2.181	0.031

ENSG00000187189.9_TSPYL4	TSPYL4	ENSG00000187189.9	-2.549	1.168	-2.183	0.030
ENSG00000214832.4_UPF3AP2	UPF3AP2	ENSG00000214832.4	-0.047	0.022	-2.183	0.030
ENSG00000165349.7_SLC7A3	SLC7A3	ENSG00000165349.7	-0.072	0.033	-2.184	0.030
ENSG00000261254.1_RP4-714D9.5	RP4-714D9.5	ENSG00000261254.1	-0.046	0.021	-2.184	0.030
ENSG00000095203.9_EPB41L4B	EPB41L4B	ENSG00000095203.9	-0.341	0.156	-2.184	0.030
ENSG00000164845.12_FAM86FP	FAM86FP	ENSG00000164845.12	-0.019	0.009	-2.185	0.030
ENSG00000145526.6_CDH18	CDH18	ENSG00000145526.6	-0.956	0.438	-2.185	0.030
ENSG00000118420.12_UBE3D	UBE3D	ENSG00000118420.12	-0.221	0.101	-2.186	0.030
ENSG00000165914.10_TTC7B	TTC7B	ENSG00000165914.10	-1.339	0.612	-2.187	0.030
ENSG00000197969.6_VPS13A	VPS13A	ENSG00000197969.6	-0.253	0.116	-2.188	0.030
ENSG00000108379.5_WNT3	WNT3	ENSG00000108379.5	-1.823	0.833	-2.189	0.030
ENSG00000235454.1_RP11-678B3.2	RP11-678B3.2	ENSG00000235454.1	-0.013	0.006	-2.190	0.030
ENSG00000228649.3_AC005682.5	AC005682.5	ENSG00000228649.3	-0.022	0.010	-2.190	0.030
ENSG00000213760.5_ATP6V1G2	ATP6V1G2	ENSG00000213760.5	-9.479	4.327	-2.191	0.030
ENSG00000205745.2_AC083864.4	AC083864.4	ENSG00000205745.2	-0.004	0.002	-2.191	0.030
ENSG00000261597.1_RP11-353B9.1	RP11-353B9.1	ENSG00000261597.1	-0.140	0.064	-2.191	0.030
ENSG00000149256.10_ODZ4	ODZ4	ENSG00000149256.10	-0.152	0.069	-2.191	0.030
ENSG00000152128.13_TMEM163	TMEM163	ENSG00000152128.13	-0.410	0.187	-2.191	0.030
ENSG00000151320.6_AKAP6	AKAP6	ENSG00000151320.6	-0.615	0.281	-2.191	0.030
ENSG00000077327.9_SPAG6	SPAG6	ENSG00000077327.9	-0.073	0.033	-2.192	0.030
ENSG00000247570.2_RP11-449M6.1	RP11-449M6.1	ENSG00000247570.2	-0.019	0.008	-2.192	0.030
ENSG00000261554.1_RP11-810K23.10	RP11-810K23.10	ENSG00000261554.1	0.000	0.000	-2.193	0.030
ENSG00000249853.3_HS3ST5	HS3ST5	ENSG00000249853.3	-0.099	0.045	-2.194	0.030
ENSG00000121297.6_TSHZ3	TSHZ3	ENSG00000121297.6	-0.181	0.083	-2.195	0.029
ENSG00000237429.1_RP1-159A19.4	RP1-159A19.4	ENSG00000237429.1	-0.073	0.033	-2.196	0.029
ENSG00000181378.9_CCDC108	CCDC108	ENSG00000181378.9	-0.064	0.029	-2.196	0.029
ENSG00000166793.6_YPEL4	YPEL4	ENSG00000166793.6	-1.612	0.733	-2.199	0.029
ENSG00000128951.8_DUT	DUT	ENSG00000128951.8	-1.575	0.716	-2.199	0.029
ENSG00000261129.1_RP11-361D14.2	RP11-361D14.2	ENSG00000261129.1	0.000	0.000	-2.201	0.029
ENSG00000263821.1_RP11-527H14.1	RP11-527H14.1	ENSG00000263821.1	-0.003	0.001	-2.202	0.029
ENSG00000237466.1_RP4-736H5.2	RP4-736H5.2	ENSG00000237466.1	-0.003	0.001	-2.203	0.029
ENSG00000106536.13_POU6F2	POU6F2	ENSG00000106536.13	-0.101	0.046	-2.204	0.029
ENSG00000122574.6_WIPF3	WIPF3	ENSG00000122574.6	-0.872	0.396	-2.205	0.029
ENSG00000179988.8_PSTK	PSTK	ENSG00000179988.8	-0.297	0.135	-2.205	0.029
ENSG00000112208.8_BAG2	BAG2	ENSG00000112208.8	-0.190	0.086	-2.205	0.029
ENSG00000154265.11_ABCA5	ABCA5	ENSG00000154265.11	-0.563	0.255	-2.206	0.029
ENSG00000185554.10_NXF2	NXF2	ENSG00000185554.10	-0.024	0.011	-2.206	0.029
ENSG00000247157.2_RP11-434C1.1	RP11-434C1.1	ENSG00000247157.2	-0.286	0.130	-2.206	0.029
ENSG00000214376.5_VSTM5	VSTM5	ENSG00000214376.5	-0.156	0.071	-2.207	0.029
ENSG00000189367.10_KIAA0408	KIAA0408	ENSG00000189367.10	-0.903	0.409	-2.208	0.029
ENSG00000116977.14_LGALS8	LGALS8	ENSG00000116977.14	-0.587	0.266	-2.209	0.029
ENSG00000146938.10_NLGN4X	NLGN4X	ENSG00000146938.10	-0.211	0.096	-2.209	0.029
ENSG00000249114.1_RP11-5N11.3	RP11-5N11.3	ENSG00000249114.1	-0.061	0.028	-2.209	0.029
ENSG00000132466.13_ANKRD17	ANKRD17	ENSG00000132466.13	-2.325	1.052	-2.210	0.028
ENSG00000175766.7_EIF4E1B	EIF4E1B	ENSG00000175766.7	-0.355	0.161	-2.211	0.028
ENSG00000239470.2_RP11-16F15.2	RP11-16F15.2	ENSG00000239470.2	-0.052	0.023	-2.212	0.028
ENSG00000129159.6_KCNC1	KCNC1	ENSG00000129159.6	-2.338	1.057	-2.212	0.028
ENSG00000072041.11_SLC6A15	SLC6A15	ENSG00000072041.11	-0.469	0.212	-2.213	0.028
ENSG00000174450.6_GOLGA6L2	GOLGA6L2	ENSG00000174450.6	-0.005	0.002	-2.213	0.028
ENSG00000166862.6_CACNG2	CACNG2	ENSG00000166862.6	-0.887	0.401	-2.213	0.028
ENSG00000180964.12_TCEAL8	TCEAL8	ENSG00000180964.12	-1.372	0.619	-2.215	0.028
ENSG00000156411.5_C14orf2	C14orf2	ENSG00000156411.5	-7.455	3.362	-2.218	0.028
ENSG00000138382.9_METTL5	METTL5	ENSG00000138382.9	-0.862	0.388	-2.220	0.028
ENSG00000156103.11_MMP16	MMP16	ENSG00000156103.11	-0.095	0.043	-2.221	0.028
ENSG00000259796.1_RP11-355I22.7	RP11-355I22.7	ENSG00000259796.1	-1.161	0.522	-2.221	0.028
ENSG00000158985.9_CDC42SE2	CDC42SE2	ENSG00000158985.9	-0.878	0.395	-2.222	0.028
ENSG00000001460.13_STPG1	STPG1	ENSG00000001460.13	-0.339	0.152	-2.223	0.028
ENSG00000164346.5_NSA2	NSA2	ENSG00000164346.5	-2.026	0.911	-2.224	0.027
ENSG00000175334.3_BANF1	BANF1	ENSG00000175334.3	-4.119	1.852	-2.224	0.027
ENSG00000229323.1_RP11-175B12.2	RP11-175B12.2	ENSG00000229323.1	-0.032	0.015	-2.224	0.027

ENSG00000163393.8_SLC22A15	SLC22A15	ENSG00000163393.8	-0.137	0.061	-2.225	0.027
ENSG00000239525.1_RP11-722P15.1	RP11-722P15.1	ENSG00000239525.1	-0.023	0.010	-2.225	0.027
ENSG00000185518.7_SV2B	SV2B	ENSG00000185518.7	-1.750	0.786	-2.226	0.027
ENSG00000267766.1_RP11-299P2.1	RP11-299P2.1	ENSG00000267766.1	-0.091	0.041	-2.227	0.027
ENSG00000171150.7_SOCS5	SOCS5	ENSG00000171150.7	-0.206	0.093	-2.228	0.027
ENSG00000136352.13_NKX2-1	NKX2-1	ENSG00000136352.13	-0.020	0.009	-2.228	0.027
ENSG00000164091.7_WDR82	WDR82	ENSG00000164091.7	-0.796	0.357	-2.229	0.027
ENSG00000185652.6_NTF3	NTF3	ENSG00000185652.6	-0.016	0.007	-2.229	0.027
ENSG00000176095.7_IP6K1	IP6K1	ENSG00000176095.7	-1.051	0.471	-2.229	0.027
ENSG00000144290.9_SLC4A10	SLC4A10	ENSG00000144290.9	-0.883	0.396	-2.229	0.027
ENSG00000229184.2_RP11-255J3.3	RP11-255J3.3	ENSG00000229184.2	-0.024	0.011	-2.231	0.027
ENSG00000164609.5_SLU7	SLU7	ENSG00000164609.5	-2.636	1.181	-2.231	0.027
ENSG00000102104.8_RS1	RS1	ENSG00000102104.8	-0.012	0.005	-2.231	0.027
ENSG00000111653.15_ING4	ING4	ENSG00000111653.15	-0.708	0.317	-2.233	0.027
ENSG00000103512.10_NOMO1	NOMO1	ENSG00000103512.10	-2.105	0.943	-2.234	0.027
ENSG00000173930.8_SLCO4C1	SLCO4C1	ENSG00000173930.8	-0.024	0.011	-2.235	0.027
ENSG00000185904.6_RP11-178A10.1	RP11-178A10.1	ENSG00000185904.6	-0.251	0.112	-2.235	0.027
ENSG00000118276.7_B4GALT6	B4GALT6	ENSG00000118276.7	-0.495	0.221	-2.236	0.027
ENSG00000214842.5_RAD51AP2	RAD51AP2	ENSG00000214842.5	-0.026	0.012	-2.237	0.027
ENSG00000132294.9_EFR3A	EFR3A	ENSG00000132294.9	-0.964	0.431	-2.238	0.027
ENSG00000151150.14_ANK3	ANK3	ENSG00000151150.14	-1.123	0.502	-2.238	0.027
ENSG00000040731.6_CDH10	CDH10	ENSG00000040731.6	-0.621	0.277	-2.238	0.026
ENSG00000249550.2_RP11-438N16.1	RP11-438N16.1	ENSG00000249550.2	-0.129	0.058	-2.239	0.026
ENSG00000078140.9_UBE2K	UBE2K	ENSG00000078140.9	-0.703	0.314	-2.239	0.026
ENSG00000070718.7_AP3M2	AP3M2	ENSG00000070718.7	-0.769	0.343	-2.239	0.026
ENSG00000115840.9_SLC25A12	SLC25A12	ENSG00000115840.9	-1.344	0.600	-2.240	0.026
ENSG00000249616.1_CTD-2194F12.1	CTD-2194F12.1	ENSG00000249616.1	-0.011	0.005	-2.241	0.026
ENSG00000146433.8_TMEM181	TMEM181	ENSG00000146433.8	-0.297	0.132	-2.242	0.026
ENSG00000182768.7_NGRN	NGRN	ENSG00000182768.7	-8.831	3.937	-2.243	0.026
ENSG00000145934.11_ODZ2	ODZ2	ENSG00000145934.11	-0.548	0.244	-2.244	0.026
ENSG00000257346.1_RP11-386G11.8	RP11-386G11.8	ENSG00000257346.1	-0.004	0.002	-2.245	0.026
ENSG00000091972.13_CD200	CD200	ENSG00000091972.13	-1.595	0.710	-2.245	0.026
ENSG00000173175.10_ADCY5	ADCY5	ENSG00000173175.10	-0.509	0.226	-2.246	0.026
ENSG00000204856.7_FAM216A	FAM216A	ENSG00000204856.7	-1.012	0.450	-2.247	0.026
ENSG00000241678.1_RP11-732A19.1	RP11-732A19.1	ENSG00000241678.1	-0.104	0.046	-2.247	0.026
ENSG00000113595.10_TRIM23	TRIM23	ENSG00000113595.10	-0.402	0.179	-2.247	0.026
ENSG00000249487.2_RP11-97O12.2	RP11-97O12.2	ENSG00000249487.2	-0.030	0.014	-2.247	0.026
ENSG00000185950.7_IRS2	IRS2	ENSG00000185950.7	-0.811	0.361	-2.247	0.026
ENSG00000069329.11_VPS35	VPS35	ENSG00000069329.11	-1.168	0.520	-2.247	0.026
ENSG00000085365.12_SCAMP1	SCAMP1	ENSG00000085365.12	-0.633	0.282	-2.248	0.026
ENSG00000114757.14_PEX5L	PEX5L	ENSG00000114757.14	-0.631	0.281	-2.249	0.026
ENSG00000169047.4_IRS1	IRS1	ENSG00000169047.4	-0.148	0.066	-2.249	0.026
ENSG00000248491.1_RP11-125O18.1	RP11-125O18.1	ENSG00000248491.1	-0.006	0.003	-2.250	0.026
ENSG00000220685.2_RP11-530A18.1	RP11-530A18.1	ENSG00000220685.2	-0.038	0.017	-2.251	0.026
ENSG00000196542.4_SPTSSB	SPTSSB	ENSG00000196542.4	-0.370	0.164	-2.251	0.026
ENSG00000165023.5_DIRAS2	DIRAS2	ENSG00000165023.5	-2.062	0.916	-2.252	0.026
ENSG00000161048.7_NAPEPLD	NAPEPLD	ENSG00000161048.7	-0.466	0.207	-2.252	0.026
ENSG00000182923.11_CEP63	CEP63	ENSG00000182923.11	-0.362	0.161	-2.253	0.026
ENSG00000138439.10_FAM117B	FAM117B	ENSG00000138439.10	-0.159	0.071	-2.253	0.026
ENSG00000225218.1_AP001628.6	AP001628.6	ENSG00000225218.1	-0.093	0.041	-2.255	0.025
ENSG00000261390.1_RP11-345M22.2	RP11-345M22.2	ENSG00000261390.1	-0.022	0.010	-2.255	0.025
ENSG00000079102.12_RUNX1T1	RUNX1T1	ENSG00000079102.12	-0.347	0.154	-2.256	0.025
ENSG00000138801.4_PAPSS1	PAPSS1	ENSG00000138801.4	-0.834	0.370	-2.256	0.025
ENSG00000212766.4_LINC00277	LINC00277	ENSG00000212766.4	-0.049	0.022	-2.256	0.025
ENSG00000119866.16_BCL11A	BCL11A	ENSG00000119866.16	-1.454	0.644	-2.257	0.025
ENSG00000107018.5_RLN1	RLN1	ENSG00000107018.5	-0.015	0.007	-2.257	0.025
ENSG00000188095.3_MESP2	MESP2	ENSG00000188095.3	-0.089	0.039	-2.258	0.025
ENSG00000227706.2_AL713998.1	AL713998.1	ENSG00000227706.2	0.000	0.000	-2.258	0.025
ENSG00000240771.2_ARHGEF25	ARHGEF25	ENSG00000240771.2	-4.463	1.976	-2.259	0.025
ENSG00000120256.4_LRP11	LRP11	ENSG00000120256.4	-0.770	0.341	-2.260	0.025

ENSG00000115350.7_POLE4	POLE4	ENSG00000115350.7	-1.158	0.513	-2.260	0.025
ENSG00000122034.8_GTF3A	GTF3A	ENSG00000122034.8	-2.254	0.997	-2.261	0.025
ENSG00000159164.5_SV2A	SV2A	ENSG00000159164.5	-4.367	1.930	-2.263	0.025
ENSG00000232474.1_NCKAP5-IT1	NCKAP5-IT1	ENSG00000232474.1	-0.018	0.008	-2.265	0.025
ENSG00000262492.1_RP11-1099M24.8	RP11-1099M24.8	ENSG00000262492.1	-0.047	0.021	-2.265	0.025
ENSG00000233002.2_AC005324.6	AC005324.6	ENSG00000233002.2	-0.026	0.012	-2.265	0.025
ENSG00000238793.1_SNORD124	SNORD124	ENSG00000238793.1	-0.003	0.001	-2.265	0.025
ENSG00000258919.1_RP11-1029J19.4	RP11-1029J19.4	ENSG00000258919.1	-0.062	0.027	-2.265	0.025
ENSG00000238820.1_HAR1A	HAR1A	ENSG00000238820.1	-0.027	0.012	-2.265	0.025
ENSG00000124207.11_CSE1L	CSE1L	ENSG00000124207.11	-0.645	0.284	-2.266	0.025
ENSG00000234485.4_AC007881.5	AC007881.5	ENSG00000234485.4	-0.031	0.014	-2.268	0.025
ENSG00000109270.8_LAMTOR3	LAMTOR3	ENSG00000109270.8	-0.504	0.222	-2.268	0.025
ENSG00000265579.1_RP11-713C5.1	RP11-713C5.1	ENSG00000265579.1	-0.641	0.283	-2.268	0.025
ENSG00000215148.3_PRSS41	PRSS41	ENSG00000215148.3	-0.001	0.000	-2.269	0.025
ENSG00000198963.5_RORB	RORB	ENSG00000198963.5	-0.264	0.116	-2.270	0.024
ENSG00000246273.2_SBF2-AS1	SBF2-AS1	ENSG00000246273.2	-0.110	0.048	-2.270	0.024
ENSG00000196243.4_LINC00615	LINC00615	ENSG00000196243.4	-0.001	0.001	-2.271	0.024
ENSG00000265139.1_RP11-227G15.2	RP11-227G15.2	ENSG00000265139.1	-0.039	0.017	-2.272	0.024
ENSG00000114391.8_RPL24	RPL24	ENSG00000114391.8	-14.374	6.325	-2.272	0.024
ENSG00000115592.7_PRKAG3	PRKAG3	ENSG00000115592.7	-0.005	0.002	-2.273	0.024
ENSG00000184517.7_ZFP1	ZFP1	ENSG00000184517.7	-0.273	0.120	-2.273	0.024
ENSG00000260391.1_RP11-71H17.7	RP11-71H17.7	ENSG00000260391.1	-0.597	0.262	-2.275	0.024
ENSG00000174469.12_CNTNAP2	CNTNAP2	ENSG00000174469.12	-1.312	0.576	-2.276	0.024
ENSG00000134049.3_IER3IP1	IER3IP1	ENSG00000134049.3	-0.451	0.198	-2.277	0.024
ENSG00000170502.8_NUDT9	NUDT9	ENSG00000170502.8	-0.821	0.361	-2.277	0.024
ENSG00000124191.12_TOX2	TOX2	ENSG00000124191.12	-0.698	0.307	-2.278	0.024
ENSG00000104804.3_TULP2	TULP2	ENSG00000104804.3	-0.100	0.044	-2.278	0.024
ENSG00000189227.4_C15orf61	C15orf61	ENSG00000189227.4	-0.525	0.230	-2.278	0.024
ENSG00000101098.7_RIMS4	RIMS4	ENSG00000101098.7	-1.142	0.501	-2.279	0.024
ENSG00000260432.1_RP11-297M9.2	RP11-297M9.2	ENSG00000260432.1	-0.804	0.353	-2.280	0.024
ENSG00000118689.10_FOXO3	FOXO3	ENSG00000118689.10	-0.473	0.207	-2.280	0.024
ENSG00000164031.12_DNAJB14	DNAJB14	ENSG00000164031.12	-0.348	0.153	-2.281	0.024
ENSG00000101489.12_CELF4	CELF4	ENSG00000101489.12	-6.799	2.981	-2.281	0.024
ENSG00000250159.1_RP11-381K20.2	RP11-381K20.2	ENSG00000250159.1	-0.128	0.056	-2.281	0.024
ENSG00000101350.6_KIF3B	KIF3B	ENSG00000101350.6	-0.631	0.277	-2.281	0.024
ENSG00000225214.1_AC079790.2	AC079790.2	ENSG00000225214.1	-0.036	0.016	-2.282	0.024
ENSG00000166206.8_GABRB3	GABRB3	ENSG00000166206.8	-1.023	0.448	-2.284	0.024
ENSG00000175893.6_ZDHHC21	ZDHHC21	ENSG00000175893.6	-0.186	0.081	-2.286	0.023
ENSG00000198399.9_ITSN2	ITSN2	ENSG00000198399.9	-0.539	0.236	-2.288	0.023
ENSG00000147036.7_LANCL3	LANCL3	ENSG00000147036.7	-0.034	0.015	-2.289	0.023
ENSG00000231699.1_AC020550.7	AC020550.7	ENSG00000231699.1	-0.023	0.010	-2.289	0.023
ENSG00000257008.2_GPR142	GPR142	ENSG00000257008.2	-0.013	0.006	-2.289	0.023
ENSG00000101349.12_PAK7	PAK7	ENSG00000101349.12	-0.393	0.172	-2.290	0.023
ENSG00000170855.3_TRIAP1	TRIAP1	ENSG00000170855.3	-0.364	0.159	-2.290	0.023
ENSG00000256625.1_RP11-582E3.4	RP11-582E3.4	ENSG00000256625.1	-0.022	0.010	-2.291	0.023
ENSG00000170426.1_SDR9C7	SDR9C7	ENSG00000170426.1	-0.017	0.007	-2.292	0.023
ENSG00000139914.6_FITM1	FITM1	ENSG00000139914.6	-0.115	0.050	-2.292	0.023
ENSG00000225135.1_RP11-361F15.2	RP11-361F15.2	ENSG00000225135.1	-0.687	0.300	-2.293	0.023
ENSG00000205981.2_DNAJC19	DNAJC19	ENSG00000205981.2	-0.785	0.342	-2.294	0.023
ENSG00000227053.1_RP11-395B7.4	RP11-395B7.4	ENSG00000227053.1	-1.991	0.867	-2.296	0.023
ENSG00000226627.1_SHANK2-AS1	SHANK2-AS1	ENSG00000226627.1	-0.061	0.027	-2.298	0.023
ENSG00000204564.7_C6orf136	C6orf136	ENSG00000204564.7	-2.375	1.033	-2.298	0.023
ENSG00000169618.4_PROKR1	PROKR1	ENSG00000169618.4	-0.003	0.001	-2.298	0.023
ENSG00000226592.1_AC004911.2	AC004911.2	ENSG00000226592.1	-0.015	0.007	-2.299	0.023
ENSG00000187672.8_ERC2	ERC2	ENSG00000187672.8	-0.555	0.241	-2.300	0.023
ENSG00000250033.1_SLC7A11-AS1	SLC7A11-AS1	ENSG00000250033.1	-0.004	0.002	-2.301	0.023
ENSG00000184408.5_KCND2	KCND2	ENSG00000184408.5	-0.237	0.103	-2.301	0.023
ENSG00000054277.8_OPN3	OPN3	ENSG00000054277.8	-0.806	0.350	-2.302	0.023
ENSG00000239705.1_RP11-65N13.8	RP11-65N13.8	ENSG00000239705.1	-0.020	0.009	-2.304	0.022
ENSG00000171817.12_ZNF540	ZNF540	ENSG00000171817.12	-0.386	0.167	-2.305	0.022

ENSG00000178567.5_EPM2AIP1	EPM2AIP1	ENSG00000178567.5	-0.606	0.263	-2.305	0.022
ENSG00000241475.1_RP4-781K5.5	RP4-781K5.5	ENSG00000241475.1	-0.001	0.001	-2.306	0.022
ENSG00000256579.1_AC135776.1	AC135776.1	ENSG00000256579.1	-0.062	0.027	-2.307	0.022
ENSG00000254893.2_RP11-466P24.2	RP11-466P24.2	ENSG00000254893.2	-0.020	0.009	-2.308	0.022
ENSG00000254051.1_RP11-403D15.1	RP11-403D15.1	ENSG00000254051.1	-0.036	0.016	-2.309	0.022
ENSG00000155966.9_AFF2	AFF2	ENSG00000155966.9	-0.086	0.037	-2.311	0.022
ENSG00000262700.1_RP11-266L9.3	RP11-266L9.3	ENSG00000262700.1	-0.056	0.024	-2.311	0.022
ENSG00000254781.1_GVINP2	GVINP2	ENSG00000254781.1	-0.009	0.004	-2.312	0.022
ENSG00000244567.1_AC096772.6	AC096772.6	ENSG00000244567.1	-0.564	0.244	-2.314	0.022
ENSG00000258274.1_RP11-887P2.5	RP11-887P2.5	ENSG00000258274.1	-0.012	0.005	-2.315	0.022
ENSG00000235100.2_RP11-80H5.4	RP11-80H5.4	ENSG00000235100.2	-0.027	0.012	-2.315	0.022
ENSG00000261675.1_RP5-1119A7.17	RP5-1119A7.17	ENSG00000261675.1	-1.115	0.481	-2.316	0.022
ENSG00000224917.1_AC016694.2	AC016694.2	ENSG00000224917.1	0.000	0.000	-2.317	0.022
ENSG00000254707.1_RP11-35J10.4	RP11-35J10.4	ENSG00000254707.1	-0.014	0.006	-2.317	0.022
ENSG00000124383.3_MPHOSPH10	MPHOSPH10	ENSG00000124383.3	-0.658	0.284	-2.318	0.022
ENSG00000140006.7_WDR89	WDR89	ENSG00000140006.7	-0.100	0.043	-2.318	0.022
ENSG00000075213.5_SEMA3A	SEMA3A	ENSG00000075213.5	-0.084	0.036	-2.320	0.022
ENSG00000169085.6_C8orf46	C8orf46	ENSG00000169085.6	-1.420	0.612	-2.321	0.021
ENSG00000254191.1_KB-1552D7.2	KB-1552D7.2	ENSG00000254191.1	-0.041	0.018	-2.321	0.021
ENSG00000185716.7_C16orf52	C16orf52	ENSG00000185716.7	-0.560	0.241	-2.321	0.021
ENSG00000247675.2_LRP4-AS1	LRP4-AS1	ENSG00000247675.2	-0.057	0.024	-2.322	0.021
ENSG00000255733.1_IFNG-AS1	IFNG-AS1	ENSG00000255733.1	-0.011	0.005	-2.322	0.021
ENSG00000181744.4_C3orf58	C3orf58	ENSG00000181744.4	-0.259	0.112	-2.322	0.021
ENSG00000135968.14_GCC2	GCC2	ENSG00000135968.14	-1.036	0.446	-2.323	0.021
ENSG00000224209.1_LINC00466	LINC00466	ENSG00000224209.1	-0.015	0.006	-2.323	0.021
ENSG00000198105.7_ZNF248	ZNF248	ENSG00000198105.7	-0.258	0.111	-2.323	0.021
ENSG00000128881.12_TTBK2	TTBK2	ENSG00000128881.12	-0.386	0.166	-2.324	0.021
ENSG00000182463.7_TSHZ2	TSHZ2	ENSG00000182463.7	-0.342	0.147	-2.324	0.021
ENSG00000170396.6_ZNF804A	ZNF804A	ENSG00000170396.6	-0.147	0.063	-2.325	0.021
ENSG00000128045.5_RASL11B	RASL11B	ENSG00000128045.5	-0.688	0.296	-2.325	0.021
ENSG00000152433.9_ZNF547	ZNF547	ENSG00000152433.9	-0.128	0.055	-2.326	0.021
ENSG00000176204.9_LRRTM4	LRRTM4	ENSG00000176204.9	-0.303	0.130	-2.327	0.021
ENSG00000229349.2_ACTG1P9	ACTG1P9	ENSG00000229349.2	-0.004	0.002	-2.328	0.021
ENSG00000150086.3_GRIN2B	GRIN2B	ENSG00000150086.3	-0.052	0.022	-2.331	0.021
ENSG00000172586.7_CHCHD1	CHCHD1	ENSG00000172586.7	-0.927	0.397	-2.333	0.021
ENSG00000213060.3_RP4-612B18.3	RP4-612B18.3	ENSG00000213060.3	-0.061	0.026	-2.333	0.021
ENSG00000131504.11_DIAPH1	DIAPH1	ENSG00000131504.11	-0.595	0.255	-2.335	0.021
ENSG00000235828.4_AC005550.6	AC005550.6	ENSG00000235828.4	-0.157	0.067	-2.336	0.021
ENSG00000237651.2_C2orf74	C2orf74	ENSG00000237651.2	-2.049	0.877	-2.337	0.021
ENSG00000100600.10_LGMN	LGMN	ENSG00000100600.10	-1.161	0.497	-2.337	0.021
ENSG00000085224.15_ATRX	ATRX	ENSG00000085224.15	-1.072	0.459	-2.338	0.021
ENSG00000226203.1_RP4-760C5.5	RP4-760C5.5	ENSG00000226203.1	-0.004	0.002	-2.338	0.021
ENSG00000061918.8_GUCY1B3	GUCY1B3	ENSG00000061918.8	-1.747	0.747	-2.338	0.021
ENSG00000160345.8_C9orf116	C9orf116	ENSG00000160345.8	-0.418	0.179	-2.340	0.020
ENSG00000263938.1_RP11-579O24.3	RP11-579O24.3	ENSG00000263938.1	-0.111	0.047	-2.342	0.020
ENSG00000108924.7_HLF	HLF	ENSG00000108924.7	-1.382	0.590	-2.342	0.020
ENSG00000163630.6_SYNPR	SYNPR	ENSG00000163630.6	-2.411	1.029	-2.343	0.020
ENSG00000231704.1_AC004895.4	AC004895.4	ENSG00000231704.1	-0.019	0.008	-2.343	0.020
ENSG00000125870.6_SNRPB2	SNRPB2	ENSG00000125870.6	-1.028	0.439	-2.345	0.020
ENSG00000225163.3_LINC00618	LINC00618	ENSG00000225163.3	-0.077	0.033	-2.345	0.020
ENSG00000077152.5_UBE2T	UBE2T	ENSG00000077152.5	-0.540	0.230	-2.345	0.020
ENSG00000156687.6_UNC5D	UNC5D	ENSG00000156687.6	-0.159	0.068	-2.346	0.020
ENSG00000073067.9_CYP2W1	CYP2W1	ENSG00000073067.9	-0.011	0.005	-2.346	0.020
ENSG00000234590.1_GNG5P5	GNG5P5	ENSG00000234590.1	-0.004	0.002	-2.347	0.020
ENSG00000164164.11_OTUD4	OTUD4	ENSG00000164164.11	-0.255	0.109	-2.347	0.020
ENSG00000226281.1_RP1-80N2.2	RP1-80N2.2	ENSG00000226281.1	-0.004	0.002	-2.348	0.020
ENSG00000148950.5_IMMP1L	IMMP1L	ENSG00000148950.5	-0.974	0.415	-2.349	0.020
ENSG00000243444.3_PALM2	PALM2	ENSG00000243444.3	-0.245	0.104	-2.349	0.020
ENSG00000138623.5_SEMA7A	SEMA7A	ENSG00000138623.5	-1.352	0.575	-2.350	0.020
ENSG00000230133.1_RP5-1100I6.2	RP5-1100I6.2	ENSG00000230133.1	-0.011	0.005	-2.351	0.020

ENSG00000229901.1_RP11-399E6.4	RP11-399E6.4	ENSG00000229901.1	-0.025	0.011	-2.352	0.020
ENSG00000118873.10_RAB3GAP2	RAB3GAP2	ENSG00000118873.10	-0.238	0.101	-2.352	0.020
ENSG00000087301.4_TXNDC16	TXNDC16	ENSG00000087301.4	-0.137	0.058	-2.353	0.020
ENSG00000197756.4_RPL37A	RPL37A	ENSG00000197756.4	-93.855	39.867	-2.354	0.020
ENSG00000186310.8_NAP1L3	NAP1L3	ENSG00000186310.8	-4.434	1.883	-2.355	0.020
ENSG00000156735.6_BAG4	BAG4	ENSG00000156735.6	-0.568	0.241	-2.355	0.020
ENSG00000005020.8_SKAP2	SKAP2	ENSG00000005020.8	-0.324	0.137	-2.358	0.020
ENSG00000127152.13_BCL11B	BCL11B	ENSG00000127152.13	-0.125	0.053	-2.360	0.019
ENSG00000174938.10_SEZ6L2	SEZ6L2	ENSG00000174938.10	-4.618	1.955	-2.362	0.019
ENSG00000230391.1_AC017040.1	AC017040.1	ENSG00000230391.1	-0.038	0.016	-2.364	0.019
ENSG00000075089.5_ACTR6	ACTR6	ENSG00000075089.5	-0.645	0.273	-2.365	0.019
ENSG00000183023.13_SLC8A1	SLC8A1	ENSG00000183023.13	-0.177	0.075	-2.365	0.019
ENSG00000186187.7_ZNRF1	ZNRF1	ENSG00000186187.7	-0.564	0.238	-2.368	0.019
ENSG00000156261.7_CCT8	CCT8	ENSG00000156261.7	-2.298	0.970	-2.369	0.019
ENSG00000074706.9_IPCEF1	IPCEF1	ENSG00000074706.9	-0.934	0.394	-2.371	0.019
ENSG00000169926.5_KLF13	KLF13	ENSG00000169926.5	-1.632	0.688	-2.371	0.019
ENSG00000117114.14_LPHN2	LPHN2	ENSG00000117114.14	-0.554	0.233	-2.371	0.019
ENSG00000233397.1_AC008063.3	AC008063.3	ENSG00000233397.1	-0.014	0.006	-2.371	0.019
ENSG00000066933.11_MYO9A	MYO9A	ENSG00000066933.11	-0.485	0.205	-2.372	0.019
ENSG00000100749.3_VRK1	VRK1	ENSG00000100749.3	-0.367	0.155	-2.374	0.019
ENSG00000147099.15_HDAC8	HDAC8	ENSG00000147099.15	-0.422	0.178	-2.375	0.019
ENSG00000226790.2_HNRNPA3P1	HNRNPA3P1	ENSG00000226790.2	-0.002	0.001	-2.375	0.019
ENSG00000248905.4_FMN1	FMN1	ENSG00000248905.4	-0.152	0.064	-2.376	0.019
ENSG00000181751.5_C5orf30	C5orf30	ENSG00000181751.5	-0.495	0.208	-2.377	0.019
ENSG00000157450.11_RNF111	RNF111	ENSG00000157450.11	-0.326	0.137	-2.378	0.019
ENSG00000251325.1_RP11-415C15.3	RP11-415C15.3	ENSG00000251325.1	-0.001	0.000	-2.378	0.019
ENSG00000182366.3_FAM87A	FAM87A	ENSG00000182366.3	-0.078	0.033	-2.379	0.018
ENSG00000142875.14_PRKACB	PRKACB	ENSG00000142875.14	-3.556	1.495	-2.380	0.018
ENSG00000155719.12_OTOA	OTOA	ENSG00000155719.12	-0.053	0.022	-2.380	0.018
ENSG00000152061.17_RABGAP1L	RABGAP1L	ENSG00000152061.17	-0.447	0.188	-2.381	0.018
ENSG00000104408.5_EIF3E	EIF3E	ENSG00000104408.5	-2.431	1.020	-2.382	0.018
ENSG00000239579.2_MetazoaSRP	RN7SL325P	ENSG00000239579.2	0.000	0.000	-2.383	0.018
ENSG00000173715.9_C11orf80	C11orf80	ENSG00000173715.9	-0.595	0.249	-2.383	0.018
ENSG00000153132.8_CLGN	CLGN	ENSG00000153132.8	-0.169	0.071	-2.384	0.018
ENSG00000255836.1_RP11-157G21.2	RP11-157G21.2	ENSG00000255836.1	-0.367	0.154	-2.384	0.018
ENSG00000197535.10_MYO5A	MYO5A	ENSG00000197535.10	-1.168	0.490	-2.384	0.018
ENSG00000176749.4_CDK5R1	CDK5R1	ENSG00000176749.4	-2.416	1.013	-2.385	0.018
ENSG00000259426.1_RP11-253M7.1	RP11-253M7.1	ENSG00000259426.1	-0.033	0.014	-2.386	0.018
ENSG00000125834.8_STK35	STK35	ENSG00000125834.8	-0.320	0.134	-2.387	0.018
ENSG00000169071.9_ROR2	ROR2	ENSG00000169071.9	-0.027	0.011	-2.388	0.018
ENSG00000184788.8_SATL1	SATL1	ENSG00000184788.8	-0.061	0.025	-2.388	0.018
ENSG00000251659.1_CTD-2154I11.1	CTD-2154I11.1	ENSG00000251659.1	-0.016	0.007	-2.388	0.018
ENSG00000187627.9_RGPD1	RGPD1	ENSG00000187627.9	-0.198	0.083	-2.390	0.018
ENSG00000227659.1_CLYBL-AS2	CLYBL-AS2	ENSG00000227659.1	-0.026	0.011	-2.390	0.018
ENSG00000153130.12_SCOC	SCOC	ENSG00000153130.12	-3.032	1.268	-2.391	0.018
ENSG00000266489.1_RP11-178F10.3	RP11-178F10.3	ENSG00000266489.1	-0.410	0.172	-2.391	0.018
ENSG00000008277.10_ADAM22	ADAM22	ENSG00000008277.10	-0.505	0.211	-2.392	0.018
ENSG00000184809.8_C21orf88	C21orf88	ENSG00000184809.8	-0.121	0.050	-2.393	0.018
ENSG00000075790.6_BCAP29	BCAP29	ENSG00000075790.6	-0.764	0.319	-2.395	0.018
ENSG00000257151.1_RP11-701H24.2	RP11-701H24.2	ENSG00000257151.1	-2.070	0.864	-2.396	0.018
ENSG00000228038.1_VN1R51P	VN1R51P	ENSG00000228038.1	-0.065	0.027	-2.397	0.018
ENSG00000258780.1_RP11-492D6.2	RP11-492D6.2	ENSG00000258780.1	-0.038	0.016	-2.397	0.018
ENSG00000229921.2_KIF25-AS1	KIF25-AS1	ENSG00000229921.2	-0.054	0.023	-2.398	0.018
ENSG00000166922.4_SCG5	SCG5	ENSG00000166922.4	-9.077	3.785	-2.398	0.018
ENSG00000158445.6_KCNB1	KCNB1	ENSG00000158445.6	-0.434	0.181	-2.399	0.018
ENSG00000170634.7_ACYP2	ACYP2	ENSG00000170634.7	-1.707	0.712	-2.399	0.018
ENSG00000216471.3_RPSAP43	RPSAP43	ENSG00000216471.3	-0.185	0.077	-2.401	0.017
ENSG00000177447.6_CBX3P1	CBX3P1	ENSG00000177447.6	-0.127	0.053	-2.402	0.017
ENSG00000115155.12_OTOF	OTOF	ENSG00000115155.12	-0.039	0.016	-2.402	0.017
ENSG00000151917.12_BEND6	BEND6	ENSG00000151917.12	-1.179	0.491	-2.403	0.017

ENSG00000238082.1_AC009948.7	AC009948.7	ENSG00000238082.1	-0.051	0.021	-2.405	0.017
ENSG00000060656.14_PTPRU	PTPRU	ENSG00000060656.14	-0.681	0.283	-2.406	0.017
ENSG00000147180.12_ZNF711	ZNF711	ENSG00000147180.12	-0.276	0.115	-2.407	0.017
ENSG00000115966.11_ATF2	ATF2	ENSG00000115966.11	-0.546	0.227	-2.407	0.017
ENSG00000120820.8_GLT8D2	GLT8D2	ENSG00000120820.8	-0.120	0.050	-2.408	0.017
ENSG00000253603.1_CTA-397H3.3	CTA-397H3.3	ENSG00000253603.1	-0.010	0.004	-2.410	0.017
ENSG00000224905.2_AP001347.6	AP001347.6	ENSG00000224905.2	-0.214	0.089	-2.410	0.017
ENSG00000235982.1_AC007875.3	AC007875.3	ENSG00000235982.1	-0.231	0.096	-2.413	0.017
ENSG00000151849.10_CENPJ	CENPJ	ENSG00000151849.10	-0.210	0.087	-2.414	0.017
ENSG00000232650.1_RP5-834N19.1	RP5-834N19.1	ENSG00000232650.1	-0.019	0.008	-2.414	0.017
ENSG00000257194.1_RP11-567C2.1	RP11-567C2.1	ENSG00000257194.1	-0.058	0.024	-2.414	0.017
ENSG00000082213.13_C5orf22	C5orf22	ENSG00000082213.13	-0.362	0.150	-2.415	0.017
ENSG00000164603.7_C7orf60	C7orf60	ENSG00000164603.7	-0.161	0.067	-2.416	0.017
ENSG00000115289.8_PCGF1	PCGF1	ENSG00000115289.8	-0.724	0.300	-2.417	0.017
ENSG00000261595.1_RP11-426L16.9	RP11-426L16.9	ENSG00000261595.1	-0.006	0.002	-2.417	0.017
ENSG00000248516.1_RP11-265O12.1	RP11-265O12.1	ENSG00000248516.1	-0.004	0.002	-2.417	0.017
ENSG00000261496.1_RP13-514E23.1	RP13-514E23.1	ENSG00000261496.1	-1.230	0.509	-2.417	0.017
ENSG00000141452.5_C18orf8	C18orf8	ENSG00000141452.5	-0.746	0.309	-2.418	0.017
ENSG00000188659.5_FAM154B	FAM154B	ENSG00000188659.5	-0.120	0.050	-2.419	0.017
ENSG00000260802.1_RP6-170F5.2	RP6-170F5.2	ENSG00000260802.1	-0.002	0.001	-2.420	0.017
ENSG00000174444.10_RPL4	RPL4	ENSG00000174444.10	-15.458	6.384	-2.421	0.017
ENSG00000128573.17_FOXP2	FOXP2	ENSG00000128573.17	-0.135	0.056	-2.424	0.016
ENSG00000257060.2_RP11-266O8.1	RP11-266O8.1	ENSG00000257060.2	-0.026	0.011	-2.424	0.016
ENSG00000152763.11_WDR78	WDR78	ENSG00000152763.11	-0.099	0.041	-2.424	0.016
ENSG00000163536.7_SERPINI1	SERPINI1	ENSG00000163536.7	-9.086	3.748	-2.424	0.016
ENSG00000140280.9_LYSMD2	LYSMD2	ENSG00000140280.9	-0.886	0.365	-2.425	0.016
ENSG00000261161.1_RP11-58A18.1	RP11-58A18.1	ENSG00000261161.1	-0.091	0.037	-2.425	0.016
ENSG00000177108.5_ZDHHC22	ZDHHC22	ENSG00000177108.5	-1.417	0.584	-2.426	0.016
ENSG00000205325.1_AC005863.1	AC005863.1	ENSG00000205325.1	-0.004	0.002	-2.427	0.016
ENSG00000223625.1_CYCSP32	CYCSP32	ENSG00000223625.1	-0.657	0.271	-2.427	0.016
ENSG00000258038.1_CTD-2384A14.1	CTD-2384A14.1	ENSG00000258038.1	-0.021	0.009	-2.428	0.016
ENSG00000100554.7_ATP6V1D	ATP6V1D	ENSG00000100554.7	-3.362	1.384	-2.428	0.016
ENSG00000111912.14_NCOA7	NCOA7	ENSG00000111912.14	-2.438	1.004	-2.429	0.016
ENSG00000111832.8_RWDD1	RWDD1	ENSG00000111832.8	-1.843	0.759	-2.430	0.016
ENSG00000225057.2_AC096574.4	AC096574.4	ENSG00000225057.2	-0.016	0.007	-2.432	0.016
ENSG00000169306.5_IL1RAPL1	IL1RAPL1	ENSG00000169306.5	-0.200	0.082	-2.432	0.016
ENSG00000250091.2_DNAH10OS	DNAH10OS	ENSG00000250091.2	-0.168	0.069	-2.434	0.016
ENSG00000245614.2_DDX11-AS1	DDX11-AS1	ENSG00000245614.2	-0.033	0.014	-2.434	0.016
ENSG00000071539.9_TRIP13	TRIP13	ENSG00000071539.9	-0.068	0.028	-2.434	0.016
ENSG00000254715.2_OR7E154P	OR7E154P	ENSG00000254715.2	-0.375	0.154	-2.435	0.016
ENSG00000114770.12_ABCC5	ABCC5	ENSG00000114770.12	-1.124	0.461	-2.436	0.016
ENSG00000107105.9_ELAVL2	ELAVL2	ENSG00000107105.9	-0.969	0.398	-2.436	0.016
ENSG00000254589.1_AP000435.1	AP000435.1	ENSG00000254589.1	-0.016	0.007	-2.437	0.016
ENSG00000166986.8_MARS	MARS	ENSG00000166986.8	-2.957	1.213	-2.438	0.016
ENSG00000266145.1_RHOT1P1	RHOT1P1	ENSG00000266145.1	-0.207	0.085	-2.438	0.016
ENSG00000186377.6_CYP4X1	CYP4X1	ENSG00000186377.6	-0.452	0.185	-2.438	0.016
ENSG00000168386.14_FILIP1L	FILIP1L	ENSG00000168386.14	-0.322	0.132	-2.438	0.016
ENSG00000139116.13_KIF21A	KIF21A	ENSG00000139116.13	-1.702	0.698	-2.439	0.016
ENSG00000189316.3_RP11-797H7.5	RP11-797H7.5	ENSG00000189316.3	-0.249	0.102	-2.439	0.016
ENSG00000260425.1_LA16c-316G12.2	LA16c-316G12.2	ENSG00000260425.1	-0.098	0.040	-2.439	0.016
ENSG00000102053.11_ZC3H12B	ZC3H12B	ENSG00000102053.11	-0.092	0.038	-2.440	0.016
ENSG00000081386.8_ZNF510	ZNF510	ENSG00000081386.8	-0.145	0.059	-2.440	0.016
ENSG00000236016.2_RP1-3J17.3	RP1-3J17.3	ENSG00000236016.2	0.000	0.000	-2.440	0.016
ENSG00000125629.10_INSIG2	INSIG2	ENSG00000125629.10	-0.324	0.133	-2.441	0.016
ENSG00000189337.10_KAZN	KAZN	ENSG00000189337.10	-0.688	0.282	-2.441	0.016
ENSG00000241360.1_PDXP	PDXP	ENSG00000241360.1	-5.703	2.335	-2.443	0.016
ENSG00000134504.8_KCTD1	KCTD1	ENSG00000134504.8	-1.330	0.545	-2.443	0.016
ENSG00000101654.13_RNMT	RNMT	ENSG00000101654.13	-1.189	0.487	-2.443	0.016
ENSG00000023572.4_GLRX2	GLRX2	ENSG00000023572.4	-1.177	0.482	-2.443	0.016
ENSG00000120057.4_SFRP5	SFRP5	ENSG00000120057.4	-0.089	0.036	-2.443	0.016

ENSG00000247903.1_RP11-421F16.3	RP11-421F16.3	ENSG00000247903.1	-0.003	0.001	-2.444	0.016
ENSG00000226649.1_AC019118.4	AC019118.4	ENSG00000226649.1	0.000	0.000	-2.444	0.016
ENSG00000236686.1_BZW1P1	BZW1P1	ENSG00000236686.1	0.000	0.000	-2.444	0.016
ENSG00000227198.1_C6orf47-AS1	C6orf47-AS1	ENSG00000227198.1	0.000	0.000	-2.444	0.016
ENSG00000232792.1_FTH1P25	FTH1P25	ENSG00000232792.1	0.000	0.000	-2.444	0.016
ENSG00000242610.1_OR5BH1P	OR5BH1P	ENSG00000242610.1	0.000	0.000	-2.444	0.016
ENSG00000234701.1_PRDX3P3	PRDX3P3	ENSG00000234701.1	0.000	0.000	-2.444	0.016
ENSG00000236894.1_RP11-128B16.3	RP11-128B16.3	ENSG00000236894.1	0.000	0.000	-2.444	0.016
ENSG00000249697.1_RP11-155L15.1	RP11-155L15.1	ENSG00000249697.1	0.000	0.000	-2.444	0.016
ENSG00000235745.1_RP11-236F9.5	RP11-236F9.5	ENSG00000235745.1	0.000	0.000	-2.444	0.016
ENSG00000218716.1_RP11-302L19.2	RP11-302L19.2	ENSG00000218716.1	0.000	0.000	-2.444	0.016
ENSG00000248317.1_RP11-463H12.2	RP11-463H12.2	ENSG00000248317.1	0.000	0.000	-2.444	0.016
ENSG00000223707.1_RP1-93L7.2	RP1-93L7.2	ENSG00000223707.1	-0.001	0.000	-2.444	0.016
ENSG00000182621.12_PLCB1	PLCB1	ENSG00000182621.12	-1.243	0.508	-2.445	0.015
ENSG00000130538.4_OR11H1	OR11H1	ENSG00000130538.4	-0.012	0.005	-2.446	0.015
ENSG00000233483.2_CTD-2020K17.4	CTD-2020K17.4	ENSG00000233483.2	-0.416	0.170	-2.446	0.015
ENSG00000178965.9_C1orf173	C1orf173	ENSG00000178965.9	-0.703	0.288	-2.446	0.015
ENSG00000186462.7_NAP1L2	NAP1L2	ENSG00000186462.7	-3.150	1.287	-2.448	0.015
ENSG00000188042.5_ARL4C	ARL4C	ENSG00000188042.5	-1.047	0.428	-2.448	0.015
ENSG00000185689.11_C6orf201	C6orf201	ENSG00000185689.11	-0.103	0.042	-2.449	0.015
ENSG00000254698.1_RP11-659G9.3	RP11-659G9.3	ENSG00000254698.1	-0.025	0.010	-2.449	0.015
ENSG00000181191.11_PJA1	PJA1	ENSG00000181191.11	-1.622	0.662	-2.450	0.015
ENSG00000233664.1_NDUFS5P3	NDUFS5P3	ENSG00000233664.1	-0.137	0.056	-2.451	0.015
ENSG00000196911.5_KPNA5	KPNA5	ENSG00000196911.5	-0.373	0.152	-2.451	0.015
ENSG00000250443.2_AC128709.1	AC128709.1	ENSG00000250443.2	-0.256	0.105	-2.452	0.015
ENSG00000111707.7_SUDS3	SUDS3	ENSG00000111707.7	-0.377	0.154	-2.455	0.015
ENSG00000123570.3_RAB9B	RAB9B	ENSG00000123570.3	-0.319	0.130	-2.455	0.015
ENSG00000240294.2_7SK	RN7SKP241	ENSG00000240294.2	-0.059	0.024	-2.457	0.015
ENSG00000074276.6_CDHR2	CDHR2	ENSG00000074276.6	-0.066	0.027	-2.458	0.015
ENSG00000181904.8_C5orf24	C5orf24	ENSG00000181904.8	-0.548	0.223	-2.458	0.015
ENSG00000260296.1_RP11-395I6.3	RP11-395I6.3	ENSG00000260296.1	-0.350	0.142	-2.459	0.015
ENSG00000251664.2_PCDHA12	PCDHA12	ENSG00000251664.2	-0.082	0.033	-2.459	0.015
ENSG00000225673.3_RP11-641C17.3	RP11-641C17.3	ENSG00000225673.3	-0.022	0.009	-2.460	0.015
ENSG00000138311.11_ZNF365	ZNF365	ENSG00000138311.11	-1.883	0.765	-2.461	0.015
ENSG00000258215.1_RP11-299A16.1	RP11-299A16.1	ENSG00000258215.1	-0.023	0.009	-2.464	0.015
ENSG00000163848.14_ZNF148	ZNF148	ENSG00000163848.14	-0.329	0.133	-2.464	0.015
ENSG00000100129.11_EIF3L	EIF3L	ENSG00000100129.11	-4.165	1.690	-2.465	0.015
ENSG00000233932.3_CTXN2	CTXN2	ENSG00000233932.3	-0.575	0.233	-2.466	0.015
ENSG00000198783.4_ZNF830	ZNF830	ENSG00000198783.4	-0.262	0.106	-2.467	0.015
ENSG00000165966.10_PDZRN4	PDZRN4	ENSG00000165966.10	-0.158	0.064	-2.467	0.015
ENSG00000145147.14_SLIT2	SLIT2	ENSG00000145147.14	-0.305	0.124	-2.468	0.015
ENSG00000156140.4_ADAMTS3	ADAMTS3	ENSG00000156140.4	-0.059	0.024	-2.469	0.015
ENSG00000234005.3_GAPDHP22	GAPDHP22	ENSG00000234005.3	-0.067	0.027	-2.471	0.014
ENSG00000255150.1_EID3	EID3	ENSG00000255150.1	-0.113	0.046	-2.472	0.014
ENSG00000263878.1_DLGAP1-AS4	DLGAP1-AS4	ENSG00000263878.1	-0.445	0.180	-2.474	0.014
ENSG00000257842.1_NOVA1-AS1	NOVA1-AS1	ENSG00000257842.1	-0.031	0.012	-2.474	0.014
ENSG00000137731.8_FXYD2	FXYD2	ENSG00000137731.8	-0.114	0.046	-2.474	0.014
ENSG00000250748.2_RP11-230G5.2	RP11-230G5.2	ENSG00000250748.2	-0.114	0.046	-2.477	0.014
ENSG00000231862.1_RP11-34F20.5	RP11-34F20.5	ENSG00000231862.1	-0.002	0.001	-2.478	0.014
ENSG00000185246.13_PRPF39	PRPF39	ENSG00000185246.13	-0.408	0.165	-2.478	0.014
ENSG00000256861.1_RP11-512M8.5	RP11-512M8.5	ENSG00000256861.1	-0.302	0.122	-2.478	0.014
ENSG00000267655.1_CTD-2286N8.2	CTD-2286N8.2	ENSG00000267655.1	-0.251	0.101	-2.480	0.014
ENSG00000146267.10_FAXC	FAXC	ENSG00000146267.10	-0.705	0.284	-2.481	0.014
ENSG00000166501.8_PRKCB	PRKCB	ENSG00000166501.8	-4.101	1.651	-2.484	0.014
ENSG00000178397.8_FAM220A	FAM220A	ENSG00000178397.8	-0.514	0.207	-2.485	0.014
ENSG00000226328.1_CTA-217C2.1	CTA-217C2.1	ENSG00000226328.1	-0.433	0.174	-2.485	0.014
ENSG00000159882.8_ZNF230	ZNF230	ENSG00000159882.8	-0.076	0.031	-2.487	0.014
ENSG00000076554.10_TPD52	TPD52	ENSG00000076554.10	-2.049	0.824	-2.487	0.014
ENSG00000087448.5_KLHDC5	KLHDC5	ENSG00000087448.5	-0.376	0.151	-2.489	0.014
ENSG00000169057.14_MECP2	MECP2	ENSG00000169057.14	-0.424	0.170	-2.490	0.014

ENSG00000228314.1_CYP4F29P	CYP4F29P	ENSG00000228314.1	-0.547	0.220	-2.492	0.014
ENSG00000168275.10_C1orf31	C1orf31	ENSG00000168275.10	-1.468	0.589	-2.492	0.014
ENSG00000005302.12_MSL3	MSL3	ENSG00000005302.12	-0.442	0.177	-2.492	0.014
ENSG00000196591.7_HDAC2	HDAC2	ENSG00000196591.7	-0.659	0.264	-2.493	0.014
ENSG00000260244.1_RP11-588K22.2	RP11-588K22.2	ENSG00000260244.1	-0.333	0.134	-2.493	0.014
ENSG00000266850.1_RP11-370A5.1	RP11-370A5.1	ENSG00000266850.1	-0.365	0.146	-2.493	0.014
ENSG00000135747.7_ZNF670	ZNF670	ENSG00000135747.7	-0.058	0.023	-2.495	0.014
ENSG00000134852.10_CLOCK	CLOCK	ENSG00000134852.10	-0.250	0.100	-2.495	0.014
ENSG00000261000.1_RP11-534L20.5	RP11-534L20.5	ENSG00000261000.1	-0.039	0.016	-2.496	0.014
ENSG00000154473.11_BUB3	BUB3	ENSG00000154473.11	-0.634	0.254	-2.497	0.013
ENSG00000196090.7_PTPRT	PTPRT	ENSG00000196090.7	-0.573	0.229	-2.500	0.013
ENSG00000129028.4_THAP10	THAP10	ENSG00000129028.4	-0.250	0.100	-2.500	0.013
ENSG00000213252.3_RP11-819M15.1	RP11-819M15.1	ENSG00000213252.3	-0.017	0.007	-2.503	0.013
ENSG00000255330.4_RP3-403A15.5	RP3-403A15.5	ENSG00000255330.4	-0.561	0.224	-2.503	0.013
ENSG00000199313.1_U4	U4	ENSG00000199313.1	-0.064	0.025	-2.509	0.013
ENSG00000196353.7_CPNE4	CPNE4	ENSG00000196353.7	-1.110	0.442	-2.511	0.013
ENSG00000235285.1_SMIM2-IT1	SMIM2-IT1	ENSG00000235285.1	-0.018	0.007	-2.512	0.013
ENSG00000220563.1_PKMP3	PKMP3	ENSG00000220563.1	-0.359	0.143	-2.512	0.013
ENSG00000177519.3_RPRM	RPRM	ENSG00000177519.3	-0.747	0.297	-2.513	0.013
ENSG00000237149.3_ZNF503-AS2	ZNF503-AS2	ENSG00000237149.3	-0.087	0.035	-2.514	0.013
ENSG00000164128.2_NPY1R	NPY1R	ENSG00000164128.2	-0.335	0.133	-2.514	0.013
ENSG00000257608.1_CTD-2311B13.6	CTD-2311B13.6	ENSG00000257608.1	-0.021	0.008	-2.515	0.013
ENSG00000125966.8_MMP24	MMP24	ENSG00000125966.8	-0.352	0.140	-2.515	0.013
ENSG00000215447.3_BX322557.10	BX322557.10	ENSG00000215447.3	-1.957	0.778	-2.517	0.013
ENSG00000172465.9_TCEAL1	TCEAL1	ENSG00000172465.9	-1.587	0.630	-2.518	0.013
ENSG00000072657.4_TRHDE	TRHDE	ENSG00000072657.4	-0.251	0.100	-2.518	0.013
ENSG00000254706.1_RP11-565P22.6	RP11-565P22.6	ENSG00000254706.1	-0.025	0.010	-2.519	0.013
ENSG00000150672.12_DLG2	DLG2	ENSG00000150672.12	-1.285	0.510	-2.520	0.013
ENSG00000260941.1_LINC00622	LINC00622	ENSG00000260941.1	-0.446	0.177	-2.521	0.013
ENSG00000260837.1_RP11-434B12.1	RP11-434B12.1	ENSG00000260837.1	-0.993	0.394	-2.523	0.013
ENSG00000214700.5_C12orf71	C12orf71	ENSG00000214700.5	-0.016	0.006	-2.524	0.013
ENSG00000254759.1_NAP1L1P1	NAP1L1P1	ENSG00000254759.1	-0.030	0.012	-2.526	0.012
ENSG00000249781.1_CTD-2143L24.1	CTD-2143L24.1	ENSG00000249781.1	-0.016	0.006	-2.526	0.012
ENSG00000057663.8_ATG5	ATG5	ENSG00000057663.8	-0.313	0.124	-2.527	0.012
ENSG00000163281.6_GNPDA2	GNPDA2	ENSG00000163281.6	-0.283	0.112	-2.527	0.012
ENSG00000102290.16_PCDH11X	PCDH11X	ENSG00000102290.16	-0.102	0.040	-2.530	0.012
ENSG00000105492.10_SIGLEC6	SIGLEC6	ENSG00000105492.10	-0.010	0.004	-2.531	0.012
ENSG00000229020.3_AKR7A2P1	AKR7A2P1	ENSG00000229020.3	-0.028	0.011	-2.531	0.012
ENSG00000138078.11_PREPL	PREPL	ENSG00000138078.11	-5.133	2.027	-2.532	0.012
ENSG00000088367.15_EPB41L1	EPB41L1	ENSG00000088367.15	-3.278	1.294	-2.533	0.012
ENSG00000169758.8_C15orf27	C15orf27	ENSG00000169758.8	-0.285	0.112	-2.533	0.012
ENSG00000151090.13_THRB	THRB	ENSG00000151090.13	-1.067	0.421	-2.534	0.012
ENSG00000005955.8_GGNBP2	GGNBP2	ENSG00000005955.8	-1.458	0.575	-2.534	0.012
ENSG00000149054.10_ZNF215	ZNF215	ENSG00000149054.10	-0.045	0.018	-2.537	0.012
ENSG00000261727.2_RP11-1166P10.6	RP11-1166P10.6	ENSG00000261727.2	-0.044	0.017	-2.537	0.012
ENSG00000263906.1_RP11-680F20.11	RP11-680F20.11	ENSG00000263906.1	-0.147	0.058	-2.539	0.012
ENSG00000111445.9_RFC5	RFC5	ENSG00000111445.9	-0.213	0.084	-2.539	0.012
ENSG00000261409.1_RP6-24A23.7	RP6-24A23.7	ENSG00000261409.1	-0.025	0.010	-2.540	0.012
ENSG00000213881.3_NPM1P6	NPM1P6	ENSG00000213881.3	-0.028	0.011	-2.541	0.012
ENSG00000156531.12_PHF6	PHF6	ENSG00000156531.12	-0.177	0.070	-2.541	0.012
ENSG00000173557.10_C2orf70	C2orf70	ENSG00000173557.10	-0.048	0.019	-2.544	0.012
ENSG00000121101.11_TEX14	TEX14	ENSG00000121101.11	-0.071	0.028	-2.545	0.012
ENSG00000139697.6_SBNO1	SBNO1	ENSG00000139697.6	-0.365	0.143	-2.545	0.012
ENSG00000067715.9_SYT1	SYT1	ENSG00000067715.9	-16.877	6.628	-2.546	0.012
ENSG00000181016.4_C7orf53	C7orf53	ENSG00000181016.4	-0.307	0.120	-2.549	0.012
ENSG00000259826.1_RP11-467D6.1	RP11-467D6.1	ENSG00000259826.1	-0.061	0.024	-2.549	0.012
ENSG00000260512.1_RP13-259F12.2	RP13-259F12.2	ENSG00000260512.1	-0.311	0.122	-2.549	0.012
ENSG00000196589.3_AC010606.1	AC010606.1	ENSG00000196589.3	-0.015	0.006	-2.558	0.011
ENSG00000229536.1_AC079776.1	AC079776.1	ENSG00000229536.1	-0.018	0.007	-2.560	0.011
ENSG00000227486.1_RP13-188A5.1	RP13-188A5.1	ENSG00000227486.1	-0.049	0.019	-2.562	0.011

ENSG00000156011.12_PSD3	PSD3	ENSG00000156011.12	-1.211	0.472	-2.564	0.011
ENSG00000170962.8_PDGFD	PDGFD	ENSG00000170962.8	-0.050	0.019	-2.564	0.011
ENSG00000149635.2_OCSTAMP	OCSTAMP	ENSG00000149635.2	-0.007	0.003	-2.567	0.011
ENSG00000178803.6_C22orf45	C22orf45	ENSG00000178803.6	-0.006	0.002	-2.568	0.011
ENSG00000101746.11_NOL4	NOL4	ENSG00000101746.11	-0.503	0.196	-2.570	0.011
ENSG00000065548.13_ZC3H15	ZC3H15	ENSG00000065548.13	-1.745	0.679	-2.570	0.011
ENSG00000165379.9_LRFN5	LRFN5	ENSG00000165379.9	-0.574	0.223	-2.572	0.011
ENSG00000238151.1_MLLT10P1	MLLT10P1	ENSG00000238151.1	-0.088	0.034	-2.573	0.011
ENSG00000187772.6_LIN28B	LIN28B	ENSG00000187772.6	-0.022	0.009	-2.573	0.011
ENSG00000261736.1_AC002551.1	AC002551.1	ENSG00000261736.1	-0.033	0.013	-2.573	0.011
ENSG00000225504.3_RP11-160A10.2	RP11-160A10.2	ENSG00000225504.3	-0.550	0.214	-2.573	0.011
ENSG00000215559.4_ANKRD20A11P	ANKRD20A11P	ENSG00000215559.4	-1.653	0.642	-2.574	0.011
ENSG00000145920.10_CPLX2	CPLX2	ENSG00000145920.10	-14.188	5.511	-2.575	0.011
ENSG00000179542.11_SLITRK4	SLITRK4	ENSG00000179542.11	-0.627	0.243	-2.576	0.011
ENSG00000237901.1_AC009161.1	AC009161.1	ENSG00000237901.1	-0.008	0.003	-2.576	0.011
ENSG00000055917.11_PUM2	PUM2	ENSG00000055917.11	-0.684	0.265	-2.580	0.011
ENSG00000173114.7_LRRN3	LRRN3	ENSG00000173114.7	-0.602	0.233	-2.582	0.011
ENSG00000250988.3_RP11-752G15.6	RP11-752G15.6	ENSG00000250988.3	-0.541	0.209	-2.583	0.011
ENSG00000230006.3_ANKRD36BP2	ANKRD36BP2	ENSG00000230006.3	-0.138	0.053	-2.587	0.011
ENSG00000179935.5_LINC00652	LINC00652	ENSG00000179935.5	-0.068	0.026	-2.588	0.010
ENSG00000168830.5_HTR1E	HTR1E	ENSG00000168830.5	-0.249	0.096	-2.589	0.010
ENSG00000102931.3_ARL2BP	ARL2BP	ENSG00000102931.3	-0.785	0.303	-2.590	0.010
ENSG00000256232.1_RP11-771K4.1	RP11-771K4.1	ENSG00000256232.1	-0.025	0.010	-2.591	0.010
ENSG00000240652.1_RP11-832N8.1	RP11-832N8.1	ENSG00000240652.1	-0.005	0.002	-2.592	0.010
ENSG00000163171.6_CDC42EP3	CDC42EP3	ENSG00000163171.6	-0.198	0.076	-2.593	0.010
ENSG00000238639.1_snoU13	snoU13	ENSG00000238639.1	0.000	0.000	-2.594	0.010
ENSG00000230027.1_RP11-550H2.2	RP11-550H2.2	ENSG00000230027.1	-0.134	0.051	-2.597	0.010
ENSG00000132139.8_GAS2L2	GAS2L2	ENSG00000132139.8	-0.070	0.027	-2.598	0.010
ENSG00000232514.1_RP11-329A14.2	RP11-329A14.2	ENSG00000232514.1	-0.043	0.017	-2.598	0.010
ENSG00000183155.4_RABIF	RABIF	ENSG00000183155.4	-0.339	0.130	-2.599	0.010
ENSG00000108176.8_DNAJC12	DNAJC12	ENSG00000108176.8	-0.942	0.362	-2.599	0.010
ENSG00000112038.12_OPRM1	OPRM1	ENSG00000112038.12	-0.063	0.024	-2.600	0.010
ENSG00000259745.1_RP11-809H16.3	RP11-809H16.3	ENSG00000259745.1	-0.007	0.003	-2.602	0.010
ENSG00000261043.1_MIR4313	MIR4313	ENSG00000261043.1	-0.002	0.001	-2.603	0.010
ENSG00000230807.1_AC099535.4	AC099535.4	ENSG00000230807.1	-0.035	0.013	-2.605	0.010
ENSG00000100442.6_FKBP3	FKBP3	ENSG00000100442.6	-3.262	1.251	-2.606	0.010
ENSG00000206052.6_DOK6	DOK6	ENSG00000206052.6	-0.286	0.110	-2.608	0.010
ENSG00000135698.5_MPHOSPH6	MPHOSPH6	ENSG00000135698.5	-0.980	0.375	-2.610	0.010
ENSG00000135333.9_EPHA7	EPHA7	ENSG00000135333.9	-0.114	0.044	-2.610	0.010
ENSG00000182931.5_WFDC10B	WFDC10B	ENSG00000182931.5	-0.026	0.010	-2.616	0.010
ENSG00000049860.9_HEXB	HEXB	ENSG00000049860.9	-1.330	0.508	-2.619	0.010
ENSG00000175707.7_C1orf172	C1orf172	ENSG00000175707.7	-0.137	0.052	-2.619	0.010
ENSG00000153253.10_SCN3A	SCN3A	ENSG00000153253.10	-0.849	0.323	-2.624	0.009
ENSG00000198673.6_FAM19A2	FAM19A2	ENSG00000198673.6	-0.855	0.326	-2.624	0.009
ENSG00000162374.12_ELAVL4	ELAVL4	ENSG00000162374.12	-2.940	1.120	-2.625	0.009
ENSG00000244427.1_RP11-52B19.1	RP11-52B19.1	ENSG00000244427.1	-0.061	0.023	-2.626	0.009
ENSG00000152377.8_SPOCK1	SPOCK1	ENSG00000152377.8	-2.114	0.805	-2.628	0.009
ENSG00000234516.1_AL121758.1	AL121758.1	ENSG00000234516.1	-1.081	0.411	-2.630	0.009
ENSG00000255494.1_KIAA1456	KIAA1456	ENSG00000255494.1	-0.022	0.009	-2.630	0.009
ENSG00000164114.14_MAP9	MAP9	ENSG00000164114.14	-2.932	1.115	-2.630	0.009
ENSG00000140992.13_PDPK1	PDPK1	ENSG00000140992.13	-1.022	0.388	-2.635	0.009
ENSG00000022840.9_RNF10	RNF10	ENSG00000022840.9	-2.637	1.001	-2.635	0.009
ENSG00000197008.5_ZNF138	ZNF138	ENSG00000197008.5	-0.211	0.080	-2.636	0.009
ENSG00000196468.5_FGF16	FGF16	ENSG00000196468.5	-0.019	0.007	-2.637	0.009
ENSG00000152402.5_GUCY1A2	GUCY1A2	ENSG00000152402.5	-0.076	0.029	-2.640	0.009
ENSG00000248585.2_RP11-90P5.1	RP11-90P5.1	ENSG00000248585.2	-0.011	0.004	-2.641	0.009
ENSG00000176542.5_KIAA2018	KIAA2018	ENSG00000176542.5	-0.201	0.076	-2.642	0.009
ENSG00000070193.4_FGF10	FGF10	ENSG00000070193.4	-0.031	0.012	-2.644	0.009
ENSG00000232001.1_AC108868.6	AC108868.6	ENSG00000232001.1	-0.026	0.010	-2.646	0.009
ENSG00000127995.12_CASD1	CASD1	ENSG00000127995.12	-0.876	0.330	-2.654	0.009

ENSG00000227713.1_AC116609.1	AC116609.1	ENSG00000227713.1	-0.047	0.018	-2.656	0.009
ENSG00000182568.11_SATB1	SATB1	ENSG00000182568.11	-1.506	0.567	-2.656	0.009
ENSG00000187258.9_NPSR1	NPSR1	ENSG00000187258.9	-0.018	0.007	-2.661	0.009
ENSG00000203877.3_RIPPLY2	RIPPLY2	ENSG00000203877.3	-1.086	0.408	-2.662	0.009
ENSG00000144331.14_ZNF385B	ZNF385B	ENSG00000144331.14	-0.482	0.181	-2.664	0.008
ENSG00000224287.1_MSL3P1	MSL3P1	ENSG00000224287.1	-0.113	0.042	-2.667	0.008
ENSG00000198689.5_SLC9A6	SLC9A6	ENSG00000198689.5	-1.459	0.545	-2.675	0.008
ENSG00000259241.1_RP11-313P18.2	RP11-313P18.2	ENSG00000259241.1	-0.010	0.004	-2.675	0.008
ENSG00000179083.5_FAM133A	FAM133A	ENSG00000179083.5	-0.379	0.142	-2.677	0.008
ENSG00000184672.7_RALYL	RALYL	ENSG00000184672.7	-1.378	0.515	-2.677	0.008
ENSG00000153714.5_LURAP1L	LURAP1L	ENSG00000153714.5	-0.240	0.090	-2.678	0.008
ENSG00000155367.10_PPM1J	PPM1J	ENSG00000155367.10	-0.459	0.171	-2.680	0.008
ENSG00000113552.11_GNPDA1	GNPDA1	ENSG00000113552.11	-0.634	0.237	-2.680	0.008
ENSG00000232110.2_RP11-149I23.3	RP11-149I23.3	ENSG00000232110.2	-0.539	0.201	-2.684	0.008
ENSG00000151962.3_RBM46	RBM46	ENSG00000151962.3	-0.008	0.003	-2.684	0.008
ENSG00000184349.8_EFNA5	EFNA5	ENSG00000184349.8	-0.366	0.136	-2.685	0.008
ENSG00000172292.10_CERS6	CERS6	ENSG00000172292.10	-0.606	0.226	-2.686	0.008
ENSG00000266664.1_RP11-160E2.21	RP11-160E2.21	ENSG00000266664.1	-0.001	0.000	-2.689	0.008
ENSG00000235266.1_RP11-753C18.8	RP11-753C18.8	ENSG00000235266.1	-0.114	0.042	-2.690	0.008
ENSG00000101977.15_MCF2	MCF2	ENSG00000101977.15	-0.460	0.171	-2.693	0.008
ENSG00000239334.1_GSTTP2	GSTTP2	ENSG00000239334.1	-0.008	0.003	-2.693	0.008
ENSG00000262951.1_RP11-670E13.2	RP11-670E13.2	ENSG00000262951.1	-0.322	0.119	-2.694	0.008
ENSG00000245598.2_DACT3-AS1	DACT3-AS1	ENSG00000245598.2	-0.095	0.035	-2.694	0.008
ENSG00000229797.1_AC140481.8	AC140481.8	ENSG00000229797.1	-0.052	0.019	-2.694	0.008
ENSG00000171385.5_KCND3	KCND3	ENSG00000171385.5	-0.837	0.310	-2.696	0.008
ENSG00000204970.5_PCDHA1	PCDHA1	ENSG00000204970.5	-0.604	0.224	-2.697	0.008
ENSG00000048540.10_LMO3	LMO3	ENSG00000048540.10	-3.111	1.152	-2.700	0.008
ENSG00000143702.11_CEP170	CEP170	ENSG00000143702.11	-0.688	0.255	-2.700	0.008
ENSG00000261311.1_RP5-1014C4.3	RP5-1014C4.3	ENSG00000261311.1	-0.170	0.063	-2.704	0.008
ENSG00000144410.4_CPO	CPO	ENSG00000144410.4	-0.132	0.049	-2.705	0.008
ENSG00000226970.2_RP11-82H13.2	RP11-82H13.2	ENSG00000226970.2	-0.164	0.061	-2.707	0.007
ENSG00000147650.6_LRP12	LRP12	ENSG00000147650.6	-0.236	0.087	-2.708	0.007
ENSG00000183807.6_FAM162B	FAM162B	ENSG00000183807.6	-0.255	0.094	-2.713	0.007
ENSG00000133019.7_CHRM3	CHRM3	ENSG00000133019.7	-0.453	0.167	-2.718	0.007
ENSG00000214595.6_EML6	EML6	ENSG00000214595.6	-0.409	0.150	-2.720	0.007
ENSG00000264985.1_RP11-449L23.2	RP11-449L23.2	ENSG00000264985.1	-0.014	0.005	-2.722	0.007
ENSG00000078328.15_RBFOX1	RBFOX1	ENSG00000078328.15	-4.543	1.662	-2.733	0.007
ENSG00000136535.10_TBR1	TBR1	ENSG00000136535.10	-1.817	0.664	-2.734	0.007
ENSG00000257512.1_RP11-486A14.1	RP11-486A14.1	ENSG00000257512.1	-0.009	0.003	-2.736	0.007
ENSG00000166226.7_CCT2	CCT2	ENSG00000166226.7	-1.672	0.610	-2.743	0.007
ENSG00000047597.5_XK	XK	ENSG00000047597.5	-0.183	0.067	-2.745	0.007
ENSG00000091664.7_SLC17A6	SLC17A6	ENSG00000091664.7	-0.230	0.084	-2.746	0.007
ENSG00000265327.1_MetazoaSRP	RN7SL411P	ENSG00000265327.1	-0.044	0.016	-2.747	0.007
ENSG00000163798.9_SLC4A1AP	SLC4A1AP	ENSG00000163798.9	-0.786	0.286	-2.751	0.007
ENSG00000196644.8_GPR89C	GPR89C	ENSG00000196644.8	-0.281	0.102	-2.752	0.007
ENSG00000259787.1_CTB-85P21.2	CTB-85P21.2	ENSG00000259787.1	-0.624	0.227	-2.753	0.007
ENSG00000047932.9_GOPC	GOPC	ENSG00000047932.9	-0.443	0.161	-2.754	0.007
ENSG00000112053.9_SLC26A8	SLC26A8	ENSG00000112053.9	-0.224	0.081	-2.754	0.007
ENSG00000052795.8_FNIP2	FNIP2	ENSG00000052795.8	-0.400	0.145	-2.755	0.007
ENSG00000169288.13_MRPL1	MRPL1	ENSG00000169288.13	-0.988	0.359	-2.755	0.007
ENSG00000259623.1_RP11-156E6.1	RP11-156E6.1	ENSG00000259623.1	-0.588	0.213	-2.759	0.006
ENSG00000215030.4_RPL13P12	RPL13P12	ENSG00000215030.4	-2.086	0.756	-2.760	0.006
ENSG00000155961.4_RAB39B	RAB39B	ENSG00000155961.4	-0.362	0.131	-2.765	0.006
ENSG00000177459.6_C8orf47	C8orf47	ENSG00000177459.6	-0.118	0.042	-2.774	0.006
ENSG00000164182.6_NDUFAF2	NDUFAF2	ENSG00000164182.6	-1.757	0.633	-2.774	0.006
ENSG00000148153.7_INIP	INIP	ENSG00000148153.7	-0.361	0.130	-2.774	0.006
ENSG00000171587.10_DSCAM	DSCAM	ENSG00000171587.10	-0.155	0.056	-2.779	0.006
ENSG00000259959.1_RP11-121C2.2	RP11-121C2.2	ENSG00000259959.1	-0.100	0.036	-2.782	0.006
ENSG00000077616.6_NAALAD2	NAALAD2	ENSG00000077616.6	-0.330	0.118	-2.785	0.006
ENSG00000162961.9_DPY30	DPY30	ENSG00000162961.9	-1.862	0.669	-2.785	0.006

ENSG00000260711.1_RP11-747H7.3	RP11-747H7.3	ENSG00000260711.1	-0.053	0.019	-2.792	0.006
ENSG00000109881.11_CCDC34	CCDC34	ENSG00000109881.11	-1.812	0.649	-2.794	0.006
ENSG00000196138.3_AC005831.1	AC005831.1	ENSG00000196138.3	-0.031	0.011	-2.796	0.006
ENSG00000182836.5_PLCXD3	PLCXD3	ENSG00000182836.5	-0.271	0.097	-2.798	0.006
ENSG00000145743.10_FBXL17	FBXL17	ENSG00000145743.10	-0.506	0.180	-2.804	0.006
ENSG00000226276.1_AC093382.1	AC093382.1	ENSG00000226276.1	-0.048	0.017	-2.806	0.006
ENSG00000251639.1_RP11-20I20.1	RP11-20I20.1	ENSG00000251639.1	-0.030	0.011	-2.810	0.006
ENSG00000175471.14_MCTP1	MCTP1	ENSG00000175471.14	-0.464	0.165	-2.811	0.006
ENSG00000213182.3_OR10D5P	OR10D5P	ENSG00000213182.3	0.000	0.000	-2.814	0.005
ENSG00000109452.8_INPP4B	INPP4B	ENSG00000109452.8	-0.184	0.065	-2.814	0.005
ENSG00000267381.1_CTD-2086O20.3	CTD-2086O20.3	ENSG00000267381.1	-0.412	0.147	-2.814	0.005
ENSG00000005249.7_PRKAR2B	PRKAR2B	ENSG00000005249.7	-1.229	0.436	-2.815	0.005
ENSG00000237818.1_AC079781.9	AC079781.9	ENSG00000237818.1	-0.170	0.060	-2.816	0.005
ENSG00000147642.12_SYBU	SYBU	ENSG00000147642.12	-2.201	0.782	-2.816	0.005
ENSG00000234735.1_AL022237.3	AL022237.3	ENSG00000234735.1	-0.005	0.002	-2.822	0.005
ENSG00000171533.7_MAP6	MAP6	ENSG00000171533.7	-2.295	0.813	-2.822	0.005
ENSG00000214174.4_AMZ2P1	AMZ2P1	ENSG00000214174.4	-0.475	0.168	-2.825	0.005
ENSG00000115252.14_PDE1A	PDE1A	ENSG00000115252.14	-0.833	0.295	-2.826	0.005
ENSG00000139209.11_SLC38A4	SLC38A4	ENSG00000139209.11	-0.011	0.004	-2.833	0.005
ENSG00000186106.7_ANKRD46	ANKRD46	ENSG00000186106.7	-1.180	0.416	-2.836	0.005
ENSG00000239739.1_RP11-373E16.4	RP11-373E16.4	ENSG00000239739.1	-0.140	0.049	-2.836	0.005
ENSG00000111785.14_RIC8B	RIC8B	ENSG00000111785.14	-0.237	0.084	-2.837	0.005
ENSG00000105289.10_TJP3	TJP3	ENSG00000105289.10	-0.078	0.027	-2.838	0.005
ENSG00000114098.13_ARMC8	ARMC8	ENSG00000114098.13	-0.732	0.258	-2.842	0.005
ENSG00000139915.13_MDGA2	MDGA2	ENSG00000139915.13	-0.439	0.154	-2.845	0.005
ENSG00000232823.1_RP11-179H18.7	RP11-179H18.7	ENSG00000232823.1	-0.018	0.006	-2.846	0.005
ENSG00000254551.1_RP11-727A23.7	RP11-727A23.7	ENSG00000254551.1	-0.025	0.009	-2.854	0.005
ENSG00000198414.5_TATDN2P1	TATDN2P1	ENSG00000198414.5	-0.007	0.002	-2.856	0.005
ENSG00000241685.3_ARPC1A	ARPC1A	ENSG00000241685.3	-4.708	1.648	-2.857	0.005
ENSG00000105993.10_DNAJB6	DNAJB6	ENSG00000105993.10	-4.006	1.402	-2.858	0.005
ENSG00000182389.13_CACNB4	CACNB4	ENSG00000182389.13	-0.468	0.164	-2.858	0.005
ENSG00000230096.1_RP11-34C15.2	RP11-34C15.2	ENSG00000230096.1	-0.007	0.002	-2.863	0.005
ENSG00000255693.1_RP11-766N7.3	RP11-766N7.3	ENSG00000255693.1	-0.150	0.052	-2.863	0.005
ENSG00000124406.12_ATP8A1	ATP8A1	ENSG00000124406.12	-1.012	0.353	-2.864	0.005
ENSG00000134709.6_HOOK1	HOOK1	ENSG00000134709.6	-0.247	0.086	-2.864	0.005
ENSG00000183814.11_LIN9	LIN9	ENSG00000183814.11	-0.062	0.022	-2.868	0.005
ENSG00000215604.3_ZNF962P	ZNF962P	ENSG00000215604.3	-0.004	0.002	-2.869	0.005
ENSG00000136928.4_GABBR2	GABBR2	ENSG00000136928.4	-3.547	1.236	-2.869	0.005
ENSG00000239666.1_RP11-459F3.6	RP11-459F3.6	ENSG00000239666.1	-0.866	0.302	-2.871	0.005
ENSG00000132170.15_PPARG	PPARG	ENSG00000132170.15	-0.126	0.044	-2.873	0.005
ENSG00000233673.2_ANAPC1P1	ANAPC1P1	ENSG00000233673.2	-0.003	0.001	-2.877	0.005
ENSG00000147655.6_RSPO2	RSPO2	ENSG00000147655.6	-0.857	0.298	-2.878	0.005
ENSG00000143318.8_CASQ1	CASQ1	ENSG00000143318.8	-0.805	0.279	-2.882	0.004
ENSG00000089289.11_IGBP1	IGBP1	ENSG00000089289.11	-1.045	0.362	-2.884	0.004
ENSG00000257272.1_RP11-317N8.3	RP11-317N8.3	ENSG00000257272.1	-0.282	0.098	-2.885	0.004
ENSG00000235736.1_RP1-10C16.1	RP1-10C16.1	ENSG00000235736.1	-0.074	0.025	-2.890	0.004
ENSG00000131773.9_KHDRBS3	KHDRBS3	ENSG00000131773.9	-2.963	1.025	-2.892	0.004
ENSG00000236740.2_RP11-411K7.1	RP11-411K7.1	ENSG00000236740.2	-0.012	0.004	-2.893	0.004
ENSG00000232053.1_AC009784.3	AC009784.3	ENSG00000232053.1	-0.002	0.001	-2.893	0.004
ENSG00000241622.1_RP11-413E6.5	RP11-413E6.5	ENSG00000241622.1	-0.017	0.006	-2.898	0.004
ENSG00000230195.1_AC118138.2	AC118138.2	ENSG00000230195.1	-0.001	0.000	-2.898	0.004
ENSG00000119946.9_CNNM1	CNNM1	ENSG00000119946.9	-0.838	0.289	-2.900	0.004
ENSG00000136531.9_SCN2A	SCN2A	ENSG00000136531.9	-1.269	0.437	-2.901	0.004
ENSG00000253547.2_RP11-959F10.5	RP11-959F10.5	ENSG00000253547.2	-0.020	0.007	-2.904	0.004
ENSG00000172260.8_NEGR1	NEGR1	ENSG00000172260.8	-2.066	0.711	-2.904	0.004
ENSG00000233347.1_ERP29P1	ERP29P1	ENSG00000233347.1	-0.053	0.018	-2.913	0.004
ENSG00000113494.12_PRLR	PRLR	ENSG00000113494.12	-0.013	0.005	-2.913	0.004
ENSG00000215006.4_CHCHD2P2	CHCHD2P2	ENSG00000215006.4	-0.519	0.178	-2.916	0.004
ENSG00000128253.9_RFPL2	RFPL2	ENSG00000128253.9	-0.767	0.263	-2.918	0.004
ENSG00000157766.11_ACAN	ACAN	ENSG00000157766.11	-0.231	0.079	-2.918	0.004

ENSG00000258675.1_RP11-299L17.3	RP11-299L17.3	ENSG00000258675.1	-0.031	0.011	-2.920	0.004
ENSG00000169760.13_NLGN1	NLGN1	ENSG00000169760.13	-0.201	0.069	-2.927	0.004
ENSG00000160714.5_UBE2Q1	UBE2Q1	ENSG00000160714.5	-0.982	0.335	-2.935	0.004
ENSG00000077279.12_DCX	DCX	ENSG00000077279.12	-0.093	0.032	-2.935	0.004
ENSG00000092140.10_G2E3	G2E3	ENSG00000092140.10	-0.110	0.037	-2.937	0.004
ENSG00000186487.12_MYT1L	MYT1L	ENSG00000186487.12	-1.684	0.573	-2.937	0.004
ENSG00000168959.9_GRM5	GRM5	ENSG00000168959.9	-0.558	0.190	-2.937	0.004
ENSG00000257818.1_RP11-1143G9.2	RP11-1143G9.2	ENSG00000257818.1	-0.033	0.011	-2.940	0.004
ENSG00000177182.6_CLVS1	CLVS1	ENSG00000177182.6	-0.293	0.100	-2.948	0.004
ENSG00000261542.1_RP11-16E18.3	RP11-16E18.3	ENSG00000261542.1	-0.095	0.032	-2.950	0.004
ENSG00000171202.2_TMEM126A	TMEM126A	ENSG00000171202.2	-1.326	0.449	-2.952	0.004
ENSG00000262061.1_RP11-1260E13.4	RP11-1260E13.4	ENSG00000262061.1	-0.207	0.070	-2.953	0.004
ENSG00000081189.9_MEF2C	MEF2C	ENSG00000081189.9	-7.417	2.512	-2.953	0.004
ENSG00000233817.1_RP11-168K11.3	RP11-168K11.3	ENSG00000233817.1	-0.163	0.055	-2.956	0.004
ENSG00000258696.1_CTD-2058B24.3	CTD-2058B24.3	ENSG00000258696.1	-0.086	0.029	-2.957	0.004
ENSG00000242140.1_RP11-262M14.2	RP11-262M14.2	ENSG00000242140.1	-16.096	5.440	-2.959	0.004
ENSG00000235667.1_AC009502.3	AC009502.3	ENSG00000235667.1	0.000	0.000	-2.961	0.004
ENSG00000178804.3_H1FOO	H1FOO	ENSG00000178804.3	-0.001	0.000	-2.961	0.004
ENSG00000167916.4_KRT24	KRT24	ENSG00000167916.4	-0.001	0.000	-2.961	0.004
ENSG00000249259.1_RP11-166J5.1	RP11-166J5.1	ENSG00000249259.1	0.000	0.000	-2.961	0.004
ENSG00000256923.1_RP11-449P1.1	RP11-449P1.1	ENSG00000256923.1	-0.004	0.001	-2.961	0.004
ENSG00000235912.1_RP1-159A19.3	RP1-159A19.3	ENSG00000235912.1	0.000	0.000	-2.961	0.004
ENSG00000229298.1_RP11-70L8.1	RP11-70L8.1	ENSG00000229298.1	-0.001	0.000	-2.961	0.004
ENSG00000234296.1_RP13-16H11.7	RP13-16H11.7	ENSG00000234296.1	0.000	0.000	-2.961	0.004
ENSG00000250627.1_TTC39CP1	TTC39CP1	ENSG00000250627.1	0.000	0.000	-2.961	0.004
ENSG00000212316.1_U6	RNU6-1228P	ENSG00000212316.1	0.000	0.000	-2.961	0.004
ENSG00000205133.7_TRIQK	TRIQK	ENSG00000205133.7	-0.369	0.125	-2.961	0.004
ENSG00000251517.1_RP11-109E24.1	RP11-109E24.1	ENSG00000251517.1	0.000	0.000	-2.961	0.004
ENSG00000189241.6_TSPYL1	TSPYL1	ENSG00000189241.6	-6.512	2.199	-2.962	0.003
ENSG00000237837.1_AC159540.3	AC159540.3	ENSG00000237837.1	-0.088	0.030	-2.965	0.003
ENSG00000226828.1_RP11-278H7.1	RP11-278H7.1	ENSG00000226828.1	-0.046	0.015	-2.966	0.003
ENSG00000172795.11_DCP2	DCP2	ENSG00000172795.11	-0.221	0.074	-2.966	0.003
ENSG00000229266.1_POM121L8P	POM121L8P	ENSG00000229266.1	-0.003	0.001	-2.970	0.003
ENSG00000145864.8_GABRB2	GABRB2	ENSG00000145864.8	-1.917	0.645	-2.972	0.003
ENSG00000184368.11_MAP7D2	MAP7D2	ENSG00000184368.11	-2.546	0.855	-2.979	0.003
ENSG00000258484.3_SPESP1	SPESP1	ENSG00000258484.3	-0.105	0.035	-2.980	0.003
ENSG00000242360.2_MetazoaSRP	RN7SL272P	ENSG00000242360.2	-0.020	0.007	-2.986	0.003
ENSG00000234344.1_MEP1AP3	MEP1AP3	ENSG00000234344.1	0.000	0.000	-2.987	0.003
ENSG00000197991.9_PCDH20	PCDH20	ENSG00000197991.9	-0.202	0.067	-3.001	0.003
ENSG00000118200.10_CAMSAP2	CAMSAP2	ENSG00000118200.10	-0.777	0.258	-3.009	0.003
ENSG00000133059.12_DSTYK	DSTYK	ENSG00000133059.12	-0.849	0.281	-3.016	0.003
ENSG00000236333.2_TRHDE-AS1	TRHDE-AS1	ENSG00000236333.2	-0.347	0.115	-3.016	0.003
ENSG00000230702.1_RP11-681L4.1	RP11-681L4.1	ENSG00000230702.1	-0.043	0.014	-3.021	0.003
ENSG00000260042.1_CTD-2034I21.1	CTD-2034I21.1	ENSG00000260042.1	-0.019	0.006	-3.034	0.003
ENSG00000240497.2_RP11-185E8.1	RP11-185E8.1	ENSG00000240497.2	-0.130	0.043	-3.036	0.003
ENSG00000106069.14_CHN2	CHN2	ENSG00000106069.14	-0.809	0.266	-3.044	0.003
ENSG00000171435.8_KSR2	KSR2	ENSG00000171435.8	-0.233	0.076	-3.050	0.003
ENSG00000248306.1_RP11-13E1.3	RP11-13E1.3	ENSG00000248306.1	-0.006	0.002	-3.058	0.003
ENSG00000167232.8_ZNF91	ZNF91	ENSG00000167232.8	-0.691	0.226	-3.060	0.003
ENSG00000198420.5_FAM115A	FAM115A	ENSG00000198420.5	-1.852	0.605	-3.061	0.003
ENSG00000233087.4_AC073869.1	AC073869.1	ENSG00000233087.4	-0.063	0.021	-3.064	0.003
ENSG00000166963.8_MAP1A	MAP1A	ENSG00000166963.8	-7.799	2.542	-3.068	0.003
ENSG00000224531.3_C6orf228	C6orf228	ENSG00000224531.3	-0.736	0.240	-3.072	0.002
ENSG00000227060.2_LINC00629	LINC00629	ENSG00000227060.2	-0.050	0.016	-3.077	0.002
ENSG00000241838.2_LA16c-3G11.7	LA16c-3G11.7	ENSG00000241838.2	-0.019	0.006	-3.078	0.002
ENSG00000100722.13_ZC3H14	ZC3H14	ENSG00000100722.13	-0.541	0.175	-3.099	0.002
ENSG00000198300.7_PEG3	PEG3	ENSG00000198300.7	-1.722	0.551	-3.123	0.002
ENSG00000253790.1_RP11-44K6.5	RP11-44K6.5	ENSG00000253790.1	-0.019	0.006	-3.123	0.002
ENSG00000198477.3_ZNF280B	ZNF280B	ENSG00000198477.3	-0.158	0.051	-3.126	0.002
ENSG00000171984.10_C20orf196	C20orf196	ENSG00000171984.10	-0.528	0.169	-3.132	0.002

ENSG00000108861.4_DUSP3	DUSP3	ENSG00000108861.4	-3.372	1.075	-3.136	0.002
ENSG00000144619.10_CNTN4	CNTN4	ENSG00000144619.10	-0.400	0.127	-3.141	0.002
ENSG00000223754.1_AC008073.9	AC008073.9	ENSG00000223754.1	-0.011	0.003	-3.144	0.002
ENSG00000198929.8_NOS1AP	NOS1AP	ENSG00000198929.8	-1.226	0.388	-3.161	0.002
ENSG00000166783.14_KIAA0430	KIAA0430	ENSG00000166783.14	-1.260	0.398	-3.164	0.002
ENSG00000217026.3_RPL10P1	RPL10P1	ENSG00000217026.3	-0.034	0.011	-3.170	0.002
ENSG00000070961.10_ATP2B1	ATP2B1	ENSG00000070961.10	-4.099	1.291	-3.174	0.002
ENSG00000156976.10_EIF4A2	EIF4A2	ENSG00000156976.10	-23.928	7.537	-3.175	0.002
ENSG00000145354.5_CISD2	CISD2	ENSG00000145354.5	-0.699	0.220	-3.178	0.002
ENSG00000138161.8_CUZD1	CUZD1	ENSG00000138161.8	-0.085	0.027	-3.184	0.002
ENSG00000109670.8_FBXW7	FBXW7	ENSG00000109670.8	-4.676	1.463	-3.196	0.002
ENSG00000223530.1_AC012506.1	AC012506.1	ENSG00000223530.1	-0.001	0.000	-3.209	0.002
ENSG00000215515.2_IFIT1P1	IFIT1P1	ENSG00000215515.2	-0.014	0.004	-3.211	0.002
ENSG00000023734.6_STRAP	STRAP	ENSG00000023734.6	-3.021	0.940	-3.213	0.002
ENSG00000165194.10_PCDH19	PCDH19	ENSG00000165194.10	-0.720	0.223	-3.229	0.001
ENSG00000123119.7_NECAB1	NECAB1	ENSG00000123119.7	-3.251	1.005	-3.236	0.001
ENSG00000089692.3_LAG3	LAG3	ENSG00000089692.3	-0.126	0.039	-3.245	0.001
ENSG00000151292.13_CSNK1G3	CSNK1G3	ENSG00000151292.13	-0.250	0.077	-3.249	0.001
ENSG00000157216.10_SSBP3	SSBP3	ENSG00000157216.10	-6.688	2.058	-3.250	0.001
ENSG00000111850.5_C6orf162	C6orf162	ENSG00000111850.5	-0.312	0.096	-3.252	0.001
ENSG00000149970.10_CNKSR2	CNKSR2	ENSG00000149970.10	-1.566	0.480	-3.265	0.001
ENSG00000253213.1_RP11-546B8.3	RP11-546B8.3	ENSG00000253213.1	-0.023	0.007	-3.268	0.001
ENSG00000095627.5_TDRD1	TDRD1	ENSG00000095627.5	-0.027	0.008	-3.269	0.001
ENSG00000237765.2_FAM200B	FAM200B	ENSG00000237765.2	-2.332	0.708	-3.293	0.001
ENSG00000236502.1_SIX3-AS1	SIX3-AS1	ENSG00000236502.1	-0.358	0.108	-3.311	0.001
ENSG00000146383.7_TAAR6	TAAR6	ENSG00000146383.7	-0.007	0.002	-3.333	0.001
ENSG00000204899.5_MZT1	MZT1	ENSG00000204899.5	-0.711	0.213	-3.337	0.001
ENSG00000119640.4_ACYP1	ACYP1	ENSG00000119640.4	-2.422	0.721	-3.359	0.001
ENSG00000138814.12_PPP3CA	PPP3CA	ENSG00000138814.12	-9.348	2.782	-3.360	0.001
ENSG00000257159.1_RP11-58A17.3	RP11-58A17.3	ENSG00000257159.1	-0.087	0.026	-3.360	0.001
ENSG00000174453.5_VWC2L	VWC2L	ENSG00000174453.5	-0.127	0.038	-3.372	0.001
ENSG00000196227.6_FAM217B	FAM217B	ENSG00000196227.6	-0.973	0.284	-3.421	0.001
ENSG00000152582.8_SPEF2	SPEF2	ENSG00000152582.8	-0.287	0.083	-3.447	0.001
ENSG00000262633.1_RP11-156P1.2	RP11-156P1.2	ENSG00000262633.1	-0.022	0.006	-3.513	0.001
ENSG00000234710.1_AC060834.3	AC060834.3	ENSG00000234710.1	-0.016	0.005	-3.546	0.001
ENSG00000178662.11_CSRNP3	CSRNP3	ENSG00000178662.11	-0.372	0.105	-3.557	0.000
ENSG00000035141.3_FAM136A	FAM136A	ENSG00000035141.3	-0.598	0.168	-3.559	0.000
ENSG00000248150.1_RP1-167G20.1	RP1-167G20.1	ENSG00000248150.1	-0.025	0.007	-3.598	0.000
ENSG00000165572.6_KBTBD6	KBTBD6	ENSG00000165572.6	-0.521	0.140	-3.713	0.000
ENSG00000244356.2_MetazoaSRP	RN7SL398P	ENSG00000244356.2	-0.041	0.010	-3.992	0.000
ENSG00000152193.7_RNF219	RNF219	ENSG00000152193.7	-0.349	0.087	-3.999	0.000
ENSG00000261098.1_RP11-819C21.1	RP11-819C21.1	ENSG00000261098.1	-0.155	0.038	-4.089	0.000
