# Supplementary material for: Loneliness 5 years ante-mortem is associated with disease-related differential gene expression in postmortem dorsolateral prefrontal cortex
Source: Transl Psychiatry. 2018 Jan 10;8:2. doi: 10.1038/s41398-017-0086-2 (PMC5802527; doi:10.1038/s41398-017-0086-2)
Supplement: Supplementary file 2 — Supplemental Table 2 [file 41398_2017_86_MOESM2_ESM.pdf]

Collection	Upregulated Sets (sorted by positive Normalized Enrichment S	SIZE	ES	NES	NOM p-val	FDR q-val	FWER p-val	RANK AT MAX	LEADING EDGE
ChemGenPerturb	BLALOCK_ALZHEIMERS_DISEASE_UP	198	0.35	4.28	0.000	0.000	0.000	1535	tags=87%, list=55%, signal=179%
CancerModules	MODULE_5	90	0.43	3.94	0.000	0.000	0.000	1067	tags=76%, list=38%, signal=118%
ChemGenPerturb	MILI_PSEUDOPODIA_HAPTOTAXIS_DN	98	0.38	3.60	0.000	0.000	0.000	1228	tags=79%, list=44%, signal=135%
ChemGenPerturb	REN_ALVEOLAR_RHABDOMYOSARCOMA_DN	65	0.43	3.55	0.000	0.000	0.000	1307	tags=88%, list=47%, signal=161%
CancerModules	MODULE_23	89	0.37	3.55	0.000	0.000	0.000	874	tags=63%, list=31%, signal=89%
CancerModules	MODULE_88	112	0.33	3.42	0.000	0.000	0.000	882	tags=60%, list=31%, signal=84%
CancerModules	MODULE_60	79	0.39	3.38	0.000	0.000	0.000	1425	tags=87%, list=51%, signal=173%
GO	GO_VASCULATURE_DEVELOPMENT	86	0.37	3.33	0.000	0.000	0.000	1545	tags=91%, list=55%, signal=196%
CancerModules	MODULE_55	108	0.33	3.29	0.000	0.000	0.000	882	tags=59%, list=31%, signal=83%
CancerModules	MODULE_38	89	0.36	3.29	0.000	0.000	0.000	1029	tags=67%, list=37%, signal=103%
CancerModules	MODULE_6	67	0.40	3.29	0.000	0.000	0.000	1096	tags=75%, list=39%, signal=120%
CancerModules	MODULE_84	76	0.38	3.26	0.000	0.000	0.000	1029	tags=70%, list=37%, signal=107%
ChemGenPerturb	LEE_BMP2_TARGETS_UP	112	0.31	3.24	0.000	0.000	0.000	1116	tags=67%, list=40%, signal=107%
CancerModules	MODULE_1	69	0.38	3.24	0.000	0.000	0.000	1102	tags=74%, list=39%, signal=119%
GO	GO_CELL_SURFACE	115	0.32	3.24	0.000	0.000	0.000	1008	tags=63%, list=36%, signal=95%
ChemGenPerturb	CHEN_METABOLIC_SYNDROM_NETWORK	172	0.27	3.14	0.000	0.000	0.000	871	tags=52%, list=31%, signal=71%
GO	GO_BLOOD_VESSEL_MORPHOGENESIS	64	0.39	3.12	0.000	0.000	0.000	1545	tags=92%, list=55%, signal=201%
GO	GO_IMMUNE_RESPONSE	90	0.34	3.11	0.000	0.000	0.000	1469	tags=84%, list=52%, signal=172%
Immunogenic	GSE21360_NAIVE_VS_PRIMARY_MEMORY_CD8_TCELL_UP	28	0.54	3.10	0.000	0.001	0.001	1230	tags=96%, list=44%, signal=170%
ChemGenPerturb	LEI_MYB_TARGETS	44	0.44	3.09	0.000	0.000	0.000	1212	tags=84%, list=43%, signal=146%
ChemGenPerturb	BLALOCK_ALZHEIMERS_DISEASE_INCIPIENT_UP	56	0.41	3.08	0.000	0.000	0.000	845	tags=64%, list=30%, signal=90%
GO	GO_CIRCULATORY_SYSTEM_DEVELOPMENT	130	0.29	3.05	0.000	0.000	0.000	1137	tags=65%, list=41%, signal=104%
GO	GO_EXTRACELLULAR_STRUCTURE_ORGANIZATION	45	0.44	3.05	0.000	0.000	0.000	1025	tags=78%, list=37%, signal=121%
ChemGenPerturb	MEISSNER_BRAIN_HCP_WITH_H3K4ME3_AND_H3K27ME3	155	0.27	3.04	0.000	0.000	0.000	1058	tags=59%, list=38%, signal=90%
ChemGenPerturb	JOHNSTONE_PARVB_TARGETS_3_UP	60	0.39	3.03	0.000	0.000	0.000	1081	tags=73%, list=39%, signal=117%
CancerModules	MODULE_47	50	0.41	3.03	0.000	0.000	0.000	616	tags=56%, list=22%, signal=71%
GO	GO_EXTRACELLULAR_SPACE	132	0.28	3.02	0.000	0.000	0.000	1098	tags=63%, list=39%, signal=99%
GO	GO_RESPONSE_TO_WOUNDING	85	0.32	3.02	0.000	0.000	0.000	1100	tags=67%, list=39%, signal=107%
GO	GO_ANGIOGENESIS	54	0.39	3.02	0.000	0.000	0.000	1539	tags=93%, list=55%, signal=202%
ChemGenPerturb	SANSOM_APC_TARGETS_DN	52	0.40	3.01	0.000	0.000	0.000	1051	tags=75%, list=38%, signal=118%
GO	GO_LEUKOCYTE_MIGRATION	24	0.56	3.01	0.000	0.000	0.000	1256	tags=100%, list=45%, signal=180%
ChemGenPerturb	HOSHIDA_LIVER_CANCER_SUBCLASS_S3	33	0.49	2.99	0.000	0.000	0.000	1431	tags=100%, list=51%, signal=202%
Hallmark	HALLMARK_EPITHELIAL_MESENCHYMAL_TRANSITION	37	0.46	2.97	0.000	0.000	0.000	941	tags=76%, list=34%, signal=112%
ChemGenPerturb	LANDIS_ERBB2_BREAST_TUMORS_324_DN	25	0.54	2.96	0.000	0.000	0.000	1040	tags=88%, list=37%, signal=139%
ChemGenPerturb	MCBRYAN_PUBERTAL_TGFB1_TARGETS_UP	23	0.54	2.96	0.000	0.000	0.000	868	tags=83%, list=31%, signal=119%
GO	GO_ANTIGEN_BINDING	22	0.56	2.95	0.000	0.000	0.001	761	tags=82%, list=27%, signal=111%
Immunogenic	GSE16266_CTRL_VS_LPS_STIM_MEF_UP	35	0.46	2.94	0.000	0.004	0.006	618	tags=60%, list=22%, signal=76%
CancerModules	MODULE_3	44	0.42	2.92	0.000	0.000	0.000	1096	tags=77%, list=39%, signal=125%
GO	GO_ANCHORING_JUNCTION	71	0.33	2.90	0.000	0.000	0.001	1100	tags=68%, list=39%, signal=109%
CancerModules	MODULE_2	70	0.35	2.90	0.000	0.000	0.000	1102	tags=70%, list=39%, signal=113%
GO	GO_WOUND_HEALING	74	0.34	2.90	0.000	0.000	0.001	1100	tags=69%, list=39%, signal=110%
GO	GO_IMMUNE_SYSTEM_PROCESS	185	0.24	2.90	0.000	0.000	0.001	1516	tags=77%, list=54%, signal=156%
Immunogenic	GSE29164_CD8_TCELL_VS_CD8_TCELL_AND_IL12_TREATED_M	28	0.50	2.90	0.000	0.004	0.009	890	tags=79%, list=32%, signal=114%
ChemGenPerturb	DEBIASI_APOPTOSIS_BY_REOVIRUS_INFECTION_DN	46	0.40	2.90	0.000	0.000	0.000	1081	tags=74%, list=39%, signal=118%
Immunogenic	GSE2405_0H_VS_3H_A_PHAGOCYTOPHILUM_STIM_NEUTROPH	33	0.47	2.90	0.002	0.003	0.009	728	tags=67%, list=26%, signal=89%
GO	GO_CELL_SUBSTRATE_JUNCTION	57	0.37	2.89	0.000	0.000	0.001	1302	tags=81%, list=46%, signal=148%
ChemGenPerturb	MOOTHA_PGC	44	0.41	2.87	0.000	0.000	0.000	335	tags=45%, list=12%, signal=51%
ChemGenPerturb	LU_AGING_BRAIN_UP	42	0.42	2.85	0.000	0.000	0.001	1437	tags=93%, list=51%, signal=188%
ChemGenPerturb	VERHAAK_GLIOBLASTOMA_MESENCHYMAL	40	0.44	2.85	0.000	0.000	0.002	1457	tags=95%, list=52%, signal=195%
ChemGenPerturb	GRAESSMANN_APOPTOSIS_BY_DOXORUBICIN_UP	116	0.28	2.84	0.000	0.000	0.002	1519	tags=80%, list=54%, signal=168%
GO	GO_SIDE_OF_MEMBRANE	59	0.35	2.84	0.000	0.000	0.003	1307	tags=81%, list=47%, signal=149%
ChemGenPerturb	LIU_PROSTATE_CANCER_DN	81	0.32	2.82	0.000	0.000	0.002	1062	tags=64%, list=38%, signal=100%

ChemGenPerturb	LEE_LIVER_CANCER_ACOX1_DN	11	0.73	2.82	0.000	0.000	0.002	583	tags=91%, list=21%, signal=114%
ChemGenPerturb	FEVR_CTNNB1_TARGETS_UP	72	0.32	2.80	0.000	0.000	0.002	868	tags=57%, list=31%, signal=80%
ChemGenPerturb	CHICAS_RB1_TARGETS_CONFLUENT	98	0.29	2.79	0.000	0.000	0.002	470	tags=39%, list=17%, signal=45%
GO	GO_BLOOD_MICROPARTICLE	19	0.57	2.79	0.000	0.000	0.004	1207	tags=100%, list=43%, signal=175%
CancerModules	MODULE_52	58	0.36	2.78	0.000	0.000	0.000	873	tags=62%, list=31%, signal=88%
Immunogenic	GSE22886_NAIVE_CD4_TCELL_VS_NEUTROPHIL_DN	18	0.58	2.78	0.000	0.007	0.026	1044	tags=94%, list=37%, signal=150%
GO	GO_RECEPTOR_ACTIVITY	164	0.24	2.77	0.000	0.000	0.006	887	tags=51%, list=32%, signal=70%
ChemGenPerturb	BERTUCCI_MEDULLARY_VS_DUCTAL_BREAST_CANCER_DN	34	0.45	2.76	0.000	0.000	0.002	484	tags=56%, list=17%, signal=67%
ChemGenPerturb	LEIN_ASTROCYTE_MARKERS	14	0.64	2.76	0.000	0.000	0.003	1016	tags=100%, list=36%, signal=156%
GO	GO_CELL_JUNCTION_ASSEMBLY	21	0.54	2.76	0.000	0.000	0.006	1160	tags=95%, list=41%, signal=161%
GO	GO_VACUOLE	135	0.26	2.75	0.000	0.000	0.006	1190	tags=64%, list=42%, signal=107%
Immunogenic	GSE21380_TFH_VS_GERMINAL_CENTER_TFH_CD4_TCELL_UP	29	0.47	2.75	0.000	0.007	0.032	606	tags=62%, list=22%, signal=78%
Immunogenic	GSE3337_CTRL_VS_4H_IFNG_IN_CD8POS_DC_UP	25	0.50	2.75	0.000	0.006	0.032	1130	tags=88%, list=40%, signal=146%
ChemGenPerturb	BROWNE_HCMV_INFECTION_18HR_DN	28	0.49	2.72	0.000	0.000	0.004	416	tags=57%, list=15%, signal=66%
Immunogenic	GSE40666_UNTREATED_VS_IFNA_STIM_EFFECTOR_CD8_TCELL_	29	0.47	2.71	0.000	0.008	0.049	628	tags=62%, list=22%, signal=79%
ChemGenPerturb	VERHAAK_GLIOBLASTOMA_CLASSICAL	36	0.43	2.70	0.002	0.000	0.006	1545	tags=97%, list=55%, signal=214%
GO	GO_SMALL_MOLECULE_CATABOLIC_PROCESS	31	0.45	2.70	0.000	0.001	0.011	1016	tags=77%, list=36%, signal=120%
GO	GO_EXTERNAL_SIDE_OF_PLASMA_MEMBRANE	37	0.41	2.70	0.000	0.001	0.011	766	tags=65%, list=27%, signal=88%
GO	GO_DEFENSE_RESPONSE	105	0.27	2.69	0.000	0.001	0.014	1430	tags=75%, list=51%, signal=148%
GO	GO_POSITIVE_REGULATION_OF_TRANSMEMBRANE_RECEPTOR_	15	0.61	2.69	0.000	0.001	0.014	771	tags=87%, list=28%, signal=119%
GO	GO_LYTIC_VACUOLE	72	0.31	2.68	0.000	0.001	0.015	1111	tags=67%, list=40%, signal=108%
GO	GO_EXTRACELLULAR_MATRIX_COMPONENT	23	0.51	2.68	0.000	0.001	0.015	895	tags=78%, list=32%, signal=114%
GO	GO_TRANSMEMBRANE_RECEPTOR_PROTEIN_SERINE_THREONII	26	0.47	2.68	0.000	0.001	0.015	753	tags=69%, list=27%, signal=94%
CancerModules	MODULE_321	20	0.54	2.67	0.000	0.000	0.000	597	tags=70%, list=21%, signal=88%
Canonical	KEGG_CYTOKINE_CYTOKINE_RECEPTOR_INTERACTION	29	0.45	2.67	0.000	0.002	0.002	1117	tags=83%, list=40%, signal=136%
ChemGenPerturb	SWEET_LUNG_CANCER_KRAS_DN	79	0.31	2.66	0.000	0.000	0.008	1049	tags=63%, list=37%, signal=98%
GO	GO_IMMUNE_EFFECTOR_PROCESS	47	0.36	2.66	0.000	0.001	0.020	1171	tags=74%, list=42%, signal=126%
ChemGenPerturb	ACOSTA_PROLIFERATION_INDEPENDENT_MYC_TARGETS_DN	21	0.51	2.66	0.000	0.000	0.009	617	tags=67%, list=22%, signal=85%
CancerModules	MODULE_79	24	0.49	2.65	0.000	0.000	0.000	1008	tags=83%, list=36%, signal=129%
GO	GO_REGULATION_OF_BODY_FLUID_LEVELS	72	0.32	2.64	0.000	0.001	0.027	421	tags=40%, list=15%, signal=46%
ChemGenPerturb	PICCALUGA_ANGIOIMMUNOBLASTIC_LYMPHOMA_UP	35	0.41	2.64	0.000	0.000	0.011	1322	tags=86%, list=47%, signal=160%
Immunogenic	GSE23568_ID3_TRANSDUCED_VS_ID3_KO_CD8_TCELL_UP	30	0.45	2.64	0.000	0.012	0.083	936	tags=73%, list=33%, signal=109%
ChemGenPerturb	SMID_BREAST_CANCER_LUMINAL_B_DN	84	0.29	2.63	0.000	0.000	0.011	1505	tags=81%, list=54%, signal=170%
ChemGenPerturb	BRUINS_UVC_RESPONSE_VIA_TP53_GROUP_B	67	0.32	2.62	0.000	0.001	0.014	1308	tags=76%, list=47%, signal=139%
CancerModules	MODULE_118	51	0.35	2.60	0.000	0.000	0.001	1062	tags=69%, list=38%, signal=109%
Immunogenic	GSE19198_1H_VS_24H_IL21_TREATED_TCELL_UP	39	0.39	2.60	0.000	0.015	0.113	767	tags=62%, list=27%, signal=84%
Immunogenic	GSE9988_ANTI_TREM1_VS_VEHICLE_TREATED_MONOCYTES_DI	31	0.43	2.59	0.000	0.015	0.118	1115	tags=81%, list=40%, signal=132%
GO	GO_REGULATION_OF_OSSIFICATION	27	0.45	2.59	0.000	0.002	0.048	1069	tags=81%, list=38%, signal=131%
Immunogenic	GSE360_DC_VS_MAC_B_MALAYI_HIGH_DOSE_UP	27	0.45	2.59	0.000	0.015	0.130	878	tags=74%, list=31%, signal=107%
GO	GO_SMALL_MOLECULE_METABOLIC_PROCESS	190	0.22	2.59	0.000	0.002	0.048	1433	tags=70%, list=51%, signal=134%
ChemGenPerturb	DELACROIX_RARG_BOUND_MEF	39	0.39	2.57	0.000	0.001	0.028	1526	tags=92%, list=54%, signal=200%
ChemGenPerturb	PETROVA_ENDOTHELIUM_LYMPHATIC_VS_BLOOD_DN	32	0.43	2.57	0.000	0.001	0.028	643	tags=59%, list=23%, signal=76%
ChemGenPerturb	CAIRO_LIVER_DEVELOPMENT_DN	30	0.43	2.57	0.000	0.001	0.028	1128	tags=80%, list=40%, signal=133%
GO	GO_ORGANIC_ACID_METABOLIC_PROCESS	101	0.26	2.57	0.002	0.002	0.057	1426	tags=75%, list=51%, signal=148%
Immunogenic	GSE2405_S_AUREUS_VS_A_PHAGOCYTOPHILUM_NEUTROPHIL_	30	0.44	2.56	0.000	0.016	0.155	1256	tags=87%, list=45%, signal=155%
Immunogenic	GSE39110_UNTREATED_VS_IL2_TREATED_CD8_TCELL_DAY6_P(	28	0.45	2.56	0.000	0.016	0.159	1282	tags=89%, list=46%, signal=163%
CancerModules	MODULE_24	57	0.33	2.56	0.000	0.000	0.003	878	tags=60%, list=31%, signal=85%
ChemGenPerturb	DELYS_THYROID_CANCER_UP	66	0.31	2.56	0.000	0.001	0.031	1137	tags=67%, list=41%, signal=110%
GO	GO_HEMOSTASIS	46	0.35	2.55	0.000	0.003	0.066	1173	tags=74%, list=42%, signal=125%
GO	GO_EXTRACELLULAR_MATRIX	62	0.33	2.54	0.000	0.003	0.069	1240	tags=74%, list=44%, signal=130%
CancerModules	MODULE_170	23	0.48	2.54	0.000	0.000	0.004	1008	tags=83%, list=36%, signal=128%
GO	GO_INNATE_IMMUNE_RESPONSE	44	0.37	2.54	0.000	0.002	0.069	1539	tags=91%, list=55%, signal=199%
ChemGenPerturb	GRAESSMANN_RESPONSE_TO_MC_AND_DOXORUBICIN_UP	69	0.30	2.54	0.000	0.001	0.039	1519	tags=83%, list=54%, signal=176%

Immunogenic	GSE29618_BCELL_VS_MDC_DAY7_FLU_VACCINE_DN	44	0.36	2.53	0.000	0.018	0.200	1322	tags=82%, list=47%, signal=153%
CancerModules	MODULE_44	49	0.35	2.53	0.000	0.000	0.005	878	tags=63%, list=31%, signal=91%
GO	GO_ENDOCYTOSIS	75	0.29	2.53	0.000	0.003	0.072	1122	tags=65%, list=40%, signal=106%
Immunogenic	GSE24634_IL4_VS_CTRL_TREATED_NAIVE_CD4_TCELL_DAY5_UI	36	0.39	2.53	0.000	0.018	0.206	926	tags=67%, list=33%, signal=98%
ChemGenPerturb	WOO_LIVER_CANCER_RECURRENCE_DN	18	0.53	2.52	0.000	0.002	0.047	1088	tags=89%, list=39%, signal=144%
Immunogenic	GSE42021_CD24LO_TREG_VS_CD24LO_TCONV_THYMUS_UP	12	0.64	2.52	0.000	0.017	0.211	284	tags=67%, list=10%, signal=74%
Immunogenic	GSE29618_PDC_VS_MDC_DAY7_FLU_VACCINE_DN	30	0.42	2.52	0.000	0.017	0.216	1354	tags=90%, list=48%, signal=172%
GO	GO_TRANSMEMBRANE_RECEPTOR_PROTEIN_KINASE_ACTIVITY	17	0.55	2.51	0.000	0.003	0.080	820	tags=82%, list=29%, signal=116%
Immunogenic	GSE21360_NAIVE_VS_SECONDARY_MEMORY_CD8_TCELL_UP	20	0.50	2.51	0.000	0.016	0.222	1139	tags=90%, list=41%, signal=151%
CancerModules	MODULE_15	54	0.33	2.51	0.002	0.000	0.006	873	tags=59%, list=31%, signal=84%
CancerModules	MODULE_128	23	0.48	2.51	0.000	0.000	0.006	1008	tags=83%, list=36%, signal=128%
Immunogenic	GSE34156_UNTREATED_VS_6H_TLR1_TLR2_LIGAND_TREATED_	25	0.45	2.50	0.000	0.018	0.254	1016	tags=80%, list=36%, signal=124%
GO	GO_CELL_JUNCTION_ORGANIZATION	31	0.42	2.50	0.000	0.003	0.089	1160	tags=81%, list=41%, signal=136%
Immunogenic	GSE29618_BCELL_VS_MDC_DN	39	0.39	2.49	0.000	0.019	0.271	1322	tags=85%, list=47%, signal=158%
Immunogenic	GSE24634_TREG_VS_TCONV_POST_DAY10_IL4_CONVERSION_D	27	0.45	2.49	0.000	0.018	0.273	1016	tags=78%, list=36%, signal=121%
GO	GO_CELL_MOTILITY	104	0.25	2.49	0.000	0.003	0.096	1194	tags=64%, list=43%, signal=108%
GO	GO_LOCOMOTION	141	0.23	2.47	0.000	0.004	0.107	1194	tags=62%, list=43%, signal=102%
GO	GO_CELL_CELL_JUNCTION_ASSEMBLY	13	0.59	2.47	0.000	0.003	0.107	1160	tags=100%, list=41%, signal=170%
GO	GO_PROTEIN_ACTIVATION_CASCADE	14	0.57	2.47	0.000	0.004	0.112	880	tags=86%, list=31%, signal=124%
CancerModules	MODULE_45	81	0.28	2.47	0.002	0.001	0.010	1425	tags=77%, list=51%, signal=151%
Immunogenic	GSE15767_MED_VS_SCS_MAC_LN_UP	36	0.39	2.47	0.000	0.022	0.338	1166	tags=78%, list=42%, signal=132%
GO	GO_SIGNALING_RECEPTOR_ACTIVITY	131	0.23	2.46	0.000	0.003	0.112	730	tags=44%, list=26%, signal=57%
GO	GO_OXIDATION_REDUCTION_PROCESS	76	0.28	2.46	0.000	0.003	0.112	993	tags=59%, list=35%, signal=89%
Immunogenic	GSE21360_TERTIARY_VS_QUATERNARY_MEMORY_CD8_TCELL_	30	0.41	2.46	0.000	0.022	0.346	993	tags=73%, list=35%, signal=112%
Immunogenic	GSE23568_CTRL_VS_ID3_TRANSDUCED_CD8_TCELL_UP	27	0.43	2.46	0.000	0.022	0.366	869	tags=70%, list=31%, signal=101%
Immunogenic	GSE9878_CTRL_VS_EBF_TRANSDUCED_PAX5_KO_PRO_BCELL_U	27	0.43	2.45	0.000	0.022	0.373	1185	tags=81%, list=42%, signal=140%
Immunogenic	GSE27670_BLIMP1_VS_LMP1_TRANSDUCED_GC_BCELL_UP	36	0.38	2.45	0.002	0.022	0.385	1523	tags=92%, list=54%, signal=198%
Immunogenic	GSE25123_WT_VS_PPARG_KO_MACROPHAGE_ROSIGLITAZONE_	21	0.48	2.44	0.000	0.023	0.402	1246	tags=90%, list=44%, signal=162%
Immunogenic	GSE20715_WT_VS_TLR4_KO_LUNG_DN	25	0.44	2.44	0.000	0.022	0.405	1081	tags=80%, list=39%, signal=129%
GO	GO_BASEMENT_MEMBRANE	18	0.52	2.44	0.000	0.004	0.140	866	tags=78%, list=31%, signal=112%
GO	GO_RECEPTOR_MEDIATED_ENDOCYTOSIS	35	0.39	2.43	0.000	0.004	0.143	1089	tags=74%, list=39%, signal=120%
GO	GO_SIGNAL_TRANSDUCER_ACTIVITY	176	0.21	2.43	0.000	0.004	0.148	820	tags=45%, list=29%, signal=59%
GO	GO_DEFENSE_RESPONSE_TO_OTHER_ORGANISM	45	0.34	2.42	0.000	0.004	0.155	1189	tags=73%, list=42%, signal=125%
Immunogenic	GSE3337_4H_VS_16H_IFNG_IN_CD8POS_DC_DN	39	0.37	2.42	0.002	0.025	0.451	1087	tags=72%, list=39%, signal=116%
Immunogenic	GSE24634_TEFF_VS_TCONV_DAY7_IN_CULTURE_DN	26	0.44	2.42	0.000	0.024	0.456	997	tags=77%, list=36%, signal=118%
Immunogenic	GSE14308_TH1_VS_NATURAL_TREG_DN	15	0.56	2.42	0.000	0.024	0.465	1095	tags=93%, list=39%, signal=152%
Oncogenic	PRC2_EED_UP.V1_DN	23	0.46	2.41	0.000	0.022	0.017	1015	tags=78%, list=36%, signal=122%
CancerModules	MODULE_324	23	0.46	2.41	0.000	0.001	0.017	1308	tags=91%, list=47%, signal=170%
Canonical	NABA_MATRISOME	109	0.24	2.41	0.000	0.012	0.019	1387	tags=72%, list=50%, signal=136%
GO	GO_POSITIVE_REGULATION_OF_RESPONSE_TO_STIMULUS	205	0.19	2.41	0.000	0.005	0.184	771	tags=41%, list=28%, signal=53%
GO	GO_POSITIVE_REGULATION_OF_CELL_PROLIFERATION	88	0.26	2.40	0.000	0.005	0.188	1546	tags=80%, list=55%, signal=172%
Immunogenic	GSE40666_UNTREATED_VS_IFNA_STIM_STAT4_KO_EFFECTOR_(	20	0.48	2.40	0.000	0.027	0.518	1067	tags=85%, list=38%, signal=136%
Immunogenic	GSE23505_UNTREATED_VS_4DAY_IL6_IL1_IL23_TREATED_CD4_	25	0.44	2.40	0.000	0.027	0.528	1098	tags=80%, list=39%, signal=130%
Immunogenic	GSE7831_1H_VS_4H_INFLUENZA_STIM_PDC_UP	31	0.41	2.39	0.000	0.027	0.532	1122	tags=77%, list=40%, signal=128%
GO	GO_PROTEINACEOUS_EXTRACELLULAR_MATRIX	50	0.33	2.39	0.002	0.006	0.221	895	tags=60%, list=32%, signal=87%
GO	GO_REGULATION_OF_MAPK_CASCADE	72	0.28	2.39	0.000	0.006	0.225	792	tags=51%, list=28%, signal=70%
Immunogenic	GSE34156_TLR1_TLR2_LIGAND_VS_NOD2_AND_TLR1_TLR2_LIG	30	0.39	2.38	0.000	0.028	0.557	1016	tags=73%, list=36%, signal=114%
Hallmark	HALLMARK_MYOGENESIS	33	0.38	2.38	0.000	0.004	0.005	973	tags=70%, list=35%, signal=106%
CancerModules	MODULE_33	55	0.31	2.38	0.000	0.001	0.024	1078	tags=65%, list=38%, signal=104%
Immunogenic	GSE44649_WT_VS_MIR155_KO_NAIVE_CD8_TCELL_UP	30	0.39	2.38	0.000	0.029	0.580	723	tags=60%, list=26%, signal=80%
Immunogenic	GSE23568_CTRL_TRANSDUCED_VS_WT_CD8_TCELL_DN	28	0.41	2.38	0.000	0.029	0.585	936	tags=71%, list=33%, signal=106%
Immunogenic	GSE18804_SPLEEN_MACROPHAGE_VS_COLON_TUMORAL_MAC	28	0.42	2.38	0.000	0.028	0.585	1553	tags=96%, list=55%, signal=214%
GO	GO_TISSUE_DEVELOPMENT	200	0.19	2.37	0.000	0.007	0.245	1439	tags=69%, list=51%, signal=131%

CancerModules	MODULE_75	41	0.35	2.37	0.000	0.001	0.025	1379	tags=83%, list=49%, signal=161%
Immunogenic	GSE2405_HEAT_KILLED_LYSATE_VS_LIVE_A_PHAGOCYTOPHILUI	31	0.40	2.37	0.000	0.029	0.608	743	tags=61%, list=27%, signal=82%
Immunogenic	GSE6092_IFNG_VS_IFNG_AND_B_BURGDORFERI_INF_ENDOTHE	32	0.38	2.37	0.000	0.029	0.620	1016	tags=72%, list=36%, signal=111%
GO	GO_ANATOMICAL_STRUCTURE_MATURATION	12	0.60	2.37	0.000	0.007	0.259	544	tags=75%, list=19%, signal=93%
Immunogenic	GSE34156_UNTREATED_VS_24H_NOD2_LIGAND_TREATED_MO	24	0.43	2.36	0.000	0.029	0.635	1279	tags=88%, list=46%, signal=160%
Immunogenic	GSE19401_PAM2CSK4_VS_RETINOIC_ACID_AND_PAM2CSK4_ST	33	0.38	2.36	0.000	0.029	0.640	945	tags=67%, list=34%, signal=99%
GO	GO_PLATELET_ACTIVATION	26	0.41	2.36	0.000	0.007	0.264	1160	tags=81%, list=41%, signal=137%
GO	GO_CARBOHYDRATE_DERIVATIVE_BIOSYNTHETIC_PROCESS	59	0.30	2.36	0.000	0.007	0.271	834	tags=54%, list=30%, signal=76%
GO	GO_REGULATION_OF_VASCULATURE_DEVELOPMENT	39	0.36	2.36	0.002	0.007	0.274	438	tags=44%, list=16%, signal=51%
GO	GO_RESPONSE_TO_EXTERNAL_STIMULUS	191	0.19	2.35	0.000	0.007	0.279	880	tags=46%, list=31%, signal=62%
Immunogenic	GSE30083_SP3_VS_SP4_THYMOCYTE_DN	29	0.40	2.34	0.000	0.033	0.697	1502	tags=93%, list=54%, signal=199%
Immunogenic	GSE6269_E_COLI_VS_STREP_PNEUMO_INF_PBMC_DN	18	0.49	2.34	0.000	0.032	0.700	1169	tags=89%, list=42%, signal=152%
Immunogenic	GSE41978_KLRG1_HIGH_VS_LOW_EFFECTOR_CD8_TCELL_UP	19	0.48	2.34	0.000	0.033	0.711	878	tags=74%, list=31%, signal=107%
GO	GO_COFACTOR_BINDING	26	0.42	2.33	0.000	0.008	0.331	1534	tags=96%, list=55%, signal=211%
GO	GO_REGULATION_OF_LIPID_METABOLIC_PROCESS	31	0.38	2.33	0.000	0.009	0.341	842	tags=65%, list=30%, signal=91%
Oncogenic	MTOR_UP.V1_DN	26	0.41	2.32	0.000	0.022	0.033	755	tags=62%, list=27%, signal=83%
CancerModules	MODULE_63	39	0.36	2.32	0.002	0.002	0.037	1103	tags=72%, list=39%, signal=117%
Immunogenic	GSE2585_CTEC_VS_MTEC_THYMUS_UP	19	0.48	2.32	0.000	0.036	0.751	993	tags=79%, list=35%, signal=121%
GO	GO_ARTERY_DEVELOPMENT	20	0.47	2.32	0.004	0.009	0.353	1482	tags=100%, list=53%, signal=211%
GO	GO_CARBOHYDRATE_DERIVATIVE_METABOLIC_PROCESS	100	0.24	2.32	0.000	0.009	0.364	991	tags=54%, list=35%, signal=81%
Oncogenic	CYCLIN_D1_UP.V1_UP	20	0.46	2.32	0.000	0.016	0.036	836	tags=70%, list=30%, signal=99%
CancerModules	MODULE_259	13	0.56	2.32	0.000	0.002	0.040	1029	tags=92%, list=37%, signal=145%
Immunogenic	GSE22935_WT_VS_MYD88_KO_MACROPHAGE_UP	31	0.38	2.31	0.002	0.038	0.776	1332	tags=84%, list=48%, signal=158%
Immunogenic	GSE25088_CTRL_VS_IL4_STIM_MACROPHAGE_DN	29	0.39	2.31	0.000	0.039	0.791	1476	tags=93%, list=53%, signal=195%
GO	GO_NEGATIVE_REGULATION_OF_MULTICELLULAR_ORGANISMA	133	0.22	2.30	0.000	0.010	0.399	866	tags=47%, list=31%, signal=65%
GO	GO_GOLGI_MEMBRANE	82	0.26	2.30	0.000	0.010	0.399	765	tags=48%, list=27%, signal=64%
GO	GO_REGULATION_OF_WOUND_HEALING	16	0.52	2.30	0.000	0.010	0.403	1359	tags=100%, list=49%, signal=193%
GO	GO_RESPONSE_TO_BMP	17	0.50	2.30	0.000	0.010	0.411	699	tags=71%, list=25%, signal=93%
Immunogenic	GSE9960_HEALTHY_VS_GRAM_NEG_SEPSIS_PBMC_UP	19	0.47	2.30	0.000	0.041	0.819	1114	tags=84%, list=40%, signal=139%
CancerModules	MODULE_16	50	0.32	2.30	0.000	0.002	0.045	1405	tags=80%, list=50%, signal=158%
GO	GO_RECEPTOR_SIGNALING_PROTEIN_ACTIVITY	26	0.41	2.30	0.000	0.010	0.422	619	tags=58%, list=22%, signal=73%
Oncogenic	CAHOY_ASTROCYTIC	22	0.44	2.29	0.002	0.014	0.042	898	tags=73%, list=32%, signal=106%
GO	GO_REGULATION_OF_B_CELL_ACTIVATION	18	0.49	2.29	0.000	0.010	0.433	1114	tags=89%, list=40%, signal=147%
GO	GO_PLATELET_DEGRANULATION	17	0.49	2.29	0.002	0.010	0.433	1160	tags=88%, list=41%, signal=150%
GO	GO_TUBE_DEVELOPMENT	84	0.25	2.29	0.000	0.010	0.434	1482	tags=77%, list=53%, signal=159%
CancerModules	MODULE_220	54	0.30	2.29	0.000	0.002	0.047	728	tags=50%, list=26%, signal=66%
Immunogenic	GSE15930_NAIVE_VS_48H_IN_VITRO_STIM_IFNAB_CD8_TCELL_	24	0.44	2.29	0.002	0.044	0.841	869	tags=71%, list=31%, signal=102%
Canonical	REACTOME_MUSCLE_CONTRACTION	11	0.60	2.28	0.000	0.023	0.053	81	tags=55%, list=3%, signal=56%
GO	GO_MONOCARBOXYLIC_ACID_METABOLIC_PROCESS	50	0.31	2.28	0.000	0.011	0.453	1499	tags=84%, list=54%, signal=177%
Immunogenic	GSE6259_CD4_TCELL_VS_CD8_TCELL_UP	33	0.37	2.28	0.000	0.045	0.858	605	tags=52%, list=22%, signal=65%
Immunogenic	GSE369_PRE_VS_POST_IL6_INJECTION_SOCS3_KO_LIVER_DN	32	0.38	2.28	0.002	0.045	0.860	1362	tags=84%, list=49%, signal=162%
GO	GO_GOLGI_APPARATUS_PART	107	0.23	2.27	0.000	0.011	0.476	765	tags=45%, list=27%, signal=59%
Immunogenic	GSE2405_0H_VS_12H_A_PHAGOCYTOPHILUM_STIM_NEUTROPI	28	0.39	2.27	0.002	0.047	0.876	1287	tags=82%, list=46%, signal=150%
Immunogenic	GSE18893_TCONV_VS_TREG_24H_CULTURE_UP	32	0.37	2.27	0.000	0.047	0.877	628	tags=53%, list=22%, signal=68%
Immunogenic	GSE12845_IGD_NEG_BLOOD_VS_DARKZONE_GC_TONSIL_BCELI	30	0.38	2.27	0.000	0.047	0.884	1151	tags=77%, list=41%, signal=129%
Hallmark	HALLMARK_IL6_JAK_STAT3_SIGNALING	13	0.54	2.27	0.000	0.007	0.014	1117	tags=92%, list=40%, signal=153%
Immunogenic	GSE34156_NOD2_LIGAND_VS_TLR1_TLR2_LIGAND_6H_TREATEI	32	0.37	2.26	0.000	0.047	0.887	1279	tags=81%, list=46%, signal=148%
GO	GO_HEART_DEVELOPMENT	70	0.27	2.26	0.000	0.012	0.506	813	tags=50%, list=29%, signal=69%
GO	GO_REGULATION_OF_ENDOTHELIAL_CELL_PROLIFERATION	12	0.57	2.26	0.000	0.012	0.509	421	tags=67%, list=15%, signal=78%
GO	GO_TRANSFORMING_GROWTH_FACTOR_BETA_RECEPTOR_SIGI	11	0.59	2.26	0.002	0.012	0.514	753	tags=82%, list=27%, signal=111%
Canonical	KEGG_JAK_STAT_SIGNALING_PATHWAY	15	0.51	2.26	0.000	0.021	0.065	1008	tags=87%, list=36%, signal=135%
Immunogenic	GSE37301_MULTIPOTENT_PROGENITOR_VS_GRAN_MONO_PR(	24	0.42	2.26	0.000	0.049	0.899	643	tags=58%, list=23%, signal=75%
Immunogenic	GSE21927_SPLEEN_VS_4T1_TUMOR_MONOCYTE_BALBC_UP	19	0.47	2.25	0.000	0.048	0.901	362	tags=53%, list=13%, signal=60%

Immunogenic	GSE45365_NK_CELL_VS_CD11B_DC_DN	19	0.46	2.25	0.000	0.048	0.901	1387	tags=95%, list=50%, signal=186%
GO	GO_MUSCLE_STRUCTURE_DEVELOPMENT	53	0.30	2.24	0.000	0.014	0.574	747	tags=51%, list=27%, signal=68%
GO	GO_ENDOTHELIUM_DEVELOPMENT	20	0.46	2.24	0.000	0.014	0.576	1302	tags=90%, list=46%, signal=167%
Oncogenic	KRAS.300_UP.V1_DN	21	0.44	2.24	0.002	0.018	0.064	360	tags=48%, list=13%, signal=54%
GO	GO_DEFENSE_RESPONSE_TO_BACTERIUM	20	0.45	2.23	0.002	0.014	0.585	1189	tags=85%, list=42%, signal=147%
GO	GO_REGULATION_OF_SYMBIOSIS_ENCOMPASSING_MUTUALISM	19	0.46	2.23	0.002	0.014	0.587	1386	tags=95%, list=49%, signal=186%
GO	GO_RESPONSE_TO_BIOTIC_STIMULUS	75	0.26	2.22	0.000	0.015	0.620	1205	tags=65%, list=43%, signal=112%
GO	GO_CYTOKINE_RECEPTOR_ACTIVITY	14	0.52	2.22	0.002	0.015	0.624	1131	tags=93%, list=40%, signal=155%
GO	GO_LYMPHOCYTE_MEDIATED_IMMUNITY	14	0.53	2.22	0.002	0.015	0.632	1171	tags=93%, list=42%, signal=159%
Canonical	PID_PDGFRB_PATHWAY	16	0.49	2.22	0.002	0.021	0.081	746	tags=69%, list=27%, signal=93%
Oncogenic	AKT_UP.V1_DN	29	0.38	2.21	0.000	0.019	0.082	877	tags=66%, list=31%, signal=94%
GO	GO_CYTOKINE_BINDING	12	0.54	2.21	0.000	0.016	0.658	699	tags=75%, list=25%, signal=100%
GO	GO_HUMORAL_IMMUNE_RESPONSE	17	0.48	2.21	0.004	0.016	0.661	880	tags=76%, list=31%, signal=111%
GO	GO_ENZYME_LINKED_RECEPTOR_PROTEIN_SIGNALING_PATHW	90	0.24	2.21	0.000	0.016	0.663	753	tags=46%, list=27%, signal=60%
GO	GO_REGENERATION	22	0.42	2.20	0.000	0.017	0.692	924	tags=73%, list=33%, signal=108%
GO	GO_LEUKOCYTE_MEDIATED_IMMUNITY	17	0.48	2.19	0.002	0.017	0.705	1171	tags=88%, list=42%, signal=151%
GO	GO_SINGLE_ORGANISM_CATABOLIC_PROCESS	97	0.23	2.19	0.002	0.018	0.713	1595	tags=79%, list=57%, signal=178%
GO	GO_CELL_PROLIFERATION	86	0.24	2.19	0.002	0.017	0.713	1180	tags=63%, list=42%, signal=105%
GO	GO_ENDOSOME	86	0.24	2.18	0.002	0.018	0.720	1190	tags=63%, list=42%, signal=106%
GO	GO_SMALL_MOLECULE_BIOSYNTHETIC_PROCESS	53	0.29	2.18	0.000	0.018	0.728	1404	tags=77%, list=50%, signal=152%
GO	GO_ORGANONITROGEN_COMPOUND_CATABOLIC_PROCESS	39	0.32	2.17	0.002	0.019	0.764	1240	tags=74%, list=44%, signal=132%
GO	GO_INTRINSIC_COMPONENT_OF_PLASMA_MEMBRANE	221	0.17	2.17	0.000	0.019	0.766	888	tags=44%, list=32%, signal=59%
GO	GO_MOVEMENT_OF_CELL_OR_SUBCELLULAR_COMPONENT	156	0.19	2.16	0.000	0.019	0.775	1194	tags=58%, list=43%, signal=96%
GO	GO_INFLAMMATORY_RESPONSE	37	0.33	2.16	0.000	0.019	0.776	1425	tags=81%, list=51%, signal=163%
GO	GO_REGULATION_OF_MAP_KINASE_ACTIVITY	35	0.34	2.16	0.002	0.019	0.779	1098	tags=71%, list=39%, signal=116%
GO	GO_VESICLE_MEDIATED_TRANSPORT	156	0.19	2.16	0.000	0.020	0.791	1219	tags=59%, list=44%, signal=99%
Hallmark	HALLMARK_ADIPOGENESIS	23	0.40	2.16	0.004	0.013	0.034	1020	tags=74%, list=36%, signal=115%
GO	GO_MYOSIN_COMPLEX	11	0.56	2.15	0.000	0.020	0.794	578	tags=73%, list=21%, signal=91%
GO	GO_RESPONSE_TO_GROWTH_FACTOR	66	0.26	2.15	0.000	0.020	0.796	765	tags=48%, list=27%, signal=65%
GO	GO_MOVEMENT_IN_ENVIRONMENT_OF_OTHER_ORGANISM_IN	12	0.54	2.15	0.002	0.020	0.808	529	tags=67%, list=19%, signal=82%
GO	GO_REGULATION_OF_LEUKOCYTE_DIFFERENTIATION	21	0.43	2.15	0.004	0.020	0.813	1279	tags=86%, list=46%, signal=157%
GO	GO_PHAGOCYTOSIS	29	0.37	2.14	0.002	0.021	0.826	1169	tags=76%, list=42%, signal=129%
Oncogenic	MTOR_UP.V1_UP	10	0.60	2.14	0.000	0.028	0.138	1139	tags=100%, list=41%, signal=168%
GO	GO_CYTOKINE_ACTIVITY	11	0.56	2.14	0.000	0.021	0.827	817	tags=82%, list=29%, signal=115%
GO	GO_LIPID_METABOLIC_PROCESS	143	0.19	2.13	0.000	0.022	0.842	993	tags=49%, list=35%, signal=72%
Hallmark	HALLMARK_XENOBIOTIC_METABOLISM	15	0.49	2.13	0.000	0.012	0.040	1431	tags=100%, list=51%, signal=203%
GO	GO_ORGANIC_HYDROXY_COMPOUND_METABOLIC_PROCESS	53	0.28	2.13	0.000	0.022	0.851	1128	tags=64%, list=40%, signal=105%
GO	GO_ORGANIC_ACID_CATABOLIC_PROCESS	22	0.41	2.12	0.000	0.024	0.868	1016	tags=73%, list=36%, signal=113%
GO	GO_MEMBRANE_LIPID_METABOLIC_PROCESS	21	0.41	2.12	0.000	0.023	0.868	406	tags=48%, list=14%, signal=55%
GO	GO_GROWTH_FACTOR_BINDING	19	0.44	2.11	0.002	0.024	0.869	705	tags=63%, list=25%, signal=84%
Canonical	REACTOME_PLATELET_ACTIVATION_SIGNALING_AND_AGGREGA	28	0.36	2.11	0.005	0.042	0.177	1160	tags=75%, list=41%, signal=127%
Oncogenic	LEF1_UP.V1_UP	30	0.35	2.11	0.002	0.032	0.172	1635	tags=93%, list=58%, signal=222%
GO	GO_CELL_SUBSTRATE_ADHESION	28	0.36	2.11	0.003	0.024	0.883	1171	tags=75%, list=42%, signal=128%
GO	GO_OXIDOREDUCTASE_ACTIVITY	59	0.27	2.10	0.002	0.025	0.886	993	tags=58%, list=35%, signal=87%
Canonical	KEGG_TGF_BETA_SIGNALING_PATHWAY	10	0.58	2.10	0.000	0.039	0.189	733	tags=80%, list=26%, signal=108%
Oncogenic	ESC_J1_UP_EARLY.V1_UP	20	0.42	2.10	0.004	0.031	0.191	1108	tags=80%, list=40%, signal=131%
GO	GO_PROTEIN_O_LINKED_GLYCOSYLATION	10	0.57	2.09	0.004	0.026	0.896	991	tags=90%, list=35%, signal=139%
GO	GO_REGULATION_OF_TISSUE_REMODELING	10	0.57	2.09	0.002	0.026	0.896	990	tags=90%, list=35%, signal=139%
GO	GO_ADAPTIVE_IMMUNE_RESPONSE	27	0.36	2.09	0.005	0.027	0.908	1387	tags=85%, list=50%, signal=167%
GO	GO_REGULATION_OF_OSTEOBLAST_DIFFERENTIATION	19	0.43	2.09	0.000	0.026	0.908	1069	tags=79%, list=38%, signal=127%
GO	GO_RECEPTOR_COMPLEX	61	0.26	2.09	0.002	0.026	0.911	887	tags=54%, list=32%, signal=77%
GO	GO_ENDOPLASMIC_RETICULUM	181	0.18	2.08	0.002	0.027	0.915	1219	tags=57%, list=44%, signal=94%
GO	GO_CONTRACTILE_FIBER	30	0.35	2.08	0.003	0.028	0.925	415	tags=43%, list=15%, signal=50%

GO	GO_CELL_MATRIX_ADHESION	18	0.43	2.08	0.007	0.028	0.926	1171	tags=83%, list=42%, signal=142%
Oncogenic	BMI1_DN.V1_UP	23	0.38	2.07	0.004	0.032	0.211	1214	tags=78%, list=43%, signal=137%
GO	GO_RESPONSE_TO_ESTRADIOL	19	0.42	2.07	0.004	0.028	0.934	1040	tags=79%, list=37%, signal=125%
GO	GO_WATER_SOLUBLE_VITAMIN_METABOLIC_PROCESS	12	0.52	2.07	0.004	0.029	0.936	1139	tags=92%, list=41%, signal=154%
GO	GO_CELLULAR_LIPID_METABOLIC_PROCESS	117	0.20	2.07	0.002	0.029	0.938	625	tags=36%, list=22%, signal=44%
GO	GO_DEVELOPMENTAL_MATURATION	30	0.34	2.06	0.002	0.029	0.939	409	tags=43%, list=15%, signal=50%
Hallmark	HALLMARK_APICAL_JUNCTION	32	0.34	2.06	0.005	0.018	0.068	1015	tags=66%, list=36%, signal=102%
GO	GO_ACTIN_FILAMENT_BUNDLE	12	0.52	2.06	0.004	0.029	0.942	952	tags=83%, list=34%, signal=126%
GO	GO_COMPLEMENT_ACTIVATION	11	0.54	2.06	0.002	0.029	0.942	880	tags=82%, list=31%, signal=119%
Oncogenic	KRAS.600_UP.V1_DN	32	0.33	2.06	0.003	0.032	0.235	974	tags=63%, list=35%, signal=95%
GO	GO_RESPONSE_TO_TRANSITION_METAL_NANOPARTICLE	14	0.49	2.06	0.002	0.029	0.946	1275	tags=93%, list=46%, signal=170%
GO	GO_ANATOMICAL_STRUCTURE_FORMATION_INVOLVED_IN_MC	125	0.20	2.06	0.003	0.029	0.946	1146	tags=57%, list=41%, signal=92%
GO	GO_PEPTIDASE_REGULATOR_ACTIVITY	25	0.37	2.06	0.002	0.029	0.949	1080	tags=72%, list=39%, signal=116%
Canonical	REACTOME_HEMOSTASIS	60	0.26	2.06	0.002	0.045	0.242	1173	tags=65%, list=42%, signal=109%
GO	GO_PROTEASE_BINDING	10	0.56	2.05	0.006	0.030	0.957	547	tags=70%, list=20%, signal=87%
GO	GO_EPITHELIUM_DEVELOPMENT	130	0.19	2.05	0.002	0.030	0.957	1439	tags=69%, list=51%, signal=136%
GO	GO_NEGATIVE_REGULATION_OF_CYTOKINE_PRODUCTION	22	0.39	2.05	0.009	0.030	0.960	353	tags=45%, list=13%, signal=52%
Canonical	KEGG_FOCAL_ADHESION	28	0.36	2.05	0.005	0.041	0.253	1062	tags=71%, list=38%, signal=114%
GO	GO_POSITIVE_REGULATION_OF_EPITHELIAL_CELL_PROLIFERATI	19	0.43	2.05	0.002	0.030	0.960	594	tags=58%, list=21%, signal=73%
GO	GO_THIOESTER_METABOLIC_PROCESS	13	0.48	2.05	0.000	0.030	0.961	1284	tags=92%, list=46%, signal=170%
GO	GO_POSITIVE_REGULATION_OF_LIPID_METABOLIC_PROCESS	16	0.46	2.04	0.005	0.031	0.965	733	tags=69%, list=26%, signal=93%
GO	GO_RECEPTOR_BINDING	152	0.17	2.04	0.002	0.031	0.965	841	tags=43%, list=30%, signal=58%
GO	GO_EPITHELIAL_CELL_DIFFERENTIATION	67	0.25	2.04	0.005	0.031	0.965	829	tags=49%, list=30%, signal=68%
GO	GO_RESPONSE_TO_EXTRACELLULAR_STIMULUS	50	0.28	2.04	0.002	0.031	0.965	926	tags=58%, list=33%, signal=85%
GO	GO_REGULATION_OF_RESPONSE_TO_WOUNDING	43	0.29	2.04	0.002	0.031	0.965	1554	tags=84%, list=55%, signal=185%
GO	GO_ORGAN_MORPHOGENESIS	127	0.19	2.03	0.006	0.031	0.968	1150	tags=57%, list=41%, signal=92%
Canonical	KEGG_ECM_RECEPTOR_INTERACTION	14	0.47	2.03	0.000	0.042	0.277	973	tags=79%, list=35%, signal=120%
GO	GO_RESPONSE_TO_LIPID	94	0.22	2.02	0.003	0.034	0.980	1554	tags=77%, list=55%, signal=166%
GO	GO_NEGATIVE_REGULATION_OF_MULTI_ORGANISM_PROCESS	14	0.48	2.02	0.004	0.034	0.980	1461	tags=100%, list=52%, signal=208%
GO	GO_CELLULAR_AMINO_ACID_METABOLIC_PROCESS	38	0.31	2.02	0.007	0.035	0.980	1404	tags=79%, list=50%, signal=156%
GO	GO_CELL_CHEMOTAXIS	13	0.49	2.01	0.004	0.035	0.981	1256	tags=92%, list=45%, signal=167%
GO	GO_RESPONSE_TO_ESTROGEN	29	0.35	2.01	0.007	0.035	0.981	833	tags=62%, list=30%, signal=87%
GO	GO_MUSCLE_ORGAN_DEVELOPMENT	35	0.32	2.01	0.002	0.035	0.981	979	tags=63%, list=35%, signal=95%
GO	GO_RESPONSE_TO_BACTERIUM	44	0.29	2.01	0.007	0.035	0.983	1430	tags=80%, list=51%, signal=160%
GO	GO_CELLULAR_RESPONSE_TO_ORGANIC_SUBSTANCE	198	0.16	2.01	0.000	0.035	0.983	1057	tags=49%, list=38%, signal=74%
Hallmark	HALLMARK_INFLAMMATORY_RESPONSE	23	0.38	2.01	0.006	0.024	0.100	1584	tags=96%, list=57%, signal=218%
GO	GO_DEFENSE_RESPONSE_TO_VIRUS	10	0.54	2.01	0.004	0.035	0.987	1279	tags=100%, list=46%, signal=183%
Canonical	KEGG_HEMATOPOIETIC_CELL_LINEAGE	12	0.51	2.01	0.009	0.045	0.326	1189	tags=92%, list=42%, signal=159%
GO	GO_MEMBRANE_MICRODOMAIN	54	0.26	2.00	0.000	0.036	0.989	1212	tags=67%, list=43%, signal=115%
GO	GO_GLOMERULUS_DEVELOPMENT	11	0.52	2.00	0.004	0.036	0.989	472	tags=64%, list=17%, signal=76%
GO	GO_ACTIN_CYTOSKELETON	58	0.25	2.00	0.007	0.036	0.989	1430	tags=74%, list=51%, signal=148%
GO	GO_VESICLE_LUMEN	11	0.51	2.00	0.000	0.037	0.992	733	tags=73%, list=26%, signal=98%
GO	GO_SENSORY_ORGAN_DEVELOPMENT	73	0.23	2.00	0.005	0.037	0.994	796	tags=47%, list=28%, signal=63%
Hallmark	HALLMARK_COAGULATION	22	0.38	1.99	0.007	0.022	0.109	1170	tags=77%, list=42%, signal=132%
GO	GO_RESPONSE_TO_OXYGEN_LEVELS	35	0.31	1.99	0.009	0.038	0.995	926	tags=60%, list=33%, signal=89%
GO	GO_ARTERY_MORPHOGENESIS	10	0.53	1.99	0.004	0.038	0.995	1307	tags=100%, list=47%, signal=187%
Oncogenic	ESC_J1_UP_LATE.V1_UP	27	0.35	1.99	0.004	0.049	0.370	1089	tags=70%, list=39%, signal=114%
GO	GO_REGULATION_OF_CELL_PROLIFERATION	160	0.18	1.99	0.006	0.038	0.996	1554	tags=72%, list=55%, signal=152%
GO	GO_REGULATION_OF_TRANSMEMBRANE_RECEPTOR_PROTEIN_	29	0.34	1.99	0.012	0.038	0.996	771	tags=59%, list=28%, signal=80%
Canonical	REACTOME_SEMA4D_IN_SEMAPHORIN_SIGNALING	10	0.55	1.98	0.006	0.046	0.356	594	tags=70%, list=21%, signal=89%
GO	GO_POSITIVE_REGULATION_OF_B_CELL_ACTIVATION	14	0.45	1.98	0.007	0.040	0.997	1114	tags=86%, list=40%, signal=142%
GO	GO_REGULATION_OF_BIOMINERAL_TISSUE_DEVELOPMENT	12	0.51	1.97	0.000	0.042	0.997	733	tags=75%, list=26%, signal=101%
GO	GO_POSITIVE_REGULATION_OF_RESPONSE_TO_WOUNDING	12	0.49	1.97	0.012	0.042	0.997	622	tags=67%, list=22%, signal=85%

GO	GO_REGULATION_OF_CELL_ADHESION	74	0.22	1.96	0.008	0.043	0.997	1214	tags=64%, list=43%, signal=109%
GO	GO_STEROL_METABOLIC_PROCESS	18	0.41	1.96	0.000	0.044	0.998	1364	tags=89%, list=49%, signal=172%
GO	GO_SERINE_HYDROLASE_ACTIVITY	23	0.37	1.96	0.008	0.044	0.998	1057	tags=74%, list=38%, signal=118%
GO	GO_TISSUE_MORPHOGENESIS	80	0.22	1.95	0.005	0.045	0.999	1435	tags=71%, list=51%, signal=142%
GO	GO_UROGENITAL_SYSTEM_DEVELOPMENT	49	0.27	1.95	0.005	0.045	0.999	1554	tags=82%, list=55%, signal=180%
GO	GO_RESPONSE_TO_ENDOGENOUS_STIMULUS	181	0.16	1.95	0.003	0.044	0.999	883	tags=43%, list=32%, signal=59%
GO	GO_NEGATIVE_REGULATION_OF_VASCULATURE_DEVELOPMEN	13	0.48	1.95	0.002	0.046	0.999	979	tags=77%, list=35%, signal=118%
GO	GO_RECEPTOR_SIGNALING_PROTEIN_SERINE_THREONINE_KINA	10	0.54	1.94	0.011	0.046	0.999	592	tags=70%, list=21%, signal=88%
GO	GO_REGULATION_OF_PROTEIN_SERINE_THREONINE_KINASE_A	48	0.27	1.94	0.007	0.046	0.999	887	tags=54%, list=32%, signal=78%
GO	GO_BIOLOGICAL_ADHESION	150	0.18	1.94	0.002	0.046	0.999	1194	tags=57%, list=43%, signal=95%
GO	GO_RESPONSE_TO_NUTRIENT	24	0.35	1.94	0.005	0.045	0.999	926	tags=67%, list=33%, signal=99%
GO	GO_ALCOHOL_METABOLIC_PROCESS	39	0.29	1.94	0.005	0.045	0.999	1364	tags=77%, list=49%, signal=148%
GO	GO_ORGANIC_HYDROXY_COMPOUND_BIOSYNTHETIC_PROCESS	18	0.40	1.94	0.007	0.046	0.999	1109	tags=78%, list=40%, signal=128%
GO	GO_ENDOTHELIAL_CELL_DIFFERENTIATION	15	0.44	1.94	0.011	0.046	0.999	1420	tags=93%, list=51%, signal=188%
GO	GO_TRANSMEMBRANE_RECEPTOR_PROTEIN_TYROSINE_KINASE	12	0.49	1.93	0.007	0.047	1.000	594	tags=67%, list=21%, signal=84%
GO	GO_GASTRULATION	21	0.38	1.93	0.005	0.048	1.000	1505	tags=90%, list=54%, signal=194%
GO	GO_RESPONSE_TO_TRANSFORMING_GROWTH_FACTOR_BETA	19	0.40	1.93	0.013	0.048	1.000	1007	tags=74%, list=36%, signal=114%
GO	GO_CORONARY_VASCULATURE_DEVELOPMENT	11	0.50	1.93	0.004	0.048	1.000	1410	tags=100%, list=50%, signal=201%
Hallmark	HALLMARK_ALLOGRAFT_REJECTION	22	0.37	1.88	0.012	0.041	0.205	1382	tags=86%, list=49%, signal=169%
Hallmark	HALLMARK_HYPOXIA	26	0.33	1.84	0.014	0.044	0.237	1308	tags=77%, list=47%, signal=143%

Collection	Downregulated Sets (sorted by negative NES)	SIZE	ES	NES	NOM p-val	FDR q-val	FWER p-val	RANK AT MAX	LEADING EDGE
ChemGenPerturb	BLALOCK_ALZHEIMERS_DISEASE_DN	158	-0.46	-5.89	0.000	0.000	0.000	1205	tags=87%, list=43%, signal=144%
ChemGenPerturb	KIM_ALL_DISORDERS_CALB1_CORR_UP	73	-0.54	-5.00	0.000	0.000	0.000	1248	tags=97%, list=45%, signal=171%
ChemGenPerturb	MILI_PSEUDOPODIA_HAPTOTAXIS_UP	48	-0.43	-3.30	0.000	0.000	0.000	1163	tags=83%, list=42%, signal=140%
ChemGenPerturb	SHEN_SMARCA2_TARGETS_UP	28	-0.53	-3.28	0.000	0.000	0.000	1243	tags=96%, list=44%, signal=172%
GO	GO_SYNAPSE_PART	101	-0.29	-3.11	0.000	0.000	0.000	1246	tags=73%, list=44%, signal=127%
ChemGenPerturb	SCHLOSSER_MYC_TARGETS_REPRESSED_BY_SERUM	19	-0.60	-3.11	0.000	0.000	0.002	1120	tags=100%, list=40%, signal=165%
ChemGenPerturb	HAMAI_APOPTOSIS_VIA_TRAIL_UP	52	-0.38	-3.10	0.000	0.000	0.002	1285	tags=83%, list=46%, signal=150%
Oncogenic	KRAS.KIDNEY_UP.V1_UP	27	-0.50	-3.06	0.000	0.000	0.000	1199	tags=93%, list=43%, signal=160%
ChemGenPerturb	LU_AGING_BRAIN_DN	25	-0.53	-3.04	0.000	0.000	0.002	1231	tags=96%, list=44%, signal=170%
GO	GO_SYNAPSE	117	-0.27	-3.04	0.000	0.000	0.000	1246	tags=71%, list=44%, signal=122%
Oncogenic	CAHOY_NEURONAL	18	-0.60	-3.02	0.000	0.000	0.000	995	tags=94%, list=36%, signal=146%
ChemGenPerturb	SENGUPTA_NASOPHARYNGEAL_CARCINOMA_WITH_LMP1_UP	37	-0.43	-2.99	0.000	0.000	0.002	1160	tags=84%, list=41%, signal=141%
ChemGenPerturb	VERHAAK_GLIOBLASTOMA_PRONEURAL	29	-0.46	-2.79	0.000	0.001	0.009	1170	tags=86%, list=42%, signal=147%
ChemGenPerturb	STARK_PREFRONTAL_CORTEX_22Q11_DELETION_DN	48	-0.36	-2.78	0.000	0.001	0.009	1175	tags=77%, list=42%, signal=131%
GO	GO_SYNAPTIC_SIGNALING	64	-0.32	-2.78	0.000	0.001	0.006	1246	tags=77%, list=44%, signal=135%
ChemGenPerturb	MIKKELSEN_MEF_ICP_WITH_H3K4ME3_AND_H3K27ME3	10	-0.74	-2.78	0.000	0.001	0.010	737	tags=100%, list=26%, signal=135%
Oncogenic	PRC2_EED_UP.V1_UP	21	-0.53	-2.76	0.000	0.000	0.002	494	tags=67%, list=18%, signal=80%
Immunogenic	GSE34156_NOD2_LIGAND_VS_TLR1_TLR2_LIGAND_24H_TREAT	13	-0.63	-2.75	0.000	0.025	0.037	878	tags=92%, list=31%, signal=134%
GO	GO_CATION_CHANNEL_COMPLEX	29	-0.45	-2.75	0.000	0.001	0.008	1226	tags=90%, list=44%, signal=158%
GO	GO_MICROTUBULE	31	-0.41	-2.69	0.000	0.002	0.014	512	tags=55%, list=18%, signal=66%
GO	GO_MRNA_METABOLIC_PROCESS	36	-0.40	-2.67	0.000	0.002	0.019	1157	tags=81%, list=41%, signal=135%
ChemGenPerturb	WEI_MYCN_TARGETS_WITH_E_BOX	65	-0.30	-2.65	0.000	0.001	0.021	1163	tags=71%, list=42%, signal=118%
GO	GO_BEHAVIOR	70	-0.29	-2.65	0.000	0.002	0.022	1248	tags=73%, list=45%, signal=128%
GO	GO_RNA_PROCESSING	48	-0.34	-2.62	0.000	0.003	0.032	1180	tags=75%, list=42%, signal=127%
ChemGenPerturb	GEORGES_TARGETS_OF_MIR192_AND_MIR215	87	-0.25	-2.59	0.000	0.002	0.037	1438	tags=77%, list=51%, signal=153%
Oncogenic	KRAS.300_UP.V1_UP	20	-0.48	-2.59	0.000	0.001	0.009	1060	tags=85%, list=38%, signal=136%
ChemGenPerturb	JOHNSTONE_PARVB_TARGETS_3_DN	74	-0.28	-2.58	0.000	0.002	0.040	894	tags=57%, list=32%, signal=81%
GO	GO_SYNAPTIC_MEMBRANE	48	-0.31	-2.45	0.000	0.012	0.144	1285	tags=79%, list=46%, signal=144%
GO	GO_POSTSYNAPSE	69	-0.27	-2.44	0.000	0.011	0.152	1246	tags=72%, list=44%, signal=127%
GO	GO_RIBONUCLEOPROTEIN_COMPLEX_BIOGENESIS	24	-0.43	-2.35	0.000	0.021	0.291	1157	tags=83%, list=41%, signal=141%
GO	GO_PEPTIDYL_LYSINE_MODIFICATION	21	-0.43	-2.29	0.000	0.032	0.417	1420	tags=95%, list=51%, signal=192%

GO	GO_MICROTUBULE_BINDING	18	-0.46	-2.28	0.000	0.031	0.434	936	tags=78%, list=33%, signal=116%
GO	GO_POTASSIUM_CHANNEL_COMPLEX	19	-0.43	-2.25	0.002	0.036	0.527	1226	tags=89%, list=44%, signal=158%
GO	GO_POSTSYNAPTIC_MEMBRANE	41	-0.32	-2.25	0.005	0.034	0.527	1285	tags=80%, list=46%, signal=147%
GO	GO_RNA_BINDING	130	-0.19	-2.23	0.000	0.036	0.574	1216	tags=62%, list=43%, signal=105%
GO	GO_RIBONUCLEOPROTEIN_COMPLEX	41	-0.31	-2.20	0.000	0.039	0.626	1211	tags=73%, list=43%, signal=127%
GO	GO_PRESYNAPSE	43	-0.30	-2.19	0.000	0.040	0.650	1231	tags=74%, list=44%, signal=131%
GO	GO_EXOCYTIC_VESICLE_MEMBRANE	13	-0.51	-2.18	0.000	0.042	0.686	1187	tags=92%, list=42%, signal=159%
GO	GO_NCRNA_METABOLIC_PROCESS	27	-0.37	-2.16	0.004	0.045	0.730	1089	tags=74%, list=39%, signal=120%
GO	GO_PROTEIN_ACYLATION	12	-0.53	-2.16	0.007	0.044	0.733	690	tags=75%, list=25%, signal=99%
GO	GO_LEARNING	19	-0.42	-2.16	0.007	0.042	0.734	1060	tags=79%, list=38%, signal=126%
GO	GO_COVALENT_CHROMATIN_MODIFICATION	29	-0.35	-2.16	0.000	0.040	0.743	972	tags=69%, list=35%, signal=105%
GO	GO_TRANSPORTER_COMPLEX	53	-0.26	-2.14	0.005	0.043	0.782	1246	tags=72%, list=44%, signal=127%

SIZE	Number of genes in the gene set after filtering out those genes not in the expression dataset
ES	Enrichment Score
NES	Normalized Enrichment Score
NOM p-val	Nominal P value
FDR q-val	False Discovery Rate q value
FWER p-val	Familywise-Error Rate q value
RANK AT MAX	Rank at maximum enrichment score
