# Supplementary material for: Loneliness 5 years ante-mortem is associated with disease-related differential gene expression in postmortem dorsolateral prefrontal cortex
Source: Transl Psychiatry. 2018 Jan 10;8:2. doi: 10.1038/s41398-017-0086-2 (PMC5802527; doi:10.1038/s41398-017-0086-2)
Supplement: Supplementary file 3 — Supplemental Table 3 [file 41398_2017_86_MOESM3_ESM.pdf]

PROBE	RANK IN GENE LIST	RANK METRIC SCORE	RUNNING ES	CORE ENRICHMENT
TAGLN	11	3.588	0.003	Yes
CDC42EP4	14	3.567	0.010	Yes
NAGA	26	3.418	0.013	Yes
IL13RA1	28	3.417	0.019	Yes
TAX1BP3	29	3.411	0.026	Yes
ST6GALNA(	31	3.402	0.033	Yes
EWSR1	38	3.360	0.038	Yes
SEC14L2	40	3.345	0.044	Yes
DAG1	70	3.198	0.040	Yes
MYO1C	80	3.163	0.043	Yes
BMP2K	83	3.152	0.049	Yes
LTBP2	93	3.127	0.052	Yes
MMP2	94	3.118	0.058	Yes
MR1	100	3.106	0.063	Yes
PLCG2	149	3.030	0.051	Yes
OGG1	157	3.013	0.054	Yes
BCAM	158	3.013	0.060	Yes
ECE1	164	3.000	0.065	Yes
CTNS	178	2.984	0.066	Yes
FBLN1	181	2.973	0.071	Yes
ERLIN2	184	2.967	0.077	Yes
COTL1	188	2.950	0.082	Yes
TCN2	191	2.941	0.087	Yes
IDH2	197	2.938	0.091	Yes
WFS1	198	2.936	0.097	Yes
SLC7A5	200	2.930	0.103	Yes
LMNA	201	2.930	0.109	Yes
PLEC	208	2.920	0.113	Yes
PTTG1IP	217	2.902	0.116	Yes
GALNT10	218	2.895	0.122	Yes
HFE	223	2.887	0.126	Yes
RIN3	236	2.855	0.127	Yes
ANXA2	238	2.848	0.133	Yes
EFEMP2	242	2.843	0.138	Yes
SCAMP2	244	2.841	0.143	Yes
PDGFB	246	2.837	0.149	Yes
SREBF1	258	2.826	0.150	Yes
FGFR3	263	2.817	0.155	Yes
CXCR7	264	2.810	0.160	Yes
IFNA16	271	2.795	0.164	Yes
PDGFRB	294	2.785	0.161	Yes
FADS1	313	2.765	0.160	Yes
ACAA1	322	2.743	0.163	Yes
MASP1	331	2.736	0.165	Yes
GJA1	342	2.723	0.167	Yes

RREB1	357	2.695	0.167	Yes
DMC1	366	2.685	0.170	Yes
CTDSP1	367	2.684	0.175	Yes
CASKIN2	369	2.683	0.181	Yes
DSC3	372	2.681	0.185	Yes
TRIOBP	377	2.670	0.189	Yes
MYO15B	394	2.643	0.189	Yes
LRP10	401	2.636	0.192	Yes
PTRF	416	2.617	0.192	Yes
TAF15	417	2.616	0.197	Yes
PLXNB2	420	2.608	0.202	Yes
GPSM3	433	2.593	0.203	Yes
ITGB5	484	2.546	0.189	Yes
ARFGAP2	502	2.527	0.187	Yes
USP4	507	2.525	0.191	Yes
LRP4	515	2.513	0.194	Yes
SOX13	531	2.502	0.193	Yes
INPP5K	545	2.490	0.193	Yes
TRPV1	548	2.489	0.198	Yes
COL18A1	561	2.481	0.198	Yes
ID3	563	2.480	0.203	Yes
DPYSL3	566	2.480	0.207	Yes
GPR107	571	2.479	0.211	Yes
TMEM8A	572	2.479	0.216	Yes
CD37	575	2.477	0.220	Yes
CD58	576	2.476	0.226	Yes
AP1B1	580	2.473	0.230	Yes
GPC4	581	2.473	0.235	Yes
EGFR	583	2.472	0.239	Yes
DDR1	587	2.470	0.243	Yes
MAPK12	592	2.467	0.247	Yes
PAX6	609	2.455	0.246	Yes
IL17RA	622	2.441	0.246	Yes
ACADS	625	2.440	0.251	Yes
NDST1	626	2.439	0.256	Yes
ZFHX3	633	2.432	0.258	Yes
EPS8	643	2.427	0.260	Yes
ABCA1	663	2.423	0.258	Yes
SNTA1	672	2.414	0.260	Yes
DAZAP1	697	2.398	0.255	Yes
TGFBR3	699	2.397	0.260	Yes
CDK12	709	2.388	0.262	Yes
PTCH1	711	2.386	0.266	Yes
SH3BP2	713	2.386	0.271	Yes
COL6A3	728	2.373	0.270	Yes
GOLGA1	729	2.373	0.275	Yes
SLC19A1	731	2.372	0.280	Yes

TGFB1	733	2.371	0.284	Yes
SERPINI2	754	2.354	0.281	Yes
ITGA6	766	2.347	0.282	Yes
PC	815	2.317	0.268	Yes
NOTCH4	829	2.308	0.268	Yes
WAS	845	2.296	0.267	Yes
PDLIM4	846	2.295	0.272	Yes
IL1R1	878	2.278	0.265	Yes
ADCY2	883	2.275	0.268	Yes
HEPH	898	2.268	0.267	Yes
TTLL3	910	2.263	0.268	Yes
PHKA2	933	2.248	0.264	Yes
MYLK	941	2.242	0.266	Yes
BCAT2	951	2.232	0.267	Yes
PBXIP1	960	2.227	0.269	Yes
ITGA7	973	2.218	0.269	Yes
SERTAD3	975	2.217	0.273	Yes
RHOBTB3	981	2.213	0.275	Yes
MLC1	1024	2.188	0.264	Yes
FGFR1	1029	2.187	0.267	Yes
PCSK5	1046	2.178	0.265	Yes
CTSH	1057	2.173	0.266	Yes
ID4	1058	2.172	0.270	Yes
FLNA	1062	2.171	0.274	Yes
MT1H	1063	2.170	0.278	Yes
NT5C	1068	2.166	0.281	Yes
SLC12A9	1071	2.164	0.285	Yes
TAPBP	1072	2.164	0.289	Yes
ARHGEF10	1073	2.164	0.294	Yes
LGALS3BP	1078	2.163	0.297	Yes
TGIF2	1095	2.153	0.295	Yes
WWTR1	1096	2.153	0.300	Yes
KCNK5	1103	2.149	0.302	Yes
TRPS1	1107	2.147	0.305	Yes
MVK	1109	2.146	0.309	Yes
CDC14A	1113	2.146	0.312	Yes
INPPL1	1122	2.141	0.314	Yes
SLC1A7	1134	2.138	0.314	Yes
SLC2A1	1139	2.136	0.317	Yes
ZC3H4	1161	2.129	0.313	Yes
TREX1	1166	2.128	0.316	Yes
WBSCR16	1175	2.119	0.317	Yes
PLIN3	1186	2.115	0.318	Yes
GPR56	1194	2.112	0.320	Yes
PLSCR3	1205	2.107	0.320	Yes
REST	1234	2.090	0.314	Yes
BGN	1240	2.089	0.316	Yes

TNS3	1251	2.083	0.317	Yes
TCF7L1	1260	2.077	0.318	Yes
CD86	1279	2.074	0.315	Yes
TRIM38	1280	2.074	0.320	Yes
S100A4	1308	2.063	0.313	Yes
FAM107A	1316	2.058	0.315	Yes
PRKX	1320	2.058	0.318	Yes
IL18	1322	2.057	0.322	Yes
MGAT1	1332	2.054	0.323	Yes
STARD3	1339	2.050	0.325	Yes
HADHA	1342	2.050	0.328	Yes
HIP1R	1374	2.036	0.321	Yes
HDAC1	1386	2.032	0.321	Yes
PTP4A3	1395	2.029	0.322	Yes
SOX2	1401	2.027	0.324	Yes
TULP3	1403	2.025	0.328	Yes
GTPBP1	1411	2.022	0.329	Yes
ABL1	1430	2.015	0.327	Yes
CIB1	1455	2.004	0.321	Yes
STK10	1471	1.997	0.320	Yes
CYHR1	1480	1.994	0.321	Yes
MAPKAPK2	1499	1.988	0.318	Yes
EVI2B	1502	1.988	0.321	Yes
ZFAND3	1504	1.988	0.325	Yes
PPP1R13L	1508	1.986	0.328	Yes
PLD2	1509	1.985	0.332	Yes
CR1	1511	1.985	0.336	Yes
WASF2	1516	1.983	0.339	Yes
SSPN	1517	1.983	0.343	Yes
HMGCL	1523	1.982	0.345	Yes
NCOR2	1525	1.981	0.349	Yes
VEGFC	1532	1.980	0.350	Yes
OS9	1535	1.978	0.354	Yes
PPP1R9A	1577	-1.981	0.342	No
AKAP9	1611	-1.994	0.334	No
SFTPC	1664	-2.010	0.318	No
RARB	1728	-2.042	0.298	No
DRD2	1795	-2.076	0.277	No
ACRV1	1822	-2.086	0.271	No
ZNF84	1887	-2.104	0.251	No
RNGTT	1912	-2.119	0.246	No
NGDN	1932	-2.125	0.243	No
RIMS2	2016	-2.168	0.216	No
SPAG6	2058	-2.192	0.205	No
DUT	2066	-2.199	0.206	No
MMP16	2092	-2.221	0.201	No
ING4	2112	-2.233	0.199	No

IRS2	2136	-2.247	0.195	No
RLN1	2154	-2.257	0.193	No
FOXO3	2189	-2.280	0.184	No
KIF3B	2193	-2.281	0.188	No
RPL37A	2282	-2.354	0.159	No
RPL4	2359	-2.421	0.135	No
ATP6V1D	2370	-2.428	0.136	No
IL1RAPL1	2374	-2.432	0.140	No
ZNF148	2434	-2.464	0.122	No
TPD52	2456	-2.487	0.119	No
ARMC8	2662	-2.842	0.047	No
PRLR	2695	-2.913	0.040	No
