# Supplementary material for: Loneliness 5 years ante-mortem is associated with disease-related differential gene expression in postmortem dorsolateral prefrontal cortex
Source: Transl Psychiatry. 2018 Jan 10;8:2. doi: 10.1038/s41398-017-0086-2 (PMC5802527; doi:10.1038/s41398-017-0086-2)
Supplement: Supplementary file 4 — Supplemental Table 4 [file 41398_2017_86_MOESM4_ESM.pdf]

| PROBE | RANK IN GENE LIST | RANK METRIC SCORE | RUNNING ES | CORE ENRICHMENT |
|---|---|---|---|---|
| VWF | 213 | 2.907 | -0.073 | No |
| AXL | 214 | 2.904 | -0.065 | No |
| FGFR3 | 263 | 2.817 | -0.076 | No |
| TFRC | 299 | 2.781 | -0.081 | No |
| S1PR1 | 544 | 2.492 | -0.167 | No |
| ABCC4 | 610 | 2.454 | -0.185 | No |
| NCAN | 719 | 2.380 | -0.219 | No |
| GPAA1 | 732 | 2.372 | -0.217 | No |
| ADI1 | 810 | 2.320 | -0.240 | No |
| ALDOC | 824 | 2.313 | -0.239 | No |
| ATP1A2 | 855 | 2.292 | -0.244 | No |
| ADCY2 | 883 | 2.275 | -0.248 | No |
| DHCR7 | 962 | 2.227 | -0.272 | No |
| GLUL | 1016 | 2.191 | -0.286 | No |
| SLC1A4 | 1384 | 2.033 | -0.419 | No |
| MAOA | 1431 | 2.014 | -0.431 | No |
| LRP8 | 1442 | 2.008 | -0.430 | No |
| DSC2 | 1457 | 2.002 | -0.430 | No |
| SEC23B | 1462 | 2.000 | -0.426 | No |
| AKAP11 | 1559 | -1.977 | -0.457 | No |
| PCDH7 | 1573 | -1.980 | -0.456 | No |
| GRIA1 | 1597 | -1.989 | -0.460 | Yes |
| RIT2 | 1603 | -1.992 | -0.456 | Yes |
| LETMD1 | 1610 | -1.994 | -0.453 | Yes |
| NELL2 | 1635 | -2.003 | -0.457 | Yes |
| STMN2 | 1644 | -2.005 | -0.454 | Yes |
| ENOPH1 | 1673 | -2.015 | -0.460 | Yes |
| KCNJ3 | 1676 | -2.016 | -0.455 | Yes |
| FAIM2 | 1689 | -2.022 | -0.454 | Yes |
| PSMA1 | 1695 | -2.024 | -0.450 | Yes |
| WDR7 | 1696 | -2.024 | -0.445 | Yes |
| CDC40 | 1697 | -2.024 | -0.439 | Yes |
| ERGIC2 | 1698 | -2.025 | -0.434 | Yes |
| PITPNB | 1704 | -2.028 | -0.430 | Yes |
| ARHGEF3 | 1705 | -2.029 | -0.425 | Yes |
| ADAM23 | 1706 | -2.029 | -0.420 | Yes |
| TERF2IP | 1708 | -2.030 | -0.414 | Yes |
| MGST3 | 1715 | -2.034 | -0.411 | Yes |
| TOP1 | 1736 | -2.045 | -0.413 | Yes |
| CDK7 | 1740 | -2.047 | -0.409 | Yes |
| GRIN2A | 1742 | -2.048 | -0.404 | Yes |
| TRIM36 | 1751 | -2.052 | -0.401 | Yes |
| HPCAL4 | 1756 | -2.053 | -0.397 | Yes |
| ATP5H | 1758 | -2.054 | -0.392 | Yes |
| REEP5 | 1759 | -2.056 | -0.387 | Yes |

| | | | | |
|---|---|---|---|---|
| MPP1 | 1770 | -2.060 | -0.385 | Yes |
| RTN4 | 1778 | -2.067 | -0.382 | Yes |
| SUMO1 | 1791 | -2.075 | -0.381 | Yes |
| ZNHIT3 | 1792 | -2.075 | -0.375 | Yes |
| B3GALT2 | 1798 | -2.077 | -0.372 | Yes |
| TRO | 1804 | -2.079 | -0.368 | Yes |
| VAMP1 | 1811 | -2.081 | -0.364 | Yes |
| MAP2K4 | 1821 | -2.085 | -0.362 | Yes |
| ZNF204P | 1837 | -2.089 | -0.362 | Yes |
| CDK14 | 1841 | -2.091 | -0.358 | Yes |
| AASDHPPT | 1871 | -2.097 | -0.363 | Yes |
| PLCL2 | 1873 | -2.097 | -0.358 | Yes |
| TXN | 1889 | -2.104 | -0.358 | Yes |
| ATP6V1H | 1906 | -2.114 | -0.358 | Yes |
| GLS | 1908 | -2.115 | -0.353 | Yes |
| TAGLN3 | 1913 | -2.119 | -0.349 | Yes |
| MATR3 | 1926 | -2.123 | -0.348 | Yes |
| CADPS | 1929 | -2.123 | -0.343 | Yes |
| B4GALNT1 | 1937 | -2.128 | -0.339 | Yes |
| UBE2B | 1940 | -2.131 | -0.335 | Yes |
| ATP8A2 | 1941 | -2.131 | -0.329 | Yes |
| GRIA3 | 1946 | -2.133 | -0.325 | Yes |
| LDB2 | 1947 | -2.135 | -0.319 | Yes |
| TMEM14A | 1972 | -2.148 | -0.322 | Yes |
| WASF1 | 1987 | -2.155 | -0.322 | Yes |
| DEAF1 | 1988 | -2.156 | -0.316 | Yes |
| PPP3R1 | 1990 | -2.156 | -0.310 | Yes |
| ELOVL4 | 2010 | -2.166 | -0.312 | Yes |
| SCN1A | 2014 | -2.167 | -0.307 | Yes |
| RIMS2 | 2016 | -2.168 | -0.302 | Yes |
| AP3B2 | 2021 | -2.170 | -0.297 | Yes |
| SLC12A5 | 2031 | -2.177 | -0.295 | Yes |
| CDH18 | 2045 | -2.185 | -0.294 | Yes |
| ATP6V1G2 | 2052 | -2.191 | -0.290 | Yes |
| LGALS8 | 2079 | -2.209 | -0.294 | Yes |
| C14orf2 | 2090 | -2.218 | -0.292 | Yes |
| METTL5 | 2091 | -2.220 | -0.286 | Yes |
| SV2B | 2101 | -2.226 | -0.283 | Yes |
| B4GALT6 | 2116 | -2.236 | -0.283 | Yes |
| ANK3 | 2119 | -2.238 | -0.277 | Yes |
| CDH10 | 2120 | -2.238 | -0.271 | Yes |
| SLC25A12 | 2124 | -2.240 | -0.266 | Yes |
| CD200 | 2130 | -2.245 | -0.262 | Yes |
| SCAMP1 | 2138 | -2.248 | -0.259 | Yes |
| DIRAS2 | 2144 | -2.252 | -0.255 | Yes |
| RUNX1T1 | 2150 | -2.256 | -0.250 | Yes |
| PAPSS1 | 2151 | -2.256 | -0.244 | Yes |

| | | | | |
|---|---|---|---|---|
| BCL11A | 2153 | -2.257 | -0.239 | Yes |
| SV2A | 2161 | -2.263 | -0.235 | Yes |
| CSE1L | 2168 | -2.266 | -0.231 | Yes |
| LAMTOR3 | 2170 | -2.268 | -0.226 | Yes |
| CNTNAP2 | 2181 | -2.276 | -0.223 | Yes |
| GABRB3 | 2195 | -2.284 | -0.222 | Yes |
| ITSN2 | 2197 | -2.288 | -0.216 | Yes |
| KCND2 | 2215 | -2.301 | -0.216 | Yes |
| ATRX | 2259 | -2.338 | -0.226 | Yes |
| GUCY1B3 | 2261 | -2.338 | -0.221 | Yes |
| SKAP2 | 2285 | -2.358 | -0.223 | Yes |
| BCL11B | 2286 | -2.360 | -0.217 | Yes |
| LPHN2 | 2295 | -2.371 | -0.213 | Yes |
| CDK5R1 | 2315 | -2.385 | -0.214 | Yes |
| BCAP29 | 2327 | -2.395 | -0.212 | Yes |
| SCG5 | 2332 | -2.398 | -0.207 | Yes |
| ACYP2 | 2334 | -2.399 | -0.201 | Yes |
| SERPINI1 | 2363 | -2.424 | -0.205 | Yes |
| ATP6V1D | 2370 | -2.428 | -0.200 | Yes |
| MARS | 2382 | -2.438 | -0.198 | Yes |
| RNMT | 2396 | -2.443 | -0.196 | Yes |
| PLCB1 | 2412 | -2.445 | -0.195 | Yes |
| NAP1L2 | 2416 | -2.448 | -0.190 | Yes |
| ARL4C | 2417 | -2.448 | -0.183 | Yes |
| PJA1 | 2420 | -2.450 | -0.178 | Yes |
| SLIT2 | 2439 | -2.468 | -0.178 | Yes |
| PRKCB | 2452 | -2.484 | -0.176 | Yes |
| PTPRT | 2469 | -2.500 | -0.175 | Yes |
| RPRM | 2477 | -2.513 | -0.171 | Yes |
| TCEAL1 | 2483 | -2.518 | -0.166 | Yes |
| DLG2 | 2486 | -2.520 | -0.160 | Yes |
| PREPL | 2497 | -2.532 | -0.157 | Yes |
| RFC5 | 2505 | -2.539 | -0.153 | Yes |
| SYT1 | 2512 | -2.546 | -0.148 | Yes |
| PSD3 | 2519 | -2.564 | -0.143 | Yes |
| ZC3H15 | 2524 | -2.570 | -0.138 | Yes |
| PUM2 | 2534 | -2.580 | -0.134 | Yes |
| LRRN3 | 2535 | -2.582 | -0.127 | Yes |
| HTR1E | 2539 | -2.589 | -0.122 | Yes |
| DNAJC12 | 2549 | -2.599 | -0.118 | Yes |
| FKBP3 | 2554 | -2.606 | -0.112 | Yes |
| EPHA7 | 2557 | -2.610 | -0.106 | Yes |
| HEXB | 2559 | -2.619 | -0.100 | Yes |
| SCN3A | 2561 | -2.624 | -0.093 | Yes |
| ELAVL4 | 2563 | -2.625 | -0.086 | Yes |
| CASD1 | 2578 | -2.654 | -0.084 | Yes |
| SLC9A6 | 2585 | -2.675 | -0.079 | Yes |

| | | | | |
|---|---|---|---|---|
| LRP12 | 2610 | -2.708 | -0.081 | Yes |
| CCT2 | 2618 | -2.743 | -0.076 | Yes |
| XK | 2619 | -2.745 | -0.069 | Yes |
| PRKAR2B | 2650 | -2.815 | -0.073 | Yes |
| ARPC1A | 2667 | -2.857 | -0.071 | Yes |
| GABBR2 | 2676 | -2.869 | -0.066 | Yes |
| CNNM1 | 2690 | -2.900 | -0.064 | Yes |
| SCN2A | 2691 | -2.901 | -0.056 | Yes |
| MYT1L | 2704 | -2.937 | -0.052 | Yes |
| MEF2C | 2711 | -2.953 | -0.047 | Yes |
| TSPYL1 | 2727 | -2.962 | -0.044 | Yes |
| MAP1A | 2750 | -3.068 | -0.044 | Yes |
| PEG3 | 2755 | -3.123 | -0.038 | Yes |
| NOS1AP | 2762 | -3.161 | -0.031 | Yes |
| ATP2B1 | 2765 | -3.174 | -0.023 | Yes |
| FBXW7 | 2769 | -3.196 | -0.016 | Yes |
| CSNK1G3 | 2776 | -3.249 | -0.010 | Yes |
| CNKSR2 | 2779 | -3.265 | -0.001 | Yes |
| PPP3CA | 2787 | -3.360 | 0.005 | Yes |
