# Supplementary material for: Loneliness 5 years ante-mortem is associated with disease-related differential gene expression in postmortem dorsolateral prefrontal cortex
Source: Transl Psychiatry. 2018 Jan 10;8:2. doi: 10.1038/s41398-017-0086-2 (PMC5802527; doi:10.1038/s41398-017-0086-2)
Supplement: Supplementary file 5 — Supplemental Table 5 [file 41398_2017_86_MOESM5_ESM.pdf]

PROBE	RANK IN GENE LIST	RANK METRIC SCORE	RUNNING ES	CORE ENRICHMENT
GRINA	477	2.553	-0.160	No
TECR	1163	2.128	-0.399	No
SERPINF1	1554	-1.976	-0.530	Yes
AKAP11	1559	-1.977	-0.520	Yes
RIT2	1603	-1.992	-0.524	Yes
NELL2	1635	-2.003	-0.524	Yes
STMN2	1644	-2.005	-0.515	Yes
PSMD7	1662	-2.010	-0.509	Yes
PISD	1666	-2.011	-0.499	Yes
GNAI1	1680	-2.018	-0.492	Yes
WDR7	1696	-2.024	-0.485	Yes
ADAM23	1706	-2.029	-0.477	Yes
MAPT	1707	-2.029	-0.465	Yes
MGST3	1715	-2.034	-0.456	Yes
GRIN2A	1742	-2.048	-0.453	Yes
REEP5	1759	-2.056	-0.447	Yes
RTN4	1778	-2.067	-0.442	Yes
BTBD3	1780	-2.069	-0.430	Yes
GARS	1782	-2.070	-0.418	Yes
GABRA1	1787	-2.072	-0.407	Yes
INPP5F	1788	-2.073	-0.395	Yes
SUMO1	1791	-2.075	-0.384	Yes
ZNHIT3	1792	-2.075	-0.372	Yes
TSPYL5	1807	-2.079	-0.365	Yes
MAP2K4	1821	-2.085	-0.357	Yes
GPD1L	1864	-2.095	-0.360	Yes
AASDHPPT	1871	-2.097	-0.350	Yes
PPP1R16B	1882	-2.101	-0.342	Yes
HIVEP2	1890	-2.105	-0.332	Yes
LDB2	1947	-2.135	-0.340	Yes
PSMA4	1974	-2.149	-0.337	Yes
RYR2	1975	-2.149	-0.325	Yes
SLITRK5	1981	-2.151	-0.314	Yes
CETN2	1999	-2.162	-0.307	Yes
SLC12A5	2031	-2.177	-0.306	Yes
TSPYL4	2039	-2.183	-0.296	Yes
SLC6A15	2086	-2.213	-0.300	Yes
SOCS5	2103	-2.228	-0.293	Yes
B4GALT6	2116	-2.236	-0.284	Yes
EFR3A	2118	-2.238	-0.271	Yes
SLC25A12	2124	-2.240	-0.260	Yes
CD200	2130	-2.245	-0.249	Yes
TRIM23	2134	-2.247	-0.237	Yes
SCAMP1	2138	-2.248	-0.225	Yes
KIF3B	2193	-2.281	-0.231	Yes

GUCY1B3	2261	-2.338	-0.242	Yes
PRKACB	2306	-2.380	-0.244	Yes
SCG5	2332	-2.398	-0.239	Yes
SERPINI1	2363	-2.424	-0.236	Yes
ATP6V1D	2370	-2.428	-0.224	Yes
ABCC5	2379	-2.436	-0.213	Yes
ZNF365	2432	-2.461	-0.217	Yes
TCEAL1	2483	-2.518	-0.221	Yes
PREPL	2497	-2.532	-0.211	Yes
SYT1	2512	-2.546	-0.201	Yes
ZC3H15	2524	-2.570	-0.190	Yes
PUM2	2534	-2.580	-0.178	Yes
SLC9A6	2585	-2.675	-0.181	Yes
RALYL	2588	-2.677	-0.166	Yes
CERS6	2595	-2.686	-0.153	Yes
LMO3	2605	-2.700	-0.140	Yes
CEP170	2606	-2.700	-0.125	Yes
CCT2	2618	-2.743	-0.113	Yes
PRKAR2B	2650	-2.815	-0.107	Yes
ANKRD46	2658	-2.836	-0.093	Yes
ARPC1A	2667	-2.857	-0.080	Yes
GABBR2	2676	-2.869	-0.066	Yes
KHDRBS3	2685	-2.892	-0.052	Yes
CAMSAP2	2738	-3.009	-0.053	Yes
DUSP3	2759	-3.136	-0.042	Yes
ATP2B1	2765	-3.174	-0.026	Yes
EIF4A2	2766	-3.175	-0.007	Yes
CNKSR2	2779	-3.265	0.008	Yes
