# Supplementary material for: Loneliness 5 years ante-mortem is associated with disease-related differential gene expression in postmortem dorsolateral prefrontal cortex
Source: Transl Psychiatry. 2018 Jan 10;8:2. doi: 10.1038/s41398-017-0086-2 (PMC5802527; doi:10.1038/s41398-017-0086-2)
Supplement: Supplementary file 6 — Supplemental Table 6 [file 41398_2017_86_MOESM6_ESM.pdf]

PROBE	RANK IN GENE LIST	RANK METRIC SCORE	RUNNING ES	CORE ENRICHMENT
LFNG	24	3.468	0.007	Yes
CSF1R	30	3.403	0.021	Yes
RAET1G	64	3.223	0.023	Yes
PROCR	82	3.154	0.032	Yes
MAVS	97	3.111	0.041	Yes
MR1	100	3.106	0.054	Yes
UNC93B1	108	3.090	0.066	Yes
NOTCH1	147	3.033	0.066	Yes
PLCG2	149	3.030	0.079	Yes
TREML1	166	2.999	0.087	Yes
AXL	214	2.904	0.083	Yes
HFE	223	2.887	0.093	Yes
IFNA16	271	2.795	0.089	Yes
MASP1	331	2.736	0.080	Yes
CTLA4	343	2.720	0.088	Yes
RAET1E	347	2.711	0.099	Yes
INPP5D	353	2.699	0.110	Yes
IGHA2	356	2.695	0.122	Yes
DBNL	387	2.651	0.123	Yes
BMP6	407	2.627	0.128	Yes
GATA2	409	2.627	0.139	Yes
IGLC3	425	2.606	0.146	Yes
FASLG	431	2.599	0.156	Yes
DEFA6	460	2.569	0.157	Yes
FCGRT	471	2.560	0.165	Yes
FCN2	504	2.527	0.165	Yes
CD58	576	2.476	0.150	Yes
APOBEC3H	584	2.471	0.159	Yes
IGLC2	595	2.465	0.167	Yes
ST6GAL1	614	2.450	0.171	Yes
CRIP1	628	2.438	0.178	Yes
IFNAR2	637	2.430	0.186	Yes
NOTCH2	640	2.430	0.196	Yes
IGHM	670	2.416	0.197	Yes
TGFBR3	699	2.397	0.197	Yes
IGKV1-5	720	2.380	0.201	Yes
RAET1L	721	2.378	0.212	Yes
GPER	730	2.372	0.220	Yes
TGFB1	733	2.371	0.230	Yes
IGKC	743	2.362	0.237	Yes
LILRB4	761	2.350	0.242	Yes
WAS	845	2.296	0.222	Yes
PTPN6	871	2.282	0.223	Yes
CST7	872	2.279	0.233	Yes
IL1R1	878	2.278	0.242	Yes

FCN3	880	2.277	0.252	Yes
IL18RAP	934	2.246	0.243	Yes
SYK	990	2.206	0.233	Yes
TYROBP	997	2.199	0.241	Yes
NOD1	1055	2.175	0.230	Yes
CTSH	1057	2.173	0.239	Yes
TAPBP	1072	2.164	0.244	Yes
TINAGL1	1089	2.156	0.248	Yes
CD40	1098	2.151	0.255	Yes
MYD88	1115	2.145	0.259	Yes
IL18R1	1117	2.144	0.268	Yes
CD7	1130	2.140	0.274	Yes
CMKLR1	1131	2.140	0.283	Yes
APOBEC3C	1158	2.129	0.284	Yes
ANXA3	1169	2.125	0.290	Yes
EMP2	1171	2.121	0.299	Yes
CD4	1189	2.114	0.302	Yes
RNF135	1197	2.111	0.309	Yes
APCS	1207	2.105	0.316	Yes
CCL17	1256	2.081	0.308	Yes
CD86	1279	2.074	0.309	Yes
TRIM38	1280	2.074	0.319	Yes
IL18	1322	2.057	0.313	Yes
CLEC4D	1387	2.032	0.299	Yes
TNFRSF1B	1405	2.023	0.302	Yes
SMAD6	1410	2.022	0.309	Yes
GTPBP1	1411	2.022	0.319	Yes
CD97	1425	2.016	0.323	Yes
ABL1	1430	2.015	0.331	Yes
NLRX1	1461	2.000	0.329	Yes
SUSD2	1469	1.998	0.336	Yes
CR1	1511	1.985	0.330	No
ANG	1539	1.977	0.329	No
CFHR5	1621	-1.997	0.308	No
COL4A3BP	1703	-2.028	0.287	No
PLCL2	1873	-2.097	0.235	No
VTCN1	1879	-2.100	0.242	No
ANKRD17	2082	-2.210	0.178	No
SEMA7A	2277	-2.350	0.117	No
PRKCB	2452	-2.484	0.064	No
ATG5	2492	-2.527	0.062	No
CPLX2	2531	-2.575	0.059	No
OPRM1	2550	-2.600	0.065	No
PPARG	2678	-2.873	0.031	No
MEF2C	2711	-2.953	0.033	No
