# Supplementary material for: Loneliness 5 years ante-mortem is associated with disease-related differential gene expression in postmortem dorsolateral prefrontal cortex
Source: Transl Psychiatry. 2018 Jan 10;8:2. doi: 10.1038/s41398-017-0086-2 (PMC5802527; doi:10.1038/s41398-017-0086-2)
Supplement: Supplementary file 7 — Supplemental Table 7 [file 41398_2017_86_MOESM7_ESM.pdf]

PROBE	RANK IN GENE LIST	RANK METRIC SCORE	RUNNING ES	CORE ENRICHMENT
ID1	19	3.522	0.008	Yes
IL13RA1	28	3.417	0.020	Yes
ST6GALNAC4	31	3.402	0.035	Yes
MYH11	44	3.316	0.045	Yes
CD34	65	3.217	0.051	Yes
MALL	66	3.214	0.065	Yes
MYL9	68	3.207	0.079	Yes
TPM2	81	3.155	0.088	Yes
ENG	86	3.146	0.101	Yes
LTBP2	93	3.127	0.112	Yes
CD151	110	3.073	0.120	Yes
RRAS	142	3.052	0.122	Yes
BCAM	158	3.013	0.129	Yes
VAMP5	171	2.994	0.138	Yes
CYB5R1	179	2.978	0.149	Yes
FBLN1	181	2.973	0.161	Yes
CSF3R	189	2.950	0.172	Yes
CES1	196	2.939	0.182	Yes
WFS1	198	2.936	0.195	Yes
PLEC	208	2.920	0.204	Yes
SLCO2B1	209	2.917	0.217	Yes
VWF	213	2.907	0.228	Yes
ANXA2	238	2.848	0.232	Yes
HSPG2	256	2.826	0.238	Yes
CAV2	261	2.819	0.249	Yes
PDGFRB	294	2.785	0.249	Yes
EPAS1	301	2.779	0.259	Yes
HEG1	302	2.778	0.272	Yes
FADS1	313	2.765	0.280	Yes
TIMP3	319	2.756	0.290	Yes
GJA1	342	2.723	0.294	Yes
MAPKAPK3	350	2.706	0.303	Yes
CAPG	373	2.678	0.307	Yes
ARHGAP4	383	2.657	0.315	Yes
GATA2	409	2.627	0.317	Yes
PTRF	416	2.617	0.327	Yes
GPSM3	433	2.593	0.332	Yes
FCGRT	471	2.560	0.330	Yes
ITGB5	484	2.546	0.336	Yes
SLC7A7	486	2.545	0.347	Yes
DPYSL3	566	2.480	0.329	Yes
RAMP2	568	2.480	0.339	Yes
CD37	575	2.477	0.348	Yes
CRIP1	628	2.438	0.340	Yes
IGHM	670	2.416	0.335	Yes

TGFBR3	699	2.397	0.335	Yes
APRT	702	2.395	0.345	Yes
COL6A3	728	2.373	0.346	Yes
TGFB1	733	2.371	0.355	Yes
DNM2	746	2.358	0.361	Yes
ITGA6	766	2.347	0.364	Yes
LAMB2	796	2.328	0.364	Yes
ALPL	807	2.321	0.370	Yes
CYP4B1	828	2.310	0.373	Yes
ACP5	844	2.297	0.377	Yes
WAS	845	2.296	0.387	Yes
LMO2	869	2.282	0.389	Yes
CST7	872	2.279	0.398	Yes
IL1R1	878	2.278	0.406	Yes
ALOX5	926	2.254	0.399	Yes
MYLK	941	2.242	0.403	Yes
DHRS3	993	2.203	0.394	Yes
CDH5	1007	2.194	0.399	Yes
CD302	1020	2.190	0.404	Yes
FGFR1	1029	2.187	0.411	Yes
SLC39A7	1034	2.185	0.419	Yes
FLNA	1062	2.171	0.418	Yes
RHOG	1067	2.166	0.426	Yes
WWTR1	1096	2.153	0.426	No
ANXA3	1169	2.125	0.408	No
EMP2	1171	2.121	0.417	No
TCIRG1	1190	2.113	0.420	No
MFAP4	1288	2.071	0.393	No
RHOB	1292	2.069	0.401	No
S100A4	1308	2.063	0.405	No
CSRP1	1354	2.044	0.397	No
PTP4A3	1395	2.029	0.391	No
TNFRSF1B	1405	2.023	0.397	No
CD97	1425	2.016	0.398	No
MAOA	1431	2.014	0.405	No
WISP2	1472	1.997	0.399	No
NEDD9	1496	1.989	0.400	No
KRT19	1533	1.979	0.395	No
SFTPC	1664	-2.010	0.356	No
TNFAIP2	1776	-2.066	0.324	No
LDB2	1947	-2.135	0.271	No
NKX2-1	2104	-2.228	0.223	No
LGMN	2258	-2.337	0.177	No
SLIT2	2439	-2.468	0.121	No
LMO3	2605	-2.700	0.072	No
